# Strain‐Release‐Driven Friedel–Crafts Spirocyclization of Azabicyclo[1.1.0]butanes

**DOI:** 10.1002/anie.202114235

**Published:** 2021-12-03

**Authors:** Jasper L. Tyler, Adam Noble, Varinder K. Aggarwal

**Affiliations:** ^1^ School of Chemistry University of Bristol Cantock's Close Bristol BS8 1TS UK

**Keywords:** azabicylo[1.1.0]butanes, azetidines, dearomatization, spiro compounds, strained molecules

## Abstract

The identification of spiro N‐heterocycles as scaffolds that display structural novelty, three‐dimensionality, beneficial physicochemical properties, and enable the controlled spatial disposition of substituents has led to a surge of interest in utilizing these compounds in drug discovery programs. Herein, we report the strain‐release‐driven Friedel–Crafts spirocyclization of azabicyclo[1.1.0]butane‐tethered (hetero)aryls for the synthesis of a unique library of azetidine spiro‐tetralins. The reaction was discovered to proceed through an unexpected interrupted Friedel–Crafts mechanism, generating a highly complex azabicyclo[2.1.1]hexane scaffold. This dearomatized intermediate, formed exclusively as a single diastereomer, can be subsequently converted to the Friedel–Crafts product upon electrophilic activation of the tertiary amine, or trapped as a Diels–Alder adduct in one‐pot. The rapid assembly of molecular complexity demonstrated in these reactions highlights the potential of the strain‐release‐driven spirocyclization strategy to be utilized in the synthesis of medicinally relevant scaffolds.

## Graphical abstract

The exploitation of the inherent strain energy of azabicyclo[1.1.0]butane for the Friedel–Crafts inspired synthesis of a unique library of azetidine‐containing spirocycles is described. The observed dearomatized intermediate of this reaction can be converted to the Friedel–Crafts product upon electrophilic activation or trapped as the Diels–Alder adduct to generate complex sp^3^‐rich architectures.

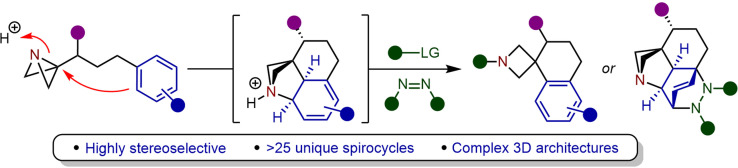

## Introduction

Nitrogen‐containing heterocycles are arguably the most significant structural components of small‐molecule pharmaceuticals. In a survey published by Paul and Kumar, it was reported that 88 % of small molecule drugs approved by the FDA between 2015 and 2020 contain one or more N‐heterocyclic units.^[1]^ Additionally, it has been recognized in recent years that increasing molecular complexity, measured by the fraction of sp^3^‐hybridized carbons (Fsp^3^), of drug candidates correlates strongly with clinical success.[^2^, ^3^]

**Figure 1:**
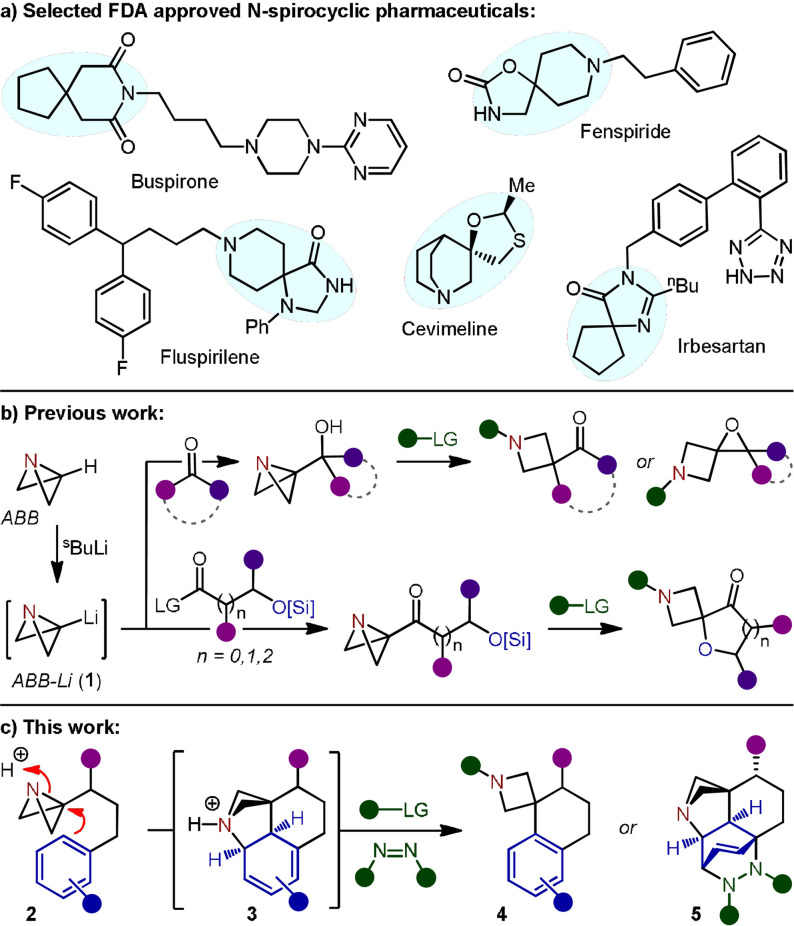
a) Selected N‐heterocyclic spirocycles in FDA approved pharmaceuticals. b) Azabicyclo[1.1.0]butane (ABB) in the synthesis of azetidine‐containing spirocycles. c) This work: strain‐release‐driven intramolecular Friedel–Crafts spirocyclization.

Spiro N‐heterocycles have been labelled as atypical scaffolds that not only have greater three‐dimensionality (Fsp^3^) than aromatic rings, but also introduce structural novelty for improved patentability (Figure 1 a).[^4^, ^5^] The limited conformational flexibility derived from a four‐membered ring, such as an azetidine, also provides a well‐defined spatial disposition of the substituents for highly predictable vectorization.^[6]^ Despite their potential, the exploration of spirocyclic chemical space is still in its infancy, due in part to the limited variety of synthetic methodologies available for their construction.^[7]^ Studies in this area typically focus on the assembly of specifically targeted molecules and, as a result, general methods for the modular synthesis of highly functionalized spirocycle‐based structures have become greatly sought‐after.^[8]^

An emerging strategy for the construction of complex heterocyclic scaffolds involves harnessing the inherent strain energy of a cyclic fragment.^[9]^ The enhanced reactivity that can be achieved through breaking strained bonds can provide the driving force to access regions of chemical space not achievable from lower energy molecules. We recently reported the application of the strained heterocycle azabicyclo[1.1.0]butane (ABB) for the synthesis of azetidine‐containing spirocycles (Figure 1 b).[^10^, ^11^] After deprotonation of the bridgehead C−H bond of ABB, the carbenoid ABB‐Li (**1**) was used in 1,2‐addition reactions with cyclic ketones or silyl ether containing Weinreb amides to access spirocyclization precursors. Subsequent electrophilic activation of the ABB nitrogen was demonstrated to trigger strain‐release‐driven spirocyclization reactions to access a variety of heterocyclic products.

After the success of the aforementioned reactions, we were eager to further extend this electrophile‐induced strain‐release cyclization strategy to the synthesis of other complex spirocyclic systems. We postulated that the Friedel–Crafts alkylation reaction, in which arenes undergo Lewis acid‐catalyzed electrophilic aromatic substitution with alkyl halides or alkenes, could serve as a template for reactivity.^[12]^ Translating this to our methodology would therefore require an ABB‐tethered aryl group that, under electrophilic activation of the aza‐bicycle, could induce the desired S_E_Ar reaction to deliver azetidine spiro‐tetralin products (Figure 1 c). This would allow the rapid construction of highly valuable spiro N‐heterocycles comprising an all‐carbon quaternary spirocentre, a synthetically challenging fragment to access.^[13]^ Herein, we report the realization of the strain‐release‐driven Friedel–Crafts spirocyclization of azabicyclo[1.1.0]butane‐tethered (hetero)aryls **2** for the synthesis of a unique library of azetidine spiro‐tetralins **4**. The reaction was discovered to proceed through an unexpected interrupted Friedel–Crafts mechanism, generating a highly complex azabicyclo[2.1.1]hexane scaffold (**3**). This dearomatized intermediate, formed exclusively as a single diastereomer, could then be subsequently converted to Friedel–Crafts product **4** upon carbamoylation of the tertiary amine, or trapped as Diels–Alder adduct **5** in one‐pot.

## Results and Discussion

**Scheme 1:**
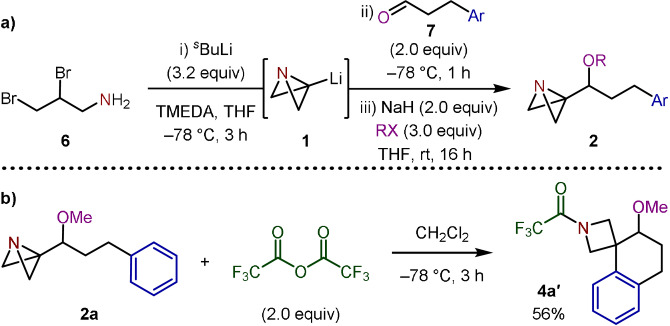
a) Preparation of ABB‐carbinol‐ethers from **1**. b) TFAA‐induced Friedel–Crafts spirocyclization of **2 a**.

We began by investigating the synthesis of spirocyclization precursors **2** through the reaction of aryl‐tethered aldehydes and ketones **7** with ABB‐Li (**1**), formed in situ from dibromo‐amine **6** (Scheme 1 a).^[14]^ Due to their instability towards chromatographic purification and the potential side reactions (semi‐pinacol rearrangement or epoxidation) that may occur upon electrophilic activation of the ABB fragment,^[10a]^ we decided to alkylate the resulting ABB‐carbinols to provide alkyl ethers **2**.

With the targeted starting materials in hand, attention then turned towards the proposed spirocyclization reaction, with ABB‐carbinol‐ether **2 a**, derived from hydrocinnamaldehyde, selected as the model substrate. We hypothesized that acylation of the ABB nitrogen with trifluoroacetic anhydride (TFAA) would make the bridgehead carbon of the ABB suitably electrophilic to trigger the desired intramolecular Friedel–Crafts reaction. Indeed, this was shown to be the case with azetidine spiro‐tetralin **4 a**′ successfully synthesized in 56 % yield (Scheme 1 b). Despite employing conditions known to promote the semi‐pinacol rearrangement for ABB‐carbinols,^[10a]^ such products were not observed from **2 a**, highlighting the importance of protecting the hydroxy group to achieve the desired reactivity. This acylation strategy limits the functionality installed on the azetidine nitrogen to that of the specific acylating agent. We therefore assessed the feasibility of employing Lewis and Brønsted acids as activators to deliver an amine intermediate that could be directly functionalized in situ and thus provide the opportunity for divergent reactivity, a desirable feature in a medicinal chemistry context.

We began by exploring the use of Lewis acids. Despite its previously reported success in promoting intramolecular Friedel–Crafts reactions,^[15]^ In(OTf)_3_ failed to yield any detectable product and instead gave a complex mixture from which no species could be identified (Table 1, entry 1). However, successful spirocyclization was observed with BF_3_⋅OEt_2_, providing **4 a** in 16 % yield after di‐*tert*‐butyl dicarbonate (Boc_2_O) protection of the amine intermediate (entry 2). Employing trifluoroacetic acid (TFA) as a Brønsted acid activator led predominantly to intermolecular addition of trifluoroacetate to the ABB with only 7 % of **4 a** observed (entry 3). We therefore sought the use of Brønsted acids with non‐nucleophilic conjugate bases to supress the competing intermolecular addition reaction. Pleasingly, TfOH, HPF_6_, and HBF_4_ all gave improved yields, with the latter proving optimum (entries 4–6). Lower loading of HBF_4_ led to reduced yields, showing that the spirocyclization reaction is not catalytic in acid (entry 7), while increasing the equivalents was found to have no significant effect on the reaction outcome (entry 8). However, during a screen of reaction solvents, a considerable improvement was observed upon changing from CH_2_Cl_2_ to CHCl_3_, providing **4 a** in a yield of 63 % (Entry 9; see Supporting Information for complete optimization results). We tested whether the Friedel–Crafts product could be generated directly from the reaction of **2 a** with Boc_2_O and Et_3_N, but no cyclization was observed in the absence of HBF_4_ (entry 10). This clearly showed that the initial spirocyclization reaction is promoted by the addition of the Brønsted acid.

**Table 1:** Optimization of the Friedel–Crafts spirocyclization reaction.^[a]^ 
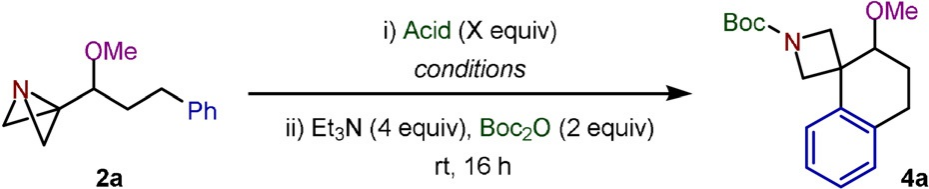

Entry	Acid	X equiv	Conditions	**4 a**: % Yield^[a]^
1	In(OTf)_3_	2.00	CH_2_Cl_2_, 0 °C–rt, 4 h	0
2	BF_3_⋅OEt_2_	1.10	CH_2_Cl_2_, 0 °C, 1.5 h	16
3	TFA	2.00	CH_2_Cl_2_, 0 °C, 1.5 h	7 (52)^[b]^
4	TfOH	2.00	CH_2_Cl_2_, 0 °C, 1.5 h	24
5	HPF_6_	1.05	CH_2_Cl_2_, 0 °C, 2 h	37
6	HBF_4_	1.05	CH_2_Cl_2_, 0 °C, 1 h	43
7	HBF_4_	0.50	CH_2_Cl_2_, 0 °C–rt, 6 h	20
8	HBF_4_	1.50	CH_2_Cl_2_, 0 °C, 1 h	41
**9**	**HBF_4_**	**1.05**	**CHCl_3_, 0 °C, 1 h**	**64 (63)** ^[c]^
10	HBF_4_	0	CHCl_3_, 0 °C, 1 h	0 (85)^[d]^

All reactions were carried out using **2 a** (0.10 mmol). [a] Yields were determined by ^1^H NMR analysis using dibromomethane as an internal standard after protection of the amine intermediate with Boc_2_O. [b] Yield of product from the intermolecular addition of trifluoroacetate to ABB. [c] 0.2 mmol scale. Isolated yield. [d] Returned **2 a**.

**Scheme 2:**
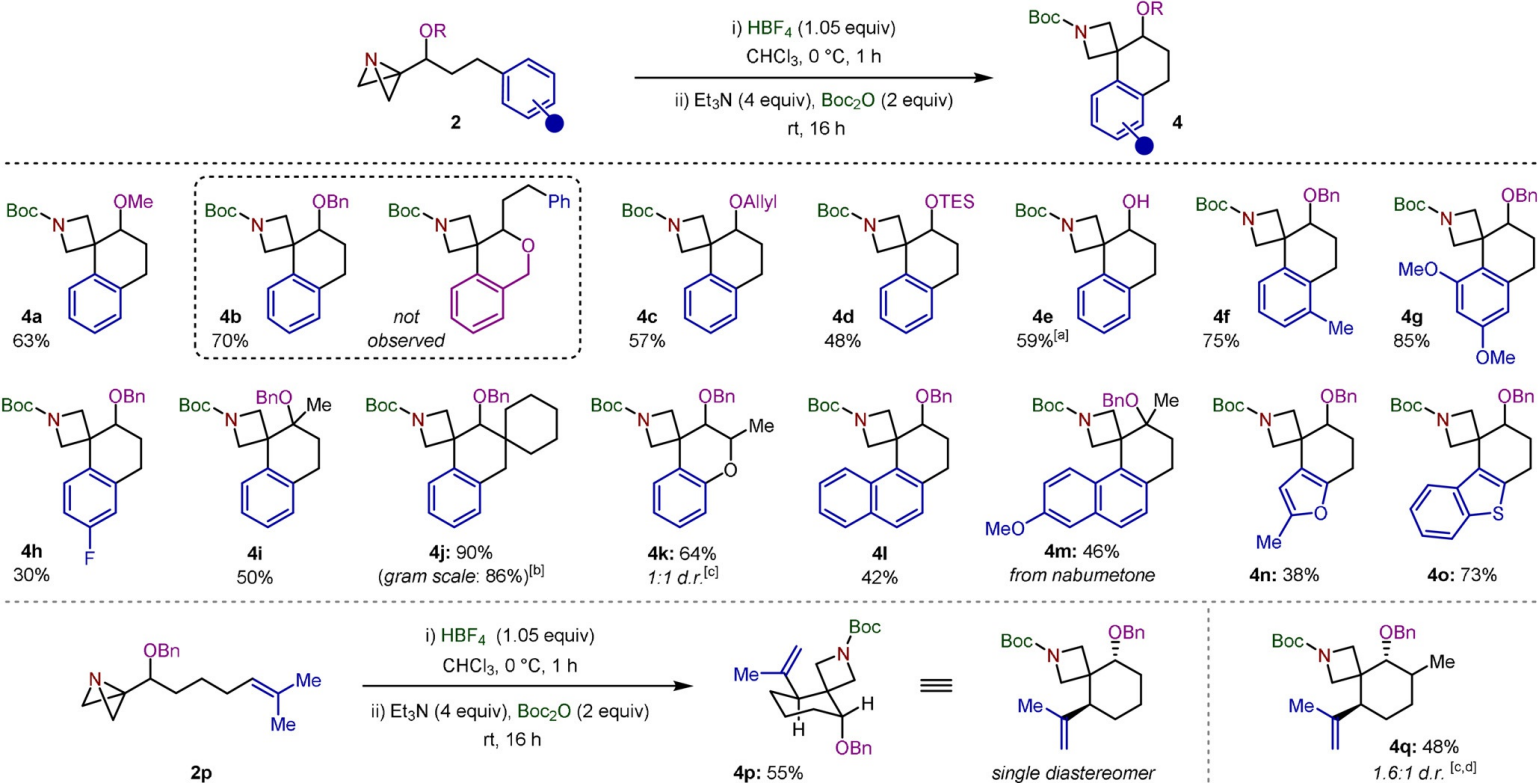
Substrate scope for the Friedel–Crafts spirocyclization reaction. All reactions were carried out using **2** (0.20 mmol) in CHCl_3_ (2.0 mL). Isolated yields given. [a] From the corresponding TMS ether. [b] Reaction was carried out using **2 j** (3.0 mmol) in CHCl_3_ (30 mL). [c] Diastereomeric ratio (d.r.) reflects that of the starting materials. [d] Diastereomers separable by column chromatography.

Having established optimal conditions, we then investigated the scope of the reaction (Scheme 2). Due to the limited synthetic utility of methyl ethers, we initially examined other alcohol protecting groups that could serve as a handle for further functionalization. Pleasingly, benzyl ether spiro‐tetralin **4 b** was synthesized in 70 % yield, providing the opportunity for alcohol deprotection orthogonal to the azetidine carbamate. Despite this protecting group also containing a potentially reactive phenyl ring with the same relationship to the ABB, complete regioselectivity for the carbon‐tethered arene was observed (see below for further discussion). As well as the benzyl functionality, allyl and triethylsilyl (TES) ether moieties could also be employed, allowing access to **4 c** and **4 d** in 57 % and 48 % yields, respectively. Interestingly, the use of the more labile trimethylsilyl (TMS) protecting group resulted in the direct formation of free alcohol **4 e**, due to concomitant silyl deprotection under the reaction conditions.

We then proceeded to explore the effect of substitution on the phenyl ring. *Ortho*‐substitution was tolerated in this reaction, delivering **4 f** in good yield. Furthermore, electron‐rich and electron‐deficient aromatic substrates underwent the desired transformation, giving **4 g** and **4 h** in yields that broadly correlated with the relative nucleophilicities of the aromatic rings (85 % and 30 %, respectively). The increasingly routine incorporation of fluorine atoms into drug candidates highlights the importance of substrate **4 h**, which was formed as a single regioisomer.

The carbon tether linking the ABB fragment to the arene was also amenable to substitution. A tertiary benzyl ether substrate derived from benzylacetone reacted smoothly to afford the corresponding spiro‐tetralin **4 i** in 50 % yield. The inclusion of a cyclohexyl ring at the homobenzylic position facilitated the synthesis of dispirocyclic compound **4 j** in excellent yield. This reaction was also amenable to scale‐up, with **4 j** isolated in comparable yield when prepared on a gram‐scale. We were pleased to find that the inclusion of heteroatoms in the tether allowed access to azetidine spirochromane **4 k** in 64 % yield, with the diastereomeric ratio (d.r.) of the product reflecting that of the ABB starting material. Novel strategies for the synthesis of chromanes are highly valuable, as they are important structural motifs present in a wide variety of bioactive natural products, pharmaceuticals, and photochromic materials.^[16]^

Encouraged by these results, we subsequently screened alternative arene nucleophiles. The 2‐naphthyl fragment was found to be a competent nucleophile in the reaction, providing **4 l** in 42 % yield. Interestingly, spirocycle **4 m**, synthesized in 46 % yield, was directly derived from the nonsteroidal anti‐inflammatory drug nabumetone. It is important to note that for these naphthalene substrates, only the depicted regioisomer was observed. Broadening the scope to heteroaromatic substrates led to the synthesis of furan and benzothiophene spirocycles **4 n** and **4 o** in 38 % and 73 % yield, respectively. Unfortunately, the elongation or contraction of the carbon tether to generate the corresponding 5 or 7‐membered rings failed to yield any detectable spirocyclic products (see Supporting Information for details).

Finally, we investigated whether olefins could be applied to this synthetic strategy to access fully saturated cyclohexyl azetidine spirocycles. Pleasingly, trisubstituted alkenes participated in the cation‐induced spirocyclization reaction and, after subsequent elimination, led to the formation of terminal alkenes **4 p** and **4 q** in moderate yields. Of most importance here is the observation that **4 p** formed as a single diastereomer, highlighting the high stereoselectivity of the acid‐induced cyclization.

**Scheme 3:**
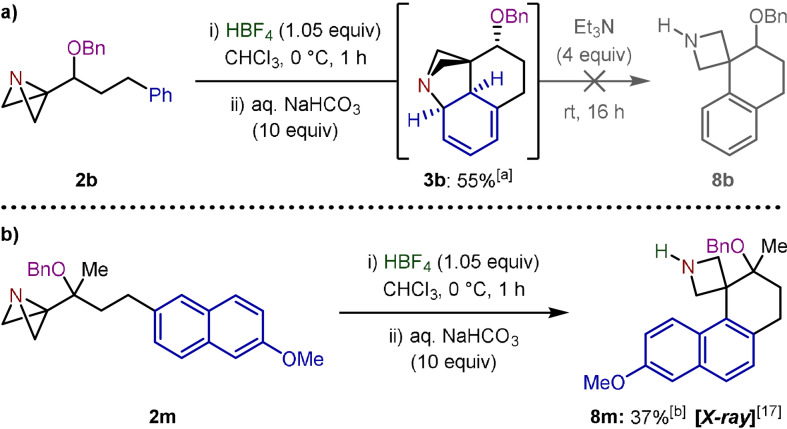
a) Observation of dearomatized diene **3 b** in the Friedel–Crafts spirocyclization reaction. b) Friedel–Crafts spirocyclization of **2 m**. [a] ^1^H NMR yield given. [b] Isolated as the HBF_4_ salt.

In an effort to exploit the modularity of this approach we then probed the feasibility of introducing groups other than *tert*‐butyl carbamate in the functionalization of the resulting azetidine. However, during attempts to quantify the initial spirocyclization reaction and directly obtain the intermediate N−H azetidine (**8 b**), an unexpected observation was made. Upon quenching the reaction with aqueous base, the only species observed was found to be dearomatized diene **3 b** (Scheme 3 a). Efforts to purify this species proved challenging due to the propensity of the molecule to rearomatize during silica gel column chromatography. The structure of **3 b** was therefore confirmed by NMR spectroscopic analysis of the crude reaction mixture (see Supporting Information for details). Although unusual, the azabicyclo[2.1.1]hexane scaffold displayed by **3 b** has been observed by Mlostoń and co‐workers, arising from the addition of 3‐phenyl‐1‐azabicyclo[1.1.0]butane to highly electron‐deficient alkenes.^[18]^ Performing the reaction under our standard conditions with Et_3_N but omitting Boc_2_O did not result in any aromatization to **8 b**, and intermediate diene **3 b** was again the only discernible species. This suggests that it is the carbamoylation of the tertiary amine that activates the system towards elimination to generate tetralin product **4 b**. Notably, this intermediate was not observed in the spirocyclization of substrate **2 m**, with Friedel–Crafts product **8 m** ^[17]^ obtained as the sole product (Scheme 3 b). In this instance, C−N bond formation is presumably disfavoured as this would require the complete dearomatization of the naphthalene ring system.

**Scheme 4:**
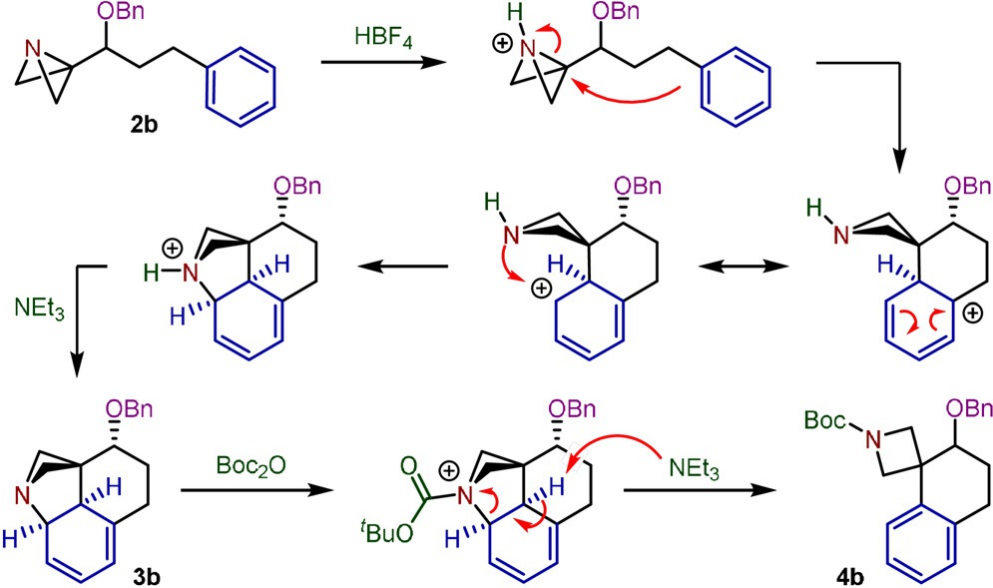
Proposed interrupted Friedel–Crafts spirocyclization mechanism in the synthesis of **4 b**.

From these results we propose the following mechanism (Scheme 4). Firstly, protonation of the ABB fragment with HBF_4_ triggers the intramolecular nucleophilic attack of the arene onto the highly electrophilic bridgehead carbon, driven by the relief of ring strain upon opening the central bond of the bicycle. The proximity of the newly formed azetidine to the resulting cationic Wheland intermediate facilitates intramolecular C−N bond formation, generating the ammonium salt of **3 b**. The formation of this pseudo‐stable diene intermediate represents a rare example of an interrupted Friedel–Crafts reaction for arene dearomatization.^[19]^ Finally, treatment with Et_3_N and subsequent carbamoylation with Boc_2_O activates the scaffold towards C−N bond cleavage and rearomatization, opening the strained azabicyclo[2.1.1]hexane framework and delivering the Friedel–Crafts spirocyclic product **4 b**.

**Scheme 5:**
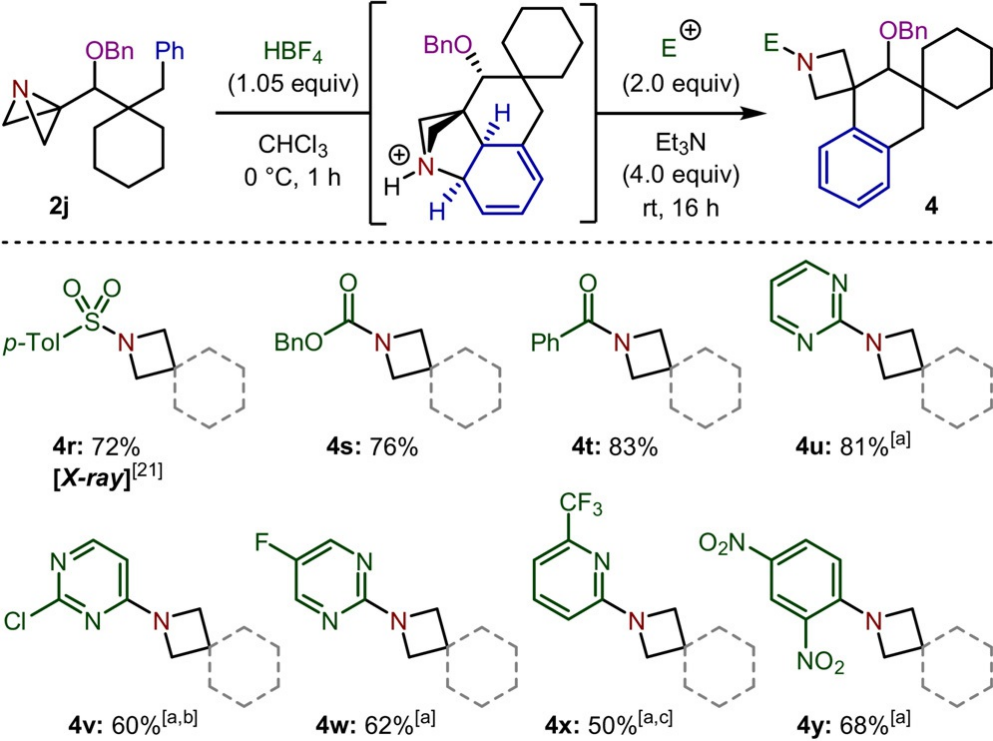
Substrate scope for the electrophile‐induced rearomatization in the Friedel–Crafts spirocyclization reaction. All reactions were carried out using **2 j** (0.20 mmol) in CHCl_3_ (2.0 mL). Isolated yields given. [a] 3.0 equiv of electrophile, heated to 50 °C for 16 h. [b] 15 % of C‐2 regioisomer detected by ^1^H NMR spectroscopy. [c] 40 h reaction time.

Having obtained a greater understanding of this Friedel–Crafts spirocyclization reaction, we then investigated other electrophiles that could trigger the rearomatization of **3** and provide alternative N‐functionalized spirocyclic products (Scheme 5). The synthesis of the corresponding sulfonamide and benzyl carbamate (**4 r** ^[21]^ and **4 s**) was achieved by employing *p*‐toluenesulfonyl chloride (TsCl) and benzyl chloroformate (CbzCl), respectively. Benzoyl amide **4 t** was also synthesized in high yield upon addition of benzoyl chloride. In 2016, Brown reported that the S_N_Ar reaction was the second‐most frequently encountered reaction in medicinal chemistry behind amide bond formation, present in 30 % of the surveyed publications.^[20]^ Due to its prevalence in drug discovery programs, high chemoselectivity and ability to rapidly install heteroaromatic fragments, we explored applying this reaction to our Friedel–Crafts spirocyclization protocol. Pleasingly, the tertiary amine intermediate could be successfully engaged in the desired S_N_Ar reaction with a variety of (hetero)aryl halides to give **4 u**–**4 y** in good yields.

**Scheme 6:**
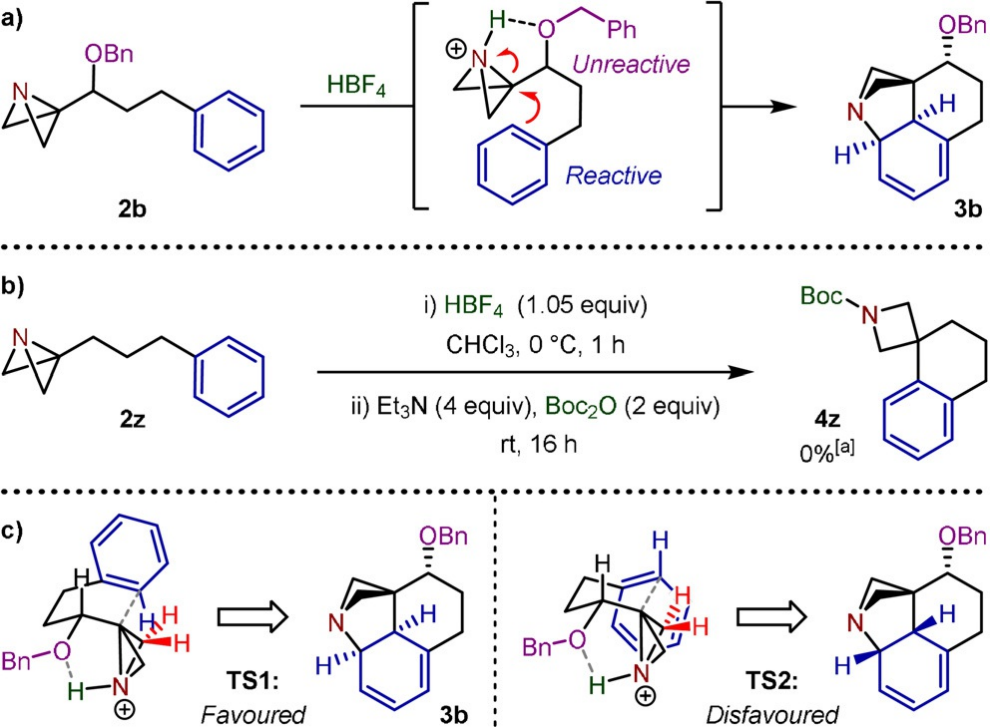
a) Regioselectivity in the Friedel–Crafts spirocyclization of **2 b**. b) Attempted spirocyclization of **2 z**. c) Diastereoselectivity in the Friedel–Crafts spirocyclization of **2 b**: proposed transition state models. [a] Complex mixture.

We were eager to establish the origin of both the regioselectivity of the benzyl ether substrates as well as the diastereoselectivity observed in this spirocyclization reaction. By analogy to the work of Floreancig and co‐workers, who reported the Lewis acid‐promoted intramolecular Friedel–Crafts alkylation of imines,^[22]^ we hypothesize that the benzyl ether fragment of **2 b** participates in hydrogen bonding to the protonated ABB (Scheme 6 a). Such an interaction prevents the aromatic ring of the benzyl ether from accessing the conformation required for cyclization and hence it is effectively inert.^[23]^ We also obtained evidence to support the key role of this hydrogen bonding interaction in facilitating the observed cyclization, with substrate **2 z** that lacks the hydrogen bond accepting ether group and fails to deliver any observable spirocycle product (Scheme 6 b). Presumably, in the absence of hydrogen bonding, a slower rate of cyclization results in decomposition pathways outcompeting the Friedel–Crafts reaction.^[22a]^ Furthermore, the intramolecular hydrogen bonding allows us to propose a model for the high diastereoselectivity observed. We postulate that **TS1**, leading to the relative stereochemistry seen in dearomatized species **3 b**, is favoured over **TS2** where the proximity of the ABB methylene to the phenyl ring in the transition state strongly disfavours its formation (Scheme 6 c).

**Scheme 7:**
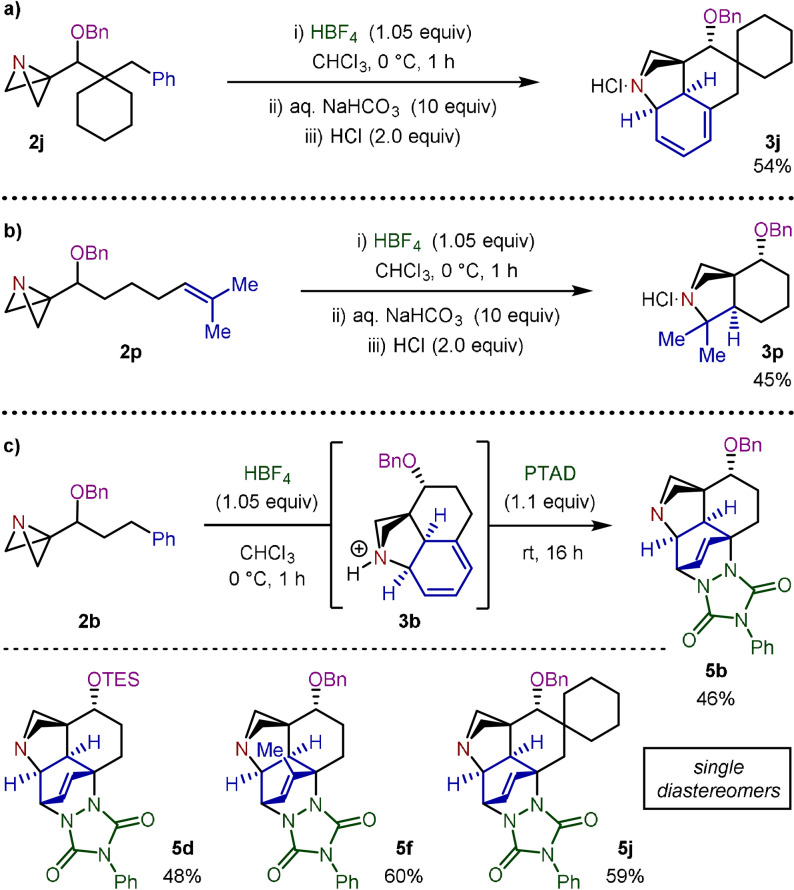
a) Isolation of dearomatized diene **3 j**. b) Formal [2+2] cycloaddition in the acid‐induced spirocyclization of **2 p**. c) One‐pot interrupted Friedel–Crafts/Diels–Alder dearomatization reaction.

Dearomatization reactions are highly sought‐after due to their ability to take feedstock aromatic systems and convert them into more complex and desirable non‐planar scaffolds.^[24]^ To investigate the potential of this stereoselective interrupted Friedel–Crafts protocol, we explored the possibility of supressing the favorable rearomatization to instead obtain the azabicyclo[2.1.1]hexane scaffold. Although unstable towards chromatographic purification, the tertiary amine intermediate from the cyclization of **2 j** could be precipitated as the hydrochloric acid salt, which allowed the straightforward isolation of the desired dearomatized species **3 j** (Scheme 7 a). We also examined the reaction of alkene substrate **2 p** in more detail and discovered that spirocyclization proceeds through a similar intermediate (**3 p**). This represents a formal [2+2] cycloaddition between the alkene π‐bond and the strained σ‐bond of the ABB (Scheme 7 b).

Finally, we looked to further react intermediate diene **3 b** in a [4+2] cycloaddition reaction. However, cyclohexadienes are notoriously poor dienes towards cycloaddition reactions due to the high distortion energies associated with accessing the transition state geometry.^[25]^ Nevertheless, 4‐phenyl‐1,2,4‐triazole‐3,5‐dione (PTAD) has been identified as a highly reactive dienophile in such systems.^[26]^ Gratifyingly, this reagent was found to generate dearomatized cycloadduct **5 b** in 46 % yield over 2 steps (Scheme 7 c). The relative stereochemistry of **5 b** was elucidated using a combination of 1D NOESY experiments and analysis of the ^1^H NMR coupling constants (see Supporting Information for full details). A selection of alternative substrates was subjected to the Friedel–Crafts/Diels–Alder conditions, generating polycyclic dearomatized compounds **5 d**, **5 f** and **5 j** in moderate to good yields. This one‐pot reaction is notable for its enormous increase in molecular complexity from simple starting materials and, despite containing 5 chiral centers, all Diels–Alder adducts were formed exclusively as single diastereomers.

## Conclusion

In summary, we have developed a novel acid‐mediated Friedel–Crafts spirocyclization reaction for the synthesis of a library of medicinally relevant azetidine spirocycles. Cyclization precursors were readily synthesized via the addition of ABB‐Li (**1**) to aldehydes and ketones followed by subsequent protection of the resulting ABB‐carbinols. Under acidic conditions, a broad range of ABB‐tethered arenes were successfully employed in this intramolecular reaction, which was also extended to diastereoselective spirocyclizations of trisubstituted alkenes. Unexpectedly, the reaction of arene substrates was found to proceed through a dearomatizing interrupted Friedel–Crafts mechanism via a complex azabicyclo[2.1.1]hexane intermediate. This species, which formed as a single diastereomer, was subsequently converted to the Friedel–Crafts product upon carbamoylation, or by engaging the tertiary amine in S_N_Ar reactions to generate highly valuable *N*‐(hetero)aryl azetidine spirocycles. The same diene‐containing intermediate was also demonstrated to undergo highly stereoselective Diels–Alder reactions with PTAD as the dienophile. The molecular complexity assembled in this one‐pot interrupted Friedel–Crafts/Diels–Alder dearomatization reaction highlights the potential of the strain‐release‐driven spirocyclization strategy to be utilized in the rapid assembly of complex sp^3^‐rich scaffolds. The development of synthetic routes towards novel building blocks, such as azetidine spiro‐tetralins, opens new vistas to access nitrogen‐containing drug‐like cores that offer significant benefits, both in terms of the placement of key binding moieties in 3D space, and in achieving improved physicochemical properties.

## Conflict of interest

The authors declare no conflict of interest.

## Supporting information

As a service to our authors and readers, this journal provides supporting information supplied by the authors. Such materials are peer reviewed and may be re‐organized for online delivery, but are not copy‐edited or typeset. Technical support issues arising from supporting information (other than missing files) should be addressed to the authors.

Supporting Information

Supporting Information
