# Supplementary material for: Strain‐Release‐Driven Friedel–Crafts Spirocyclization of Azabicyclo[1.1.0]butanes
Source: Angew Chem Int Ed Engl. 2021 Dec 3;61(3):e202114235. doi: 10.1002/anie.202114235 (PMC9299780; doi:10.1002/anie.202114235)
Supplement: Supplementary file 2 — Supporting Information [file ANIE-61-0-s002.pdf]

Angewandte Chemie
Eine Zeitschrift der Gesellschaft Deutscher Chemiker

Supporting Information

Strain-Release-Driven Friedel–Crafts Spirocyclization of Azabicyclo-[1.1.0]butanes

*Jasper L. Tyler, Adam Noble, and Varinder K. Aggarwal**

anie_202114235_sm_miscellaneous_information.pdf
anie_202114235_sm_cif.zip

TABLE OF CONTENTS

LIST OF SUPPLEMENTARY SCHEMES, FIGURES AND TABLES 2

LIST OF CHARACTERISED PRODUCTS 2

1. MATERIALS AND GENERAL METHODS 5

1.1. Glassware, Solvents and Reagents 5

1.2. Chromatography and Instrumentation 5

1.3. Naming of Compounds 5

2. EXPERIMENTAL DATA 6

2.1. Reaction Optimisation 6

2.1.1. Electrophile screen 6

2.1.2. Reaction time and temperature 6

2.1.3. Electrophile stoichiometry 7

2.1.4. Reaction solvent 8

2.2. General Procedures 8

2.2.1. **General Procedure A**: Synthesis of aldehydes from carboxylic acids 8

2.2.2. Synthesis of amine **6**: 9

2.2.3. **General Procedure B**: Synthesis of ABB-carbinols 9

2.2.4. **General Procedure C**: Alkylation of ABB-carbinols 10

2.2.5. **General Procedure D**: Silylation and acetylation of ABB-carbinols 10

2.2.6. **General Procedure E**: Friedel-Crafts spirocyclisation reaction 11

2.2.7. **General Procedure F**: Friedel-Crafts spirocyclisation/S_NAr reaction 11

2.2.8. **General Procedure G**: Acid-induced spirocyclisation of ABB-carbinol-ethers 11

2.2.9. **General Procedure H**: Interrupted Friedel-Crafts spirocyclisation/Diels-Alder reaction 12

2.3. Synthesis of aldehydes 13

2.4. Synthesis of ABB-carbinols 18

2.5. Synthesis of protected ABB-carbinols 23

2.6. Synthesis of spiro-tetralins 36

2.7. Synthesis of Friedel-Crafts intermediates 55

2.8. Synthesis of Diels-Alder adducts 57

2.9. Unsuccessful substrates 60

3. STUDIES OF THE INTERRUPTED FRIEDEL-CRAFTS MECHANISM 61

3.1. Mechanistic studies of the interrupted Friedel-Crafts mechanism 61

3.2. Determination of the structure and stereochemistry of dearomatised intermediate **3** 62

3.3. Stereochemistry of terminal alkene spirocycle **4p** 65

3.4. Unsuccessful derivatisation of dearomatised intermediate **3b** 67

4. X-RAY CRYSTALLOGRAPHIC ANALYSIS 68

4.1. **4r** (CCDC number: 2110073) and **8m** (CCDC number: 2112703) 68

5. SPECTROSCOPIC DATA 70

6. REFERENCES 143

LIST OF SUPPLEMENTARY SCHEMES, FIGURES AND TABLES

Table S1: Electrophile screen in the Friedel-Crafts spirocyclisation reaction. 6
Table S2: Time and temperature optimisation of the Friedel-Crafts spirocyclisation reaction. 6
Table S3: Electrophile stoichiometry optimisation of the Friedel-Crafts spirocyclisation 7
Table S4: Solvent optimisation of the Friedel-Crafts spirocyclisation reaction. 8
Scheme S1: Unsuccessful substrates in the Friedel-Crafts spirocyclisation reaction. 60
Scheme S2: Determination of the trigger for rearomatisation in the interrupted Friedel-Crafts spirocyclisation of **2b**. 61
Scheme S3: Friedel-Crafts spirocyclisation of **2m**. 62
Figure S1: HMBC spectrum of **3j** with key signal correlations highlighted. 63
Scheme S4: Interrupted Friedel-Crafts/Diels-Alder dearomatisation reaction for the synthesis of **5b**. 63
Figure S2: Coupling constant analysis for the stereochemical assignment of **5b**. 64
Figure S3: 1D NOESY analysis for the stereochemical assignment of **5b**. 65
Scheme S5: Observed azabicyclo[2.1.1]hexane intermediate in the spirocyclisation of **2p**. 65
Figure S4: Coupling constant analysis for the stereochemical assignment of **3p**. 66
Figure S5: Further coupling constant analysis for the stereochemical assignment of **3p**. 67
Scheme S6: Attempted derivatisation reactions of dearomatised intermediate **3b**. 67
Table S5: Crystal data and structure refinement for **4r** and **8m**. 69
Figure S6: Crystal structure of **4r** with the anisotropic displacement parameters depicted at the 50% probability level and hydrogens omitted for clarity. 69
Figure S7. Crystal structure of **8m** with the anisotropic displacement parameters depicted at the 50% probability level. Disorder on the counterion and hydrogens, except those on the heteroatom, omitted for clarity. 69

LIST OF CHARACTERISED PRODUCTS

3-(*o*-Tolyl)propanal: **7f** 13
3-(3,5-Dimethoxyphenyl)propanal: **7g** 13
3-(3-Fluorophenyl)propanal: **7h** 14
Synthesis of 1-benzylcyclohexane-1-carbaldehyde: **7j** 14
2-Phenoxypropanal: **7k** 15
Synthesis of 3-(naphthalen-2-yl)propanal: **7l** 15
3-(Benzo[*b*]thiophen-2-yl)propanal: **7o** 16
Synthesis of 6-methylhept-5-enal: **7p** 16
4-Phenylbutanal 17
1-(1-Azabicyclo[1.1.0]butan-3-yl)-3-phenylpropan-1-ol: **9a** 18
1-(1-Azabicyclo[1.1.0]butan-3-yl)-3-(*o*-tolyl)propan-1-ol: **9f** 18
1-(1-Azabicyclo[1.1.0]butan-3-yl)-3-(3,5-dimethoxyphenyl)propan-1-ol: **9g** 18
1-(1-Azabicyclo[1.1.0]butan-3-yl)-3-(3-fluorophenyl)propan-1-ol: **9h** 19
2-(1-Azabicyclo[1.1.0]butan-3-yl)-4-phenylbutan-2-ol: **9i** 19
Benzylcyclohexyl(1-azabicyclo[1.1.0]butan-3-yl)methanol: **9j** 19

1-(1-Azabicyclo[1.1.0]butan-3-yl)-2-phenoxypropan-1-ol: **9k** ... 20
1-(1-Azabicyclo[1.1.0]butan-3-yl)-3-(naphthalen-2-yl)propan-1-ol: **9l** ... 20
2-(1-Azabicyclo[1.1.0]butan-3-yl)-4-(6-methoxynaphthalen-2-yl)butan-2-ol: **9m** ... 20
1-(1-Azabicyclo[1.1.0]butan-3-yl)-3-(5-methylfuran-2-yl)propan-1-ol: **9n** ... 20
3-(Benzo[*b*]thiophen-2-yl)-1-(1-azabicyclo[1.1.0]butan-3-yl)propan-1-ol: **9o** ... 21
1-(1-Azabicyclo[1.1.0]butan-3-yl)-6-methylhept-5-en-1-ol: **9p** ... 21
1-(1-Azabicyclo[1.1.0]butan-3-yl)-2,6-dimethylhept-5-en-1-ol: **9q** ... 21
1-(1-Azabicyclo[1.1.0]butan-3-yl)-3-phenylpropan-1-one ... 21
3-(3-Phenylpropyl)-1-azabicyclo[1.1.0]butane: **2z** ... 22
1-(1-Azabicyclo[1.1.0]butan-3-yl)-2-phenylethan-1-ol ... 22
1-(1-Azabicyclo[1.1.0]butan-3-yl)-4-phenylbutan-1-ol ... 23
3-(1-Methoxy-3-phenylpropyl)-1-azabicyclo[1.1.0]butane: **2a** ... 23
3-(1-(Benzyloxy)-3-phenylpropyl)-1-azabicyclo[1.1.0]butane: **2b** ... 24
3-(1-(Allyloxy)-3-phenylpropyl)-1-azabicyclo[1.1.0]butane: **2c** ... 24
3-(3-Phenyl-1-((triethylsilyl)oxy)propyl)-1-azabicyclo[1.1.0]butane: **2d** ... 25
3-(3-Phenyl-1-((trimethylsilyl)oxy)propyl)-1-azabicyclo[1.1.0]butane: **2e** ... 25
3-(1-(Benzyloxy)-3-(*o*-tolyl)propyl)-1-azabicyclo[1.1.0]butane: **2f** ... 26
3-(1-(Benzyloxy)-3-(3,5-dimethoxyphenyl)propyl)-1-azabicyclo[1.1.0]butane: **2g** ... 27
3-(1-(Benzyloxy)-3-(3-fluorophenyl)propyl)-1-azabicyclo[1.1.0]butane: **2h** ... 27
3-(2-(Benzyloxy)-4-phenylbutan-2-yl)-1-azabicyclo[1.1.0]butane: **2i** ... 28
3-((1-Benzylcyclohexyl)(benzyloxy)methyl)-1-azabicyclo[1.1.0]butane: **2j** ... 28
3-(1-(Benzyloxy)-2-phenoxypropyl)-1-azabicyclo[1.1.0]butane: **2k** ... 29
3-(1-(Benzyloxy)-3-(naphthalen-2-yl)propyl)-1-azabicyclo[1.1.0]butane: **2l** ... 30
3-(2-(Benzyloxy)-4-(6-methoxynaphthalen-2-yl)butan-2-yl)-1-azabicyclo[1.1.0]butane: **2m** ... 30
3-(1-(Benzyloxy)-3-(5-methylfuran-2-yl)propyl)-1-azabicyclo[1.1.0]butane: **2n** ... 31
3-(3-(Benzo[*b*]thiophen-2-yl)-1-(benzyloxy)propyl)-1-azabicyclo[1.1.0]butane: **2o** ... 32
3-(1-(Benzyloxy)-6-methylhept-5-en-1-yl)-1-azabicyclo[1.1.0]butane: **2p** ... 32
3-(1-(Benzyloxy)-2,6-dimethylhept-5-en-1-yl)-1-azabicyclo[1.1.0]butane: **2q** ... 33
3-(1-(Methoxymethoxy)-3-phenylpropyl)-1-azabicyclo[1.1.0]butane ... 34
1-(1-Azabicyclo[1.1.0]butan-3-yl)-3-phenylpropyl acetate ... 34
3-(1-(Benzyloxy)-2-phenylethyl)-1-azabicyclo[1.1.0]butane ... 35
3-(1-(Benzyloxy)-4-phenylbutyl)-1-azabicyclo[1.1.0]butane ... 36
tert-Butyl 2'-methoxy-3',4'-dihydro-2'*H*-spiro[azetidine-3,1'-naphthalene]-1-carboxylate: **4a** ... 36
tert-Butyl 2'-(benzyloxy)-3',4'-dihydro-2'*H*-spiro[azetidine-3,1'-naphthalene]-1-carboxylate: **4b** ... 37
tert-Butyl 2'-(allyloxy)-3',4'-dihydro-2'*H*-spiro[azetidine-3,1'-naphthalene]-1-carboxylate: **4c** ... 38
tert-Butyl 2'-((triethylsilyl)oxy)-3',4'-dihydro-2'*H*-spiro[azetidine-3,1'-naphthalene]-1-carboxylate: **4d** ... 38
tert-Butyl 2'-hydroxy-3',4'-dihydro-2'*H*-spiro[azetidine-3,1'-naphthalene]-1-carboxylate: **4e** ... 39
tert-Butyl 2'-(benzyloxy)-5'-methyl-3',4'-dihydro-2'*H*-spiro[azetidine-3,1'-naphthalene]-1-carboxylate: **4f** ... 40
tert-Butyl 2'-(benzyloxy)-6',8'-dimethoxy-3',4'-dihydro-2'*H*-spiro[azetidine-3,1'-naphthalene]-1-carboxylate: **4g** ... 40
tert-Butyl 2'-(benzyloxy)-6'-fluoro-3',4'-dihydro-2'*H*-spiro[azetidine-3,1'-naphthalene]-1-carboxylate: **4h** ... 41
tert-Butyl 2'-(benzyloxy)-2'-methyl-3',4'-dihydro-2'*H*-spiro[azetidine-3,1'-naphthalene]-1-carboxylate: **4i** ... 42
tert-Butyl 2'-(benzyloxy)-2'*H*,4'*H*-dispiro[azetidine-3,1'-naphthalene-3',1''-cyclohexane]-1-carboxylate: **4j** ... 42
tert-Butyl 3'-(benzyloxy)-2'-methylspiro[azetidine-3,4'-chromane]-1-carboxylate: **4k** ... 43
tert-Butyl 3'-(benzyloxy)-2',3'-dihydro-1'*H*-spiro[azetidine-3,4'-phenanthrene]-1-carboxylate: **4l** ... 44

tert-Butyl 2-(benzyloxy)-2-methyl-3,4-dihydro-2*H*-spiro[anthracene-1,3'-azetidine]-1'-carboxylate: **4m** 45
tert-Butyl 5'-(benzyloxy)-2'-methyl-6',7'-dihydro-5'*H*-spiro[azetidine-3,4'-benzofuran]-1-carboxylate: **4n** 45
tert-Butyl 2'-(benzyloxy)-3',4'-dihydro-2'*H*-spiro[azetidine-3,1'-dibenzo[*b*,*d*]thiophene]-1-carboxylate: **4o** 46
tert-Butyl (5*R**,9*S**)-5-(benzyloxy)-9-(prop-1-en-2-yl)-2-azaspiro[3.5]nonane-2-carboxylate: **4p** 47
tert-Butyl-5-(benzyloxy)-6-methyl-9-(prop-1-en-2-yl)-2-azaspiro[3.5]nonane-2-carboxylate: **4q** 47
2'-(Benzyloxy)-1-tosyl-2'*H*,4'*H*-dispiro[azetidine-3,1'-naphthalene-3',1''-cyclohexane]: **4r** 48
Benzyl 2'-(benzyloxy)-2'*H*,4'*H*-dispiro[azetidine-3,1'-naphthalene-3',1''-cyclohexane]-1-carboxylate: **4s** 49
(2'-(Benzyloxy)-2'*H*,4'*H*-dispiro[azetidine-3,1'-naphthalene-3',1''-cyclohexan]-1-yl)(phenyl)methanone: **4t** 50
2'-(Benzyloxy)-1-(pyrimidin-2-yl)-2'*H*,4'*H*-dispiro[azetidine-3,1'-naphthalene-3',1''-cyclohexane]: **4u** 50
2'-(Benzyloxy)-1-(2-chloropyrimidin-4-yl)-2'*H*,4'*H*-dispiro[azetidine-3,1'-naphthalene-3',1''-cyclohexane]: **4v**.. 51
2'-(Benzyloxy)-1-(5-fluoropyrimidin-2-yl)-2'*H*,4'*H*-dispiro[azetidine-3,1'-naphthalene-3',1''-cyclohexane]: **4w**.. 52
2'-(Benzyloxy)-1-(6-(trifluoromethyl)pyridin-2-yl)-2'*H*,4'*H*-dispiro[azetidine-3,1'-naphthalene-3',1''-cyclohexane]: **4x** 53
2'-(Benzyloxy)-1-(2,4-dinitrophenyl)-2'*H*,4'*H*-dispiro[azetidine-3,1'-naphthalene-3',1''-cyclohexane]: **4y** 53
Synthesis of 2,2,2-Trifluoro-1-(2'-methoxy-3',4'-dihydro-2'*H*-spiro[azetidine-3,1'-naphthalen]-1-yl)ethan-1-one: **4a′** 54
3'-(Benzyloxy)-2a1',8a'-dihydro-2'*H*,3'*H*,5'*H*-spiro[cyclohexane-1,4'-[1,2a]methanobenzo[*cd*]indole]: **3j** 55
3'-(Benzyloxy)-7'-methoxy-3'-methyl-2',3'-dihydro-1'*H*- spiro[azetidine-3,4'-phenanthren]-1-ium tetrafluoroborate: **8m** 56
4-(Benzyloxy)-1,1- dimethyloctahydro-2,3a-methanoisoindol-2-ium chloride: **3p** 56
3-(Benzyloxy)-9-phenyl-2,3,3a1,5a-tetrahydro-1*H*,4*H*,6*H*,8*H*-6,11a-etheno-3a,5-methanopyrrolo[2,3,4-*de*][1,2,4]triazolo[1,2-*a*]cinnoline-8,10(9*H*)-dione: **5b** 57
9-Phenyl-3-((triethylsilyl)oxy)-2,3,3a1,5a-tetrahydro-1H,4H,6H,8H-6,11a-etheno-3a,5-methanopyrrolo[2,3,4-de][1,2,4]triazolo[1,2-a]cinnoline-8,10(9H)-dione: **5d** 58
3-(Benzyloxy)-12-methyl-9-phenyl-2,3,3a1,5a-tetrahydro-1H,4H,6H,8H-6,11a-etheno-3a,5-methanopyrrolo[2,3,4-de][1,2,4]triazolo[1,2-a]cinnoline-8,10(9H)-dione: **5f** 59
3'-(Benzyloxy)-9'-phenyl-3a1',5a'-dihydro-1'*H*,3'*H*,4'*H*,6'*H*,8'*H*-spiro[cyclohexane-1,2'-[6,11a]etheno[3a,5]methanopyrrolo[2,3,4-*de*][1,2,4]triazolo[1,2-*a*]cinnoline]-8',10'(9'*H*)-dione: **5j** 59

1. MATERIALS AND GENERAL METHODS

1.1. Glassware, Solvents and Reagents

All reactions were conducted under an inert atmosphere of nitrogen using standard Schlenk manifold techniques unless stated otherwise. All glassware was flame-dried prior to use.

All anhydrous solvents were commercially supplied or dried using an Anhydrous Engineering alumina column drying system (THF, hexane, toluene, Et_2O, CH_2Cl_2) and stored over 3 Å mol sieves. Reagents were purchased from commercial sources and used as received. ***Exceptions***: Trifluoroacetic anhydride (TFAA) was distilled over P_2O_5 under an inert atmosphere at standard pressure, *N*,*N*,*N′*,*N′*-tetramethylethylenediamine (TMEDA) and trimethylsilyl chloride (TMSCl) were distilled over CaH_2 under an inert atmosphere at standard pressure. *n*-Butyllithium (1.6 M in hexane) and *s*-butyllithium (1.3 M in cyclohexane/hexane (92/8)) were purchased from Acros and titrated against N-benzylbenzamide prior to use.[1]

1.2. Chromatography and Instrumentation

Thin layer chromatography (TLC) was performed to monitor reactions when practical using Merck Kieselgel 60 F254 fluorescent treated silica, which was visualised under UV light, or by staining with aqueous basic potassium permanganate followed by heating. **Flash column chromatography** (FCC) was carried out using Sigma-Aldrich silica gel (60 Å, 230–400 mesh, 40–63 µm). **NMR spectra** were recorded at various field strengths, as indicated, using Varian VNMR 400 MHz, Varian VNMR 500 MHz, or Bruker Cryo 500 MHz for ^{1}H, ^{13}C and ^{19}F acquisitions. All NMR spectra were recorder at 25 °C unless otherwise stated. Chemical shifts (δ) are reported in parts per million (ppm) and referenced to $CDCl_3$ (^{1}H: 7.26 ppm; ^{13}C: 77.16 ppm). Coupling constants (*J*) are given in Hertz (Hz) and refer to corresponding multiplicities (s = singlet, d = doublet, t = triplet, q = quartet, quin = quintet, sex = hextet, h = heptet, m = multiplet, app = apparent, br = broad signal, dd = doublet of doublets, etc.). The ^{1}H NMR spectra are reported as follows: chemical shift (multiplicity, coupling constants, number of protons, assignment). NMR assignments were made according to spin systems, using two-dimensional NMR spectroscopy (COSY, HSQC, HMBC) to assist the characterisation. Where an assignment could not be made unambiguously, no assignments are given. NMR yields were determined by ^{1}H NMR analysis using dibromomethane as an internal standard. Diastereomeric ratios (d.r.) were determined by ^{1}H NMR analysis of the crude reaction mixture. **High resolution mass spectra (HRMS)** were recorded on a Bruker Daltonics MicrOTOF II by Electrospray Ionisation (ESI) or a Thermo Scientific Orbitrap Elite by ESI or Atmospheric Pressure Chemical Ionisation (APCI). **IR spectra** were recorded neat as a thin film on a Perkin Elmer Spectrum One FT-IR. Selected absorption maxima (ν_{max}) are reported in wavenumbers (cm^{-1}).

1.3. Naming of Compounds

Compound names are those generated by ChemDraw Professional 20.0 software (PerkinElmer), following the IUPAC nomenclature.

2. EXPERIMENTAL DATA

2.1. Reaction Optimisation

Reactions were performed using **2a** (0.10 mmol) according to **General Procedure E** (*see below*). Modifications to standard conditions and key observations from each study are stated.

2.1.1. Electrophile screen

Table S1: Electrophile screen in the Friedel-Crafts spirocyclisation reaction.

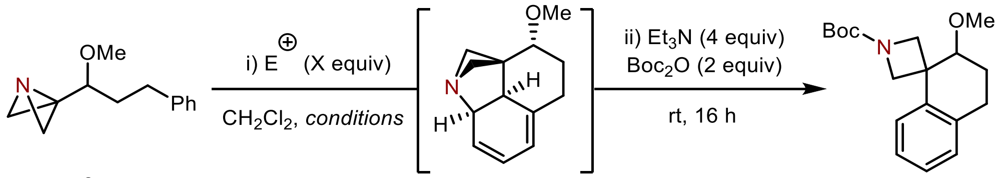

Entry	Electrophile	Conditions	% Yield[a]
**1**	$BF_3{\cdot}OEt_2$ (1.10 equiv)	0 °C, 1.5 h	16
**2**	$Sn(OTf)_3$ (2.00 equiv)	0 °C - rt, 2.5 h	trace
**3**	$In(OTf)_3$ (2.00 equiv)	0 °C - rt, 4 h	0
**4**	TFA (2.00 equiv)	0 °C, 1.5 h	7 (52)[b]
**5**	TfOH (2.00 equiv)	0 °C, 1.5 h	24
**6**	$HNTf_2$ (1.10 equiv)	0 °C, 2 h	30
**7**	$HClO_4$ (1.00 equiv)	0 °C, 2 h	21
**8**	HPF_6 (1.00 equiv)	0 °C, 2 h	37
**9**	**HBF_4 (1.05 equiv)**	**0 °C, 1 h**	**43**
**10**	**TFAA (2.00 equiv)**	**−78 °C, 3 h**	**56**[c]

[a] Yields were determined by ^{1}H NMR analysis using dibromomethane as an internal standard. [b] NMR yield of intermolecular OTFA addition. [c] 0.2 mmol scale. Without Et_3N and Boc_2O; directly forms trifluoroacetamide product **4a′**.

Key observations: Lewis acids performed poorly in the reaction with a maximum yield of 16% observed for $BF_3{\cdot}OEt_2$ (entries 1-3). In the case of TFA, trifluoroacetate addition was the major component of the reaction (entry 4). However, acids with weakly nucleophilic conjugate bases significantly improved the reaction, with HBF_4 giving the highest observed yield of 43% (entries 5-9). Although TFAA gave a superior yield of spiro-tetralin product (entry 10), the limited potential for further functionalisation meant this electrophile was not explored further.

2.1.2. Reaction time and temperature

Table S2: Time and temperature optimisation of the Friedel-Crafts spirocyclisation reaction.

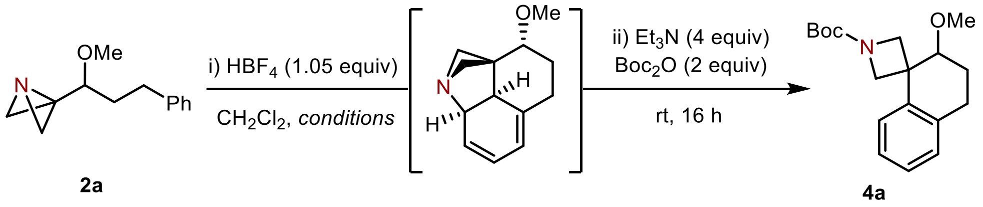

Entry	Conditions	% Yield[a]
**1**	**0 °C, 1 h**	**43**
**2**	0 °C, 6 h	40
**3**	−20 °C, 1 h	16
**4**	−20 °C, 6 h	30
**5**	−78 °C, 6 h	0

[a] Yields were determined by ^{1}H NMR analysis using dibromomethane as an internal standard.

Key observations: The reaction reached completion after 1 h at 0 °C. Lowering the temperature further had a deleterious effect on yield.

2.1.3. Electrophile stoichiometry

Table S3: Electrophile stoichiometry optimisation of the Friedel-Crafts spirocyclisation

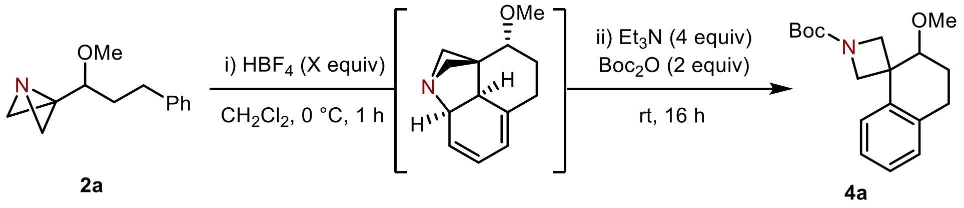

Entry	X	% Yield[a]
**1**[b]	0.20	11
**2**[b]	0.50	20
**3**	1.50	41
**4**	1.20	41
**5**	**1.05**	**43**
**6**	0	0 (85) [c]

[a] Yields were determined by ^{1}H NMR analysis using dibromomethane as an internal standard. [b] Reaction warmed to rt for 6 h. [c] Returned **2a**.

Key observations: Using sub-stoichiometric amounts of electrophile demonstrated that the spirocyclisation reaction is not catalytic in acid (entries 1-2). The reaction was found to not be sensitive towards the equivalents

of acid (entries 3-5). To show that Boc_2O does not directly activate **2a**, the reaction was performed in the absence of HBF_4 and as predicted, only returned starting material was observed (entry 6).

2.1.4. Reaction solvent

Table S4: Solvent optimisation of the Friedel-Crafts spirocyclisation reaction.

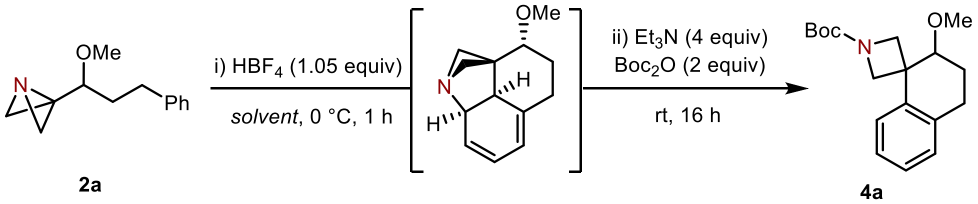

2a **4a**

Entry	Solvent	% Yield[a]
**1**	CH_2Cl_2	43
**2**	Et_2O	23
**3**	MeCN	15
**4**	DCE	34
**5**	MeOH	0
**6**	**$CHCl_3$**	**64**[b]
**7**	toluene	22
**8**	DMF	0
**9**	Nitromethane	17
**10**	EtOAc	16
**11**	Acetone	<5
**12**	*o*-DCB	32
**13**	$PhCF_3$	38

[a] Yields were determined by ^{1}H NMR analysis using dibromomethane as an internal standard. [b] 0.2 mmol scale.

Key observations: In general, halogenated solvents were the most successful in this reaction with $CHCl_3$ providing the highest observed yield.

2.2. General Procedures

2.2.1. General Procedure A: Synthesis of aldehydes from carboxylic acids

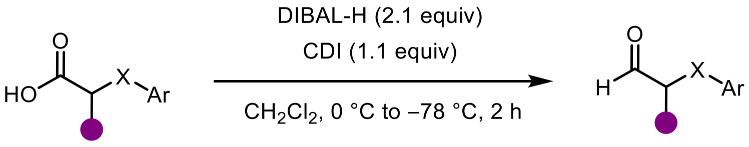

7

Following a modified literature procedure.[2]

To a solution of carboxylic acid (10.0 mmol, 1.00 equiv) in CH_2Cl_2 (50 mL, 0.2 M) at 0 °C was added 1,1′-carbonyldimidazole (CDI) (1.78 g, 1.10 equiv) and then stirred for 1 h. The resulting solution was cooled to −78 °C before the addition of DIBAL-H (1.0 M in toluene, 21 mL, 2.1 equiv) over 20 minutes *via* syringe pump. The reaction was stirred at −78 °C for 1 h before Et_2O (20 mL) was added and the solution was warmed to 0 °C. H_2O (0.84 mL) was then added followed by 15% aq. NaOH solution (0.84 mL). A further 2.1 mL of H_2O was added, the solution was warmed to rt and stirred for 15 minutes or until a white precipitate had formed. The mixture was then dried with $MgSO_4$, filtered and concentrated under reduced pressure. The crude residue was purified by flash column chromatography on silica gel to yield the corresponding aldehyde.

2.2.2. Synthesis of amine 6:

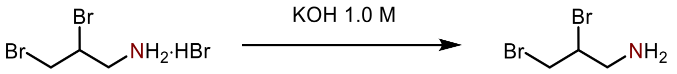

6

Following a modified literature procedure.[3] Reaction performed in the absence of light.

1-Amino-2,3-dibromopropane hydrobromide (0.75 g, 2.5 mmol) was dissolved in KOH (aq.) (1.0 M, 5 mL) and subsequently extracted with DCM (2× 5 mL). The combined organic phases were dried ($MgSO_4$), filtered, and concentrated under reduced pressure to give amine **6** (0.528 g, 2.43 mmol) as a colourless oil. The amine was immediately used for the subsequent reaction as polymerisation of **6** could be detected within 1 h at rt.

2.2.3. General Procedure B: Synthesis of ABB-carbinols

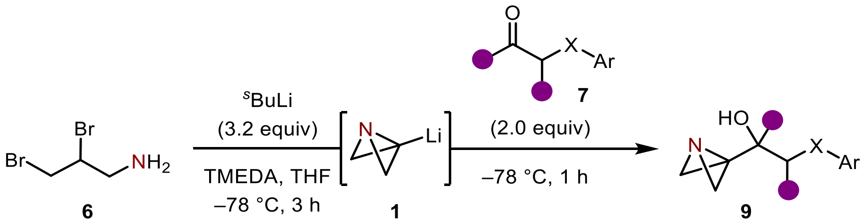

Following a modified literature procedure.[3]

To a solution of freshly made **6** (434 mg, 2.00 mmol, 1.00 equiv) and TMEDA (0.960 mL, 3.20 equiv)**[A]** in THF (5.3 mL, 0.38 M) at −78 °C was added ^{s}BuLi (1.30 M in cyclohexane/hexane (92/8), 4.92 mL, 3.20 equiv) **[B]** at a rate of 0.5 mL/min *via* syringe pump. The resulting solution was stirred for 3 h at −78 °C before the addition of the aldehyde or ketone (2.00 equiv) as a 2.0 M solution in anhydrous THF at a rate of 0.25 mL/min *via* syringe pump. The reaction was stirred at −78 °C for a final 1 h. H_2O was then added to quench the reaction, and the mixture was extracted with EtOAc (3× 20 mL). The combined organic phases were dried ($MgSO_4$), filtered, and concentrated under reduced pressure. The crude residue was used without purification in the subsequent reaction.**[C]**

Notes: (**A**) TMEDA should be distilled over CaH_2 prior to use. (**B**) Organolithiums should be carefully titrated prior to use. (**C**) ABB-carbinols were observed to be unstable towards silica gel and so are unsuitable for

purification by standard column chromatography. ABB-containing compounds can be directly visualised on silica gel thin-layer chromatography (TLC) plates as a dark yellow spot that appears after prolonged heating in the absence of a stain.

2.2.4. General Procedure C: Alkylation of ABB-carbinols

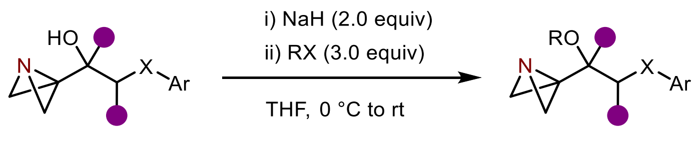

To a solution of NaH (2.00 equiv)[A] in THF (0.2 M) at 0 °C was added crude ABB-carbinol (1.00 equiv) in THF (0.2 M). The resulting solution was stirred for 15 mins at 0 °C before alkyl halide (3.00 equiv) was added and the solution stirred at rt until full consumption of the starting material was observed.[B] NH_4Cl (sat. aq.) was then added to quench the reaction, and the mixture was extracted with EtOAc (3× 20 mL). The combined organic phases were dried ($MgSO_4$), filtered, and concentrated under reduced pressure. The resulting residue was purified by flash column chromatography on silica gel to yield the corresponding protected ABB-carbinol.[C]

Notes: (**A**) Sodium hydride, 60% dispersion in mineral oil was washed with dry hexane (3× 5 mL) prior to use. (**B**) If full conversion of the starting material was not observed after 16 h at rt then a further 2 equiv of each reagent was added to the reaction. (**C**) ABB-containing compounds can be directly visualised on silica gel thin-layer chromatography (TLC) plates as a dark yellow spot that appears after prolonged heating in the absence of a stain.

2.2.5. General Procedure D: Silylation and acetylation of ABB-carbinols

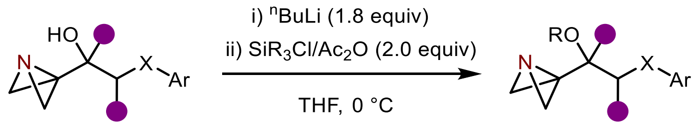

To a solution of crude ABB-carbinol (1.00 equiv) in THF (0.1 M) at 0 °C was added ^{n}BuLi (1.6 M in hexane, 1.80 equiv) dropwise.[A] The resulting solution was stirred for 10 mins at 0 °C before silyl chloride or acetic anhydride (2.00 equiv) was added and the solution stirred at 0 °C until full consumption of the starting material was observed.[B] H_2O was then added to quench the reaction, and the mixture was extracted with EtOAc (3× 20 mL). The combined organic phases were dried ($MgSO_4$), filtered, and concentrated under reduced pressure. The resulting residue was purified by flash column chromatography on silica gel to yield the corresponding protected ABB-carbinol.[C]

Notes: (**A**) Organolithiums should be carefully titrated prior to use. (**B**) If full conversion of the starting material was not observed after 3 h at 0 °C then a further 1 equiv of each reagent was added to the reaction. (**C**) ABB-containing compounds can be directly visualised on silica gel thin-layer chromatography (TLC) plates as a dark

yellow spot that appears after prolonged heating in the absence of a stain.

2.2.6. General Procedure E: Friedel-Crafts spirocyclisation reaction

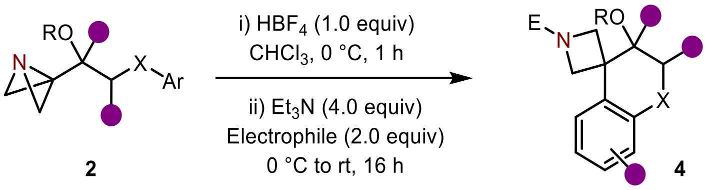

To a stirred solution of protected ABB-carbinol (0.200 mmol, 1.00 equiv) in anhydrous $CHCl_3$ (2 mL) at 0 °C was added $HBF_4{\cdot}OEt_2$ (28.6 µL, 1.05 equiv) dropwise. The solution was then stirred at 0 °C for 1 h before the addition of Et_3N (0.112 mL, 4.00 equiv) and electrophile (2.00 equiv). The resulting solution was left in an ice bath to warm to rt overnight, diluted with H_2O and extracted with EtOAc (3× 10 mL). The combined organic phases were dried ($MgSO_4$), filtered, and concentrated under reduced pressure. The residue was purified by flash column chromatography on silica gel to yield the corresponding spirocyclic product.

2.2.7. General Procedure F: Friedel-Crafts spirocyclisation/S_NAr reaction

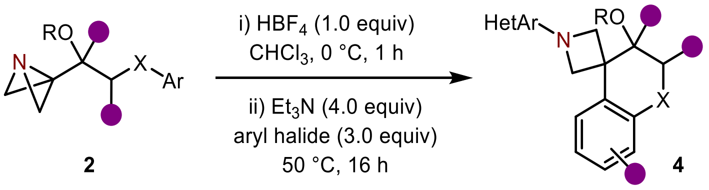

To a stirred solution of protected ABB-carbinol (0.200 mmol, 1.00 equiv) in anhydrous $CHCl_3$ (2 mL) at 0 °C was added $HBF_4{\cdot}OEt_2$ (28.6 µL, 1.05 equiv) dropwise. The solution was then stirred at 0 °C for 1 h before the addition of Et_3N (0.112 mL, 4.00 equiv) and (hetero)aryl halide (3.00 equiv). The resulting solution was heated to 50 °C and stirred overnight, cooled to rt, diluted with H_2O and extracted with EtOAc (3× 10 mL). The combined organic phases were dried ($MgSO_4$), filtered, and concentrated under reduced pressure. The residue was purified by flash column chromatography on silica gel to yield the corresponding spirocyclic product.

2.2.8. General Procedure G: Acid-induced spirocyclisation of ABB-carbinol-ethers

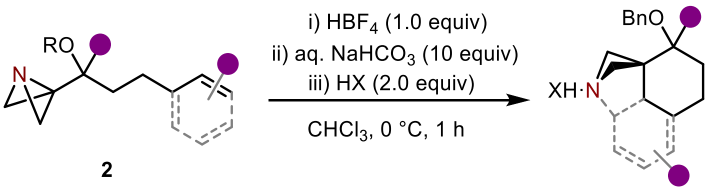

To a stirred solution of protected ABB-carbinol (0.200 mmol, 1.00 equiv) in anhydrous $CHCl_3$ (2 mL) at 0 °C was added $HBF_4{\cdot}OEt_2$ (28.6 µL, 1.05 equiv) dropwise. The solution was then stirred at 0 °C for 1 h before the reaction was quenched upon addition of aq. $NaHCO_3$ (1.0 M, 2.0 mL, 10 equiv). The resulting solution was diluted with

H_2O and extracted with EtOAc (3× 10 mL). The combined organic phases were dried ($MgSO_4$), filtered, and concentrated under reduced pressure. The residue was then dissolved in Et_2O and precipitated as the ammonium salt upon addition of acid (2 equiv). The resulting solid was collected by filtration and washed with Et_2O.

2.2.9. General Procedure H: Interrupted Friedel-Crafts spirocyclisation/Diels-Alder reaction

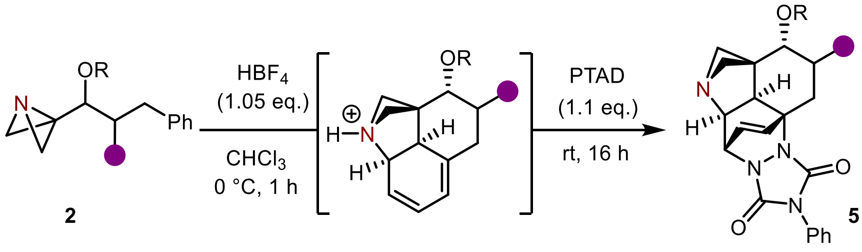

To a stirred solution of protected ABB-carbinol (0.200 mmol, 1.00 equiv) in anhydrous $CHCl_3$ (2 mL) at 0 °C was added $HBF_4{\cdot}OEt_2$ (28.6 µL, 1.05 equiv) dropwise. The solution was then stirred at 0 °C for 1 h before the addition of 4-phenyl-1,2,4-triazoline-3,5-dione (PTAD) (38.5 mg, 1.10 equiv). The resulting solution was left in an ice bath to warm to rt overnight before the addition of aq. $NaHCO_3$ (1.0 M, 2.0 mL, 10 equiv). The resulting solution was then diluted with H_2O and extracted with EtOAc (3× 10 mL). The combined organic phases were dried ($MgSO_4$), filtered, and concentrated under reduced pressure. The residue was purified by flash column chromatography on Et_3N-deactivated silica gel or was dissolved in Et_2O and precipitated as the ammonium salt upon addition of HCl (2.0 M in Et_2O, 0.2 mL, 2 equiv). The resulting solid was collected by filtration and washed with Et_2O before revealing the free-base upon washing with sat. aq. $NaHCO_3$ and concentrating under reduced pressure.

2.3. Synthesis of aldehydes

3-(*o*-Tolyl)propanal: 7f

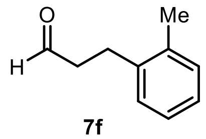

7f

Synthesised according to **General Procedure A** from: 3-(*o*-Tolyl)propanoic acid (1.64 g, 10.0 mmol, 1.00 equiv), CDI (1.78 g, 1.10 equiv) and DIBAL-H (1.0 M in toluene, 21.0 mL, 2.10 equiv). Purified by flash column chromatography (SiO_2; 92:8 pentane:Et_2O) to afford **7f** (873 mg, 5.89 mmol, 59%) as a colourless oil.

TLC: R_f = 0.28 (92:8 pentane:Et_2O).

NMR Spectroscopy (<u>*see spectra*</u>):

^{1}**H NMR** (400 MHz, $CDCl_3$): δ_H 9.85 (t, *J* = 1.4 Hz, 1H, *H*(C=O)), 7.22 – 7.07 (m, 4H, ArC*H*), 2.96 (d, *J* = 7.5 Hz, 2H, (C=O)CH_2CH_2), 2.80 – 2.67 (m, 2H, (C=O)CH_2CH_2), 2.32 (s, 3H, ArCH_3) ppm;

^{13}**C NMR** (126 MHz, $CDCl_3$): δ_C 201.8 (H(*C*=O)), 138.6 (Ar*C*), 136.1 (Ar*C*), 130.5 (Ar*C*H), 128.6 (Ar*C*H), 126.6 (Ar*C*H), 126.4 (Ar*C*H), 44.2 ((C=O)CH_2CH_2), 25.6 ((C=O)CH_2CH_2), 19.4 (ArCH_3) ppm.

All characterisation data are consistent with that reported in the literature.[4]

3-(3,5-Dimethoxyphenyl)propanal: 7g

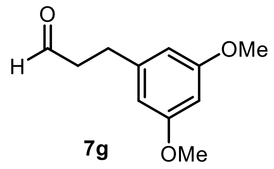

7g

Synthesised according to **General Procedure A** from: 3-(3,5-Dimethoxyphenyl)propanoic acid (2.10 g, 10.0 mmol, 1.00 equiv), CDI (1.78 g, 1.10 equiv) and DIBAL-H (1.0 M in toluene, 21.0 mL, 2.10 equiv). Purified by flash column chromatography (SiO_2; 70:30 hexane:EtOAc) to afford **7g** (1.15 g, 5.92 mmol, 59%) as a colourless oil.

TLC: R_f = 0.22 (70:30 hexane:EtOAc).

NMR Spectroscopy (<u>*see spectra*</u>):

^{1}**H NMR** (400 MHz, $CDCl_3$): δ_H 9.82 (t, *J* = 1.4 Hz, 1H, *H*(C=O)), 6.45 – 6.21 (m, 3H, ArC*H*), 3.78 (s, 6H, ArOCH_3), 2.90 (t, *J* = 7.5 Hz, 2H, (C=O)CH_2CH_2), 2.81 – 2.74 (m, 2H, (C=O)CH_2CH_2) ppm;

^{13}**C NMR** (126 MHz, $CDCl_3$): δ_C 201.6 (H(*C*=O)), 161.1 (Ar$COCH_3$), 142.9 (Ar*C*), 106.5 (Ar*C*H), 98.3 (Ar*C*H), 55.4 (ArOCH_3), 45.2 ((C=O)CH_2CH_2), 28.6 ((C=O)CH_2CH_2) ppm.

All characterisation data are consistent with that reported in the literature.[5]

3-(3-Fluorophenyl)propanal: 7h

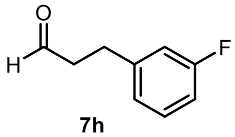

7h

Synthesised according to **General Procedure A** from: 3-(3-Fluorophenyl)propanoic acid (1.68 g, 10.0 mmol, 1.00 equiv), CDI (1.78 g, 1.10 equiv) and DIBAL-H (1.0 M in toluene, 21.0 mL, 2.10 equiv). Purified by flash column chromatography (SiO_2; 92.5:7.5 pentane:Et_2O) to afford **7h** (910 g, 5.98 mmol, 60%) as a colourless oil.

TLC: R_f = 0.28 (92.5:7.5 pentane:Et_2O).

NMR Spectroscopy (*see spectra*):

> ^{1}**H NMR** (400 MHz, $CDCl_3$): δ_H 9.82 (s, 1H, *H*(C=O)), 7.31 – 7.19 (m, 1H, ArC*H*), 6.97 (d, *J* = 7.6 Hz, 1H, ArC*H*), 6.94 – 6.86 (m, 2H, ArC*H*), 2.96 (t, *J* = 7.5 Hz, 2H, (C=O)CH_2CH_2), 2.79 (t, *J* = 7.5 Hz, 2H, (C=O)C$H_2$$CH_2$) ppm;

> ^{13}**C NMR** (126 MHz, $CDCl_3$): δ_C 201.1 (H(*C*=O)), 163.1 (d, *J* = 245.8 Hz, Ar*C*F), 143.0 (d, *J* = 7.3 Hz, Ar*C*), 130.2 (d, *J* = 8.4 Hz, Ar*C*H), 124.1 (d, *J* = 2.8 Hz, Ar*C*H), 115.4 (d, *J* = 21.1 Hz, Ar*C*H), 113.4 (d, *J* = 21.0 Hz, Ar*C*H), 45.1 ((C=O)*C*$H_2$$CH_2$), 27.9 ((C=O)$CH_2$*C*$H_2$) ppm.

All characterisation data are consistent with that reported in the literature.[6]

Synthesis of 1-benzylcyclohexane-1-carbaldehyde: 7j

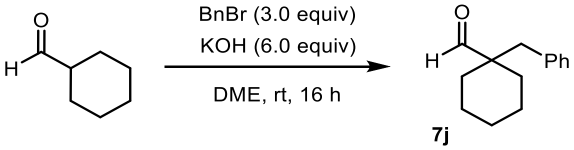

7j

Following a modified literature procedure.[7]

To a stirred suspension of freshly ground KOH (3.37 g, 6.00 equiv) in anhydrous DME (30 mL) was added benzyl bromide (3.56 mL, 3.00 equiv) and cyclohexanecarbaldehyde (1.12 g, 10.0 mmol, 1.00 equiv). The solution was then stirred at rt overnight. After this time, excess KOH was filtered off, rinsing with CH_2Cl_2, and the filtrate was added to 2 M aq. HCl (20 mL). The organic phase was separated, and the aqueous phase was extracted with CH_2Cl_2 (3× 50 mL). The combined organic phases were dried ($MgSO_4$), filtered, and concentrated under reduced pressure. The crude residue was purified by flash column chromatography (SiO_2; 97:3 pentane:Et_2O) to afford **7j** (1.57 g, 7.76 mmol, 78%) as a colourless oil.

TLC: R_f = 0.32 (97:3 pentane:Et_2O).

NMR Spectroscopy (*see spectra*):

> ^{1}**H NMR** (500 MHz, $CDCl_3$): δ_H 9.52 (s, 1H, *H*(C=O)), 7.24 – 7.15 (m, 3H, ArC*H*), 7.10 – 7.02 (m, 2H, ArC*H*), 2.72 (s, 2H, ArCH_2), 1.97 – 1.87 (m, 2H, cy-CH_2), 1.67 – 1.57 (m, 3H, cy-CH_2), 1.37 – 1.13 (m, 5H, cy-CH_2) ppm;

^{13}C NMR (126 MHz, $CDCl_3$): δ_C 207.5 (H(*C*=O)), 136.4 (Ar*C*), 130.4 (Ar*C*H), 128.3 (Ar*C*H), 126.7 (Ar*C*H), 50.8 (cy-*C*(C=O)), 43.7 (Ar*C*H_2), 31.3 (cy-*C*H_2), 25.7 (cy-*C*H_2), 22.8 (cy-*C*H_2) ppm.

All characterisation data are consistent with that reported in the literature.[7]

2-Phenoxypropanal: 7k

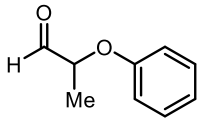

7k

Synthesised according to **General Procedure A** from: 2-Phenoxypropanoic acid (1.66 g, 10.0 mmol, 1.00 equiv), CDI (1.78 g, 1.10 equiv) and DIBAL-H (1.0 M in toluene, 21.0 mL, 2.10 equiv). Purified by flash column chromatography (SiO_2; 90:10 hexane:EtOAc) to afford **7k** (657 g, 4.37 mmol, 44%) as a colourless oil.

TLC: R_f = 0.24 (90:10 hexane:EtOAc).

NMR Spectroscopy (*see spectra*):

^{1}H NMR (400 MHz, $CDCl_3$): δ_H 9.73 (d, *J* = 2.0 Hz, 1H, *H*(C=O)), 7.33 – 7.27 (m, 2H, ArC*H*), 7.07 – 6.97 (m, 1H, ArC*H*), 6.93 – 6.85 (m, 2H, ArC*H*), 4.64 (qd, *J* = 6.9, 2.0 Hz, 1H, (C=O)C*H*), 1.49 (d, *J* = 6.9 Hz, 3H, CH(C*H*$_3$)) ppm;

^{13}C NMR (126 MHz, $CDCl_3$): δ_C 202.7 (H(*C*=O)), 157.4 (Ar*C*O), 129.9 (Ar*C*H), 122.0 (Ar*C*H), 115.4 (Ar*C*H), 77.9 ((C=O)*C*H), 15.7 (CH(*C*H$_3$)) ppm.

All characterisation data are consistent with that reported in the literature.[8]

Synthesis of 3-(naphthalen-2-yl)propanal: 7l

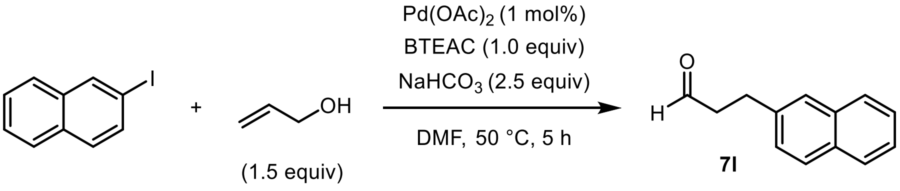

Following a modified literature procedure.[9]

To a stirred solution of 2-iodonaphthalene (1.52 g, 6.00 mmol, 1.00 equiv) in anhydrous DMF (24 mL) was added $Pd(OAc)_2$ (13.5 mg, 1.00 mol%), benzyltriethylammonium chloride (BTEAC) (1.37 g, 1.00 equiv), $NaHCO_3$ (1.26 g, 2.50 equiv) and allyl alcohol (0.612 mL, 1.50 equiv). The solution was then stirred at 50 °C for 5 h. After this time, the reaction mixture was filtered through a short plug of silica, rinsing with EtOAc, and the filtrate was added to H_2O (10 mL). The organic phase was separated and washed with H_2O (2× 20 mL). The organic phase was then dried ($MgSO_4$), filtered, and concentrated under reduced pressure. The crude residue was purified by flash column chromatography (SiO_2; 90:10 pentane:Et_2O) to afford **7l** (743 mg, 4.03 mmol, 67%) as a pale yellow oil.

TLC: R_f = 0.25 (90:10 pentane:Et_2O).

NMR Spectroscopy (see spectra):

^{1}H NMR (400 MHz, $CDCl_3$): δ_H 9.87 (t, *J* = 1.4 Hz, 1H, *H*(C=O)), 7.90 – 7.73 (m, 3H, ArC*H*), 7.64 (s, 1H, ArC*H*), 7.53 – 7.41 (m, 2H, ArC*H*), 7.33 (dd, *J* = 8.4, 1.8 Hz, 1H, ArC*H*), 3.13 (t, *J* = 7.6 Hz, 2H, (C=O)CH_2CH_2), 2.88 (td, *J* = 7.6, 1.4 Hz, 2H, (C=O)CH_2CH_2) ppm;

^{13}C NMR (126 MHz, $CDCl_3$): δ_C 201.6 (H(*C*=O)), 137.9 (Ar*C*), 133.7 (Ar*C*), 132.3 (Ar*C*), 128.4 (Ar*C*H), 127.8 (Ar*C*H), 127.6 (Ar*C*H), 127.0 (Ar*C*H), 126.6 (Ar*C*H), 126.3 (Ar*C*H), 125.6 (Ar*C*H), 45.3 ((C=O)CH_2CH_2), 28.4 ((C=O)CH_2CH_2) ppm.

All characterisation data are consistent with that reported in the literature.[9]

3-(Benzo[*b*]thiophen-2-yl)propanal: 7o

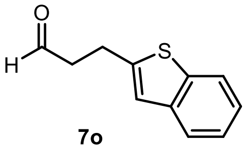

7o

Synthesised according to **General Procedure A** from: 3-Benzo[*b*]thiophen-2-yl)propanoic acid (2.06 g, 10.0 mmol, 1.00 equiv), CDI (1.78 g, 1.10 equiv) and DIBAL-H (1.0 M in toluene, 21.0 mL, 2.10 equiv). Purified by flash column chromatography (SiO_2; 90:10 pentane:Et_2O) to afford **7o** (1.05 g, 5.52 mmol, 55%) as a colourless oil.

TLC: R_f = 0.30 (90:10 pentane:Et_2O).

NMR Spectroscopy (see spectra):

^{1}H NMR (400 MHz, $CDCl_3$): δ_H 9.86 (t, *J* = 1.1 Hz, 1H, *H*(C=O)), 7.76 (d, *J* = 8.0 Hz, 1H, ArC*H*), 7.67 (d, *J* = 7.0 Hz, 1H, ArC*H*), 7.36 – 7.21 (m, 2H, ArC*H*), 7.04 (s, 1H, ArC*H*), 3.26 (t, *J* = 7.4, 2H, (C=O)CH_2CH_2), 2.93 (td, *J* = 7.4, 1.1 Hz, 2H, (C=O)CH_2CH_2) ppm;

^{13}C NMR (126 MHz, $CDCl_3$): δ_C 200.7 (H(*C*=O)), 144.0 (Ar*C*), 140.1 (Ar*C*), 139.5 (Ar*C*), 124.4 (Ar*C*H), 124.0 (Ar*C*H), 123.1 (Ar*C*H), 122.3 (Ar*C*H), 121.5 (Ar*C*H), 44.9 ((C=O)CH_2CH_2), 23.4 ((C=O)CH_2CH_2) ppm.

All characterisation data are consistent with that reported in the literature.[10]

Synthesis of 6-methylhept-5-enal: 7p

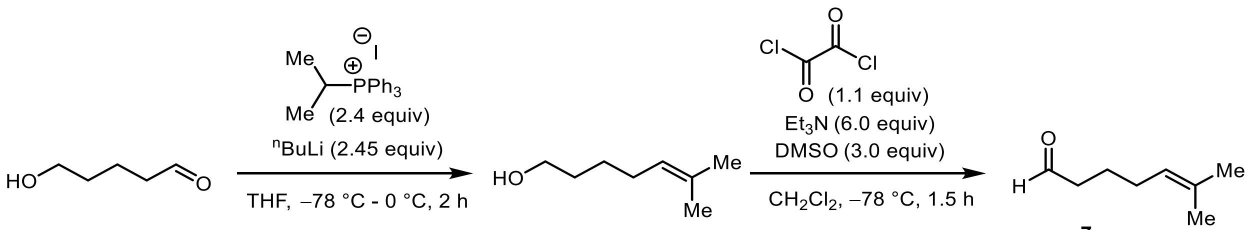

7p

Following modified literature procedures.[11,12]

To a stirred solution of isopropyltriphenylphosphonium iodide (15.6 g, 2.40 equiv) in anhydrous THF (83 mL) at 0 °C was added ^{n}BuLi (1.6 M in hexane, 23.0 mL, 2.45 equiv) at a rate of 1 mL/min *via* syringe pump. The reaction was stirred for 5 minutes then cooled to −78 °C. A solution 5-hydroxypentanal (1.45 mL, 15.0 mmol, 1.00 equiv) in THF (30 mL) was added at a rate of 2 mL/min *via* syringe pump. The solution was then stirred at −78 °C for 30 minutes before being transferred to an ice bath and warmed to 0 °C over 1 h. Sat. aq. NH_4Cl (50 mL) was then added to quench the reaction, and the mixture was extracted with EtOAc (3× 50 mL). The combined organic phases were dried ($MgSO_4$), filtered, and concentrated under reduced pressure. The crude residue was filtered through a short plug of silica, eluting with 4:1 pentane:Et_2O, and concentrated under reduced pressure.

To a stirred solution of oxalyl chloride (2 M in CH_2Cl_2, 3.85 mL, 1.10 equiv) in anhydrous CH_2Cl_2 (8.4 mL) at −78 °C was added DMSO (1.49 mL, 3.00 equiv) in CH_2Cl_2 (8.4 mL). The solution was stirred for 10 minutes before a solution of crude alcohol (7.00 mmol, 1.00 equiv) in CH_2Cl_2 (12 mL) was added at a rate of 0.8 mL/min *via* syringe pump. The solution was stirred for 30 minutes before the addition of Et_3N (5.85 mL, 1.00 equiv). The solution was stirred for a final 30 minutes before H_2O was added and the reaction warmed to rt. The organic phase was separated, and the aqueous phase was extracted with CH_2Cl_2 (3× 50 mL). The combined organic phases were dried ($MgSO_4$), filtered, and concentrated under reduced pressure. The crude residue was purified by flash column chromatography (SiO_2; 95:5 pentane:Et_2O) to afford **7p** (636 mg, 5.04 mmol, 72%) as a colourless liquid.

TLC: R_f = 0.25 (95:5 pentane:Et_2O).

NMR Spectroscopy (*see spectra*):

^{1}H NMR (400 MHz, $CDCl_3$): δ_H 9.76 (t, *J* = 1.8 Hz, 1H, *H*(C=O)), 5.08 (t, *J* = 7.3 Hz, 1H, *H*C=C), 2.41 (td, *J* = 7.3, 1.8 Hz, 2H, (C=O)CH_2CH$_2$), 2.03 (app-q, *J* = 7.3 Hz, 2H, CH_2(HC=C)), 1.73 – 1.64 (m, 5H, (C=O)CH$_2$CH_2, *H*C=C(CH_3)$_2$), 1.59 (s, 3H, HC=C(CH_3)$_2$) ppm;

^{13}C NMR (126 MHz, $CDCl_3$): δ_C 203.0 (H(*C*=O)), 133.0 (HC=*C*(CH$_3$)$_2$), 123.5 (H*C*=C(CH$_3$)$_2$), 43.5 ((C=O)*C*H$_2$CH$_2$), 27.4 (*C*H$_2$(HC=C)), 25.9 (HC=C(*C*H$_3$)$_2$), 22.4 ((C=O)CH$_2$*C*H$_2$), 17.9 (HC=C(*C*H$_3$)$_2$) ppm.

All characterisation data are consistent with that reported in the literature.[12]

4-Phenylbutanal

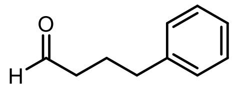

Synthesised according to **General Procedure A** from: 4-Phenylbutanoic acid (1.64 g, 10.0 mmol, 1.00 equiv), CDI (1.78 g, 1.10 equiv) and DIBAL-H (1.0 M in toluene, 21.0 mL, 2.10 equiv). Purified by flash column chromatography (SiO_2; 90:10 pentane:Et_2O) to afford 4-phenylbutanal (653 g, 4.41 mmol, 44%) as a colourless oil.

TLC: R_f = 0.35 (90:10 pentane:Et_2O).

NMR Spectroscopy (*see spectra*):

^{1}H NMR (400 MHz, $CDCl_3$): δ_H 9.76 (t, *J* = 1.6 Hz, 1H, *H*(C=O)), 7.33 – 7.27 (m, 2H, ArC*H*), 7.23 – 7.14 (m, 3H, ArC*H*), 2.67 (t, *J* = 7.5 Hz, 2H, (C=O)$CH_2CH_2CH_2$), 2.46 (td, *J* = 7.5, 1.6 Hz, 2H, (C=O)$CH_2CH_2CH_2$), 2.12 – 1.85 (m, 2H, (C=O)$CH_2CH_2CH_2$) ppm;

^{13}C NMR (126 MHz, $CDCl_3$): δ_C 202.5 (H(*C*=O)), 141.4 (Ar*C*), 128.6 (Ar*C*H), 126.3 (Ar*C*H), 43.3 ((C=O)$CH_2CH_2CH_2$), 35.2 ((C=O)$CH_2CH_2CH_2$), 23.8 ((C=O)$CH_2CH_2CH_2$) ppm.

All characterisation data are consistent with that reported in the literature.[13]

2.4. Synthesis of ABB-carbinols

1-(1-Azabicyclo[1.1.0]butan-3-yl)-3-phenylpropan-1-ol: 9a

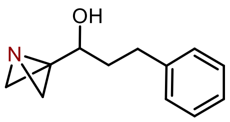

9a

ABB-Li was synthesised according to **General Procedure B** followed by the addition of hydrocinnamaldehyde (0.527 mL, 2.00 equiv) as a solution in anhydrous THF (2 mL). Yield of the corresponding ABB-carbinol was determined to be 77% by ^{1}H NMR analysis using dibromomethane as an internal standard.

1-(1-Azabicyclo[1.1.0]butan-3-yl)-3-(*o*-tolyl)propan-1-ol: 9f

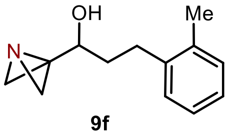

9f

ABB-Li was synthesised according to **General Procedure B** followed by the addition of **7f** (593 mg, 2.00 equiv) as a solution in anhydrous THF (2 mL). Yield of the corresponding ABB-carbinol was determined to be 70% by ^{1}H NMR analysis using dibromomethane as an internal standard.

1-(1-Azabicyclo[1.1.0]butan-3-yl)-3-(3,5-dimethoxyphenyl)propan-1-ol: 9g

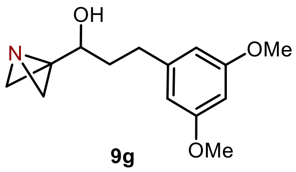

9g

ABB-Li was synthesised according to **General Procedure B** followed by the addition of **7g** (777 mg, 2.00 equiv)

as a solution in anhydrous THF (2 mL). Yield of the corresponding ABB-carbinol was determined to be 75% by ^{1}H NMR analysis using dibromomethane as an internal standard.

1-(1-Azabicyclo[1.1.0]butan-3-yl)-3-(3-fluorophenyl)propan-1-ol: 9h

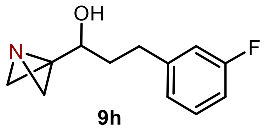

9h

ABB-Li was synthesised according to **General Procedure B** followed by the addition of **7h** (609 mg, 2.00 equiv) as a solution in anhydrous THF (2 mL). Yield of the corresponding ABB-carbinol was determined to be 80% by ^{1}H NMR analysis using dibromomethane as an internal standard.

2-(1-Azabicyclo[1.1.0]butan-3-yl)-4-phenylbutan-2-ol: 9i

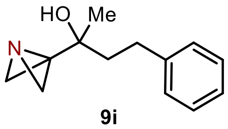

9i

ABB-Li was synthesised according to **General Procedure B** followed by the addition of 4-phenylbutan-2-one (0.599 mL, 2.00 equiv) as a solution in anhydrous THF (2 mL). Yield of the corresponding ABB-carbinol was determined to be 80% by ^{1}H NMR analysis using dibromomethane as an internal standard.

Benzylcyclohexyl(1-azabicyclo[1.1.0]butan-3-yl)methanol: 9j

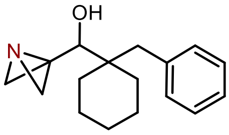

9j

Synthesised following a modified **General Procedure B**. ABB-Li was synthesised according to **General Procedure B** followed by the addition of **7j** (809 mg, 2.00 equiv) as a solution in anhydrous THF (2 mL). After 1 h at −78 °C the reaction was warmed to rt for 5 minutes before the reaction was quenched with H_2O. Yield of the corresponding ABB-carbinol was determined to be 85% by ^{1}H NMR analysis using dibromomethane as an internal standard.

1-(1-Azabicyclo[1.1.0]butan-3-yl)-2-phenoxypropan-1-ol: 9k

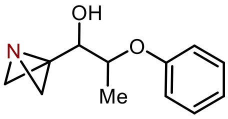

9k

ABB-Li was synthesised according to **General Procedure B** followed by the addition of **7k** (601 mg, 2.00 equiv) as a solution in anhydrous THF (2 mL). Yield of the corresponding ABB-carbinol was determined to be 58% by ^{1}H NMR analysis using dibromomethane as an internal standard (d.r. could not be accurately determined from the crude reaction mixture).

1-(1-Azabicyclo[1.1.0]butan-3-yl)-3-(naphthalen-2-yl)propan-1-ol: 9l

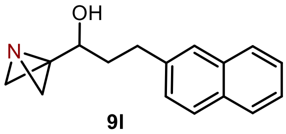

9l

ABB-Li was synthesised according to **General Procedure B** followed by the addition of **7l** (737 mg, 2.00 equiv) as a solution in anhydrous THF (2 mL). Yield of the corresponding ABB-carbinol was determined to be 75% by ^{1}H NMR analysis using dibromomethane as an internal standard.

2-(1-Azabicyclo[1.1.0]butan-3-yl)-4-(6-methoxynaphthalen-2-yl)butan-2-ol: 9m

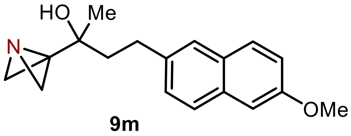

9m

ABB-Li was synthesised according to **General Procedure B** followed by the addition of nabumetone (913 mg, 2.00 equiv) as a solution in anhydrous THF (2 mL). Yield of the corresponding ABB-carbinol was determined to be 82% by ^{1}H NMR analysis using dibromomethane as an internal standard.

1-(1-Azabicyclo[1.1.0]butan-3-yl)-3-(5-methylfuran-2-yl)propan-1-ol: 9n

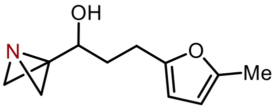

9n

ABB-Li was synthesised according to **General Procedure B** followed by the addition of 3-(5-methylfuran-2-yl)propanal (0.533 mL, 2.00 equiv) as a solution in anhydrous THF (2 mL). Yield of the corresponding ABB-carbinol was determined to be 74% by ^{1}H NMR analysis using dibromomethane as an internal standard.

3-(Benzo[*b*]thiophen-2-yl)-1-(1-azabicyclo[1.1.0]butan-3-yl)propan-1-ol: 9o

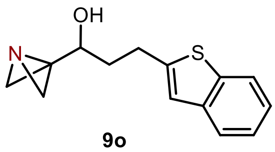

9o

ABB-Li was synthesised according to **General Procedure B** followed by the addition of **7o** (761 mg, 2.00 equiv) as a solution in anhydrous THF (2 mL). Yield of the corresponding ABB-carbinol was determined to be 71% by ^{1}H NMR analysis using dibromomethane as an internal standard.

1-(1-Azabicyclo[1.1.0]butan-3-yl)-6-methylhept-5-en-1-ol: 9p

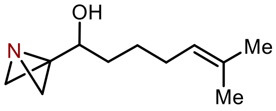

9p

ABB-Li was synthesised according to **General Procedure B** followed by the addition of **7p** (505 mg, 2.00 equiv) as a solution in anhydrous THF (2 mL). Yield of the corresponding ABB-carbinol was determined to be 80% by ^{1}H NMR analysis using dibromomethane as an internal standard.

1-(1-Azabicyclo[1.1.0]butan-3-yl)-2,6-dimethylhept-5-en-1-ol: 9q

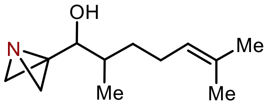

9q

ABB-Li was synthesised according to **General Procedure B** followed by the addition of 2,6-dimethylhept-5-enal (0.638 mL, 2.00 equiv) as a solution in anhydrous THF (2 mL). Yield of the desired product was determined to be 75% by ^{1}H NMR analysis using dibromomethane as an internal standard (d.r. could not be accurately determined from the crude reaction mixture).

1-(1-Azabicyclo[1.1.0]butan-3-yl)-3-phenylpropan-1-one

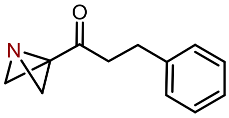

Synthesised following a modified **General Procedure B**. ABB-Li was synthesised according to **General Procedure B** followed by the addition of ethyl 3-phenylpropanoate (0.705 mL, 2.00 equiv) as a solution in anhydrous THF (2 mL). Purified by flash column chromatography (SiO_2; 80:20 hexane:EtOAc) to afford 1-(1-azabicyclo[1.1.0]butan-3-yl)-3-phenylpropan-1-one (101 mg, 0.539 mmol, 27%) as a white solid.

TLC: R_f = 0.31 (80:20 hexane:EtOAc).

NMR Spectroscopy (*see spectra*):

^{1}H NMR (500 MHz, $CDCl_3$): δ_H 7.31 – 7.27 (m, 2H, ArC*H*), 7.23 – 7.16 (m, 3H, ArC*H*), 2.97 – 2.91 (m, 4H, NC*H*$_2$, C*H*$_2$Ph), 2.88 – 2.85 (m, 2H, (C=O)C*H*$_2$), 1.55 (s, 2H, NC*H*$_2$) ppm;

^{13}C NMR (126 MHz, $CDCl_3$): δ_C 205.5 (*C*=O), 140.7 (Ar*C*), 128.7 (Ar*C*H), 128.5 (Ar*C*H), 126.4 (Ar*C*H), 55.5 (N*C*H$_2$), 41.0 ((C=O)*C*H$_2$), 30.0 (*C*N(CH$_2$)$_2$), 29.5 (*C*H$_2$Ph) ppm.

HRMS (ESI$^+$): m/z calc'd for $C_{12}H_{13}NNaO$ [M+Na]$^+$: 210.088935, found: 210.089689.

IR (film): ν_{max} 3062, 3028, 2948, 1698 (C=O), 1496, 1454, 1407, 1136 cm^{-1}.

3-(3-Phenylpropyl)-1-azabicyclo[1.1.0]butane: 2z

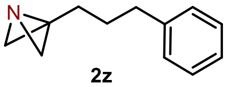

2z

Synthesised following a modified **General Procedure B**. ABB-Li was synthesised according to **General Procedure B** followed by the addition of (3-iodopropyl)benzene (0.418 mL, 1.30 equiv). The resulting solution was allowed to warm slowly to rt overnight. Purified by flash column chromatography (SiO_2; 80:20 pentane:Et_2O, 1% Et_3N) to afford **2z** (114 mg, 0.658 mmol, 33%) as a colourless oil.

TLC: R_f = 0.25 (80:20 pentane:Et_2O, 1% Et_3N).

NMR Spectroscopy (*see spectra*):

^{1}H NMR (500 MHz, $CDCl_3$): δ_H 7.31 – 7.27 (m, 2H, ArC*H*), 7.21 – 7.16 (m, 3H, ArC*H*), 2.70 (t, *J* = 7.7 Hz, 2H, C*H*$_2$Ph), 2.18 (s, 2H, NC*H*$_2$), 1.97 (t, *J* = 7.7 Hz, 2H, C*H*$_2$CH$_2$CH$_2$Ph), 1.87 – 1.72 (m, 2H, C*H*$_2$CH$_2$Ph), 1.09 (s, 2H, NC*H*$_2$) ppm;

^{13}C NMR (126 MHz, $CDCl_3$): δ_C 142.1 (Ar*C*), 128.6 (Ar*C*H), 128.5 (Ar*C*H), 126.0 (Ar*C*H), 53.8 (N*C*H$_2$), 35.7 (*C*H$_2$Ph), 30.3 (*C*N(CH$_2$)$_2$), 28.3 (CH$_2$*C*H$_2$CH$_2$Ph), 27.4 (*C*H$_2$CH$_2$CH$_2$Ph) ppm.

HRMS (ESI$^+$): m/z calc'd for $C_{12}H_{15}NNa$ [M+Na]$^+$: 196.109670, found: 196.110581.

IR (film): ν_{max} 3026, 2938, 1496, 1453, 904 cm^{-1}.

1-(1-Azabicyclo[1.1.0]butan-3-yl)-2-phenylethan-1-ol

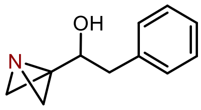

ABB-Li was synthesised according to **General Procedure B** followed by the addition of 2-phenylacetaldehyde

(0.445 mL, 2.00 equiv) as a solution in anhydrous THF (2 mL). Yield of the desired product was determined to be 68% by ^{1}H NMR analysis using dibromomethane as an internal standard.

1-(1-Azabicyclo[1.1.0]butan-3-yl)-4-phenylbutan-1-ol

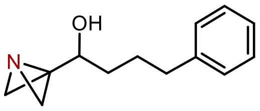

ABB-Li was synthesised according to **General Procedure B** followed by the addition of 4-phenylbutanal (0.593 mL, 2.00 equiv) as a solution in anhydrous THF (2 mL). Yield of the corresponding ABB-carbinol was determined to be 66% by ^{1}H NMR analysis using dibromomethane as an internal standard.

2.5. Synthesis of protected ABB-carbinols

3-(1-Methoxy-3-phenylpropyl)-1-azabicyclo[1.1.0]butane: 2a

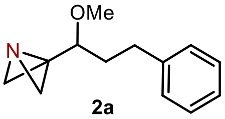

2a

Synthesised according to **General Procedure C** from: **9a** (189 mg, 1.00 mmol, 1.00 equiv), NaH (60% dispersion in mineral oil, 80.0 mg, 2.00 equiv) and MeI (0.187 mL, 3.00 equiv). Purified by flash column chromatography (SiO_2; 80:20 hexane:EtOAc) to afford **2a** (166 mg, 0.817 mmol, 82%) as a pale-yellow oil.

TLC: R_f = 0.26 (80:20 hexane:EtOAc).

NMR Spectroscopy (*see spectra*):

> **^{1}H NMR** (500 MHz, $CDCl_3$): δ_H 7.34 – 7.27 (m, 2H, ArC*H*), 7.24 – 7.21 (m, 3H, ArC*H*), 3.52 (dd, *J* = 8.4, 4.4 Hz, 1H, C*H*OCH_3), 3.49 (s, 3H, OCH_3), 2.88 (ddd, *J* = 14.1, 9.7, 5.4 Hz, 1H, CH_2Ph), 2.74 (ddd, *J* = 14.1, 9.5, 7.0 Hz, 1H, CH_2Ph), 2.39 (dd, *J* = 6.5, 2.8 Hz, 1H, NCH_2), 2.28 (dd, *J* = 6.5, 2.6 Hz, 1H, NCH_2), 2.03 – 1.81 (m, 2H, CH_2CH_2Ph), 1.23 (dd, *J* = 2.8, 0.7 Hz, 1H, NCH_2), 1.17 (dd, *J* = 2.6, 0.7 Hz, 1H, NCH_2) ppm;
>
> **^{13}C NMR** (126 MHz, $CDCl_3$): δ_C 141.9 (Ar*C*), 128.6 (Ar*C*H), 128.6 (Ar*C*H), 126.0 (Ar*C*H), 77.6 (*C*HOCH_3), 57.8 (O*C*H_3), 53.0 (N*C*H_2), 51.9 (N*C*H_2), 34.9 (*C*H_2CH_2Ph), 31.7 (*C*H_2Ph), 30.7 (*C*N$(CH_2)_2$) ppm.

HRMS (ESI$^+$): m/z calc'd for $C_{13}H_{17}NNaO$ $[M+Na]^+$: 226.120235, found: 226.121221.

IR (film): ν_{max} 3026, 2942, 2834, 1496, 1454, 1109 cm^{-1}.

3-(1-(Benzyloxy)-3-phenylpropyl)-1-azabicyclo[1.1.0]butane: 2b

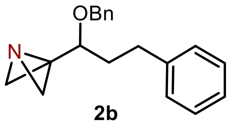

2b

Synthesised according to **General Procedure C** from: **9a** (189 mg, 1.00 mmol, 1.00 equiv), NaH (60% dispersion in mineral oil, 80.0 mg, 2.00 equiv) and BnBr (0.356 mL, 3.00 equiv). Purified by flash column chromatography (SiO_2; 85:15 hexane:EtOAc) to afford **2b** (209 mg, 0.748 mmol, 75%) as a colourless oil.

TLC: R_f = 0.22 (85:15 hexane:EtOAc).

NMR Spectroscopy (see spectra):

^{1}**H NMR** (400 MHz, $CDCl_3$): δ_H 7.40 – 7.33 (m, 4H, ArC*H*), 7.32 – 7.28 (m, 2H, ArC*H*), 7.26 – 7.24 (m, 1H, ArC*H*), 7.21 – 7.13 (m, 3H, ArC*H*), 4.81 (d, *J* = 11.6 Hz, 1H, OC*H*$_2$Ph), 4.55 (d, *J* = 11.6 Hz, 1H, OC*H*$_2$Ph), 3.73 (dd, *J* = 8.7, 4.1 Hz, 1H, C*H*OCH$_2$Ph), 2.89 (ddd, *J* = 14.2, 9.8, 5.3 Hz, 1H, CH$_2$C*H*$_2$Ph), 2.71 (ddd, *J* = 14.2, 9.5, 6.9 Hz, 1H, CH$_2$C*H*$_2$Ph), 2.39 (dd, *J* = 6.5, 2.8 Hz, 1H, NC*H*$_2$), 2.28 (dd, *J* = 6.5, 2.5 Hz, 1H, NC*H*$_2$), 2.03 (dddd, *J* = 14.0, 9.5, 8.7, 5.3 Hz, 1H, C*H*$_2$CH$_2$Ph), 1.91 (dddd, *J* = 14.0, 9.8, 6.9, 4.1 Hz, 1H, C*H*$_2$CH$_2$Ph), 1.21 (d, *J* = 2.8 Hz, 1H, NC*H*$_2$), 1.16 (d, *J* = 2.5 Hz, 1H, NC*H*$_2$) ppm;

^{13}**C NMR** (126 MHz, $CDCl_3$): δ_C 141.9 (Ar*C*), 138.6 (Ar*C*), 128.6 (Ar*C*H), 128.6 (Ar*C*H), 128.5 (Ar*C*H), 128.0 (Ar*C*H), 127.8 (Ar*C*H), 126.0 (Ar*C*H), 75.5 (*C*HOCH$_2$Ph), 71.7 (O*C*H$_2$Ph), 52.9 (N*C*H$_2$), 52.0 (N*C*H$_2$), 35.2 (*C*H$_2$CH$_2$Ph), 31.9 (*C*H$_2$Ph), 31.1 (*C*N(CH$_2$)$_2$) ppm.

HRMS (ESI$^+$): m/z calc'd for $C_{19}H_{21}NNaO$ $[M+Na]^+$: 302.151535 found: 302.152201.

IR (film): ν_{max} 3027, 2944, 2862, 1496, 1454, 1100 cm^{-1}.

3-(1-(Allyloxy)-3-phenylpropyl)-1-azabicyclo[1.1.0]butane: 2c

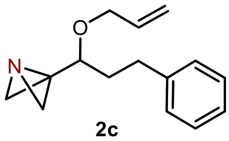

2c

Synthesised according to **General Procedure C** from: **9a** (132 mg, 0.700 mmol, 1.00 equiv), NaH (60% dispersion in mineral oil, 56.0 mg, 2.00 equiv) and allyl bromide (0.182 mL, 3.00 equiv). Purified by flash column chromatography (SiO_2; 90:10 hexane:EtOAc) to afford **2c** (127 mg, 0.554 mmol, 79%) as a colourless oil.

TLC: R_f = 0.21 (90:10 hexane:EtOAc).

NMR Spectroscopy (see spectra):

^{1}**H NMR** (400 MHz, $CDCl_3$): δ_H 7.33 – 7.24 (m, 2H, ArC*H*), 7.22 – 7.16 (m, 3H, ArC*H*), 5.94 (app-ddt, *J* = 17.3, 10.4, 5.6 Hz, 1H, *H*C=CH$_2$), 5.30 (app-dq, *J* = 17.3, 1.6 Hz, 1H, HC=C*H*$_2$), 5.18 (app-dq, *J* = 10.4, 1.6 Hz, 1H, HC=C*H*$_2$), 4.26 (app-ddt, *J* = 12.7, 5.6, 1.6 Hz, 1H, OC*H*$_2$(C=C)), 4.02 (app-ddt, *J* = 12.7, 5.6, 1.6 Hz, 1H, OC*H*$_2$(C=C)), 3.66 (dd, *J* = 8.6, 4.3 Hz, 1H, C*H*O), 2.88 (ddd, *J* = 14.0, 9.8, 5.5 Hz, 1H,

CH_2CH_2Ph), 2.73 (ddd, *J* = 14.0, 9.5, 6.9 Hz, 1H, CH_2CH_2Ph), 2.36 (dd, *J* = 6.5, 2.8 Hz, 1H, NCH_2), 2.26 (dd, *J* = 6.5, 2.6 Hz, 1H, NCH_2), 2.09 – 1.85 (m, 2H, CH_2CH_2Ph), 1.19 (dd, *J* = 2.8, 0.7 Hz, 1H, NCH_2), 1.15 (dd, *J* = 2.6, 0.7 Hz, 1H, NCH_2) ppm;

^{13}C NMR (126 MHz, $CDCl_3$): δ_C 141.9 (ArC), 135.0 (HC=CH_2), 128.6 (ArCH), 128.5 (ArCH), 126.0 (ArCH), 117.1 (HC=CH_2), 75.6 (CHO), 70.8 (OCH_2(HC=C)), 53.0 (NCH_2), 52.0 (NCH_2), 35.1 (CH_2CH_2Ph), 31.8 (CH_2CH_2Ph), 31.0 (CN$(CH_2)_2$) ppm.

HRMS (ESI$^+$): m/z calc'd for $C_{15}H_{19}NNaO$ [M+Na]$^+$: 252.135885 found: 252.136272.

IR (film): ν_{max} 3026, 2942, 2861, 1496, 1455, 1123, 1098 cm^{-1}.

3-(3-Phenyl-1-((triethylsilyl)oxy)propyl)-1-azabicyclo[1.1.0]butane: 2d

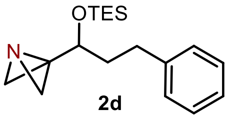

2d

Synthesised according to **General Procedure D** from: **9a** (151 mg, 0.800 mmol, 1.00 equiv), ^{n}BuLi (1.6 M in hexane, 0.900 mL, 1.80 equiv) and TESCl (0.282 mL, 2.00 equiv). Purified by flash column chromatography (SiO_2; 95:5 hexane:EtOAc) to afford **2d** (175 mg, 0.577 mmol, 72%) as a colourless oil.

TLC: R_f = 0.21 (95:5 hexane:EtOAc).

NMR Spectroscopy (*see spectra*):

^{1}H NMR (400 MHz, $CDCl_3$): δ_H 7.33 – 7.24 (m, 2H, ArCH), 7.22 – 7.16 (m, 3H, ArCH), 4.02 (dd, *J* = 6.8, 5.4 Hz, 1H, CHOSi), 2.88 – 2.80 (m, 1H, CH_2Ph), 2.70 (ddd, *J* = 13.7, 9.6, 6.8 Hz, 1H, CH_2Ph), 2.35 (dd, *J* = 6.5, 2.8 Hz, 1H, NCH_2), 2.31 (dd, *J* = 6.5, 2.5 Hz, 1H, NCH_2), 1.97 – 1.87 (m, 2H, CH_2CH_2Ph), 1.18 (d, *J* = 2.5 Hz, 1H, NCH_2), 1.14 (d, *J* = 2.8 Hz, 1H, NCH_2), 0.98 (t, *J* = 7.9 Hz, 9H, Si$(CH_2CH_3)_3$)), 0.63 (q, *J* = 7.9 Hz, 6H, Si$(CH_2CH_3)_3$) ppm;

^{13}C NMR (126 MHz, $CDCl_3$): δ_C 142.2 (ArC), 128.5 (ArCH), 128.5 (ArCH), 126.0 (ArCH), 70.0 (CHOSi), 52.9 (NCH_2), 52.7 (NCH_2), 37.8 (CH_2CH_2Ph), 32.7 (CN$(CH_2)_2$), 31.9 (CH_2Ph), 7.0 (Si$(CH_2CH_3)_3$), 5.2 (Si$(CH_2CH_3)_3$) ppm.

HRMS (ESI$^+$): m/z calc'd for $C_{18}H_{29}NNaOSi$ [M+Na]$^+$: 326.1911, found: 326.1923.

IR (film): ν_{max} 3027, 2952, 2876, 1455, 1239, 1103 cm^{-1}.

3-(3-Phenyl-1-((trimethylsilyl)oxy)propyl)-1-azabicyclo[1.1.0]butane: 2e

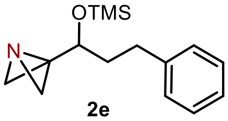

2e

Synthesised according to **General Procedure D** from: **9a** (94.6 mg, 0.500 mmol, 1.00 equiv), ^{n}BuLi (1.6 M in hexane, 0.563 mL, 1.80 equiv) and TMSCl (0.127 mL, 2.00 equiv). Purified by flash column chromatography (SiO_2; 92:8 hexane:EtOAc) to afford **2e** (78.8 mg, 0.301 mmol, 60%) as a colourless oil.

TLC: R_f = 0.23 (92:8 hexane:EtOAc).

NMR Spectroscopy (*see spectra*):

^{1}H NMR (400 MHz, $CDCl_3$): δ_H 7.30 – 7.26 (m, 2H, ArC*H*), 7.23 – 7.13 (m, 3H, ArC*H*), 3.99 (app-t, *J* = 6.3 Hz, 1H, C*H*OSi), 2.88 – 2.77 (m, 1H, CH_2Ph), 2.64 (app-dt, *J* = 13.7, 8.1 Hz, 1H, CH_2Ph), 2.32 (dd, *J* = 6.5, 2.6 Hz, 1H, NCH_2), 2.29 (dd, *J* = 6.5, 2.4 Hz, 1H, NCH_2), 1.93 – 1.87 (m, 2H, CH_2CH_2Ph), 1.17 (d, *J* = 2.4 Hz, 1H, NCH_2), 1.13 (d, *J* = 2.6 Hz, 1H, NCH_2), 0.14 (s, 9H, Si(CH_3)$_3$) ppm;

^{13}C NMR (126 MHz, $CDCl_3$): δ_C 142.0 (Ar*C*), 128.6 (Ar*C*H), 128.5 (Ar*C*H), 126.0 (Ar*C*H), 70.0 (*C*HOSi), 52.7 (N*C*H_2), 52.6 (N*C*H_2), 37.4 (*C*H_2CH_2Ph), 32.7 (*C*N(CH_2)$_2$), 32.0 (*C*H_2Ph), 0.5 (Si(*C*H_3)$_3$) ppm.

HRMS (ESI$^+$): m/z calc'd for $C_{15}H_{23}NNaOSi$ $[M+Na]^+$: 284.1441, found: 284.1431.

IR (film): ν_{max} 3027, 2952, 2861, 1250, 1104 cm^{-1}.

3-(1-(Benzyloxy)-3-(*o*-tolyl)propyl)-1-azabicyclo[1.1.0]butane: 2f

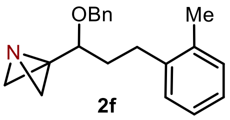

2f

Synthesised according to **General Procedure C** from: **9f** (285 mg, 1.40 mmol, 1.00 equiv), NaH (60% dispersion in mineral oil, 112 mg, 2.00 equiv) and BnBr (0.499 mL, 3.00 equiv). Purified by flash column chromatography (SiO_2; 86.5:13.5 hexane:EtOAc) to afford **2f** (399 mg, 1.36 mmol, 97%) as a colourless oil.

TLC: R_f = 0.21 (86.5:13.5 hexane:EtOAc).

NMR Spectroscopy (*see spectra*):

^{1}H NMR (400 MHz, $CDCl_3$): δ_H 7.41 – 7.28 (m, 5H, ArC*H*), 7.17 – 7.07 (m, 4H, ArC*H*), 4.83 (d, *J* = 11.7 Hz, 1H, OCH_2Ph), 4.59 (d, *J* = 11.7 Hz, 1H, OCH_2Ph), 3.78 (dd, *J* = 8.5, 4.2 Hz, 1H, C*H*OCH_2Ph), 2.89 (ddd, *J* = 14.0, 10.4, 5.2 Hz, 1H, CH_2CH_2Ph), 2.67 (ddd, *J* = 14.0, 10.2, 6.5 Hz, 1H, CH_2CH_2Ph), 2.40 (dd, *J* = 6.5, 2.8 Hz, 1H, NCH_2), 2.33 – 2.25 (m, 4H, ArCH_3, NCH_2), 2.02 – 1.81 (m, 2H, CH_2CH_2Ph), 1.22 (dd, *J* = 2.8, 0.7 Hz, 1H, NCH_2), 1.17 (dd, *J* = 2.6, 0.7 Hz, 1H, NCH_2) ppm;

^{13}C NMR (126 MHz, $CDCl_3$): δ_C 140.1 (Ar*C*), 138.6 (Ar*C*), 136.1 (Ar*C*), 130.4 (Ar*C*H), 129.0 (Ar*C*H), 128.5 (Ar*C*H), 127.9 (Ar*C*H), 127.8 (Ar*C*H), 126.2 (Ar*C*H), 126.1 (Ar*C*H), 75.8 (*C*HOCH_2Ph), 71.7 (O*C*H_2Ph), 53.0 (N*C*H_2), 52.0 (N*C*H_2), 33.9 (*C*H_2CH_2Ph), 31.0 (*C*N(CH_2)$_2$), 29.3 (*C*H_2Ph), 19.4 (Ar*C*H_3) ppm.

HRMS (ESI$^+$): m/z calc'd for $C_{20}H_{23}NNaO$ $[M+Na]^+$: 316.167185 found: 316.167435.

IR (film): ν_{max} 3029, 2944, 2869, 1494, 1455, 1096 cm^{-1}.

3-(1-(Benzyloxy)-3-(3,5-dimethoxyphenyl)propyl)-1-azabicyclo[1.1.0]butane: 2g

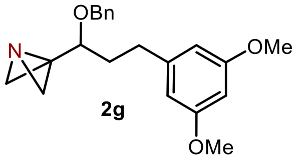

2g

Synthesised according to **General Procedure C** from: **9g** (280 mg, 1.12 mmol, 1.00 equiv), NaH (60% dispersion in mineral oil, 90.0 mg, 2.00 equiv) and BnBr (0.401 mL, 3.00 equiv). Purified by flash column chromatography (SiO_2; 75:25 hexane:EtOAc) to afford **2g** (340 mg, 1.00 mmol, 89%) as a colourless oil.

TLC: R_f = 0.24 (75:25 hexane:EtOAc).

NMR Spectroscopy (see spectra):

^{1}H NMR (400 MHz, $CDCl_3$): δ_H 7.41 – 7.27 (m, 5H, ArC*H*), 6.34 (d, *J* = 2.3 Hz, 2H, ArC*H*), 6.30 (t, *J* = 2.3 Hz, 1H, ArC*H*), 4.82 (d, *J* = 11.6 Hz, 1H, OC*H*$_2$Ph), 4.56 (d, *J* = 11.6 Hz, 1H, OC*H*$_2$Ph), 3.77 – 3.72 (m, 7H, ArOC*H*$_3$, C*H*OCH$_2$Ph), 2.84 (ddd, *J* = 13.8, 9.9, 5.3 Hz, 1H, CH$_2$C*H*$_2$Ph), 2.65 (ddd, *J* = 13.8, 9.7, 6.7 Hz, 1H, CH$_2$C*H*$_2$Ph), 2.39 (dd, *J* = 6.5, 2.8 Hz, 1H, NC*H*$_2$), 2.29 (dd, *J* = 6.5, 2.5 Hz, 1H, NC*H*$_2$), 2.08 – 1.83 (m, 2H, C*H*$_2$CH$_2$Ph), 1.21 (dd, *J* = 2.8, 0.7 Hz, 1H, NC*H*$_2$), 1.17 (dd, *J* = 2.5, 0.7 Hz, 1H, NC*H*$_2$) ppm;

^{13}C NMR (126 MHz, $CDCl_3$): δ_C 160.9 (Ar*C*OCH$_3$), 144.3 (Ar*C*), 138.6 (Ar*C*), 128.6 (Ar*C*H), 127.9 (Ar*C*H), 127.8 (Ar*C*H), 106.6 (Ar*C*H), 98.1 (Ar*C*H), 75.6 (*C*HOCH$_2$Ph), 71.8 (O*C*H$_2$Ph), 55.4 (ArCO*C*H$_3$), 52.9 (N*C*H$_2$), 52.0 (N*C*H$_2$), 35.1 (*C*H$_2$CH$_2$Ph), 32.3 (*C*H$_2$Ph), 31.0(*C*N(CH$_2$)$_2$) ppm.

HRMS (ESI$^+$): m/z calc'd for $C_{21}H_{25}NNaO_3$ $[M+Na]^+$: 362.1727 found: 362.1730.

IR (film): ν_{max} 2941, 2838, 1596, 1556, 1429, 1205, 1151, 1058 cm^{-1}.

3-(1-(Benzyloxy)-3-(3-fluorophenyl)propyl)-1-azabicyclo[1.1.0]butane: 2h

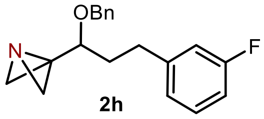

2h

Synthesised according to **General Procedure C** from: **9h** (332 mg, 1.60 mmol, 1.00 equiv), NaH (60% dispersion in mineral oil, 128 mg, 2.00 equiv) and BnBr (0.570 mL, 3.00 equiv). Purified by flash column chromatography (SiO_2; 85:15 hexane:EtOAc) to afford **2h** (373 mg, 1.25 mmol, 78%) as a colourless oil.

TLC: R_f = 0.22 (85:15 hexane:EtOAc).

NMR Spectroscopy (see spectra):

^{1}H NMR (500 MHz, $CDCl_3$): δ_H 7.42 – 7.30 (m, 5H, ArC*H*), 7.27 – 7.20 (m, 1H, ArC*H*), 6.97 – 6.84 (m, 3H, ArC*H*), 4.83 (d, *J* = 11.7 Hz, 1H, OC*H*$_2$Ph), 4.57 (d, *J* = 11.7 Hz, 1H, OC*H*$_2$Ph), 3.74 (dd, *J* = 8.8, 4.1 Hz, 1H, OC*H*$_2$Ph), 2.90 (ddd, *J* = 14.2, 9.7, 5.3 Hz, 1H, CH$_2$C*H*$_2$Ph), 2.72 (ddd, *J* = 14.2, 9.5, 6.9 Hz, 1H, CH$_2$C*H*$_2$Ph), 2.41 (dd, *J* = 6.5, 2.7 Hz, 1H, NC*H*$_2$), 2.31 (dd, *J* = 6.5, 2.5 Hz, 1H, NC*H*$_2$), 2.11 – 1.98 (m, 1H, C*H*$_2$CH$_2$Ph), 1.98 – 1.85 (m, 1H, C*H*$_2$CH$_2$Ph), 1.24 (d, *J* = 2.7 Hz, 1H, NC*H*$_2$), 1.19 (d, *J* = 2.5 Hz, 1H,

NC*H*$_2$) ppm;

^{13}C NMR (126 MHz, $CDCl_3$): δ_C 163.1 (d, *J* = 245.3 Hz, Ar*C*F), 144.5 (d, *J* = 7.2 Hz, Ar*C*), 138.4 (Ar*C*), 129.9 (d, *J* = 8.3 Hz, Ar*C*H), 128.6 (Ar*C*H), 128.0 (Ar*C*H), 127.9 (Ar*C*H), 124.2 (d, *J* = 2.8 Hz, Ar*C*H), 115.4 (d, *J* = 20.8 Hz, Ar*C*H), 112.9 (d, *J* = 21.1 Hz, Ar*C*H), 75.3 (*C*HOCH$_2$Ph), 71.7 (O*C*H$_2$Ph), 53.0 (N*C*H$_2$), 51.9 (N*C*H$_2$), 34.9 (*C*H$_2$CH$_2$Ph), 31.6 (*C*H$_2$Ph), 30.9 (*C*N(CH$_2$)$_2$) ppm;

^{19}F NMR (376 MHz, $CDCl_3$): δ_F –113.6 ppm.

HRMS (ESI$^+$): m/z calc'd for $C_{19}H_{20}FNNaO$ [M+Na]$^+$: 320.142113 found: 320.142613.

IR (film): ν_{max} 3032, 2944, 2864, 1588, 1488, 1453, 1249, 1100 cm^{-1}.

3-(2-(Benzyloxy)-4-phenylbutan-2-yl)-1-azabicyclo[1.1.0]butane: 2i

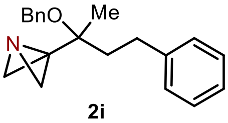

2i

Synthesised according to **General Procedure C** from: **9i** (337 mg, 1.66 mmol, 1.00 equiv), NaH (60% dispersion in mineral oil, 133 mg, 2.00 equiv) and BnBr (0.592 mL, 3.00 equiv). Purified by flash column chromatography (SiO_2; 86:14 hexane:EtOAc) to afford **2i** (358 mg, 1.22 mmol, 74%) as a pale yellow oil.

TLC: R_f = 0.24 (86:14 hexane:EtOAc).

NMR Spectroscopy (*see spectra*):

^{1}H NMR (400 MHz, $CDCl_3$): δ_H 7.38 – 7.26 (m, 7H, ArC*H*), 7.18 (m, 3H, ArC*H*), 4.66 (s, 2H, OC*H*$_2$Ph), 2.90 – 2.71 (m, 2H, CH$_2$C*H*$_2$Ph), 2.44 (dd, *J* = 6.7, 2.8 Hz, 1H, NC*H*$_2$), 2.31 (dd, *J* = 6.7, 2.6 Hz, 1H, NC*H*$_2$), 2.09 (ddd, *J* = 13.9, 11.5, 5.5 Hz, 1H, C*H*$_2$CH$_2$Ph), 1.94 (ddd, *J* = 13.9, 11.7, 5.8 Hz, 1H, C*H*$_2$CH$_2$Ph), 1.38 (s, 3H, C(C*H*$_3$)OCH$_2$Ph) 1.10 (dd, *J* = 2.8, 0.6 Hz, 1H, NC*H*$_2$), 1.08 (dd, *J* = 2.6, 0.6 Hz, 1H, NC*H*$_2$) ppm;

^{13}C NMR (126 MHz, $CDCl_3$): δ_C 142.5 (Ar*C*), 139.5 (Ar*C*), 128.6 (Ar*C*H), 128.5 (Ar*C*H), 128.5 (Ar*C*H), 127.4 (Ar*C*H), 127.3 (Ar*C*H), 126.0 (Ar*C*H), 74.3 (*C*(CH$_3$)OCH$_2$Ph), 64.9 (O*C*H$_2$Ph), 51.8 (N*C*H$_2$), 51.3 (N*C*H$_2$), 40.2 (*C*H$_2$CH$_2$Ph), 34.4 (*C*N(CH$_2$)$_2$), 30.0 (*C*H$_2$Ph), 20.9 (C(*C*H$_3$)) ppm.

HRMS (ESI$^+$): m/z calc'd for $C_{20}H_{23}NNaO$ [M+Na]$^+$: 316.1672 found: 316.1676.

IR (film): ν_{max} 3027, 2943, 2862, 1496, 1454, 1105, 1066 cm^{-1}.

3-((1-Benzylcyclohexyl)(benzyloxy)methyl)-1-azabicyclo[1.1.0]butane: 2j

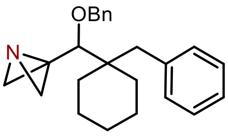

2j

Synthesised according to **General Procedure C** from: **9j** (438 mg, 1.70 mmol, 1.00 equiv), NaH (60% dispersion in mineral oil, 136 mg, 2.00 equiv) and BnBr (0.606 mL, 3.00 equiv). Purified by flash column chromatography (SiO_2; 98:2 toluene:Et_2O) to afford **2j** (498 mg, 1.43 mmol, 84%) as a colourless oil.

TLC: R_f = 0.22 (98:2 toluene:Et_2O).

NMR Spectroscopy (*see spectra*):

^{1}H NMR (400 MHz, $CDCl_3$): δ_H 7.39 – 7.32 (m, 4H, ArC*H*), 7.30 – 7.16 (m, 1H, ArC*H*), 7.16 – 7.09 (m, 5H, ArC*H*), 4.91 (d, *J* = 11.8 Hz, 1H, OC*H*$_2$Ph), 4.44 (d, *J* = 11.8 Hz, 1H, OC*H*$_2$Ph), 3.75 (s, 1H, C*H*OCH$_2$Ph), 2.97 (d, *J* = 13.4 Hz, 1H, (C)C*H*$_2$Ph), 2.89 (d, *J* = 13.4 Hz, 1H, (C)C*H*$_2$Ph), 2.52 (dd, *J* = 6.5, 2.8 Hz, 1H, NC*H*$_2$), 2.18 (dd, *J* = 6.5, 2.6 Hz, 1H, NC*H*$_2$), 1.81 – 1.70 (m, 2H, cy-C*H*$_2$), 1.54 – 1.25 (m, 8H, cy-C*H*$_2$), 1.22 (d, *J* = 2.8 Hz, 1H, NC*H*$_2$), 1.16 (d, *J* = 2.6 Hz, 1H, NC*H*$_2$) ppm;

^{13}C NMR (126 MHz, $CDCl_3$): δ_C 139.3 (Ar*C*), 139.1 (Ar*C*), 131.3 (Ar*C*H), 128.4 (Ar*C*H), 127.8 (Ar*C*H), 127.5 (Ar*C*H), 127.4 (Ar*C*H), 125.9 (Ar*C*H), 77.6 (*C*HOCH$_2$Ph), 72.7 (O*C*H$_2$Ph), 52.8 (N*C*H$_2$), 52.1 (N*C*H$_2$), 43.1 ((*C*)CH$_2$Ph), 40.0 (*C*H$_2$Ph), 31.8 (cy-*C*H$_2$), 30.6 (cy-*C*H$_2$), 29.8 (*C*N(CH$_2$)$_2$), 26.2 (cy-*C*H$_2$), 21.8 (cy-*C*H$_2$), 21.7 (cy-*C*H$_2$) ppm.

HRMS (ESI$^+$): m/z calc'd for $C_{24}H_{29}NNaO$ $[M+Na]^+$: 370.214135 found: 370.214791.

IR (film): ν_{max} 3028, 2932, 2861, 1496, 1453, 1070 cm^{-1}.

3-(1-(Benzyloxy)-2-phenoxypropyl)-1-azabicyclo[1.1.0]butane: 2k

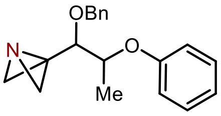

2k

Synthesised according to **General Procedure C** from: **9k** (238 mg, 1.16 mmol, 1.00 equiv), NaH (60% dispersion in mineral oil, 92.8 mg, 2.00 equiv) and BnBr (0.413 mL, 3.00 equiv). Purified by flash column chromatography (SiO_2; 95:5 toluene:Et_2O) to afford **2k** as a 1:1 mixture of diastereomers (341 mg, 1.15 mmol, 99%) as a colourless oil.

TLC: R_f = 0.24 (95:5 toluene:Et_2O).

NMR Spectroscopy (*see spectra*):

^{1}H NMR (500 MHz, $CDCl_3$) *Distinct diastereomer signals are labelled as d^1 and d^2*: δ_H 7.39 – 7.27 (m, 6H, ArC*H*), 7.25 – 7.20 (m, 1H, ArC*H*), 6.97 – 6.80 (m, 3H, ArC*H*), 4.81 (d, *J* = 11.9 Hz, 0.5H, *d^1*-OC*H*$_2$Ph), 4.81 (d, *J* = 11.7 Hz, 0.5H, *d^2*-OC*H*$_2$Ph), 4.70 – 4.55 (m, 2H, OC*H*$_2$Ph, C*H*(CH$_3$)OPh), 3.89 (d, *J* = 5.1 Hz, 0.5H, *d^1*-C*H*OCH$_2$Ph), 3.83 (d, *J* = 5.1 Hz, 0.5H, *d^2*-C*H*OCH$_2$Ph), 2.55 (dd, *J* = 6.5, 2.9 Hz, 0.5H, *d^1*-NC*H*$_2$), 2.49 (dd, *J* = 6.5, 2.8 Hz, 0.5H, *d^2*-NC*H*$_2$), 2.30 (dd, *J* = 6.5, 2.6 Hz, 0.5H, *d^2*-NC*H*$_2$), 2.23 (dd, *J* = 6.5, 2.5 Hz, 0.5H, *d^1*-NC*H*$_2$), 1.45 (d, *J* = 9.1 Hz, 1.5H, *d^1*-CHC*H*$_3$), 1.44 (d, *J* = 9.1 Hz, 1.5H, *d^2*-CHC*H*$_3$), 1.26 (dd, *J* = 2.6, 0.7 Hz, 0.5H, *d^2*-NC*H*$_2$), 1.20 – 1.17 (m, 1H, NC*H*$_2$), 1.13 (d, *J* = 2.9 Hz, 0.5H, *d^1*-NC*H*$_2$) ppm;

^{13}C NMR (126 MHz, $CDCl_3$): δ_C 157.9 and 157.8 (Ar*C*O), 138.3 and 138.3 (Ar*C*), 129.7 and 129.7 (Ar*C*H),

128.5 (Ar*C*H), 128.2 and 128.0 (Ar*C*H), 127.8 and 127.8 (Ar*C*H), 121.2 and 121.1 (Ar*C*H), 116.1 and 116.0 (Ar*C*H), 77.9 and 77.3 (*C*HOCH$_2$Ph), 75.8 and 75.5 (*C*H(CH$_3$)OPh), 73.1 and 72.9 (O*C*H$_2$Ph), 53.0 and 51.9 (N*C*H$_2$), 52.7 and 51.6 (N*C*H$_2$), 30.3 and 29.5 (*C*N(CH$_2$)$_2$), 16.2 (CH(*C*H$_3$)OPh) ppm.

HRMS (ESI$^+$): m/z calc'd for $C_{19}H_{21}NNaO_2$ [M+Na]$^+$: 318.146450 found: 318.145851.

IR (film): ν_{max} 3031, 2981, 2940, 2878, 1560, 1494, 1241, 1092 cm^{-1}.

3-(1-(Benzyloxy)-3-(naphthalen-2-yl)propyl)-1-azabicyclo[1.1.0]butane: 2l

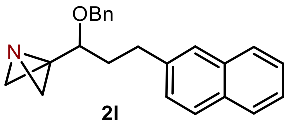

2l

Synthesised according to **General Procedure C** from: **9l** (179 mg, 0.750 mmol, 1.00 equiv), NaH (60% dispersion in mineral oil, 60.0 mg, 2.00 equiv) and BnBr (0.267 mL, 3.00 equiv). Purified by flash column chromatography (SiO$_2$; 85:15 hexane:EtOAc) to afford **2l** (164 mg, 0.499 mmol, 67%) as a colourless oil.

TLC: R_f = 0.29 (85:15 hexane:EtOAc).

NMR Spectroscopy (*see spectra*):

^{1}**H NMR** (500 MHz, CDCl$_3$): δ_H 7.85 – 7.70 (m, 3H, ArC*H*), 7.57 (s, 1H, ArC*H*), 7.48 – 7.29 (m, 8H, ArC*H*), 4.82 (d, *J* = 11.7 Hz, 1H, OC*H*$_2$Ph), 4.56 (d, *J* = 11.7 Hz, 1H, OC*H*$_2$Ph), 3.76 (dd, *J* = 8.8, 4.1 Hz, 1H, C*H*OCH$_2$Ph), 3.05 (ddd, *J* = 14.1, 9.4, 5.3 Hz, 1H, CH$_2$C*H*$_2$Ar), 2.88 (ddd, *J* = 14.1, 9.3, 7.0 Hz, 1H, CH$_2$C*H*$_2$Ar), 2.39 (dd, *J* = 6.5, 2.8 Hz, 1H, NC*H*$_2$), 2.29 (dd, *J* = 6.5, 2.6 Hz, 1H, NC*H*$_2$), 2.18 – 1.96 (m, 2H, C*H*$_2$CH$_2$Ar), 1.21 (d, *J* = 2.8 Hz, 1H, NC*H*$_2$), 1.17 (d, *J* = 2.6 Hz, 1H, NC*H*$_2$) ppm;

^{13}**C NMR** (126 MHz, CDCl$_3$): δ_C 139.4 (Ar*C*), 138.6 (Ar*C*), 133.8 (Ar*C*), 132.2 (Ar*C*), 128.6 (Ar*C*H), 128.1 (Ar*C*H), 128.0 (Ar*C*H), 127.8 (Ar*C*H), 127.7 (Ar*C*H), 127.6 (Ar*C*H), 127.5 (Ar*C*H), 126.7 (Ar*C*H), 126.1 (Ar*C*H), 125.3 (Ar*C*H), 75.4 (*C*HOCH$_2$Ph), 71.8 (O*C*H$_2$Ph), 53.0 (N*C*H$_2$), 52.0 (N*C*H$_2$), 35.1 (*C*H$_2$CH$_2$Ar), 32.0 (*C*H$_2$Ar), 31.1 (*C*N(CH$_2$)$_2$) ppm.

HRMS (ESI$^+$): m/z calc'd for $C_{23}H_{23}NNaO$ [M+Na]$^+$: 352.167185 found: 352.168407.

IR (film): ν_{max} 3052, 2944, 281, 1508, 1454, 1098, 1066 cm^{-1}.

3-(2-(Benzyloxy)-4-(6-methoxynaphthalen-2-yl)butan-2-yl)-1-azabicyclo[1.1.0]butane: 2m

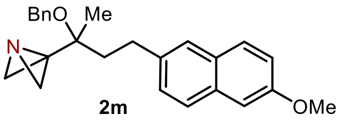

2m

Synthesised according to **General Procedure C** from: **9m** (465 mg, 1.64 mmol, 1.00 equiv), NaH (60% dispersion in mineral oil, 131 mg, 2.00 equiv) and BnBr (0.584 mL, 3.00 equiv). Purified by flash column

chromatography (SiO_2; 82.5:17.5 hexane:EtOAc) to afford **2m** (421 mg, 1.13 mmol, 69%) as a pale yellow oil.

TLC: R_f = 0.23 (82.5:17.5 hexane:EtOAc).

NMR Spectroscopy (*see spectra*):

^{1}H NMR (500 MHz, $CDCl_3$): δ_H 7.67 (dd, *J* = 8.6, 2.8 Hz, 2H, ArC*H*), 7.56 (s, 1H, ArC*H*), 7.43 – 7.28 (m, 6H, ArC*H*), 7.16 – 7.10 (m, 2H, ArC*H*), 4.70 (s, 2H, OCH_2Ph), 3.91 (s, 3H, ArOCH_3), 3.04 – 2.88 (m, 2H, CH_2CH_2Ar), 2.47 (dd, *J* = 6.8, 2.8 Hz, 1H, NCH_2), 2.34 (dd, *J* = 6.8, 2.6 Hz, 1H, NCH_2), 2.18 (ddd, *J* = 13.8, 11.6, 5.3 Hz, 1H, CH_2CH_2Ar), 2.03 (ddd, *J* = 13.8, 11.8, 5.6 Hz, 1H, CH_2CH_2Ar), 1.42 (s, 3H, (C)CH_3), 1.13 (d, *J* = 2.8 Hz, 1H, NCH_2), 1.11 (d, *J* = 2.6 Hz, 1H, NCH_2) ppm;

^{13}C NMR (126 MHz, $CDCl_3$): δ_C 157.3 (Ar*C*O), 139.6 (Ar*C*), 137.6 (Ar*C*), 133.1 (Ar*C*), 129.3 (Ar*C*), 129.0 (Ar*C*H), 128.5 (Ar*C*H), 127.9 (Ar*C*H), 127.4 (Ar*C*H), 127.3 (Ar*C*H), 127.0 (Ar*C*H), 126.2 (Ar*C*H), 118.9 (Ar*C*H), 105.8 (Ar*C*H), 74.4 ((*C*)OCH_2Ph), 64.9 (OCH_2Ph), 55.4 (ArOCH_3), 51.8 (NCH_2), 51.3 (NCH_2), 40.2 (CH_2CH_2Ar), 34.4 (*C*N(CH_2)$_2$), 29.9 (CH_2Ar), 20.9 ((C)CH_3) ppm.

HRMS (ESI^+): m/z calc'd for $C_{25}H_{27}NNaO_2$ $[M+Na]^+$: 396.1934 found: 396.1945.

IR (film): ν_{max} 3057, 2940, 1606, 1484, 1391, 1263, 1231, 1029 cm^{-1}.

3-(1-(Benzyloxy)-3-(5-methylfuran-2-yl)propyl)-1-azabicyclo[1.1.0]butane: 2n

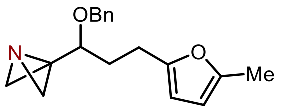

2n

Synthesised according to **General Procedure C** from: **9n** (286 mg, 1.48 mmol, 1.00 equiv), NaH (60% dispersion in mineral oil, 118 mg, 2.00 equiv) and BnBr (0.572 mL, 3.00 equiv). Purified by flash column chromatography (SiO_2; 86:14 hexane:EtOAc) to afford **2n** (327 mg, 1.15 mmol, 78%) as a yellow oil.

TLC: R_f = 0.27 (86:14 hexane:EtOAc).

NMR Spectroscopy (*see spectra*):

^{1}H NMR (400 MHz, $CDCl_3$): δ_H 7.38 – 7.26 (m, 5H, ArC*H*), 5.84 – 5.75 (m, 2H, ArC*H*), 4.78 (d, *J* = 11.6 Hz, 1H, OCH_2Ph), 4.54 (d, *J* = 11.6 Hz, 1H, OCH_2Ph), 3.75 (dd, *J* = 8.2, 4.7 Hz, 1H, C*H*OCH_2Ph), 2.81 (ddd, *J* = 15.1, 8.7, 5.9 Hz, 1H, CH_2CH_2Ar), 2.69 (ddd, *J* = 15.1, 7.9, 7.6 Hz, 1H, CH_2CH_2Ar), 2.39 (dd, *J* = 6.5, 2.8 Hz, 1H, NCH_2), 2.28 (dd, *J* = 6.5, 2.6 Hz, 1H, NCH_2), 2.23 (s, 3H, ArCH_3), 2.05 – 1.89 (m, 2H, CH_2CH_2Ar), 1.19 (d, *J* = 2.8 Hz, 1H, NCH_2), 1.16 (d, *J* = 2.6 Hz, 1H, NCH_2) ppm;

^{13}C NMR (126 MHz, $CDCl_3$): δ_C 153.7 (Ar*C*), 150.5 (Ar*C*), 138.5 (Ar*C*), 128.5 (Ar*C*H), 128.0 (Ar*C*H), 127.8 (Ar*C*H), 105.9 (Ar*C*H), 105.8 (Ar*C*H), 75.3 (*C*HOCH_2Ph), 71.9 (OCH_2Ph), 52.7 (NCH_2), 52.0 (NCH_2), 32.2 (CH_2CH_2Ar), 31.1 (*C*N(CH_2)$_2$), 24.2 (CH_2Ar), 13.7 (ArCH_3) ppm.

HRMS (ESI^+): m/z calc'd for $C_{18}H_{21}NNaO_2$ $[M+Na]^+$: 306.1464 found: 306.1466.

IR (film): ν_{max} 3031, 2945, 2860, 1570, 1454, 1218, 1100, 1070, 1021 cm^{-1}.

3-(3-(Benzo[*b*]thiophen-2-yl)-1-(benzyloxy)propyl)-1-azabicyclo[1.1.0]butane: 2o

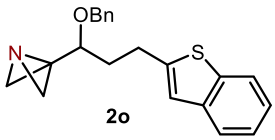

2o

Synthesised according to **General Procedure C** from: **9o** (348 mg, 1.42 mmol, 1.00 equiv), NaH (60% dispersion in mineral oil, 114 mg, 2.00 equiv) and BnBr (0.506 mL, 3.00 equiv). Purified by flash column chromatography (SiO_2; 88:12 hexane:EtOAc) to afford **2o** (319 mg, 0.951 mmol, 67%) as a colourless oil.

TLC: R_f = 0.22 (88:12 hexane:EtOAc).

NMR Spectroscopy (*see spectra*):

^{1}**H NMR** (500 MHz, $CDCl_3$): δ_H 7.75 (d, *J* = 9.0 Hz, 1H, ArC*H*), 7.64 (d, *J* = 7.8 Hz, 1H, ArC*H*), 7.39 – 7.27 (m, 7H, ArC*H*), 6.91 (s, 1H, ArC*H*), 4.81 (d, *J* = 11.6 Hz, 1H, OCH_2Ph), 4.56 (d, *J* = 11.6 Hz, 1H, OCH_2Ph), 3.81 (dd, *J* = 8.8, 4.0 Hz, 1H, C*H*OCH_2Ph), 3.14 (ddd, *J* = 14.8, 8.8, 5.5 Hz, 1H, CH_2CH_2Ar), 3.04 (app-dt, *J* = 14.8, 7.7 Hz, 1H, CH_2CH_2Ar), 2.40 (dd, *J* = 6.5, 2.7 Hz, 1H, NCH_2), 2.30 (dd, *J* = 6.5, 2.5 Hz, 1H, NCH_2), 2.18 – 1.99 (m, 2H, CH_2CH_2Ar), 1.22 (d, *J* = 2.5 Hz, 1H, NCH_2), 1.18 (d, *J* = 2.7 Hz, 1H, NCH_2) ppm;

^{13}**C NMR** (126 MHz, $CDCl_3$): δ_C 145.6 (Ar*C*), 140.3 (Ar*C*), 139.5 (Ar*C*), 138.4 (Ar*C*), 128.6 (Ar*C*H), 128.1 (Ar*C*H), 127.9 (Ar*C*H), 124.2 (Ar*C*H), 123.7 (Ar*C*H), 122.9 (Ar*C*H), 122.3 (Ar*C*H), 121.1 (Ar*C*H), 74.9 (*C*HOCH_2Ph), 71.9 (O*C*H_2Ph), 52.9 (N*C*H_2), 52.0 (N*C*H_2), 34.9 (*C*H_2CH_2Ar), 31.0 (*C*N(CH_2)$_2$), 26.8 (*C*H_2Ar) ppm.

HRMS (ESI^+): m/z calc'd for $C_{21}H_{22}NOS$ $[M+H]^+$: 336.141662 found: 336.141970.

IR (film): ν_{max} 3060, 2943, 1456, 1436, 1097, 1066 cm^{-1}.

3-(1-(Benzyloxy)-6-methylhept-5-en-1-yl)-1-azabicyclo[1.1.0]butane: 2p

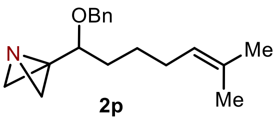

2p

Synthesised according to **General Procedure C** from: **9p** (145 mg, 0.800 mmol, 1.00 equiv), NaH (60% dispersion in mineral oil, 64.0 mg, 2.00 equiv) and BnBr (0.285 mL, 3.00 equiv). Purified by flash column chromatography (SiO_2; 98:2 toluene:Et_2O) to afford **2p** (174 mg, 0.640 mmol, 80%) as a colourless oil.

TLC: R_f = 0.23 (98:2 toluene:Et_2O).

NMR Spectroscopy (*see spectra*):

^{1}**H NMR** (500 MHz, $CDCl_3$): δ_H 7.38 – 7.27 (m, 5H, ArC*H*), 5.10 (app-t, *J* = 7.1 Hz, 1H, *H*C=C), 4.77 (d, *J* = 11.7 Hz, 1H, OCH_2Ph), 4.56 (d, *J* = 11.7 Hz, 1H, OCH_2Ph), 3.71 (dd, *J* = 8.2, 4.1 Hz, 1H, C*H*OCH_2Ph),

2.38 (dd, *J* = 6.5, 2.8 Hz, 1H, NC*H*2), 2.28 (dd, *J* = 6.5, 2.6 Hz, 1H, NC*H*2), 1.98 (app-q, *J* = 7.1 Hz, 2H, C*H*2(HC=C)), 1.73 – 1.65 (m, 4H, C*H*2CH2CH2(HC=C), C=C(C*H*3)2), 1.63 – 1.54 (m, 5H, C*H*2CH2CH2(HC=C), C=C(C*H*3)2, C*H*2CH2(HC=C)), 1.49 – 1.39 (m, 1H, C*H*2CH2(HC=C)), 1.19 (d, *J* = 2.8 Hz, 1H, NC*H*2), 1.16 (d, *J* = 2.6 Hz, 1H, NC*H*2) ppm;

^{13}C NMR (126 MHz, $CDCl_3$): δ_C 138.7 (Ar*C*), 131.9 (HC=*C*(CH3)2), 128.5 (H*C*=C(CH3)2), 127.9 (Ar*C*H), 127.7 (Ar*C*H), 124.5 (Ar*C*H), 76.2 (*C*HOCH2Ph), 71.7 (O*C*H2Ph), 52.8 (N*C*H2), 52.0 (N*C*H2), 33.2 *C*H2CH2CH2(HC=C), 31.2 (*C*N(CH2)2), 28.0 (*C*H2(HC=C)), 26.0 (*C*H2CH2(HC=C)), 25.9 (HC=C(*C*H3)2), 17.9 (HC=C(*C*H3)2) ppm.

HRMS (ESI$^+$): m/z calc'd for $C_{18}H_{25}NNaO$ [M+Na]$^+$: 294.182835 found: 294.183212.

IR (film): ν_{max} 2930, 2859, 1454, 1377, 1099, 1073 cm^{-1}.

3-(1-(Benzyloxy)-2,6-dimethylhept-5-en-1-yl)-1-azabicyclo[1.1.0]butane: 2q

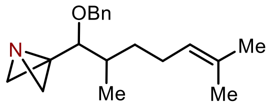

2q

Synthesised according to **General Procedure C** from: **9q** (293 mg, 1.50 mmol, 1.00 equiv), NaH (60% dispersion in mineral oil, 120 mg, 2.00 equiv) and BnBr (0.534 mL, 3.00 equiv). Purified by flash column chromatography (SiO_2; 92:8 hexane:EtOAc) to afford **2q** as a 1.6:1 mixture of diastereomers (329 mg, 1.15 mmol, 77%) as a colourless oil.

TLC: R_f = 0.23 (92:8 hexane:EtOAc).

NMR Spectroscopy (*see spectra*):

^{1}H NMR (500 MHz, $CDCl_3$) *Distinct diastereomer signals are labelled as d¹ and d²*: δ_H δ 7.35 – 7.27 (m, 5H, ArC*H*), 5.13 – 4.98 (m, 1H, *H*C=C), 4.77 (d, *J* = 11.8 Hz, 0.62H, *d^1*-OC*H*2Ph), 4.76 (d, *J* = 11.8 Hz, 0.38H, *d^2*-OC*H*2Ph), 4.49 (d, *J* = 11.8 Hz, 0.38H, *d^2*-OC*H*2Ph), 4.48 (d, *J* = 11.8 Hz, 0.62H, *d^1*-OC*H*2Ph), 3.52 (d, *J* = 4.9 Hz, 0.62H, *d^1*-C*H*OCH2Ph), 3.50 (d, *J* = 5.6 Hz, 0.38H, *d^2*-C*H*OCH2Ph), 2.53 – 2.45 (m, 1H, NC*H*2), 2.20 (dd, *J* = 6.4, 2.6 Hz, 1H, NC*H*2), 2.10 – 2.00 (m, 1H, C*H*2C=C), 1.99 – 1.90 (m, 1H, C*H*2C=C), 1.89 – 1.81 (m, 1H, C*H*(CH3)), 1.72 – 1.62 (m, 4H, HC=C(C*H*3)2, C*H*2CH2C=C), 1.59 (s, 3H, HC=C(C*H*3)2), 1.35 – 1.24 (m, 1H, C*H*2CH2C=C), 1.20 – 1.17 (m, 1H, NC*H*2), 1.15 – 1.11 (m, 1H, NC*H*2), 1.05 (d, *J* = 6.8 Hz, 1.14H, *d^2*-CH(C*H*3)), 1.04 (d, *J* = 6.9 Hz, 1.86H, *d^1*-CH(C*H*3)) ppm;

^{13}C NMR (126 MHz, $CDCl_3$): δ_C 139.0 and 138.9 (Ar*C*), 131.6 and 131.6 (HC=*C*(CH3)2), 128.4 (Ar*C*H), 127.8 and 127.8 (Ar*C*H), 127.5 (Ar*C*H), 124.7 and 124.7 (H*C*=C(CH3)2), 79.6 and 79.2 (*C*HOCH2Ph), 72.4 and 72.4 (O*C*H2Ph), 52.6 and 52.4 (N*C*H2), 51.5, and 51.4 (N*C*H2), 37.8 (*C*H(CH3)), 33.1 and 33.0 (*C*H2CH2C=C), 31.1 and 30.7 (*C*N(CH2)2), 25.9 (HC=C(*C*H3)2), 25.8 and 25.7 (CH2C=C), 17.8 and 17.8 (HC=C(*C*H3)2), 15.7 and 15.6 (CH(*C*H3)) ppm.

HRMS (ESI$^+$): m/z calc'd for $C_{19}H_{27}NNaO$ [M+Na]$^+$: 308.1985 found: 308.1979.

IR (film): ν_{max} 3032, 2916, 2878, 2856, 1454, 1377, 1090, 1071 cm^{-1}.

3-(1-(Methoxymethoxy)-3-phenylpropyl)-1-azabicyclo[1.1.0]butane

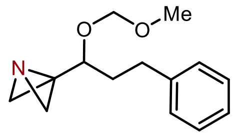

Synthesised according to **General Procedure C** from: **9a** (132 mg, 0.700 mmol, 1.00 equiv), NaH (60% dispersion in mineral oil, 56.0 mg, 2.00 equiv) and chloromethyl methyl ether (0.160 mL, 3.00 equiv). Purified by flash column chromatography (SiO_2; 4:1 hexane:EtOAc) to afford 3-(1-(methoxymethoxy)-3-phenylpropyl)-1-azabicyclo[1.1.0]butane (101 mg, 0.435 mmol, 62%) as a colourless oil.

TLC: R_f = 0.24 (4:1 hexane:EtOAc).

NMR Spectroscopy (*see spectra*):

> ^{1}**H NMR** (400 MHz, $CDCl_3$): δ_H 7.32 – 7.26 (m, 2H, ArC*H*), 7.24 – 7.16 (m, 3H, ArC*H*), 4.90 (d, *J* = 6.8 Hz, 1H, OC*H*$_2$OCH$_3$), 4.64 (d, *J* = 6.8 Hz, 1H, OC*H*$_2$OCH$_3$), 3.95 (dd, *J* = 7.9, 5.0 Hz, 1H, C*H*OCH$_2$OCH$_3$), 3.43 (s, 3H, OCH$_2$OC*H*$_3$), 2.89 (ddd, *J* = 13.7, 9.9, 6.0 Hz, 1H, CH$_2$C*H*$_2$Ph), 2.73 (ddd, *J* = 13.7, 9.6, 6.6 Hz, 1H, CH$_2$C*H*$_2$Ph), 2.37 (dd, *J* = 6.5, 2.7 Hz, 1H, NC*H*$_2$), 2.29 (dd, *J* = 6.5, 2.5 Hz, 1H, NC*H*$_2$), 2.09 – 1.87 (m, 2H, C*H*$_2$CH$_2$Ph), 1.22 – 1.14 (m, 2H, NC*H*$_2$) ppm;

> ^{13}**C NMR** (126 MHz, $CDCl_3$): δ_C 141.8 (Ar*C*), 128.6 (Ar*C*H), 128.5 (Ar*C*H), 126.1 (Ar*C*H), 95.7 (O*C*H$_2$OCH$_3$), 73.7 (*C*HOCH$_2$OCH$_3$), 55.8 (OCH$_2$O*C*H$_3$), 52.9 (N*C*H$_2$), 52.5 (N*C*H$_2$), 35.4 (*C*H$_2$CH$_2$Ph), 31.9 (*C*H$_2$Ph), 31.1 (*C*N(CH$_2$)$_2$) ppm.

HRMS (ESI$^+$): m/z calc'd for $C_{14}H_{19}NNaO_2$ [M+Na]$^+$: 256.130800 found: 256.131659.

IR (film): ν_{max} 3027, 2947, 1496, 1455, 1147, 1100, 1028 cm^{-1}.

1-(1-Azabicyclo[1.1.0]butan-3-yl)-3-phenylpropyl acetate

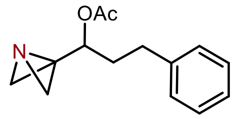

Synthesised according to **General Procedure D** from: **9a** (151 mg, 0.800 mmol, 1.00 equiv), ^{n}BuLi (1.6 M in hexane, 0.900 mL, 1.80 equiv) and acetic anhydride (0.151 mL, 2.00 equiv). Purified by flash column chromatography (SiO_2; 19:1 hexane:EtOAc) to afford 1-(1-azabicyclo[1.1.0]butan-3-yl)-3-phenylpropyl acetate (62.9 mg, 0.272 mmol, 34%)[A] as a colourless oil.

Notes: (**A**) Decomposition of product was observed during column chromatography, the yield of **115** was determined to be 72% by ^{1}H NMR analysis of the crude reaction mixture using dibromomethane as an internal standard.

TLC: R_f = 0.21 (19:1 hexane:EtOAc).

NMR Spectroscopy (*see spectra*):

^{1}H NMR (400 MHz, $CDCl_3$): δ_H 7.30 – 7.26 (m, 2H, ArC*H*), 7.21 – 7.15 (m, 3H, ArC*H*), 5.26 (dd, *J* = 8.4, 4.9 Hz, 1H, C*H*OAc), 2.71 (m, 2H, C*H*$_2$Ph), 2.37 (dd, *J* = 6.6, 2.3 Hz, 1H, NC*H*$_2$), 2.34 (dd, *J* = 6.6, 2.3 Hz, 1H, NC*H*$_2$), 2.07 (s, 3H, C*H*$_3$(C=O)), 2.05 – 1.91 (m, 2H, C*H*$_2$CH$_2$Ph), 1.20 (d, *J* = 2.3 Hz, 2H, NC*H*$_2$) ppm;

^{13}C NMR (126 MHz, $CDCl_3$): δ_C 170.5 (*C*=O), 141.1 (Ar*C*), 128.6 (Ar*C*H), 128.5 (Ar*C*H), 126.3 (Ar*C*H), 70.9 (*C*HOAc), 53.5 (N*C*H$_2$), 55.3 (N*C*H$_2$), 33.7 (*C*H$_2$CH$_2$Ph), 31.8 (*C*H$_2$Ph), 30.6 (*C*N(CH$_2$)$_2$), 21.1 (*C*H$_3$(C=O)) ppm.

HRMS (ESI$^+$): m/z calc'd for $C_{14}H_{17}NNaO_2$ $[M+Na]^+$: 254.115149 found: 254.116093.

IR (film): ν_{max} 3027, 2950, 1743 (C=O), 1372, 1233, 1029 cm^{-1}.

3-(1-(Benzyloxy)-2-phenylethyl)-1-azabicyclo[1.1.0]butane

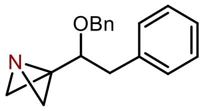

Synthesised according to **General Procedure C** from: 1-(1-Azabicyclo[1.1.0]butan-3-yl)-2-phenylethan-1-ol (238 mg, 1.36 mmol, 1.00 equiv), NaH (60% dispersion in mineral oil, 109 mg, 2.00 equiv) and BnBr (0.485 mL, 3.00 equiv). Purified by flash column chromatography (SiO_2; 86:14 hexane:EtOAc) to afford 3-(1-(Benzyloxy)-2-phenylethyl)-1-azabicyclo[1.1.0]butane (184 mg, 0.692 mmol, 51%) as a pale yellow solid.

TLC: R_f = 0.22 (86:14 hexane:EtOAc).

NMR Spectroscopy (*see spectra*):

^{1}H NMR (400 MHz, $CDCl_3$): δ_H 7.31 – 7.22 (m, 8H, ArC*H*), 7.16 – 7.12 (m, 2H, ArC*H*), 4.68 (d, *J* = 11.9 Hz, 1H, OC*H*$_2$Ph), 4.46 (d, *J* = 11.9 Hz, 1H, OC*H*$_2$Ph), 3.93 (dd, *J* = 8.4, 4.9 Hz, 1H, C*H*OCH$_2$Ph), 3.07 – 2.87 (m, 2H, C*H*$_2$Ph), 2.42 (dd, *J* = 6.5, 2.8 Hz, 1H, NC*H*$_2$), 2.24 (dd, *J* = 6.5, 2.5 Hz, 1H, NC*H*$_2$), 1.21 (dd, *J* = 2.8, 0.7 Hz, 1H, NC*H*$_2$), 1.15 (dd, *J* = 2.5, 0.7 Hz, 1H, NC*H*$_2$) ppm;

^{13}C NMR (126 MHz, $CDCl_3$): δ_C 138.3 (Ar*C*), 138.3 (Ar*C*), 129.7 (Ar*C*H), 128.4 (Ar*C*H), 128.4 (Ar*C*H), 127.8 (Ar*C*H), 127.6 (Ar*C*H), 126.5 (Ar*C*H), 77.4 (*C*HOCH$_2$Ph), 72.0 (O*C*H$_2$Ph), 52.9 (N*C*H$_2$), 52.2 (N*C*H$_2$), 40.3 (*C*H$_2$Ph), 31.3 (*C*N(CH$_2$)$_2$) ppm.

HRMS (ESI$^+$): m/z calc'd for $C_{18}H_{19}NNaO$ $[M+Na]^+$: 288.1359 found: 288.1358.

IR (film): ν_{max} 3029, 2943, 1496, 1454, 1095, 1074 cm^{-1}.

3-(1-(Benzyloxy)-4-phenylbutyl)-1-azabicyclo[1.1.0]butane

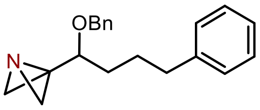

Synthesised according to General Procedure C from: 1-(1-Azabicyclo[1.1.0]butan-3-yl)-4-phenylbutan-1-ol (228 mg, 1.32 mmol, 1.00 equiv), NaH (60% dispersion in mineral oil, 106 mg, 2.00 equiv) and BnBr (0.470 mL, 3.00 equiv). Purified by flash column chromatography (SiO_2; 95:5 toluene:Et_2O) to afford 3-(1-(benzyloxy)-4-phenylbutyl)-1-azabicyclo[1.1.0]butane (375 mg, 1.28 mmol, 97%) as a colourless oil.

TLC: R_f = 0.30 (95:5 toluene:Et_2O).

NMR Spectroscopy (*see spectra*):

^{1}**H NMR** (500 MHz, $CDCl_3$): δ_H 7.36 – 7.27 (m, 7H, ArC*H*), 7.21 – 7.14 (m, 3H, ArC*H*), 4.77 (d, *J* = 11.7 Hz, 1H, OC*H*$_2$Ph), 4.54 (d, *J* = 11.7 Hz, 1H, OC*H*$_2$Ph), 3.73 (dd, *J* = 7.7, 4.5 Hz, 1H, C*H*OCH$_2$Ph), 2.61 (t, *J* = 7.3 Hz, 2H, CH$_2$C*H*$_2$Ph), 2.38 (dd, *J* = 6.5, 2.8 Hz, 1H, NC*H*$_2$), 2.26 (dd, *J* = 6.5, 2.6 Hz, 1H, NC*H*$_2$), 1.96 – 1.84 (m, 1H, C*H*$_2$CH$_2$Ph), 1.79 – 1.62 (m, 3H, C*H*$_2$CH$_2$Ph, C*H*$_2$CH$_2$CH$_2$Ph), 1.19 (d, *J* = 2.8 Hz, 1H, NC*H*$_2$), 1.15 (d, *J* = 2.6 Hz, 1H, NC*H*$_2$) ppm;

^{13}**C NMR** (126 MHz, $CDCl_3$): δ_C 142.4 (Ar*C*), 138.6 (Ar*C*), 128.6 (Ar*C*H), 128.5 (Ar*C*H), 128.5 (Ar*C*H), 128.0 (Ar*C*H), 127.8 (Ar*C*H), 125.9 (Ar*C*H), 76.0 (*C*HOCH$_2$Ph), 71.7 (O*C*H$_2$Ph), 52.8 (N*C*H$_2$), 52.0 (N*C*H$_2$), 36.0 (CH$_2$*C*H$_2$Ph), 33.3 (*C*H$_2$CH$_2$CH$_2$Ph), 31.2 (*C*N(CH$_2$)$_2$), 27.5 (*C*H$_2$CH$_2$Ph) ppm.

HRMS (ESI$^+$): m/z calc'd for $C_{20}H_{23}NNaO$ [M+Na]$^+$: 316.1672 found: 316.1701.

IR (film): ν_{max} 3027, 2942, 2861, 1496, 1454, 1101, 1090, 1079 cm^{-1}.

2.6. Synthesis of spiro-tetralins

***tert*-Butyl 2'-methoxy-3',4'-dihydro-2'*H*-spiro[azetidine-3,1'-naphthalene]-1-carboxylate: 4a**

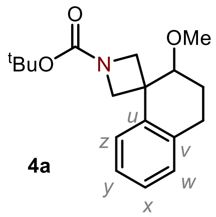

4a

Synthesised according to **General Procedure E** from: **2a** (40.7 mg, 0.200 mmol, 1.00 equiv), $HBF_4{\cdot}OEt_2$ (28.6 µL, 1.05 equiv), Et_3N (0.112 mL, 4.00 equiv) and Boc_2O (91.9 µL, 2.00 equiv). Purified by flash column chromatography (SiO_2; 4.7:1 hexane:EtOAc) to afford **4a** (38.1 mg, 0.126 mmol, 63%) as a colourless oil.

TLC: R_f = 0.26 (4.7:1 hexane:EtOAc).

NMR Spectroscopy (*see spectra*): *Doubling of signals due to presence of rotamers*

^{1}**H NMR** (500 MHz, $CDCl_3$): δ_H 7.62 (dd, *J* = 7.9, 1.3 Hz, 1H, ArC^z*H*), 7.28 – 7.24 (m, 1H, ArC^y*H*), 7.16

(app-td, *J* = 7.5, 1.3 Hz, 1H, ArC^x*H*), 7.06 (dd, *J* = 7.5, 1.3 Hz, 1H, ArC^w*H*), 4.27 (d, *J* = 8.6 Hz, 1H, NC*H*$_2$), 3.98 (m, 2H, NC*H*$_2$), 3.87 (d, *J* = 8.6 Hz, 1H, NC*H*$_2$), 3.59 (dd, *J* = 5.1, 4.4 Hz, 1H, C*H*OCH$_3$), 3.46 (s, 3H, OC*H*$_3$), 2.93 (app-dt, *J* = 16.8, 7.0 Hz, 1H, C*H*$_2$Ar), 2.70 (app-dt, *J* = 16.8, 6.0 Hz, 1H, C*H*$_2$Ar), 1.89 (br s, 2H, C*H*$_2$CH$_2$Ar) 1.48 (s, 9H, OC(C*H*$_3$)$_3$) ppm;

^{13}C NMR (126 MHz, CDCl$_3$): δ$_C$ 156.8 (*C*=O), 139.2 (Ar*C*^{u}), 135.7 (Ar*C*^{v}), 128.7 (Ar*C*^{w}H), 127.1 (Ar*C*^{y}H), 126.8 (Ar*C*^{x}H), 126.7 (Ar*C*^{z}H), 80.7 (*C*HOCH$_3$), 79.6 (O*C*(CH$_3$)$_3$), 61.8 (br N*C*H$_2$), 60.4 (br N*C*H$_2$), 59.3 (br N*C*H$_2$), 58.2 (br N*C*H$_2$), 56.9 (O*C*H$_3$), 41.1 (*spiro*-*C*), 28.6 (OC(*C*H$_3$)$_3$), 26.0 (*C*H$_2$Ar), 22.2 (*C*H$_2$CH$_2$Ar) ppm.

HRMS (ESI$^+$): m/z calc'd for $C_{18}H_{25}NNaO_3$ [M+Na]$^+$: 326.172664 found: 326.173007.

IR (film): ν_{max} 2930, 2885, 1698 (C=O), 1390, 1162, 1096 cm^{-1}.

tert-Butyl 2'-(benzyloxy)-3',4'-dihydro-2'*H*-spiro[azetidine-3,1'-naphthalene]-1-carboxylate: 4b

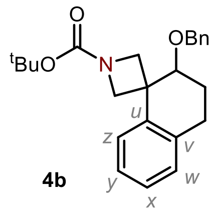

4b

Synthesised according to **General Procedure E** from: **2b** (55.9 mg, 0.200 mmol, 1.00 equiv), HBF$_4$·OEt$_2$ (28.6 µL, 1.05 equiv), Et$_3$N (0.112 mL, 4.00 equiv) and Boc$_2$O (91.9 µL, 2.00 equiv). Purified by flash column chromatography (SiO$_2$; 3:1 hexane:EtOAc) to afford **4b** (53.2 mg, 0.140 mmol, 70%) as a colourless oil.

TLC: R$_f$ = 0.25 (3:1 hexane:EtOAc).

NMR Spectroscopy (*see spectra*): *Doubling of signals due to presence of rotamers*

^{1}H NMR (400 MHz, CDCl$_3$): δ$_H$ 7.64 (dd, *J* = 7.9, 1.3 Hz, 1H, ArC^z*H*), 7.39 – 7.31 (m, 4H, ArC*H*), 7.31 – 7.24 (m, 2H, ArC*H,* ArC^y*H*), 7.16 (app-td, *J* = 7.6, 1.3 Hz, 1H, ArC^x*H*), 7.07 (dd, *J* = 7.6, 1.3 Hz, 1H, ArC^w*H*), 4.79 (d, *J* = 11.8 Hz, 1H, OC*H*$_2$Ph), 4.55 (d, *J* = 11.8 Hz, 1H, OC*H*$_2$Ph), 4.42 – 4.30 (m, 1H, NC*H*$_2$), 4.11 – 4.00 (m, 2H, NC*H*$_2$), 3.96 – 3.85 (m, 1H, NC*H*$_2$), 3.81 – 3.75 (m, 1H, C*H*OCH$_2$Ph), 2.97 (app-dt, *J* = 16.8, 6.4 Hz, 1H, C*H*$_2$Ar), 2.74 (app-dt, *J* = 16.8, 6.4 Hz, 1H, C*H*$_2$Ar), 2.03 – 1.81 (m, 2H, C*H*$_2$CH$_2$Ar), 1.47 (s, 9H, OC(C*H*$_3$)$_3$) ppm;

^{13}C NMR (126 MHz, CDCl$_3$): δ$_C$ 156.8 (*C*=O), 139.6 (Ar*C*^{u}), 138.5 (Ar*C*), 135.7 (Ar*C*^{v}), 128.6 (Ar*C*H), 128.5 (Ar*C*H), 127.8 (Ar*C*H), 127.8 (Ar*C*H), 127.1 (Ar*C*H), 126.8 (Ar*C*H), 126.7 (Ar*C*H), 79.6 (O*C*(CH$_3$)$_3$), 78.4 (*C*HOCH$_2$Ph), 70.9 (O*C*H$_2$Ph), 61.4 (br N*C*H$_2$), 60.2 (br N*C*H$_2$), 59.5 (br N*C*H$_2$), 58.3 (br N*C*H$_2$), 41.2 (*spiro*-*C*), 28.6 (OC(*C*H$_3$)$_3$), 26.6 (*C*H$_2$Ar), 23.1 (*C*H$_2$CH$_2$Ar) ppm.

HRMS (ESI$^+$): m/z calc'd for $C_{24}H_{29}NNaO_3$ [M+Na]$^+$: 402.203964 found: 402.204489.

IR (film): ν_{max} 2973, 2931, 2883, 1698 (C=O), 1390, 1164, 1110 cm^{-1}.

tert-Butyl 2'-(allyloxy)-3',4'-dihydro-2'_H_-spiro[azetidine-3,1'-naphthalene]-1-carboxylate: 4c

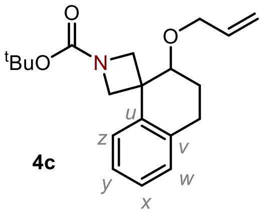

4c

Synthesised according to **General Procedure E** from: **2c** (45.9 mg, 0.200 mmol, 1.00 equiv), HBF_4·OEt_2 (28.6 µL, 1.05 equiv), Et_3N (0.112 mL, 4.00 equiv) and Boc_2O (91.9 µL, 2.00 equiv). Purified by flash column chromatography (SiO_2; 7.3:1 hexane:EtOAc) to afford **4c** (37.8 mg, 0.115 mmol, 57%) as a colourless oil.

TLC: R_f = 0.20 (7.3:1 hexane:EtOAc).

NMR Spectroscopy (see spectra): *Doubling of signals due to presence of rotamers*

^{1}H NMR (400 MHz, $CDCl_3$): δ_H 7.63 (dd, *J* = 7.9, 1.3 Hz, 1H, ArC^z*H*), 7.31 – 7.21 (m, 1H, ArC^y*H*), 7.16 (app-td, *J* = 7.6, 1.3 Hz, 1H, ArC^x*H*), 7.06 (dd, *J* = 7.6, 1.3 Hz, 1H, ArC^w*H*), 5.95 (app-ddt, *J* = 17.2, 10.6, 5.6 Hz, 1H, *H*C=CH_2), 5.30 (app-dq, *J* = 17.2, 1.5 Hz, 1H, HC=CH_2), 5.17 (app-dq, *J* = 10.6, 1.5 Hz, 1H, HC=CH_2), 4.33 (d, *J* = 8.6 Hz, 1H, NCH_2), 4.24 (app-ddt, *J* = 12.8, 5.6, 1.5 Hz, 1H, OCH_2(C=C)), 4.06 – 3.95 (m, 2H, NCH_2), 3.95 – 3.85 (m, 2H, OCH_2(C=C), NCH_2), 3.74 – 3.68 (m, 1H, C*H*OCH_2(C=C)), 2.93 (app-dt, *J* = 16.8, 6.5 Hz, 1H, CH_2Ar), 2.71 (app-dt, *J* = 16.8, 6.5 Hz, 1H, CH_2Ar), 1.88 (br s, 2H, CH_2CH_2Ar), 1.48 (s, 9H, OC(CH_3)$_3$) ppm;

^{13}C NMR (126 MHz, $CDCl_3$): δ_C 156.8 (*C*=O), 138.6 (Ar*C*^{u}), 135.7 (Ar*C*^{v}), 135.1 (H*C*=CH_2), 128.6 (Ar*C*^{w}H), 127.1 (Ar*C*^{y}H), 126.7 (Ar*C*^{z}H), 126.7 (Ar*C*^{x}H), 117.0 (HC=*C*H_2), 79.6 (O*C*(CH_3)$_3$), 78.5 (*C*HOCH_2(C=C)), 70.1 (O*C*H_2(HC=C)), 61.6 (br N*C*H_2), 60.1 (br N*C*H_2), 59.5 (br N*C*H_2), 58.2 (br N*C*H_2), 41.2 (*spiro-C*), 28.6 (OC(*C*H_3)$_3$), 26.6 (*C*H_2Ar), 23.2 (*C*H_2CH_2Ar) ppm.

HRMS (ESI$^+$): m/z calc'd for $C_{20}H_{27}NNaO_3$ $[M+Na]^+$: 352.188314 found: 352.189959.

IR (film): ν_{max} 2974, 2885, 1701 (C=O), 1392, 1165, 1111 cm^{-1}.

tert-Butyl 2'-((triethylsilyl)oxy)-3',4'-dihydro-2'_H_-spiro[azetidine-3,1'-naphthalene]-1-carboxylate: 4d

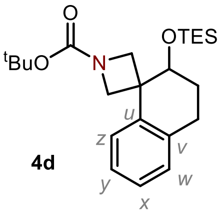

4d

Synthesised according to **General Procedure E** from: **2d** (60.7 mg, 0.200 mmol, 1.00 equiv), HBF_4·OEt_2 (28.6 µL, 1.05 equiv), Et_3N (0.112 mL, 4.00 equiv) and Boc_2O (91.9 µL, 2.00 equiv). Purified by flash column chromatography (SiO_2; 99:1 toluene:Et_2O) to afford **4d** (38.9 mg, 0.096 mmol, 48%) as a colourless oil.

TLC: R_f = 0.22 (99:1 toluene:Et_2O).

NMR Spectroscopy (see spectra): *Doubling of signals due to presence of rotamers*

^{1}H NMR (400 MHz, $CDCl_3$): δ_H 7.66 (dd, *J* = 7.7, 1.4 Hz, 1H, ArC^z*H*), 7.27 (app-td, *J* = 7.7, 1.4 Hz, 1H, ArC^y*H*), 7.16 (app-td, *J* = 7.6, 1.4 Hz, 1H, ArC^x*H*), 7.05 (dd, *J* = 7.6, 1.4 Hz, 1H, ArC^w*H*), 4.37 (d, *J* = 8.0 Hz, 1H, NCH_2), 4.09 (d, *J* = 8.0 Hz, 1H, NCH_2), 3.95 (m, 2H, C*H*OSi, NCH_2), 3.68 (d, *J* = 7.8 Hz, 1H, NCH_2), 2.89 (app-dt, *J* = 16.7, 5.3 Hz, 1H, CH_2Ar), 2.79 (ddd, *J* = 16.7, 9.9, 5.3 Hz, 1H, CH_2Ar), 1.94 – 1.82 (m, 1H, CH_2CH$_2$Ar), 1.76 – 1.63 (m, 1H, CH_2CH$_2$Ar), 1.47 (s, 9H, OC(CH_3)$_3$), 0.99 (t, *J* = 7.9 Hz, 9H Si(CH$_2$CH_3)$_3$)), 0.66 (q, *J* = 7.9 Hz, 6H, Si(CH_2CH$_3$)$_3$) ppm;

^{13}C NMR (126 MHz, $CDCl_3$): δ_C 156.7 (*C*=O), 140.3 (Ar*C*^{u}), 135.5 (Ar*C*^{v}), 128.5 (Ar*C*^{w}H), 127.0 (Ar*C*^{y}H), 126.9 (Ar*C*^{x}H), 126.7 (Ar*C*^{z}H), 79.4 (O*C*(CH$_3$)$_3$), 72.2 (*C*HOSi), 59.5 (br N*C*H$_2$), 58.9 (br N*C*H$_2$), 58.4 (br N*C*H$_2$), 57.6 (br N*C*H$_2$), 42.2 (*spiro*-*C*), 28.9 (*C*H$_2$Ar), 28.6 (OC(*C*H$_3$)$_3$), 27.8 (*C*H$_2$CH$_2$Ar), 7.1 (Si(CH$_2$*C*H$_3$)$_3$), 5.4 (Si(*C*H$_2$CH$_3$)$_3$) ppm.

HRMS (ESI$^+$): m/z calc'd for $C_{23}H_{37}NNaO_3Si$ [M+Na]$^+$: 426.243491 found: 426.243093.

IR (film): ν_{max} 2954, 2877, 1703 (C=O), 1390, 1168, 1108 cm^{-1}.

***tert*-Butyl 2'-hydroxy-3',4'-dihydro-2'*H*-spiro[azetidine-3,1'-naphthalene]-1-carboxylate: 4e**

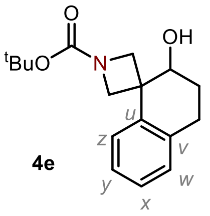

4e

Synthesised according to **General Procedure E** from: **2e** (52.3 mg, 0.200 mmol, 1.00 equiv), $HBF_4{\cdot}OEt_2$ (28.6 µL, 1.05 equiv), Et_3N (0.112 mL, 4.00 equiv) and Boc_2O (91.9 µL, 2.00 equiv). Purified by flash column chromatography (SiO_2; 65:35 hexane:EtOAc) to afford **4e** (34.2 mg, 0.118 mmol, 59%) as a colourless oil.

TLC: R_f = 0.22 (65:35 hexane:EtOAc).

NMR Spectroscopy (see spectra): *Doubling of signals due to presence of rotamers*

^{1}H NMR (400 MHz, $CDCl_3$): δ_H 7.64 (d, *J* = 7.9 Hz, 1H, ArC^z*H*), 7.34 – 7.24 (m, 1H, ArC^y*H*), 7.18 (app-t, *J* = 7.6 Hz, 1H, ArC^x*H*), 7.08 (d, *J* = 7.6 Hz, 1H, ArC^w*H*), 4.32 (d, *J* = 8.6 Hz, 1H, NCH_2), 4.12 – 4.03 (m, 2H, NCH_2, C*H*OH), 3.97 (d, *J* = 8.6 Hz, 1H, NCH_2), 3.91 (d, *J* = 8.5 Hz, 1H, NCH_2), 2.96 (app-dt, *J* = 17.1, 6.4 Hz, 1H, CH_2Ar), 2.81 (app-dt, *J* = 17.1, 6.8 Hz, 1H, CH_2Ar), 2.09 – 1.89 (m, 2H, CH_2CH$_2$Ar, CHO*H*), 1.88 – 1.79 (m, 1H, CH_2CH$_2$Ar), 1.48 (s, 9H, OC(CH_3)$_3$) ppm;

^{13}C NMR (126 MHz, $CDCl_3$): δ_C 156.7 (*C*=O), 138.9 (Ar*C*^{u}), 135.4 (Ar*C*^{v}), 128.8 (Ar*C*^{w}H), 127.2 (Ar*C*^{y}H), 127.0 (Ar*C*^{x}H), 127.0 (Ar*C*^{z}H), 79.8 (O*C*(CH$_3$)$_3$), 71.7 (*C*HOH), 60.5 (br N*C*H$_2$), 59.7 (br N*C*H$_2$), 58.7 (br N*C*H$_2$), 57.6 (br N*C*H$_2$), 41.6 (*spiro*-*C*), 28.6 (OC(*C*H$_3$)$_3$), 27.6 (*C*H$_2$Ar), 26.5 (*C*H$_2$CH$_2$Ar) ppm.

HRMS (ESI$^+$): m/z calc'd for $C_{17}H_{23}NNaO_3$ [M+Na]$^+$: 312.1570 found: 312.1591.

IR (film): ν_{max} 3424 (O-H), 2931, 1677 (C=O), 1408, 1165 cm^{-1}.

tert-Butyl 2'-(benzyloxy)-5'-methyl-3',4'-dihydro-2'_H_-spiro[azetidine-3,1'-naphthalene]-1-carboxylate: 4f

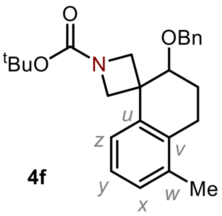

Synthesised according to **General Procedure E** from: **2f** (58.7 mg, 0.200 mmol, 1.00 equiv), $HBF_4 \cdot OEt_2$ (28.6 µL, 1.05 equiv), Et_3N (0.112 mL, 4.00 equiv) and Boc_2O (91.9 µL, 2.00 equiv). Purified by flash column chromatography (SiO_2; 88:12 hexane:EtOAc) to afford **4f** (59.0 mg, 0.150 mmol, 75%) as a colourless oil.

TLC: R_f = 0.21 (88:12 hexane:EtOAc).

NMR Spectroscopy (see spectra): *Doubling of signals due to presence of rotamers*

^{1}**H NMR** (400 MHz, $CDCl_3$): δ_H 7.52 (d, *J* = 7.7 Hz, 1H, ArC^z*H*), 7.41 – 7.28 (m, 5H, ArC*H*), 7.20 (app-t, *J* = 7.7 Hz, 1H, ArC^y*H*), 7.05 (d, *J* = 7.7 Hz, 1H, ArC^x*H*), 4.79 (d, *J* = 11.8 Hz, 1H, OC*H*$_2$Ph), 4.55 (d, *J* = 11.8 Hz, 1H, OC*H*$_2$Ph), 4.33 (br s, 1H, NC*H*$_2$), 4.07 – 3.97 (m, 1H, NC*H*$_2$), 3.96 – 3.85 (m, 2H, NC*H*$_2$), 3.79 – 3.70 (m, 1H, C*H*OCH$_2$Ph), 2.79 (app-dt, *J* = 17.2, 6.5 Hz, 1H, C*H*$_2$Ar), 2.58 (app-dt, *J* = 17.2, 6.5 Hz, 1H, C*H*$_2$Ar), 2.23 (s, 3H, ArC*H*$_3$), 2.03 – 1.87 (m, 2H, C*H*$_2$CH$_2$Ar), 1.47 (s, 9H, OC(C*H*$_3$)$_3$) ppm;

^{13}**C NMR** (126 MHz, $CDCl_3$): δ_C 156.8 (*C*=O), 139.5 (ArC^u), 138.5 (Ar*C*), 136.0 (ArC^v), 134.3 (ArC^w), 128.5 (Ar*C*H), 128.3 (Ar*C*H), 127.8 (Ar*C*H), 127.8 (Ar*C*H), 126.8 (Ar*C*H), 124.5 (Ar*C*H), 79.5 (O*C*(CH$_3$)$_3$), 77.9 (*C*HOCH$_2$Ph), 70.9 (O*C*H$_2$Ph), 61.5 (br N*C*H$_2$), 60.2 (br N*C*H$_2$), 59.6 (br N*C*H$_2$), 58.4 (br N*C*H$_2$), 41.4 (*spiro-C*), 28.6 (OC(*C*H$_3$)$_3$), 24.1 (*C*H$_2$Ar), 22.8 (*C*H$_2$CH$_2$Ar), 19.9 (Ar*C*H$_3$) ppm.

HRMS (ESI$^+$): m/z calc'd for $C_{25}H_{31}NNaO_3$ $[M+Na]^+$: 416.2196 found: 416.2205.

IR (film): ν_{max} 2971, 2932, 2882, 1697 (C=O), 1455, 1389, 1159, 1095 cm^{-1}.

tert-Butyl 2'-(benzyloxy)-6',8'-dimethoxy-3',4'-dihydro-2'_H_-spiro[azetidine-3,1'-naphthalene]-1-carboxylate: 4g

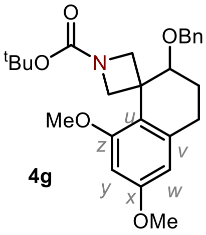

Synthesised according to **General Procedure E** from: **2g** (67.9 mg, 0.200 mmol, 1.00 equiv), $HBF_4 \cdot OEt_2$ (28.6 µL, 1.05 equiv), Et_3N (0.112 mL, 4.00 equiv) and Boc_2O (91.9 µL, 2.00 equiv). Purified by flash column chromatography (SiO_2; 80:20 hexane:EtOAc) to afford **4g** (74.8 mg, 0.170 mmol, 85%) as a colourless oil.

TLC: R_f = 0.28 (80:20 hexane:EtOAc).

NMR Spectroscopy (see spectra): *Doubling of signals due to presence of rotamers*

^{1}**H NMR** (500 MHz, $CDCl_3$): δ_H 7.42 – 7.27 (m, 5H, ArC*H*), 6.34 (d, *J* = 2.5 Hz, 1H, ArC^w*H*), 6.22 (d, *J* =

2.5 Hz, 1H, ArC^y*H*), 4.74 (d, *J* = 11.7, 0.5H, OC*H*$_2$Ph), 4.73 (d, *J* = 11.7, 0.5H, OC*H*$_2$Ph), 4.56 (d, *J* = 11.7 Hz, 0.5H, OC*H*$_2$Ph), 4.53 (d, *J* = 11.7 Hz, 0.5H, OC*H*$_2$Ph), 4.49 (d, *J* = 8.1 Hz, 0.5H, NC*H*$_2$), 4.39 (d, *J* = 8.1 Hz, 0.5H, NC*H*$_2$), 4.24 (d, *J* = 7.9 Hz, 0.5H, NC*H*$_2$), 4.16 (d, *J* = 7.9 Hz, 0.5H, NC*H*$_2$), 4.10 (d, *J* = 8.0 Hz, 1H, NC*H*$_2$), 3.82 (s, 3H, ArOC*H*$_3$), 3.80 – 3.74 (m, 4H, ArOC*H*$_3$, C*H*OCH$_2$Ph), 3.65 (d, *J* = 8.2 Hz, 0.5H, NC*H*$_2$), 3.63 (d, *J* = 8.2 Hz, 0.5H, NC*H*$_2$), 3.04 – 2.89 (m, 1H, C*H*$_2$Ar), 2.69 – 2.53 (m, 1H, C*H*$_2$Ar), 2.07 – 1.88 (m, 1H, C*H*$_2$CH$_2$Ar), 1.87 – 1.67 (m, 1H, C*H*$_2$CH$_2$Ar), 1.48 (s, 9H, OC(C*H*$_3$)$_3$) ppm;

^{13}**C NMR** (126 MHz, CDCl$_3$): δ_C 160.7 (Ar*C*^{x}), 159.1 (Ar*C*^{z}), 157.5 (*C*=O), 139.5 (Ar*C*), 138.5 and 138.4 (Ar*C*^{v}), 128.5 (Ar*C*H), 128.0 (Ar*C*H), 127.7 (Ar*C*H), 118.3 and 118.2 (Ar*C*^{u}), 104.7 (Ar*C*^{w}H), 97.5 (Ar*C*^{y}H), 80.3 (O*C*(CH$_3$)$_3$), 78.9 (*C*HOCH$_2$Ph), 71.2 (O*C*H$_2$Ph), 60.0 (N*C*H$_2$), 58.4 (N*C*H$_2$), 57.0 (N*C*H$_2$), 55.8 (N*C*H$_2$), 55.5 (ArO*C*H$_3$), 55.4 (ArO*C*H$_3$), 39.7 (*spiro*-*C*), 28.7 (OC(*C*H$_3$)$_3$), 26.8 and 26.3 (*C*H$_2$Ar), 22.6 and 22.3 (*C*H$_2$CH$_2$Ar) ppm.

HRMS (ESI$^+$): m/z calc'd for $C_{26}H_{33}NNaO_5$ [M+Na]$^+$: 462.2251 found: 462.2252.

IR (film): v_{max} 2937, 2880, 1692 (C=O), 1606, 1390, 1159, 1114 cm^{-1}.

***tert*-Butyl 2'-(benzyloxy)-6'-fluoro-3',4'-dihydro-2'*H*-spiro[azetidine-3,1'-naphthalene]-1-carboxylate: 4h**

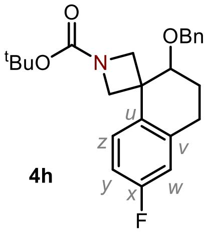

4h

Synthesised according to **General Procedure E** from: **2h** (59.5 mg, 0.200 mmol, 1.00 equiv), $HBF_4{\cdot}OEt_2$ (28.6 µL, 1.05 equiv), Et_3N (0.112 mL, 4.00 equiv) and Boc_2O (91.9 µL, 2.00 equiv). Purified by flash column chromatography (SiO_2; 85:15 hexane:EtOAc) to afford **4h** (23.9 mg, 0.060 mmol, 30%) as a colourless oil.

TLC: R_f = 0.26 (85:15 hexane:EtOAc).

NMR Spectroscopy (*see spectra*): *Doubling of signals due to presence of rotamers*

^{1}**H NMR** (500 MHz, CDCl$_3$): δ_H 7.58 (dd, *J* = 8.7, 5.6 Hz, 1H, ArC^z*H*), 7.37 – 7.28 (m, 5H, ArC*H*), 6.96 (app-td, *J* = 8.5, 2.8 Hz, 1H, ArC^y*H*), 6.76 (dd, *J* = 9.5, 2.6 Hz, 1H, ArC^w*H*), 4.77 (d, *J* = 11.9 Hz, 1H, OC*H*$_2$Ph), 4.54 (d, *J* = 11.9 Hz, 1H, OC*H*$_2$Ph), 4.31 (br s, 1H, NC*H*$_2$), 3.99 (br s, 1H, NC*H*$_2$), 3.90 (br s, 1H, NC*H*$_2$), 3.85 – 3.73 (m, 2H, NC*H*$_2$, C*H*OCH$_2$Ph), 2.95 (app-dt, *J* = 16.9, 6.8 Hz, 1H, C*H*$_2$Ar), 2.70 (app-dt, *J* = 16.9, 6.1 Hz, 1H, C*H*$_2$Ar), 1.91 (br s, 2H, C*H*$_2$CH$_2$Ar), 1.46 (s, 9H, OC(C*H*$_3$)$_3$) ppm;

^{13}**C NMR** (126 MHz, CDCl$_3$): δ_C 161.5 (d, *J* = 245.5 Hz, Ar*C*^{x}), 156.7 (*C*=O), 138.3 (Ar*C*), 138.0 (d, *J* = 7.4 Hz, Ar*C*^{v}), 135.2 (d, *J* = 3.0 Hz, Ar*C*^{u}), 128.6 (Ar*C*H), 128.5 (d, *J* = 8.3 Hz, Ar*C*^{z}H), 127.9 (Ar*C*H), 127.9 (Ar*C*H), 114.8 (d, *J* = 20.6 Hz, Ar*C*^{y}H), 114.3 (d, *J* = 21.5 Hz, Ar*C*^{w}H), 79.7 (O*C*(CH$_3$)$_3$), 78.2 (*C*HOCH$_2$Ph), 71.0 (O*C*H$_2$Ph), 61.7 (br N*C*H$_2$), 60.3 (br N*C*H$_2$), 59.5 (br N*C*H$_2$), 58.2 (br N*C*H$_2$), 40.8 (*spiro*-*C*), 28.6 (OC(*C*H$_3$)$_3$), 26.5 (*C*H$_2$Ar), 22.8 (*C*H$_2$CH$_2$Ar) ppm;

^{19}**F NMR** (376 MHz, CDCl$_3$): δ_F –116.5 ppm.

HRMS (ESI+): m/z calc'd for $C_{24}H_{28}NNaO_3F$ [M+Na]+: 420.1945 found: 420.1940.

IR (film): v_{max} 2932, 2885, 1699 (C=O), 1498, 1391, 1166, 1106 cm^{-1}.

tert-Butyl 2'-(benzyloxy)-2'-methyl-3',4'-dihydro-2'*H*-spiro[azetidine-3,1'-naphthalene]-1-carboxylate: 4i

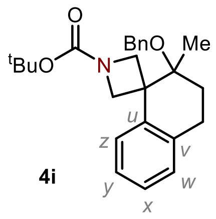

4i

Synthesised according to **General Procedure E** from: **2i** (58.7 mg, 0.200 mmol, 1.00 equiv), $HBF_4·OEt_2$ (28.6 µL, 1.05 equiv), Et_3N (0.112 mL, 4.00 equiv) and Boc_2O (91.9 µL, 2.00 equiv). Purified by flash column chromatography (SiO_2; 95:5 toluene:Et_2O) to afford **4i** (39.4 mg, 0.100 mmol, 50%) as a colourless oil.

TLC: R_f = 0.24 (95:5 toluene:Et_2O).

NMR Spectroscopy (*see spectra*): *Doubling of signals due to presence of rotamers*

^{1}H NMR (400 MHz, $CDCl_3$): $δ_H$ 7.68 (d, *J* = 7.9 Hz, 1H, ArC^z*H*), 7.38 – 7.28 (m, 5H, ArC*H*), 7.27 – 7.20 (m, 1H, ArC^y*H*), 7.18 (app-td, *J* = 7.5, 1.3 Hz, 1H, ArC^x*H*), 7.09 (dd, *J* = 7.5, 1.3 Hz, 1H, ArC^w*H*), 4.69 – 4.50 (m, 3H, OC*H*$_2$Ph, NC*H*$_2$), 4.50 – 4.40 (m, 1H, NC*H*$_2$), 4.02 (d, *J* = 8.7 Hz, 1H, NC*H*$_2$), 3.58 (br s, 1H, NC*H*$_2$), 2.98 – 2.74 (m, 2H, C*H*$_2$Ar), 2.08 (br s, 1H, C*H*$_2$CH$_2$Ar), 1.74 (br s, 1H, C*H*$_2$CH$_2$Ar), 1.50 – 1.38 (m, 9H, OC(C*H*$_3$)$_3$), 1.23 (s, 3H, (C)C*H*$_3$) ppm;

^{13}C NMR (126 MHz, $CDCl_3$): $δ_C$ 157.2 (*C*=O), 141.2 (ArC^u), 139.8 (Ar*C*), 134.3 (ArC^v), 128.7 (Ar*C*H), 128.4 (Ar*C*H), 127.1 (Ar*C*H), 127.1 (Ar*C*H), 126.7 (Ar*C*H), 126.6 (Ar*C*H), 126.4 (Ar*C*H), 79.4 (O*C*(CH$_3$)$_3$), 75.2 (O*C*H$_2$Ph), 63.2 ((*C*)CH$_3$), 59.9 (br N*C*H$_2$), 58.6 (br N*C*H$_2$), 56.7 (br N*C*H$_2$), 55.4 (br N*C*H$_2$), 45.8 (*spiro-C*), 28.6 (OC(*C*H$_3$)$_3$), 28.2 (*C*H$_2$Ar), 27.4 (*C*H$_2$CH$_2$Ar), 17.1 ((C)*C*H$_3$) ppm.

HRMS (ESI+): m/z calc'd for $C_{25}H_{31}NNaO_3$ [M+Na]+: 416.2196 found: 416.2203.

IR (film): v_{max} 2974, 2857, 1701 (C=O), 1389, 1146, 1110 cm^{-1}.

tert-Butyl 2'-(benzyloxy)-2'*H*,4'*H*-dispiro[azetidine-3,1'-naphthalene-3',1''-cyclohexane]-1-carboxylate: 4j

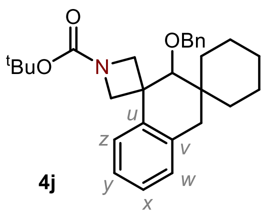

4j

Synthesised according to **General Procedure E** from: **2j** (69.5 mg, 0.200 mmol, 1.00 equiv), $HBF_4·OEt_2$ (28.6 µL, 1.05 equiv), Et_3N (0.112 mL, 4.00 equiv) and Boc_2O (91.9 µL, 2.00 equiv). Purified by flash column chromatography (SiO_2; 90:10 hexane:EtOAc) to afford **4j** (80.6 mg, 0.180 mmol, 90%) as a white semi-solid.

TLC: R_f = 0.25 (90:10 hexane:EtOAc).

NMR Spectroscopy (see spectra): *Doubling of signals due to presence of rotamers*

^{1}H NMR (400 MHz, $CDCl_3$): δ_H 7.71 (dd, *J* = 7.9, 1.3 Hz, 1H, ArC^z*H*), 7.41 – 7.27 (m, 6H, ArC*H*, ArC^y*H*), 7.18 (app-td, *J* = 7.5, 1.3 Hz, 1H, ArC^x*H*), 7.05 (dd, *J* = 7.5, 1.3 Hz, 1H, ArC^w*H*), 4.87 (d, *J* = 11.2 Hz, 1H, OC*H*$_2$Ph), 4.81 (d, *J* = 11.2 Hz, 1H, OC*H*$_2$Ph), 4.53 (d, *J* = 8.5 Hz, 1H, NC*H*$_2$), 4.19 (br s, 1H, NC*H*$_2$), 3.82 (m, 2H, NC*H*$_2$), 3.53 (s, 1H, C*H*OCH$_2$Ph), 3.10 (d, *J* = 16.6 Hz, 1H, C*H*$_2$Ar), 2.54 (d, *J* = 16.6 Hz, 1H, C*H*$_2$Ar), 1.66 – 1.21 (m, 19H, cy-C*H*$_2$, OC(C*H*$_3$)$_3$) ppm;

^{13}C NMR (126 MHz, $CDCl_3$): δ_C 156.4 (*C*=O), 141.1 (Ar*C*^{u}), 138.7 (Ar*C*), 134.4 (Ar*C*^{v}), 128.8 (Ar*C*H), 128.5 (Ar*C*H), 127.7 (Ar*C*H), 127.5 (Ar*C*H), 127.2 (Ar*C*H), 126.9 (Ar*C*H), 126.3 (Ar*C*H), 87.2 (*C*HOCH$_2$Ph), 79.6 (O*C*(CH$_3$)$_3$), 76.8 (*C*HOCH$_2$Ph), 63.1 (br N*C*H$_2$), 61.8 (br N*C*H$_2$), 61.0 (br N*C*H$_2$), 59.8 (br N*C*H$_2$), 41.1 (*spiro*-*C*), 39.4 ((*C*)-cy), 36.6 (*C*H$_2$Ar), 33.8 (cy-*C*H$_2$), 33.5 (cy-*C*H$_2$), 28.6 (OC(*C*H$_3$)$_3$), 26.3 (cy-*C*H$_2$), 21.6 (cy-*C*H$_2$) ppm.

HRMS (ESI$^+$): m/z calc'd for $C_{29}H_{37}NNaO_3$ $[M+Na]^+$: 470.266565 found: 470.264649.

IR (film): ν_{max} 2932, 2857, 1699 (C=O), 1391, 1158, 1114, 1068 cm^{-1}.

Scale-up reaction of 4j

Scale-up reaction was performed according to a modified **General Procedure E** from: **2j** (1.04 g, 3.00 mmol, 1.00 equiv), $CHCl_3$ (30 mL), $HBF_4{\cdot}OEt_2$ (0.429 mL, 1.05 equiv), Et_3N (1.67 mL, 4.00 equiv) and Boc_2O (1.38 mL, 2.00 equiv). Purified by flash column chromatography (90:10 hexane:EtOAc) to afford **4j** (1.15 g, 2.58 mmol, 86%) as a white semi-solid.

tert-Butyl 3'-(benzyloxy)-2'-methylspiro[azetidine-3,4'-chromane]-1-carboxylate: 4k

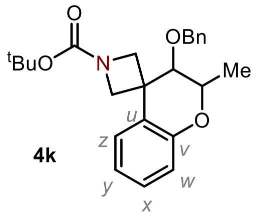

4k

Synthesised according to **General Procedure E** from: **2k** (59.1 mg, 0.200 mmol, 1.00 equiv), $HBF_4{\cdot}OEt_2$ (28.6 µL, 1.05 equiv), Et_3N (0.112 mL, 4.00 equiv) and Boc_2O (91.9 µL, 2.00 equiv). Purified by flash column chromatography (SiO_2; 85:15 hexane:EtOAc) to afford **4k** as a 1:1 mixture of diastereomers (50.6 mg, 0.128 mmol, 64%) as a colourless oil.

TLC: R_f = 0.28 (85:15 hexane:EtOAc).

NMR Spectroscopy (see spectra): *Doubling of signals due to presence of rotamers*

^{1}H NMR (400 MHz, $CDCl_3$) *Distinct diastereomer signals are labelled as d^1 and d^2*: δ_H 7.59 – 7.54 (m, 1H, ArC^z*H*), 7.43 – 7.29 (m, 5H, ArC*H*), 7.19 – 7.12 (m, 1H, ArC^y*H*), 7.06 – 6.99 (m, 1H, ArC^x*H*), 6.80 (dd, *J* =

8.2, 1.3 Hz, 1H, ArC^{w}*H*), 4.92 – 4.80 (m, 1H, OC*H*$_2$Ph), 4.81 – 4.67 (m, 1H, OC*H*$_2$Ph), 4.51 – 4.30 (m, 1.5H, NC*H*$_2$, *d*^{1}-C*H*(CH$_3$)OAr), 4.21 – 3.74 (m, 4H, NC*H*$_2$, *d*^{2}-C*H*(CH$_3$)OAr, *d*^{1}-C*H*OCH$_2$Ph), 3.55 (d, *J* = 8.3 Hz, 1H, *d*^{2}-C*H*OCH$_2$Ph), 1.52 – 1.42 (m, 11.5H, *d*^{1}-CHC*H*$_3$, OC(C*H*$_3$)$_3$), 1.29 (d, *J* = 6.7 Hz, 1.5H, *d*^{2}-CHC*H*$_3$) ppm;

^{13}C NMR (126 MHz, CDCl$_3$): δ$_C$ 156.7 (*C*=O), 153.0 and 152.2 (Ar*C*^{v}), 137.9 (Ar*C*), 128.7 and 128.6 (Ar*C*H), 128.1 (Ar*C*H), 127.9 (Ar*C*H), 127.0 (Ar*C*H), 126.4 (Ar*C*H), 125.6 (Ar*C*^{u}), 121.9 and 121.8 (Ar*C*H), 117.1 and 116.7 (Ar*C*H), 79.8 and 77.9 (C*H*OCH$_2$Ph), 79.8 (O*C*(CH$_3$)$_3$), 74.0 (O*C*H$_2$Ph), 72.4 and 70.3 (C*H*(CH$_3$)OAr), 60.9 (br N*C*H$_2$), 59.5 (br N*C*H$_2$), 59.2 (br N*C*H$_2$), 58.0 (br N*C*H$_2$), 37.8 (*spiro*-*C*), 28.6 (OC(*C*H$_3$)$_3$), 18.0 and 15.1 (CHC*H*$_3$) ppm. *Weak azetidine ^{13}C NMR signals due to severe peak broadening, chemical shifts confirmed with HSQC spectroscopy.*

HRMS (ESI$^+$): m/z calc'd for $C_{24}H_{29}NNaO_4$ [M+Na]$^+$: 418.198879 found: 418.198451.

IR (film): ν_{max} 2976, 2884, 1702 (C=O), 1489, 1454, 1392, 1170, 1113 cm^{-1}.

***tert*-Butyl 3'-(benzyloxy)-2',3'-dihydro-1'*H*-spiro[azetidine-3,4'-phenanthrene]-1-carboxylate: 4l**

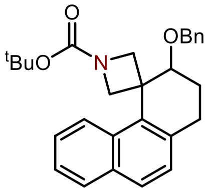

4l

Synthesised according to **General Procedure E** from: **2l** (65.9 mg, 0.200 mmol, 1.00 equiv), HBF$_4$·OEt$_2$ (28.6 µL, 1.05 equiv), Et$_3$N (0.112 mL, 4.00 equiv) and Boc$_2$O (91.9 µL, 2.00 equiv). Purified by flash column chromatography (SiO$_2$; 90:10 hexane:EtOAc) to afford **4l** (35.9 mg, 0.084 mmol, 42%) as white solid.

TLC: R$_f$ = 0.20 (90:10 hexane:EtOAc).

NMR Spectroscopy (see spectra): *Doubling of signals due to presence of rotamers*

^{1}H NMR (500 MHz, CDCl$_3$): δ$_H$ 8.64 – 8.60 (m, 1H, ArC*H*), 7.82 (dd, *J* = 8.0, 1.6 Hz, 1H, ArC*H*), 7.64 (d, *J* = 8.4 Hz, 1H, ArC*H*), 7.52 (app-t, *J* = 8.6 Hz, 1H, ArC*H*), 7.47 – 7.45 (app-t, *J* = 8.0 Hz, 1H, ArC*H*), 7.46 – 7.43 (m, 5H, ArC*H*), 7.16 (d, *J* = 8.4 Hz, 1H, ArC*H*), 4.82 (d, *J* = 11.9 Hz, 1H, OC*H*$_2$Ph), 4.63 – 4.44 (m, 3.5H, OC*H*$_2$Ph, NC*H*$_2$), 4.38 (d, *J* = 9.0 Hz, 0.5H, NC*H*$_2$), 4.04 (d, *J* = 8.8 Hz, 0.5H, NC*H*$_2$), 3.98 (d, *J* = 8.8 Hz, 0.5H, NC*H*$_2$), 3.78 (m, 1H, C*H*OCH$_2$Ph), 3.17 – 3.07 (m, 1H, C*H*$_2$Ar), 2.96 – 2.84 (m, 1H, C*H*$_2$Ar), 2.07 – 1.90 (m, 2H, C*H*$_2$CH$_2$Ar), 1.49 (s, 9H, OC(C*H*$_3$)$_3$) ppm;

^{13}C NMR (126 MHz, CDCl$_3$): δ$_C$ 156.9 (*C*=O), 138.3 (Ar*C*), 136.0 (Ar*C*), 133.5 (Ar*C*), 132.7 (Ar*C*), 131.8 (Ar*C*), 129.6 (Ar*C*H), 128.6 (Ar*C*H), 128.1 (Ar*C*H), 127.8 (Ar*C*H), 127.8 (Ar*C*H), 127.6 (Ar*C*H), 126.5 (Ar*C*H), 124.9 (Ar*C*H), 124.2 (Ar*C*H), 80.9 (*C*HOCH$_2$Ph), 79.8 (O*C*(CH$_3$)$_3$), 71.2 (O*C*H$_2$Ph), 60.3 (br N*C*H$_2$), 59.1 (br N*C*H$_2$), 58.2 (br N*C*H$_2$), 57.0 (br N*C*H$_2$), 42.1 (*spiro*-*C*), 28.6 (OC(*C*H$_3$)$_3$), 28.6 (*C*H$_2$Ar), 22.2 (*C*H$_2$CH$_2$Ar) ppm.

HRMS (ESI^+): m/z calc'd for $C_{28}H_{31}NNaO_3$ $[M+Na]^+$: 452.219615 found: 452.217906.

IR (film): v_{max} 2975, 1699 (C=O), 1387, 1366, 1158, 1093 cm^{-1}.

tert-Butyl 2-(benzyloxy)-2-methyl-3,4-dihydro-2_H_-spiro[anthracene-1,3'-azetidine]-1'-carboxylate: 4m

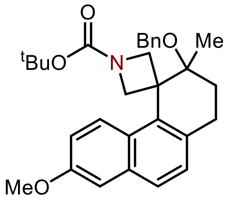

4m

Synthesised according to **General Procedure E** from: **2m** (74.7 mg, 0.200 mmol, 1.00 equiv), $HBF_4·OEt_2$ (28.6 µL, 1.05 equiv), Et_3N (0.112 mL, 4.00 equiv) and Boc_2O (91.9 µL, 2.00 equiv). Purified by flash column chromatography (SiO_2; 85:15 hexane:EtOAc) to afford **4m** (43.6 mg, 0.092 mmol, 46%) as a white solid.

TLC: R_f = 0.28 (85:15 hexane:EtOAc).

NMR Spectroscopy (see spectra): *Doubling of signals due to presence of rotamers*

> **^{1}H NMR** (400 MHz, $CDCl_3$): δ_H 8.74 (br s, 1H, ArC*H*), 7.55 (d, *J* = 8.3 Hz, 1H, ArC*H*), 7.35 – 7.29 (m, 4H), 7.23 – 7.13 (m, 3H), 7.10 (d, *J* = 8.3 Hz, 1H, ArC*H*), 4.75 – 4.08 (m, 6H, OC*H*$_2$Ph, NC*H*$_2$), 3.92 (s, 3H, ArOC*H*$_3$), 2.94 (br s, 2H, C*H*$_2$Ar), 2.17 (br s, 1H, C*H*$_2$CH$_2$Ar), 1.76 (br s, 1H, C*H*$_2$CH$_2$Ar), 1.49 – 1.22 (m, 12H, (C)C*H*$_3$, OC(C*H*$_3$)$_3$) ppm;

> **^{13}C NMR** (126 MHz, $CDCl_3$): δ_C 157.2 and 156.8 (*C*=O), 156.5 (Ar*C*OCH$_3$), 139.6 (Ar*C*), 139.6 (Ar*C*), 134.9 (Ar*C*), 128.3 (Ar*C*H), 128.0 (Ar*C*), 127.8 (Ar*C*H), 127.0 and 127.0 (Ar*C*H), 126.9 and 126.9 (Ar*C*H), 126.7 (Ar*C*H), 126.0 (Ar*C*H), 125.9 (Ar*C*), 118.1 and 118.1 (Ar*C*H), 107.8 (Ar*C*H), 79.5 (O*C*(CH$_3$)$_3$), 77.0 ((*C*)OCH$_2$Ph), 63.4 (O*C*H$_2$Ph), 58.2 (N*C*H$_2$), 57.0 (N*C*H$_2$), 55.3 and 55.2 (ArO*C*H$_3$), 46.3 (*spiro-C*), 28.9 (*C*H$_2$Ar), 28.4 and 28.4 (OC(*C*H$_3$)$_3$), 27.1 (*C*H$_2$CH$_2$Ar), 16.7 ((C)*C*H$_3$) ppm.

HRMS (ESI^+): m/z calc'd for $C_{30}H_{35}NNaO_4$ $[M+Na]^+$: 496.2458 found: 496.2460.

IR (film): v_{max} 2975, 2932, 1697 (C=O), 1377, 1244, 1150, 1112 cm^{-1}.

tert-Butyl 5'-(benzyloxy)-2'-methyl-6',7'-dihydro-5'_H_-spiro[azetidine-3,4'-benzofuran]-1-carboxylate: 4n

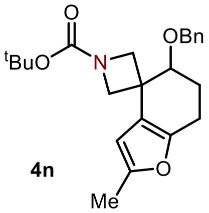

4n

Synthesised according to **General Procedure E** from: **2n** (56.7 mg, 0.200 mmol, 1.00 equiv), $HBF_4·OEt_2$ (28.6 µL, 1.05 equiv), Et_3N (0.112 mL, 4.00 equiv) and Boc_2O (91.9 µL, 2.00 equiv). Purified by flash column

chromatography (SiO_2; 88:12 hexane:EtOAc) to afford **4n** (29.2 mg, 0.076 mmol, 38%) as a colourless oil.

TLC: R_f = 0.21 (88:12 hexane:EtOAc).

NMR Spectroscopy (see spectra): *Doubling of signals due to presence of rotamers*

^{1}**H NMR** (400 MHz, $CDCl_3$): δ_H 7.38 – 7.27 (m, 5H, ArC*H*), 6.04 (s, 1H, ArC*H*), 4.75 (d, *J* = 11.8 Hz, 1H, OC*H*$_2$Ph), 4.55 (d, *J* = 11.8 Hz, 1H, OC*H*$_2$Ph), 4.23 (d, *J* = 8.2 Hz, 1H, NC*H*$_2$), 3.87 (br s, 1H, NC*H*$_2$), 3.86 – 3.74 (m, 2H, NC*H*$_2$), 3.72 – 3.62 (m, 1H, C*H*OCH$_2$Ph), 2.68 (app-dt, *J* = 16.3, 6.0 Hz, 1H, C*H*$_2$Ar), 2.53 (app-dt, *J* = 16.3, 6.5 Hz, 1H, C*H*$_2$Ar), 2.25 (s, 3H, ArC*H*$_3$), 1.98 – 1.83 (m, 2H, C*H*$_2$CH$_2$Ar), 1.45 (s, 9H, OC(C*H*$_3$)$_3$) ppm;

^{13}**C NMR** (126 MHz, $CDCl_3$): δ_C 156.7 (*C*=O), 151.6 (Ar*C*), 147.7 (Ar*C*), 138.5 (Ar*C*), 128.6 (Ar*C*H), 127.9 (Ar*C*H), 127.8 (Ar*C*H), 121.1 (Ar*C*), 104.0 (Ar*C*H), 79.4 (O*C*(CH$_3$)$_3$), 78.4 (*C*HOCH$_2$Ph), 71.5 (O*C*H$_2$Ph), 59.2 (N*C*H$_2$), 57.8 (N*C*H$_2$), 57.2 (N*C*H$_2$), 56.1 (N*C*H$_2$), 37.9 (*spiro*-*C*), 28.6 (OC(*C*H$_3$)$_3$), 23.6 (*C*H$_2$CH$_2$Ar), 20.4 (*C*H$_2$Ar), 13.8 (Ar*C*H$_3$) ppm.

HRMS (ESI$^+$): m/z calc'd for $C_{23}H_{29}NNaO_4$ $[M+Na]^+$: 406.1989 found: 406.1987.

IR (film): ν_{max} 2947, 2880, 1701 (C=O), 1393, 1170, 1112 cm^{-1}.

***tert*-Butyl 2'-(benzyloxy)-3',4'-dihydro-2'*H*-spiro[azetidine-3,1'-dibenzo[*b*,*d*]thiophene]-1-carboxylate: 4o**

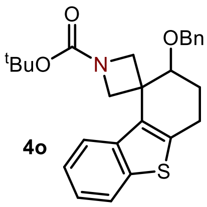

4o

Synthesised according to **General Procedure E** from: **2o** (67.1 mg, 0.200 mmol, 1.00 equiv), $HBF_4{\cdot}OEt_2$ (28.6 µL, 1.05 equiv), Et_3N (0.112 mL, 4.00 equiv) and Boc_2O (91.9 µL, 2.00 equiv). Purified by flash column chromatography (SiO_2; 85:15 hexane:EtOAc) to afford **4o** (63.7 mg, 0.146 mmol, 73%) as a colourless oil.

TLC: R_f = 0.26 (85:15 hexane:EtOAc).

NMR Spectroscopy (see spectra): *Doubling of signals due to presence of rotamers*

^{1}**H NMR** (500 MHz, $CDCl_3$): δ_H 8.07 (s, 1H, ArC*H*), 7.77 (d, *J* = 7.9 Hz, 1H, ArC*H*), 7.45 – 7.28 (m, 7H, ArC*H*), 4.83 (d, *J* = 11.6 Hz, 1H, OC*H*$_2$Ph), 4.61 (d, *J* = 11.6 Hz, 1H, OC*H*$_2$Ph), 4.45 – 4.24 (m, 3H, NC*H*$_2$), 4.10 – 3.96 (m, 1H, NC*H*$_2$), 3.89 – 3.78 (m, 1H, C*H*OCH$_2$Ph), 3.00 (app-dt, *J* = 16.9, 5.8 Hz, 1H, C*H*$_2$Ar), 2.90 – 2.77 (m, 1H, C*H*$_2$Ar), 2.21 – 1.87 (m, 2H, C*H*$_2$CH$_2$Ar), 1.50 (s, 9H, OC(C*H*$_3$)$_3$) ppm;

^{13}**C NMR** (126 MHz, $CDCl_3$): δ_C 156.7 (*C*=O), 139.5 (Ar*C*), 138.3 (Ar*C*), 138.3 (Ar*C*), 138.2 (Ar*C*), 130.6 (Ar*C*), 128.6 (Ar*C*H), 127.9 (Ar*C*H), 127.8 (Ar*C*H), 124.6 (Ar*C*H), 123.9 (Ar*C*H), 122.8 (Ar*C*H), 122.0 (Ar*C*H), 79.8 (O*C*(CH$_3$)$_3$), 79.4 (*C*HOCH$_2$Ph), 71.5 (O*C*H$_2$Ph), 58.4 (N*C*H$_2$), 57.0 (N*C*H$_2$), 56.3 (N*C*H$_2$), 55.1 (N*C*H$_2$), 40.7 (*spiro*-*C*), 28.6 (OC(*C*H$_3$)$_3$), 24.0 (*C*H$_2$CH$_2$Ar), 23.5 (*C*H$_2$Ar) ppm.

HRMS (ESI$^+$): m/z calc'd for $C_{26}H_{29}NSO_3$ $[M+H]^+$: 436.1941 found: 436.1931.

IR (film): ν_{max} 2974, 2885, 1696 (C=O), 1390, 1157, 1118 cm^{-1}.

tert-Butyl (5_R_*,9_S_*)-5-(benzyloxy)-9-(prop-1-en-2-yl)-2-azaspiro[3.5]nonane-2-carboxylate: 4p

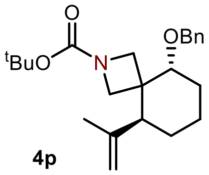

4p

Synthesised according to **General Procedure E** from: **2p** (54.3 mg, 0.200 mmol, 1.00 equiv), $HBF_4{\cdot}OEt_2$ (28.6 µL, 1.05 equiv), Et_3N (0.112 mL, 4.00 equiv) and Boc_2O (91.9 µL, 2.00 equiv). Purified by flash column chromatography (SiO_2; 90:10 hexane:EtOAc) to afford **4p** (40.8 mg, 0.110 mmol, 55%) as a colourless oil.

TLC: R_f = 0.21 (90:10 hexane:EtOAc).

NMR Spectroscopy (_see spectra_): _Doubling of signals due to presence of rotamers_

^{1}**H NMR** (500 MHz, $CDCl_3$): δ_H 7.38 – 7.27 (m, 5H, ArC*H*), 4.94 (s, 1H, C=C*H*$_2$), 4.72 – 4.60 (m, 2H, C=C*H*$_2$, OC*H*$_2$Ph), 4.44 (d, *J* = 11.9 Hz, 1H, OC*H*$_2$Ph), 3.94 – 3.53 (m, 5H, NC*H*$_2$, C*H*OCH$_2$Ph), 2.58 (dd, *J* = 8.4, 3.5 Hz, 1H, C*H*(C=C)), 1.79 (s, 3H, C*H*$_3$(C=C)), 1.68 (br s, 1H, CH(OCH$_2$Ph)C*H*$_2$), 1.62 – 1.50 (m, 2H, CH(OCH$_2$Ph)C*H*$_2$, (C=C)CHCH$_2$C*H*$_2$), 1.49 – 1.44 (m, 12H, (C=C)CHC*H*$_2$, (C=C)CHCH$_2$C*H*$_2$, OC(C*H*$_3$)$_3$) ppm;

^{13}**C NMR** (126 MHz, $CDCl_3$): δ_C 156.5 (*C*=O), 145.4 (CH$_3$(*C*=CH$_2$)), 139.0 (Ar*C*), 128.5 (Ar*C*H), 127.6 (Ar*C*H), 127.6 (Ar*C*H), 113.8 (CH$_3$(C=*C*H$_2$)), 79.2 (O*C*(CH$_3$)$_3$), 79.0 (*C*HOCH$_2$Ph), 70.8 (O*C*H$_2$Ph), 56.6 (br N*C*H$_2$), 55.3 (br N*C*H$_2$), 54.9 (br N*C*H$_2$), 53.6 (br N*C*H$_2$), 45.8 (*C*H(C=C)), 42.3 (*spiro-C*), 28.6 (OC(*C*H$_3$)$_3$), 27.2 ((C=C)CH*C*H$_2$), 25.4 (CH(OCH$_2$Ph)*C*H$_2$), 23.7 (*C*H$_3$(C=CH$_2$)), 20.1 ((C=C)CHCH$_2$*C*H$_2$) ppm.

HRMS (ESI^+): m/z calc'd for $C_{23}H_{33}NNaO_3$ $[M+Na]^+$: 394.235265 found: 394.236904.

IR (film): ν_{max} 2933, 2863, 1701 (C=O), 1393, 1365, 1145, 1094 cm^{-1}.

tert-Butyl-5-(benzyloxy)-6-methyl-9-(prop-1-en-2-yl)-2-azaspiro[3.5]nonane-2-carboxylate: 4q

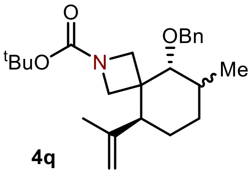

4q

Synthesised according to **General Procedure E** from: **2q** (57.1 mg, 0.200 mmol, 1.00 equiv), $HBF_4{\cdot}OEt_2$ (28.6 µL, 1.05 equiv), Et_3N (0.112 mL, 4.00 equiv) and Boc_2O (91.9 µL, 2.00 equiv). Purified by flash column chromatography (SiO_2; 92:8 hexane:EtOAc) to afford **4q** as a 1:1.6 mixture of diastereomers[A] (37.3 mg, 0.097 mmol, 48%) as a white solid.

Notes: (**A**) The mixture of diastereomers could not be effectively characterised using NMR spectroscopy so a further column was performed to separate the major and minor diastereomers (95:5 toluene:Et_2O) to allow full characterisation and confirm the formation of the product.

TLC: R_f = 0.21 (92:8 hexane:EtOAc).

NMR Spectroscopy (see spectra): *Doubling of signals due to presence of rotamers*

Data given for major diastereomer.

^{1}**H NMR** (500 MHz, $CDCl_3$): δ_H 7.39 – 7.27 (m, 5H, ArC*H*), 4.95 (s, 1H, C=C*H*$_2$), 4.81 (s, 1H, C=C*H*$_2$), 4.74 (d, *J* = 11.7 Hz, 1H, OC*H*$_2$Ph), 4.67 (d, *J* = 11.7 Hz, 1H, OC*H*$_2$Ph), 3.97 (d, *J* = 9.0 Hz, 1H, NC*H*$_2$), 3.81 (br s, 1H, NC*H*$_2$), 3.71 – 3.53 (m, 2H, NC*H*$_2$, C*H*OCH$_2$Ph), 3.44 (d, *J* = 9.0 Hz, 1H, NC*H*$_2$), 2.54 (dd, *J* = 12.2, 3.1 Hz, 1H, C*H*(C=C)), 1.83 (s, 3H, C*H*$_3$(C=C)), 1.62 – 1.50 (m, 2H, C*H*(CH$_3$), (C=C)CHC*H*$_2$), 1.50 – 1.36 (m, 12H, OC(C*H*$_3$)$_3$, (C=C)CHC*H*$_2$, (C=C)CHCH$_2$C*H*$_2$), 1.04 (d, *J* = 6.8 Hz, 3H, OCHCH(C*H*$_3$)) ppm;

^{13}**C NMR** (126 MHz, $CDCl_3$): δ_C 156.5 (*C*=O), 146.4 (CH$_3$(*C*=CH$_2$)), 139.3 (Ar*C*), 128.5 (Ar*C*H), 127.6 (Ar*C*H), 127.1 (Ar*C*H), 114.3 (CH$_3$(C=*C*H$_2$)), 86.4 (*C*HOCH$_2$Ph), 79.3 (O*C*(CH$_3$)$_3$), 75.3 (O*C*H$_2$Ph), 56.4 (br N*C*H$_2$), 55.1 (br N*C*H$_2$), 54.7 (br N*C*H$_2$), 53.5 (br N*C*H$_2$), 43.7 (*CH*(C=C)), 43.1 (*spiro*-*C*), 33.3 (OCH*C*H(CH$_3$)), 28.7 (CH(CH$_3$)*C*H$_2$), 28.6 (OC(*C*H$_3$)$_3$), 27.9 ((C=C)CH*C*H$_2$), 23.3 (*C*H$_3$(C=CH$_2$)), 18.8 (OCHCH(*C*H$_3$)) ppm.

HRMS (ESI$^+$): m/z calc'd for $C_{24}H_{35}NNaO_3$ [M+Na]$^+$: 408.250915 found: 408.252006.

IR (film): ν_{max} 2965, 2928, 2875, 1703 (C=O), 1393, 1153, 1093, 1067 cm^{-1}.

2'-(Benzyloxy)-1-tosyl-2'*H*,4'*H*-dispiro[azetidine-3,1'-naphthalene-3',1''-cyclohexane]: 4r

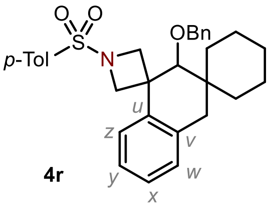

4r

Synthesised according to **General Procedure E** from: **2j** (69.5 mg, 0.200 mmol, 1.00 equiv), HBF_4·OEt_2 (28.6 µL, 1.05 equiv), Et_3N (0.112 mL, 4.00 equiv) and *p*-toluenesulfonyl chloride (76.3 mg, 2.00 equiv). Purified by flash column chromatography (SiO_2; 85:15 hexane:EtOAc) to afford **4r** (72.6 mg, 0.145 mmol, 72%) as a white solid.

TLC: R_f = 0.22 (85:15 hexane:EtOAc).

NMR Spectroscopy (see spectra):

^{1}**H NMR** (500 MHz, $CDCl_3$): δ_H 7.79 (d, *J* = 8.2 Hz, 2H, ArC*H*), 7.50 (dd, *J* = 7.4, 1.8 Hz, 1H, ArC^z*H*), 7.39 – 7.28 (m, 5H, ArC*H*), 7.22 (d, *J* = 6.9 Hz, 2H, ArC*H*), 7.18 – 7.08 (m, 2H, ArC^y*H*, ArC^x*H*), 7.03 – 6.97 (m, 1H, ArC^w*H*), 4.60 (d, *J* = 11.6 Hz, 1H, OC*H*$_2$Ph), 4.49 (d, *J* = 11.6 Hz, 1H, OC*H*$_2$Ph), 4.29 (d, *J* = 8.2 Hz, 1H, NC*H*$_2$), 4.02 (d, *J* = 8.0 Hz, 1H, NC*H*$_2$), 3.83– 3.79 (m, 2H, NC*H*$_2$), 3.27 (s, 1H, C*H*OCH$_2$Ph), 2.97 (d,

J = 16.5 Hz, 1H, C*H*$_2$Ar), 2.50 (d, *J* = 16.5 Hz, 1H, C*H*$_2$Ar), 2.39 (s, 3H, ArC*H*$_3$), 1.61 – 1.54 (m, 1H, cy-C*H*$_2$), 1.52 – 1.20 (m, 8H, cy-C*H*$_2$), 1.14 – 1.03 (m, 1H, cy-C*H*$_2$) ppm;

^{13}C NMR (126 MHz, CDCl$_3$): δ$_C$ 144.3 (Ar*C*), 139.2 (Ar*C^u*), 138.4 (Ar*C*), 134.7 (Ar*C^v*), 132.2 (Ar*C*), 129.9 (Ar*C*H), 128.9 (Ar*C*H), 128.6 (Ar*C*H), 128.4 (Ar*C*H), 127.6 (Ar*C*H), 127.2 (Ar*C*H), 127.2 (Ar*C*H), 126.9 (Ar*C*H), 126.6 (Ar*C*H), 87.7 (*C*HOCH$_2$Ph), 76.5 (O*C*H$_2$Ph), 63.7 (N*C*H$_2$), 61.3 (N*C*H$_2$), 40.4 (*spiro*-*C*), 39.0 ((*C*)-cy), 36.0 (*C*H$_2$Ar), 32.7 (cy-*C*H$_2$), 32.2 (cy-*C*H$_2$), 26.3 (cy-*C*H$_2$), 21.7 (Ar*C*H$_3$), 21.5 (cy-*C*H$_2$) ppm.

HRMS (ESI$^+$): m/z calc'd for $C_{31}H_{35}NNaSO_3$ [M+Na]$^+$: 524.2230 found: 524.2220.

IR (film): ν_{max} 2930, 2857, 1453, 1345 (S=O), 1158, 1068 cm^{-1}.

Benzyl 2'-(benzyloxy)-2'*H*,4'*H*-dispiro[azetidine-3,1'-naphthalene-3',1''-cyclohexane]-1-carboxylate: 4s

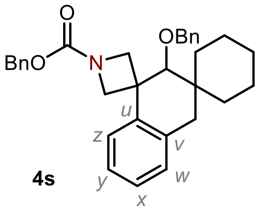

4s

Synthesised according to **General Procedure E** from: **2j** (69.5 mg, 0.200 mmol, 1.00 equiv), $HBF_4{\cdot}OEt_2$ (28.6 µL, 1.05 equiv), Et_3N (0.112 mL, 4.00 equiv) and CbzCl (57.1 µL, 2.00 equiv). Purified by flash column chromatography (SiO_2; 85:15 hexane:EtOAc) to afford **4s** (73.5 mg, 0.153 mmol, 76%) as a white solid.

TLC: R_f = 0.22 (85:15 hexane:EtOAc).

NMR Spectroscopy (*see spectra*): *Doubling of signals due to presence of rotamers*

^{1}H NMR (500 MHz, CDCl$_3$): δ$_H$ 7.68 (dd, *J* = 8.0, 1.3 Hz, 1H, ArC^z*H*), 7.37 – 7.27 (m, 11H, ArC*H*, ArC^y*H*), 7.18 (app-td, *J* = 7.3, 1.3 Hz, 1H, ArC^x*H*), 7.05 (d, *J* = 7.3 Hz, 1H, ArC^w*H*), 5.13 (br s, 2H, (C=O)OC*H*$_2$Ph), 4.89 (br s, 1H, OC*H*$_2$Ph), 4.79 (d, *J* = 11.5 Hz, 1H, OC*H*$_2$Ph), 4.63 (d, *J* = 8.5 Hz, 1H, NC*H*$_2$), 4.24 (br s, 1H, NC*H*$_2$), 3.99 (br s, 1H, NC*H*$_2$), 3.86 (br s, 1H, NC*H*$_2$), 3.55 (s, 1H, C*H*OCH$_2$Ph), 3.10 (d, *J* = 16.5 Hz, 1H, C*H*$_2$Ar), 2.54 (d, *J* = 16.5 Hz, 1H, C*H*$_2$Ar), 1.68 – 1.21 (m, 10H, cy-C*H*$_2$) ppm;

^{13}C NMR (126 MHz, CDCl$_3$): δ$_C$ 156.6 (*C*=O), 140.6 (Ar*C^u*), 138.6 (Ar*C*), 136.9 (Ar*C*), 134.4 (Ar*C^v*), 128.9 (Ar*C*H), 128.6 (Ar*C*H), 128.5 (Ar*C*H), 128.1 (Ar*C*H), 128.1 (Ar*C*H), 127.7 (Ar*C*H), 127.3 (Ar*C*H), 127.2 (Ar*C*H), 127.1 (Ar*C*H), 126.2 (Ar*C*H), 87.8 and 87.3 (*C*HOCH$_2$Ph), 77.4 (O*C*H$_2$Ph), 66.8 ((C=O)O*C*H$_2$Ph), 63.0 (br N*C*H$_2$), 62.5 (br N*C*H$_2$), 61.0 (br N*C*H$_2$), 60.2 (br N*C*H$_2$), 41.6 (*spiro*-*C*), 39.4 ((*C*)-cy), 36.5 (*C*H$_2$Ar), 33.7 (cy-*C*H$_2$), 30.3 and 30.2 (cy-*C*H$_2$), 26.3 (cy-*C*H$_2$), 21.6 and 21.6 (cy-*C*H$_2$) ppm.

HRMS (ESI$^+$): m/z calc'd for $C_{32}H_{35}NNaO_3$ [M+Na]$^+$: 504.2509 found: 504.2499.

IR (film): ν_{max} 2931, 2856, 1705 (C=O), 1411, 1351, 1110 cm^{-1}.

(2'-(Benzyloxy)-2'*H*,4'*H*-dispiro[azetidine-3,1'-naphthalene-3',1''-cyclohexan]-1-yl)(phenyl)methanone: 4t

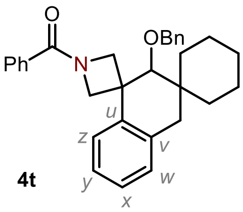

4t

Synthesised according to **General Procedure E** from: **2j** (69.5 mg, 0.200 mmol, 1.00 equiv), $HBF_4 \cdot OEt_2$ (28.6 µL, 1.05 equiv), Et_3N (0.112 mL, 4.00 equiv) and benzoyl chloride (57.1 µL, 2.00 equiv). Purified by flash column chromatography (SiO_2; 75:25 hexane:EtOAc) to afford **4t** (74.6 mg, 0.165 mmol, 83%) as a white solid.

TLC: R_f = 0.27 (75:25 hexane:EtOAc).

NMR Spectroscopy (*see spectra*): *Doubling of signals due to presence of rotamers*

> **^{1}H NMR** (500 MHz, $CDCl_3$): δ_H 7.76 – 7.70 (m, 1H, ArC^z*H*), 7.68 – 7.58 (m, 2H, ArC*H*), 7.52 – 7.30 (m, 9H, ArC^y*H*, ArC*H*), 7.25 – 7.19 (m, 1H, ArC^x*H*), 7.10 (d, *J* = 7.5 Hz, 1H, ArC^w*H*), 5.00 – 4.71 (m, 3H, OC*H*$_2$Ph, NC*H*$_2$), 4.51 (d, *J* = 10.2 Hz, 0.6H, NC*H*$_2$), 4.43 – 4.23 (m, 1.4H, NC*H*$_2$), 4.09 (d, *J* = 9.8 Hz, 0.6H, NC*H*$_2$), 4.07 (d, *J* = 9.8 Hz, 0.4H, NC*H*$_2$), 3.60 (s, 0.6H, C*H*OCH$_2$Ph), 3.54 (s, 0.4H, C*H*OCH$_2$Ph), 3.14 (d, *J* = 16.6 Hz, 0.6H, C*H*$_2$Ar), 3.12 (d, *J* = 16.6 Hz, 0.4H, C*H*$_2$Ar), 2.57 (d, *J* = 16.6, 0.4H, C*H*$_2$Ar), 2.54 (d, *J* = 16.6, 0.6H, C*H*$_2$Ar), 1.86 – 1.11 (m, 10H, cy-C*H*$_2$) ppm;

> **^{13}C NMR** (126 MHz, $CDCl_3$): δ_C 170.7 and 170.4 (*C*=O), 140.7 and 140.6 (ArC^u), 138.5 and 138.4 (Ar*C*), 134.5 and 134.4 (ArC^v), 133.5 (Ar*C*), 131.1 (Ar*C*H), 129.0 (Ar*C*H), 128.5 (Ar*C*H), 128.5 (Ar*C*H), 128.1 (Ar*C*H), 127.8 and 127.7 (Ar*C*H), 127.6 (Ar*C*H), 127.3 (Ar*C*H), 127.2 (Ar*C*H), 126.2 and 126.0 (Ar*C*H), 88.0 and 87.2 (*C*HOCH$_2$Ph), 77.0 and 77.0 (O*C*H$_2$Ph), 66.4 (N*C*H$_2$), 64.3 (N*C*H$_2$), 61.9 (N*C*H$_2$), 60.0 (N*C*H$_2$), 41.8 and 41.6 (*spiro-C*), 39.5 ((*C*)-cy), 36.9 and 36.4 (*C*H$_2$Ar), 34.1 and 33.7 (cy-*C*H$_2$), 30.2 and 28.7 (cy-*C*H$_2$), 26.3 (cy-*C*H$_2$), 21.6 and 21.5 (cy-*C*H$_2$) ppm.

HRMS (ESI^+): m/z calc'd for $C_{31}H_{34}NO_2$ $[M+H]^+$: 452.2584 found: 452.2580.

IR (film): ν_{max} 2931, 2856, 1628 (C=O), 1450, 1404, 1067 cm^{-1}.

2'-(Benzyloxy)-1-(pyrimidin-2-yl)-2'*H*,4'*H*-dispiro[azetidine-3,1'-naphthalene-3',1''-cyclohexane]: 4u

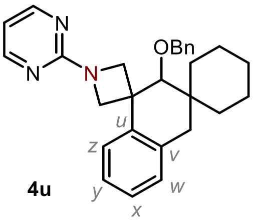

4u

Synthesised according to **General Procedure F** from: **2j** (69.5 mg, 0.200 mmol, 1.00 equiv), $HBF_4 \cdot OEt_2$ (28.6 µL, 1.05 equiv), Et_3N (0.112 mL, 4.00 equiv) and 2-chloropyrimidine (68.7 mg, 3.00 equiv). Purified by flash column chromatography (SiO_2; 80:20 hexane:EtOAc) to afford **4u** (68.7 mg, 0.161 mmol, 81%) as a white solid.

TLC: R_f = 0.21 (4:1 hexane:EtOAc).

NMR Spectroscopy (*see spectra*):

^{1}H NMR (500 MHz, $CDCl_3$): δ_H 8.34 (d, *J* = 4.8 Hz, 2H, ArC*H*), 7.80 (dd, *J* = 7.9, 1.3 Hz, 1H, ArC^{z}*H*), 7.35 – 7.25 (m, 1H, ArC^{y}*H*), 7.23 – 7.16 (m, 6H, ArC*H*, ArC^{x}*H*), 7.08 (dd, *J* = 7.6, 1.3 Hz, 1H, ArC^{w}*H*), 6.57 (t, *J* = 4.8 Hz, 1H, ArC*H*), 4.88 (d, *J* = 11.4 Hz, 1H, OCH_2Ph), 4.84 – 4.74 (m, 2H, OCH_2Ph, NCH_2), 4.39 (d, *J* = 8.6 Hz, 1H, NCH_2), 4.16 (d, *J* = 8.6 Hz, 1H, NCH_2), 4.05 (d, *J* = 8.6 Hz, 1H, NCH_2), 3.63 (s, 1H, C*H*OCH_2Ph), 3.14 (d, *J* = 16.5 Hz, 1H, CH_2Ar), 2.58 (d, *J* = 16.5 Hz, 1H, CH_2Ar), 1.68 – 1.24 (m, 10H, cy-CH_2) ppm;

^{13}C NMR (126 MHz, $CDCl_3$): δ_C 162.7 (Ar*C*), 158.1 (Ar*C*H), 141.2 (Ar*C*^{u}), 138.7 (Ar*C*), 134.5 (Ar*C*^{v}), 128.9 (Ar*C*H), 128.3 (Ar*C*H), 127.4 (Ar*C*H), 127.2 (Ar*C*H), 127.0 (Ar*C*H), 126.9 (Ar*C*H), 126.6 (Ar*C*H), 110.4 (Ar*C*H), 87.8 (*C*HOCH_2Ph), 76.8 (OCH_2Ph), 63.4 (NCH_2), 61.3 (NCH_2), 42.0 (*spiro*-*C*), 39.4 ((*C*)-cy), 36.8 (CH_2Ar), 33.8 (cy-CH_2), 30.1 (cy-CH_2), 26.4 (cy-CH_2), 21.7 (cy-CH_2) ppm.

HRMS (ESI$^+$): m/z calc'd for $C_{28}H_{32}N_3O$ $[M+H]^+$: 426.2540 found: 426.2533

IR (film): ν_{max} 2932, 2857, 1579, 1465, 1377, 1068 cm^{-1}.

2'-(Benzyloxy)-1-(2-chloropyrimidin-4-yl)-2'*H*,4'*H*-dispiro[azetidine-3,1'-naphthalene-3',1''-cyclohexane]: 4v

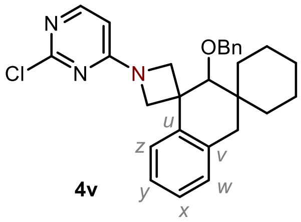

4v

Synthesised according to **General Procedure F** from: **2j** (69.5 mg, 0.200 mmol, 1.00 equiv), $HBF_4 \cdot OEt_2$ (28.6 µL, 1.05 equiv), Et_3N (0.112 mL, 4.00 equiv) and 2,4-dichloropyrimidine (89.4 mg, 3.00 equiv). Purified by flash column chromatography (SiO_2; 75:25 hexane:EtOAc) to afford **4v** (55.3 mg, 0.120 mmol, 60%) as a white solid.[**A**]

Notes: (**A**) 15% of 4-chloropyrimidin-2-yl regioisomer detected by ^{1}H NMR spectroscopy.

TLC: R_f = 0.25 (75:25 hexane:EtOAc).

NMR Spectroscopy (*see spectra*): *Doubling of signals due to presence of rotamers*

^{1}H NMR (400 MHz, $CDCl_3$): δ_H 7.98 (s, 1H, ArC*H*), 7.74 – 7.48 (m, 1H, ArC^{z}*H*), 7.33 – 7.17 (m, 7H, ArC*H*, ArC^{y}*H*, ArC^{x}*H*), 7.09 (dd, *J* = 7.6, 1.4 Hz, 1H, ArC^{w}*H*), 6.11 – 5.91 (m, 1H, ArC*H*), 5.01 – 4.80 (m, 1.5H, OCH_2Ph, NCH_2), 4.69 (d, *J* = 11.6 Hz, 1H, OCH_2Ph), 4.56 (br s, 0.5H, NCH_2), 4.39 – 3.79 (m, 3H, NCH_2), 3.57 (s, 1H, C*H*OCH_2Ph), 3.16 (d, *J* = 16.6 Hz, 1H, CH_2Ar), 2.56 (d, *J* = 16.6 Hz, 1H, CH_2Ar), 1.77 – 1.21 (m, 10H, cy-CH_2) ppm;

^{13}C NMR (126 MHz, $CDCl_3$): δ_C 163.3 (Ar*C*), 161.0 (Ar*C*Cl), 156.4 (Ar*C*H), 140.3 (Ar*C*^{u}), 138.3 (Ar*C*), 134.5 (Ar*C*^{v}), 129.1 (Ar*C*H), 128.5 (Ar*C*H), 127.9 (Ar*C*H), 127.4 (Ar*C*H), 127.3 (Ar*C*H), 127.1 (Ar*C*H), 126.2

(Ar*C*H), 100.9 (Ar*C*H), 87.6 (*C*HOCH$_2$Ph), 77.4 (O*C*H$_2$Ph), 62.9 (N*C*H$_2$), 61.1 (N*C*H$_2$), 42.5 (*spiro-C*), 39.6 ((*C*)-cy), 36.9 (*C*H$_2$Ar), 34.2 (cy-*C*H$_2$), 28.8 (cy-*C*H$_2$), 26.3 (cy-*C*H$_2$), 21.6 (cy-*C*H$_2$) ppm.

HRMS (ESI$^+$): m/z calc'd for $C_{28}H_{31}NOCl$ [M+H]$^+$: 460.2150 found: 460.2144.

IR (film): ν_{max} 2932, 2857, 1585, 1498, 1466, 1355 cm^{-1}.

2'-(Benzyloxy)-1-(5-fluoropyrimidin-2-yl)-2'*H*,4'*H*-dispiro[azetidine-3,1'-naphthalene-3',1''-cyclohexane]: 4w

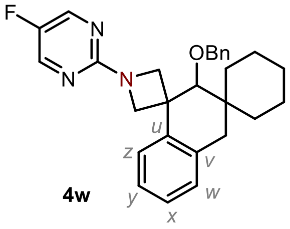

4w

Synthesised according to **General Procedure F** from: **2j** (69.5 mg, 0.200 mmol, 1.00 equiv), $HBF_4{\cdot}OEt_2$ (28.6 µL, 1.05 equiv), Et_3N (0.112 mL, 4.00 equiv) and 2-chloro-5-fluoropyrimidine (55.3 µL, 3.00 equiv). Purified by flash column chromatography (99:1 toluene:Et_2O) to afford **4w** (54.7 mg, 0.123 mmol, 62%) as a white solid.

TLC: R_f = 0.24 (99:1 toluene:Et_2O).

NMR Spectroscopy (*see spectra*):

^{1}H NMR (400 MHz, $CDCl_3$): δ_H 8.20 (d, *J* = 0.8 Hz, 2H, ArC*H*), 7.79 (dd, *J* = 7.9, 1.3 Hz, 1H, ArC^z*H*), 7.33 – 7.26 (m, 1H, ArC^y*H*), 7.23 – 7.13 (m, 6H, ArC^x*H*, ArC*H*), 7.08 (dd, *J* = 7.6, 1.3 Hz, 1H, ArC^w*H*), 4.89 (d, *J* = 11.4 Hz, 1H, OC*H*$_2$Ph), 4.81 – 4.70 (m, 2H, OC*H*$_2$Ph, NC*H*$_2$), 4.33 (d, *J* = 8.5 Hz, 1H, NC*H*$_2$), 4.13 (d, *J* = 8.5 Hz, 1H, NC*H*$_2$), 3.99 (d, *J* = 8.5 Hz, 1H, NC*H*$_2$), 3.58 (s, 1H, C*H*OCH$_2$Ph), 3.13 (d, *J* = 16.6 Hz, 1H, C*H*$_2$Ar), 2.56 (d, *J* = 16.6 Hz, 1H, C*H*$_2$Ar), 1.73 – 1.23 (m, 10H, cy-C*H*$_2$) ppm;

^{13}C NMR (126 MHz, $CDCl_3$): δ_C 160.2 (Ar*C*), 152.4 (d, *J* = 248.0 Hz, Ar*C*F), 145.6 (d, *J* = 21.8 Hz, Ar*C*H), 141.1 (Ar*C*^{u}), 138.6 (Ar*C*), 134.5 (Ar*C*^{v}), 128.9 (Ar*C*H), 128.3 (Ar*C*H), 127.4 (Ar*C*H), 127.2 (Ar*C*H), 127.0 (Ar*C*H), 126.9 (Ar*C*H), 126.6 (Ar*C*H), 87.8 (*C*HOCH$_2$Ph), 76.8 (O*C*H$_2$Ph), 63.8 (N*C*H$_2$), 61.8 (N*C*H$_2$), 42.0 (*spiro-C*), 39.4 ((*C*)-cy), 36.9 (*C*H$_2$Ar), 33.9 (cy-*C*H$_2$), 29.7 (cy-*C*H$_2$), 26.4 (cy-*C*H$_2$), 21.7 (cy-*C*H$_2$) ppm;

^{19}F NMR (376 MHz, $CDCl_3$): δ_F –156.1 ppm.

HRMS (ESI$^+$): m/z calc'd for $C_{28}H_{31}NOF$ [M+H]$^+$: 444.2446 found: 444.2438.

IR (film): ν_{max} 2931, 2857, 1556, 1498, 1465, 1386, 1069 cm^{-1}.

2'-(Benzyloxy)-1-(6-(trifluoromethyl)pyridin-2-yl)-2'*H*,4'*H*-dispiro[azetidine-3,1'-naphthalene-3',1''-cyclohexane]: 4x

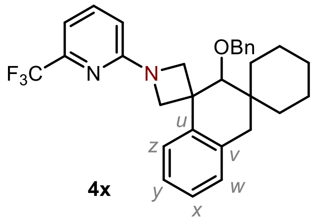

4x

Synthesised according to **General Procedure F** from: **2j** (69.5 mg, 0.200 mmol, 1.00 equiv), $HBF_4{\cdot}OEt_2$ (28.6 µL, 1.05 equiv), Et_3N (0.112 mL, 4.00 equiv) and 2-fluoro-6-(trifluoromethyl)pyridine (72.3 µL, 3.00 equiv). Purified by flash column chromatography (19:1 hexane:EtOAc) to afford **4x** (49.4 mg, 0.100 mmol, 50%) as a white solid.

TLC: R_f = 0.25 (19:1 hexane:EtOAc).

NMR Spectroscopy (*see spectra*):

^{1}**H NMR** (500 MHz, $CDCl_3$): δ_H 7.82 (dd, *J* = 7.4, 1.3 Hz, 1H, ArC^zH), 7.53 (dd, *J* = 7.3, 8.4 Hz, 1H, ArC*H*), 7.29 (app-t, *J* = 7.4 Hz, 1H, ArC^yH), 7.22 – 7.14 (m, 6H, ArC*H*, ArC^xH), 7.09 (d, *J* = 7.6 Hz, 1H, ArC^wH), 6.97 (d, *J* = 7.3 Hz, 1H, ArC*H*), 6.41 (d, *J* = 8.4 Hz, 1H, ArC*H*), 4.87 (d, *J* = 11.3 Hz, 1H, OCH_2Ph), 4.73 (d, *J* = 11.3 Hz, 1H, OCH_2Ph), 4.61 (d, *J* = 8.0 Hz, 1H, NCH_2), 4.29 (d, *J* = 8.3 Hz, 1H, NCH_2), 4.10 (d, *J* = 8.3 Hz, 1H, NCH_2), 3.93 (d, *J* = 8.0 Hz, 1H, NCH_2), 3.57 (s, 1H, C*H*OCH_2Ph), 3.14 (d, *J* = 16.5 Hz, 1H, CH_2Ar), 2.58 (d, *J* = 16.5 Hz, 1H, CH_2Ar), 1.68 – 1.24 (m, 10H, cy-CH_2) ppm;

^{13}**C NMR** (126 MHz, $CDCl_3$): δ_C 160.3 (Ar*C*), 146.9 (q, *J* = 33.8 Hz, Ar*C*), 141.2 (ArC^u), 138.5 (Ar*C*), 137.7 (Ar*C*H), 134.4 (ArC^v), 128.9 (Ar*C*H), 128.3 (Ar*C*H), 127.5 (Ar*C*H), 127.2 (Ar*C*H), 127.2 (Ar*C*H), 126.9 (Ar*C*H), 126.8 (Ar*C*H), 121.9 (q, *J* = 274.1 Hz, CF_3), 109.1 (Ar*C*H), 108.8 (q, *J* = 3.3 Hz, Ar*C*H), 87.9 (*C*HOCH_2Ph), 76.8 (OCH_2Ph), 63.7 (NCH_2), 61.9 (NCH_2), 42.4 (*spiro*-*C*), 39.5 ((*C*)-cy), 36.7 (CH_2Ar), 33.9 (cy-CH_2), 29.7 (cy-CH_2), 26.4 (cy-CH_2), 21.7 (cy-CH_2) ppm;

^{19}**F NMR** (376 MHz, $CDCl_3$): δ_F –68.4 ppm.

HRMS (ESI^+): m/z calc'd for $C_{30}H_{32}N_2OF_3$ $[M+H]^+$: 493.2461 found:493.2454.

IR (film): ν_{max} 2933, 2859, 1604, 1467, 1330, 1282, 1195, 1136, 1105 cm^{-1}.

2'-(Benzyloxy)-1-(2,4-dinitrophenyl)-2'*H*,4'*H*-dispiro[azetidine-3,1'-naphthalene-3',1''-cyclohexane]: 4y

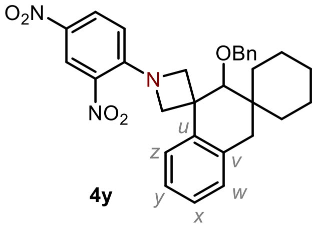

4y

Synthesised according to **General Procedure F** from: **2j** (69.5 mg, 0.200 mmol, 1.00 equiv), $HBF_4{\cdot}OEt_2$ (28.6 µL, 1.05 equiv), Et_3N (0.112 mL, 4.00 equiv) and 1-fluoro-2,4-dinitrobenzene (75.9 µL, 3.00 equiv).

Purified by flash column chromatography (6.1:1 hexane:EtOAc) to afford **4y** (70.1 mg, 0.136 mmol, 68%) as a yellow solid.

TLC: R_f = 0.30 (6.1:1 hexane:EtOAc).

NMR Spectroscopy (see spectra):

^{1}H NMR (500 MHz, $CDCl_3$): δ_H 8.75 (d, *J* = 2.6 Hz, 1H, ArC*H*), 8.11 (dd, *J* = 9.4, 2.6 Hz, 1H, ArC*H*), 7.66 (dd, *J* = 7.7, 1.4 Hz, 1H, ArC^{z}*H*), 7.31 (app-td, *J* = 7.7, 1.4 Hz, 1H, ArC^{y}*H*), 7.25 – 7.06 (m, 7H, ArC*H*, ArC^{x}*H*, ArC^{w}*H*), 6.49 (d, *J* = 9.3 Hz, 1H, ArC*H*), 4.97 (d, *J* = 11.6 Hz, 1H, OCH_2Ph), 4.61 (d, *J* = 9.2 Hz, 1H, NCH_2), 4.55 (d, *J* = 11.6 Hz, 1H, OCH_2Ph), 4.28 (d, *J* = 9.7 Hz, 1H, NCH_2), 3.99 (d, *J* = 9.2 Hz, 1H, NCH_2), 3.95 (d, *J* = 9.7 Hz, 1H, NCH_2), 3.53 (s, 1H, C*H*OCH_2Ph), 3.18 (d, *J* = 16.7 Hz, 1H, CH_2Ar), 2.54 (d, *J* = 16.7 Hz, 1H, CH_2Ar), 1.89 – 1.16 (m, 10H, cy-CH_2) ppm;

^{13}C NMR (126 MHz, $CDCl_3$): δ_C 147.5 (Ar*C*), 140.0 (Ar*C*^{u}), 138.1 (Ar*C*), 136.2 (Ar*C*NO_2), 134.4 (Ar*C*^{v}), 133.3 (Ar*C*NO_2), 129.1 (Ar*C*H), 128.5 (Ar*C*H), 127.9 (Ar*C*H), 127.9 (Ar*C*H), 127.6 (Ar*C*H), 127.5 (Ar*C*H), 127.2 (Ar*C*H), 126.3 (Ar*C*H), 124.1 (Ar*C*H), 115.2 (Ar*C*H), 87.7 (*C*HOCH_2Ph), 77.2 (O*C*H_2Ph), 66.7 (N*C*H_2), 64.7 (N*C*H_2), 41.8 (*spiro*-*C*), 39.7 ((*C*)-cy), 37.0 (*C*H_2Ar), 34.6 (cy-*C*H_2), 28.0 (cy-*C*H_2), 26.2 (cy-*C*H_2), 21.5 (cy-*C*H_2) ppm.

HRMS (ESI$^+$): m/z calc'd for $C_{30}H_{32}N_3O_5$ $[M+H]^+$: 414.2336 found: 414.2336.

IR (film): ν_{max} 2931, 2858, 1607, 1580, 1525, 1500, 1330, 1308, 1138 cm^{-1}.

Synthesis of 2,2,2-Trifluoro-1-(2'-methoxy-3',4'-dihydro-2'*H*-spiro[azetidine-3,1'-naphthalen]-1-yl)ethan-1-one: 4a′

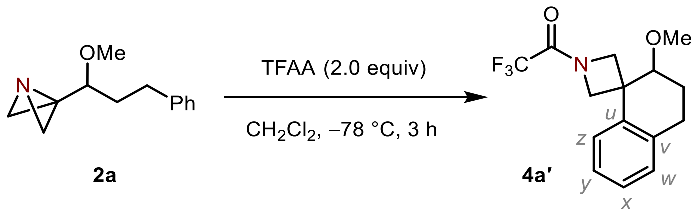

To a stirred solution of **2a** (40.7 mg, 0.200 mmol, 1.00 equiv) in anhydrous CH_2Cl_2 (2 mL) at −78 °C was added trifluoroacetic anhydride (TFAA) (55.6 µL, 2.00 equiv) dropwise. The solution was then stirred at the same temperature for 3 h. After this time, the reaction mixture was quenched with 1:1 MeOH:CH_2Cl_2 (0.5 mL) before warming to rt. The resulting solution was concentrated under reduced pressure then directly purified by flash column chromatography (SiO_2; 85:15 hexane:EtOAc) to afford **4a′** (33.5 mg, 0.112 mmol, 56%) as a colourless oil.

TLC: R_f = 0.39 (80:20 hexane:EtOAc).

NMR Spectroscopy (see spectra): *Doubling of signals due to presence of rotamers*.

^{1}H NMR (500 MHz, $CDCl_3$): δ_H 7.55 (d, *J* = 7.7 Hz, 1H, ArC^{z}*H*), 7.30 (app-t, *J* = 7.7 Hz, 1H, ArC^{y}*H*), 7.21 (app-t, *J* = 7.7 Hz, 1H, ArC^{x}*H*), 7.10 (d, *J* = 7.7 Hz, 1H, ArC^{w}*H*), 4.75 (d, *J* = 9.5 Hz, 0.5H, NCH_2), 4.52 (d, *J* = 10.5 Hz, 0.5H, NCH_2), 4.48 (d, *J* = 9.5 Hz, 0.5H, NCH_2), 4.40 (d, *J* = 9.4 Hz, 0.5H, NCH_2), 4.30 – 4.23

(m, 1H, NC*H*$_2$), 4.19 (d, *J* = 10.5 Hz, 0.5H, NC*H*$_2$), 4.06 (d, *J* = 10.5 Hz, 0.5H, NC*H*$_2$), 3.56 (app-t, *J* = 2.4 Hz, 0.5H, C*H*OCH$_3$), 3.54 (app-t, *J* = 2.4 Hz, 0.5H, C*H*OCH$_3$), 3.49 (s, 1.5H, CHOC*H*$_3$), 3.49 (s, 1.5H, CHOC*H*$_3$), 2.98 – 2.90 (m, 1H, C*H*$_2$Ar), 2.82 – 2.72 (m, 1H, C*H*$_2$Ar), 2.11 – 1.95 (m, 1H, C*H*$_2$CH$_2$Ar), 1.85 – 1.71 (m, 1H, C*H*$_2$CH$_2$Ar) ppm;

^{13}C NMR (126 MHz, CDCl$_3$): δ_C 156.7 (q, *J* = 37.3 Hz, F$_3$C(*C*=O)) and 156.6 (q, *J* = 37.3 Hz, F$_3$C(*C*=O)), 138.0 and 137.9 (Ar*C*^{u}), 135.8 (Ar*C*^{v}), 129.0 and 129.0 (Ar*C*^{w}H), 127.5 (Ar*C*^{y}H), 127.5 (Ar*C*^{x}H), 126.1 and 126.0 (Ar*C*^{z}H), 116.3 (q, *J* = 288 Hz, F$_3$*C*(C=O)) and 116.3 (q, *J* = 288 Hz, F$_3$*C*(C=O)), 80.2 and 80.1 (*C*HOCH$_3$), 62.4 and 61.3 (N*C*H$_2$), 59.5 and 58.5 (N*C*H$_2$), 57.0 and 57.0 (CHO*C*H$_3$), 42.2 (*spiro*-*C*), 26.8 and 26.6 (*C*H$_2$Ar), 22.8 and 22.5 (*C*H$_2$CH$_2$Ar) ppm;

^{19}F NMR (376 MHz, CDCl$_3$): δ_F –72.5, –72.5 ppm.

HRMS (APCI$^+$): m/z calc'd for $C_{15}H_{16}NO_2F_3$ [M+H]$^+$: 300.1206, found: 300.1193.

IR (film): ν_{max} 2946, 2830, 1698 (C=O), 1465, 1248, 12-3, 1146 cm^{-1}.

2.7. Synthesis of Friedel-Crafts intermediates

3'-(Benzyloxy)-2a1',8a'-dihydro-2'*H*,3'*H*,5'*H*-spiro[cyclohexane-1,4'-[1,2a]methanobenzo[*cd*]indole]: 3j

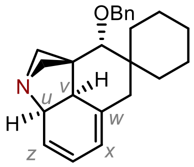

3j

Synthesised according to **General Procedure G** from: **2j** (69.5 mg, 0.200 mmol, 1.00 equiv) and HBF$_4$·OEt$_2$ (28.6 µL, 1.05 equiv). The residue was precipitated from Et$_2$O with HCl (2.0 M in Et$_2$O, 0.20 mL, 2.0 equiv) to separate from impurities before revealing the free-base upon washing with sat. aq. NaHCO$_3$ and concentrated under reduced pressure to afford **3j** (37.7 mg, 0.108 mmol, 54%) as a white solid.

NMR Spectroscopy (*see spectra*):

^{1}H NMR (500 MHz, CDCl$_3$): δ_H 7.37 – 7.28 (m, 5H, ArC*H*), 6.00 (dd, *J* = 9.8, 5.5 Hz, 1H, C=C^y*H*), 5.70 (dd, *J* = 9.8, 4.5 Hz, 1H, C=C^z*H*), 5.57 (d, *J* = 5.5 Hz, 1H, C=C^x*H*), 4.56 – 4.47 (m, 2H, OC*H*$_2$Ph), 3.88 (dd, *J* = 10.7, 4.5 Hz, 1H, C^u*H*), 3.51 (s, 1H, C*H*OCH$_2$Ph), 3.10 (d, *J* = 10.7 Hz, 1H, C^v*H*), 2.94 – 2.92 (m, 2H, NC*H*$_2$), 2.53 (dd, *J* = 9.7, 6.5 Hz, 1H, NC*H*$_2$), 2.30 (d, *J* = 12.8 Hz, 1H, C*H*$_2$(C=C)), 2.23 – 2.14 (m, 2H, NC*H*$_2$, C*H*$_2$(C=C)), 1.59 – 1.21 (m, 9H, cy-C*H*$_2$), 1.09 – 1.05 (m, 1H, cy-C*H*$_2$) ppm;

^{13}C NMR (126 MHz, CDCl$_3$): δ_C 139.0 (Ar*C*), 138.1 (C=*C*^{w}), 128.5 (Ar*C*H), 128.4 (C=*C*^{y}*H*), 127.8 (Ar*C*H), 127.6 (Ar*C*H), 122.6 (C=*C*^{z}*H*), 116.6 (C=*C*^{x}*H*), 81.2 (*C*HOCH$_2$Ph), 74.1 (O*C*H$_2$Ph), 65.3 (*spiro*-*C*), 62.6 (*C*^{u}H), 62.1 (N*C*H$_2$), 60.1 (N*C*H$_2$), 41.8 (cy-(*C*)), 40.5 (*C*^{v}H), 40.3 (C=C*C*H$_2$,), 35.6 (cy-*C*H$_2$), 32.6 (cy-*C*H$_2$), 26.4 (cy-*C*H$_2$), 21.9 (cy-*C*H$_2$), 21.6 (cy-*C*H$_2$) ppm.

HRMS (ESI$^+$): m/z calc'd for $C_{24}H_{30}NO$ [M+H]$^+$: 348.2322 found: 348.2320.

IR (film): v_{max} 2923, 2855, 1453, 1090, 1065 cm^{-1}.

3'-(Benzyloxy)-7'-methoxy-3'-methyl-2',3'-dihydro-1'*H*- spiro[azetidine-3,4'-phenanthren]-1-ium tetrafluoroborate: 8m

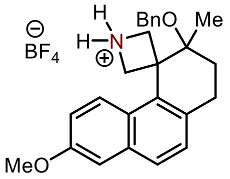

8m

Synthesised according to **General Procedure G** from: **2m** (74.7 mg, 0.200 mmol, 1.00 equiv) and $HBF_4{\cdot}OEt_2$ (28.6 µL, 1.05 equiv). The residue was precipitated from Et_2O with $HBF_4{\cdot}OEt_2$ (54.5 µL, 2.00 equiv) to afford **8m** (34.3 mg, 0.744 mmol, 37%) as a white solid.

NMR Spectroscopy (*see spectra*):

^{1}H NMR (500 MHz, $CDCl_3$): δ_H 8.26 (d, *J* = 9.4 Hz, 1H, ArC*H*), 7.63 (d, *J* = 8.4 Hz, 1H, ArC*H*), 7.53 – 7.45 (m, 3H, ArC*H*), 7.40 – 7.36 (m, 2H, ArC*H*), 7.33 – 7.28 (m, 1H, ArC*H*), 7.20 (d, *J* = 2.8 Hz, 1H, ArC*H*), 7.14 (d, *J* = 8.4 Hz, 1H, ArC*H*), 5.54 (d, *J* = 11.5 Hz, 1H, NCH_2), 4.85 – 4.70 (m, 3H, OCH_2Ph, NCH_2), 4.64 (d, *J* = 10.7 Hz, 1H, NCH_2), 4.60 (d, *J* = 11.5 Hz, 1H, NCH_2), 3.93 (s, 3H, ArOCH_3), 3.09 – 2.86 (m, 2H, ArCH_2), 2.28 – 2.18 (m, 1H, ArCH$_2$CH_2), 1.97 – 1.79 (m, 1H, ArCH$_2$CH_2), 1.64 (s, 2H, N-*H*), 1.34 (s, 3H, C(OCH$_2$Ph)CH_3) ppm;

^{13}C NMR (126 MHz, $CDCl_3$): δ_C 157.0 (ArOCH$_3$), 138.8 (Ar*C*), 135.3 (Ar*C*), 133.1 (Ar*C*), 128.9 (Ar*C*H), 128.6 (Ar*C*H), 128.2 (Ar*C*H), 128.2 (Ar*C*H), 128.0 (Ar*C*), 127.5 (Ar*C*H), 126.7 (Ar*C*), 124.3 (Ar*C*H), 119.0 (Ar*C*H), 109.2 (Ar*C*H), 79.0 ((*C*)OCH$_2$Ph), 65.0 (O*C*H$_2$Ph), 56.2 (N*C*H$_2$), 55.5 (ArO*C*H$_3$), 54.3 (N*C*H$_2$), 50.1 (*spiro-C*), 28.9 (Ar*C*H$_2$), 27.7 (ArCH$_2$*C*H$_2$), 17.7 (C(OCH$_2$Ph)*C*H$_3$) ppm;

^{19}F NMR (376 MHz, $CDCl_3$): δ_F –68.4 ppm.

HRMS (ESI$^+$): m/z calc'd for $C_{25}H_{28}NO_2$ [M–BF$_4$]$^+$: 374.2115 found: 374.2111.

IR (film): v_{max} 3615 (N-H), 3224 (N-H), 2939, 1609, 1107, 1038 cm^{-1}.

4-(Benzyloxy)-1,1- dimethyloctahydro-2,3a-methanoisoindol-2-ium chloride: 3p

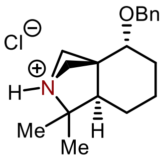

3p

Synthesised according to **General Procedure G** from: **2p** (54.3 mg, 0.200 mmol, 1.00 equiv) and $HBF_4{\cdot}OEt_2$ (28.6 µL, 1.05 equiv). The residue was precipitated from Et_2O with HCl (2.0 M in Et_2O, 0.20 mL, 2.0 equiv) to afford **3p** (27.7 mg, 0.900 mmol, 45%) as a white solid.

NMR Spectroscopy (*see spectra*):

^{1}H NMR (500 MHz, $CDCl_3$): δ_H 11.98 (s, 1H, N-*H*), 7.40 – 7.24 (m, 5H, ArC*H*), 4.60 (d, *J* = 12.0 Hz, 1H, OC*H*$_2$Ph), 4.33 (d, *J* = 12.0 Hz, 1H, OC*H*$_2$Ph), 3.80 (app-t, *J* = 2.7 Hz, 1H, C*H*OCH$_2$Ph), 3.66 – 3.56 (m, 2H, NC*H*$_2$), 3.38 – 3.31 (m, 1H, NC*H*$_2$), 3.29 – 3.21 (m, 1H, NC*H*$_2$), 2.35 (dd, *J* = 12.8, 4.6 Hz, 1H, C*H*C(CH$_3$)$_2$), 2.04 (d, *J* = 13.0 Hz, 1H, CH(OCH$_2$Ph)C*H*$_2$), 1.88 – 1.61 (m, 3H, CHC(CH$_3$)$_2$C*H*$_2$, CH(OCH$_2$Ph)CH$_2$C*H*$_2$), 1.60 (s, 3H, C(C*H*$_3$)$_2$), 1.49 (s, 3H, C(C*H*$_3$)$_2$), 1.30 – 1.11 (m, 2H, CH(OCH$_2$Ph)C*H*$_2$, CHC(CH$_3$)$_2$C*H*$_2$) ppm;

^{13}C NMR (126 MHz, $CDCl_3$): δ_C 137.9 (Ar*C*), 128.7 (Ar*C*H), 128.3 (Ar*C*H), 127.8 (Ar*C*H), 71.2 (*C*HOCH$_2$Ph), 70.8 (O*C*H$_2$Ph), 68.4 (*C*(CH$_3$)$_2$), 62.3 (N*C*H$_2$), 60.1 (N*C*H$_2$), 54.7 (*spiro*-*C*), 44.8 (*C*HC(CH$_3$)$_2$), 26.2 (C(*C*H$_3$)$_2$), 25.9 (CH(OCH$_2$Ph)*C*H$_2$), 24.9 (CHC(CH$_3$)$_2$*C*H$_2$), 20.8 (C(*C*H$_3$)$_2$), 19.3 (CH(OCH$_2$Ph)CH$_2$*C*H$_2$) ppm.

HRMS (APCI$^+$): m/z calc'd for $C_{18}H_{26}NO$ [M–Cl]$^+$: 272.2009, found: 272.2011.

IR (film): ν_{max} 2931, 2862, 1469, 1454, 1092, 1059 cm^{-1}.

2.8. Synthesis of Diels-Alder adducts

3-(Benzyloxy)-9-phenyl-2,3,3a1,5a-tetrahydro-1*H*,4*H*,6*H*,8*H*-6,11a-etheno-3a,5-methanopyrrolo[2,3,4-*de*][1,2,4]triazolo[1,2-*a*]cinnoline-8,10(9*H*)-dione: 5b

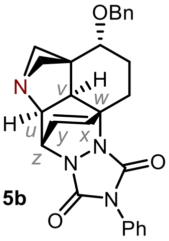

5b

Synthesised according to **General Procedure H** from: **2b** (55.9 mg, 0.200 mmol, 1.00 equiv), $HBF_4 \cdot OEt_2$ (28.6 µL, 1.05 equiv) and PTAD (38.5 mg, 1.10 eq.). The residue was purified by flash column chromatography on Et_3N deactivated silica gel (Et_3N deact. SiO_2; 98:2 CH_2Cl_2:MeOH) to afford **5b** (41.8 mg, 0.092 mmol, 46%) as a white solid.

NMR Spectroscopy (*see spectra*):

^{1}H NMR (400 MHz, $CDCl_3$): δ_H 7.45 – 7.26 (m, 10H, ArC*H*), 6.51 – 6.41 (m, 2H, *H*C^y=C^x*H*), 5.38 (dd, *J* = 5.7, 4.2 1H, C^z*H*), 4.57 (d, *J* = 12.0 Hz, 1H, OC*H*$_2$Ph), 4.43 (d, *J* = 12.0 Hz, 1H, OC*H*$_2$Ph), 3.85 (dd, *J* = 8.0, 4.2 Hz, 1H, C^u*H*), 3.75 (app-t, *J* = 2.6 Hz, 1H, CHOCH$_2$Ph), 3.64 (dd, *J* = 10.7, 7.4 Hz, 1H, NC*H*$_2$), 3.19 (d, *J* = 5.8 Hz, 1H, NC*H*$_2$), 3.03 (d, *J* = 8.0 Hz, 1H, C^v*H*), 2.69 – 2.59 (m, 2H, CH(OCH$_2$Ph)CH$_2$C*H*$_2$), 2.51 (d, *J* = 7.4 Hz, 1H, NC*H*$_2$), 2.44 (dd, *J* = 10.7, 5.8 Hz, 1H, NC*H*$_2$), 2.30 – 2.18 (m, 1H, CH(OCH$_2$Ph)C*H*$_2$), 1.51 – 1.35 (m, 1H, CH(OCH$_2$Ph)C*H*$_2$) ppm;

^{13}C NMR (101 MHz, $CDCl_3$): δ_C 156.8 (*C*=O), 156.0 (*C*=O), 138.4 (Ar*C*), 136.3 (C=*C*^{x}H), 131.4 (Ar*C*), 129.2 (Ar*C*H), 128.6 (Ar*C*H), 128.4 (Ar*C*H), 128.1 (C=*C*^{y}H), 127.9 (Ar*C*H), 127.4 (Ar*C*H), 125.8 (Ar*C*H), 71.5 (*C*HOCH$_2$Ph), 71.1 (O*C*H$_2$Ph), 70.3 (N*C*H$_2$), 62.3 (*C*^{u}H), 61.4 (*C*^{w}), 60.5 (*spiro*-*C*), 56.8 (*C*^{z}H), 54.6 (N*C*H$_2$),

43.2 (C^{v}H), 27.0 (CH(OCH$_2$Ph)CH$_2$*C*H$_2$), 25.4 (CH(OCH$_2$Ph)*C*H$_2$) ppm.

HRMS (ESI$^+$): m/z calc'd for $C_{27}H_{27}N_4O_3$ [M+H]$^+$: 455.207767, found: 455.207970.

IR (film): ν_{max} 3029, 2942, 2864, 1712 (C=O), 1500, 1402, 1071 cm^{-1}.

9-Phenyl-3-((triethylsilyl)oxy)-2,3,3a1,5a-tetrahydro-1H,4H,6H,8H-6,11a-etheno-3a,5-methanopyrrolo[2,3,4-de][1,2,4]triazolo[1,2-a]cinnoline-8,10(9H)-dione: 5d

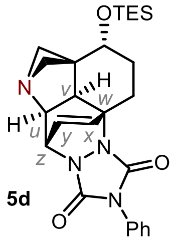

5d

Synthesised according to **General Procedure H** from: **2d** (60.7 mg, 0.200 mmol, 1.00 equiv), HBF$_4$·OEt$_2$ (28.6 µL, 1.05 equiv) and PTAD (38.5 mg, 1.10 eq.). The residue was precipitated from Et$_2$O with HCl (2.0 M in Et$_2$O, 0.20 mL, 2.0 equiv) to separate from impurities before revealing the free-base upon washing with sat. aq. NaHCO$_3$ and concentrated under reduced pressure to afford **5d** (46.1 mg, 0.096 mmol, 48%) as a white solid.

NMR Spectroscopy (*see spectra*):

^{1}H NMR (500 MHz, CDCl$_3$): δ_H 7.45 – 7.38 (m, 4H, ArC*H*), 7.37 – 7.33 (m, 1H, ArC*H*), 6.51 – 6.41 (m, 2H, *H*C^{y}=C^{x}*H*), 5.36 (ddd, *J* = 5.8, 4.3, 1.6 Hz, 1H, C^{z}*H*), 4.04 (app-t, *J* = 2.7 Hz, 1H, CHOSi), 3.81 (dd, *J* = 8.1, 4.3 Hz, 1H, C^{u}*H*), 3.60 (dd, *J* = 10.7, 7.5 Hz, 1H, NC*H*$_2$), 3.08 (d, *J* = 5.8 Hz, 1H, NC*H*$_2$), 3.01 (d, *J* = 8.1 Hz, 1H, C^{v}*H*), 2.71 – 2.56 (m, 2H, CH(OSi)CH$_2$C*H*$_2$), 2.44 (d, *J* = 7.5 Hz, 1H, NC*H*$_2$), 2.35 (dd, *J* = 10.7, 5.8 1H, NC*H*$_2$), 1.96 – 1.90 (m, 1H, CH(OSi)C*H*$_2$), 1.54 – 1.46 (m, 1H, CH(OSi)C*H*$_2$), 0.96 (t, *J* = 7.9 Hz, 9H, Si(CH$_2$C*H*$_3$)$_3$)), 0.58 (q, *J* = 7.9, 6H, Si(C*H*$_2$CH$_3$)$_3$) ppm;

^{13}C NMR (126 MHz, CDCl$_3$): δ_C 156.9 (*C*=O), 156.0 (*C*=O), 136.4 (C=C^{x}H), 131.4 (Ar*C*), 129.2 (Ar*C*H), 128.4 (Ar*C*H), 128.1 (C=C^{y}H), 125.8 (Ar*C*H), 70.2 (N*C*H$_2$), 65.2 (*C*HOSi), 62.4 (C^{u}H), 62.2 (C^{w}), 61.7 (*spiro*-*C*), 56.9 (C^{z}H), 54.3 (N*C*H$_2$), 42.7 (C^{v}H), 29.4 (CH(OSi)*C*H$_2$), 26.6 (CH(OSi)CH$_2$*C*H$_2$), 7.0 (Si(CH$_2$*C*H$_3$)$_3$), 4.9 (Si(*C*H$_2$CH$_3$)$_3$) ppm.

HRMS (ESI$^+$): m/z calc'd for $C_{26}H_{35}N_4O_3Si$ [M+H]$^+$: 479.2473, found: 479.2468.

IR (film): ν_{max} 2952, 2876, 1715 (C=O), 1501, 1402, 1237, 1072 cm^{-1}.

3-(Benzyloxy)-12-methyl-9-phenyl-2,3,3a1,5a-tetrahydro-1H,4H,6H,8H-6,11a-etheno-3a,5-methanopyrrolo[2,3,4-de][1,2,4]triazolo[1,2-a]cinnoline-8,10(9H)-dione: 5f

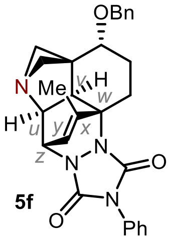

5f

Synthesised according to **General Procedure H** from: **2f** (58.7 mg, 0.200 mmol, 1.00 equiv), $HBF_4{\cdot}OEt_2$ (28.6 µL, 1.05 equiv) and PTAD (38.5 mg, 1.10 eq.). The residue was precipitated from Et_2O with HCl (2.0 M in Et_2O, 0.20 mL, 2.0 equiv) to separate from impurities before revealing the free-base upon washing with sat. aq. $NaHCO_3$ and concentrated under reduced pressure to afford **5f** (56.4 mg, 0.120 mmol, 60%) as a white solid.

NMR Spectroscopy (*see spectra*):

^{1}H NMR (400 MHz, $CDCl_3$): δ_H 7.46 – 7.29 (m, 10H, ArC*H*), 6.03 (d, *J* = 6.0 Hz, 1H, *H*C^y=C^x), 5.32 (dd, *J* = 6.0, 4.5 Hz, 1H, C^z*H*), 4.57 (d, *J* = 12.0 Hz, 1H, OCH_2Ph), 4.44 (d, *J* = 12.0 Hz, 1H, OCH_2Ph), 3.85 – 3.70 (m, 2H, C^u*H*, CHOCH_2Ph), 3.39 (dd, *J* = 10.6, 7.3 Hz, 1H, NCH_2), 3.18 (d, *J* = 6.0 Hz, 1H, NCH_2), 3.02 (d, *J* = 8.0 Hz, 1H, C^v*H*), 2.84 (app-dt, *J* = 13.7, 3.3 Hz, 1H, CH(OCH_2Ph)CH_2CH_2), 2.62 – 2.49 (m, 2H, NCH_2, CH(OCH_2Ph)CH_2CH_2), 2.44 (dd, *J* = 10.6, 6.0 Hz, 1H, NCH_2), 2.29 – 2.16 (m, 1H, CH(OCH_2Ph)CH_2), 2.01 (s, 3H, C^y=C^{x}CH_3), 1.52 – 1.39 (m, 1H, CH(OCH_2Ph)CH_2) ppm;

^{13}C NMR (126 MHz, $CDCl_3$): δ_C 157.3 (*C*=O), 156.5 (*C*=O), 143.4 (C=*C*^{x}), 138.5 (Ar*C*), 131.5 (Ar*C*), 129.2 (Ar*C*H), 128.6 (Ar*C*H), 128.4 (Ar*C*H), 127.9 (Ar*C*H), 127.4 (Ar*C*H), 125.6 (Ar*C*H), 121.3 (C=*C*^{y}*H*), 71.5 (O*C*H_2Ph), 71.2 (*C*HOCH_2Ph), 70.1 (N*C*H_2), 63.0 (*C*^{w}), 62.3 (*C*^{u}H), 60.4 (*spiro*-*C*), 58.8 (*C*^{z}H), 54.4 (N*C*H_2), 43.8 (*C*^{v}H), 25.9 (CH(OCH_2Ph)*C*H_2), 25.2 (CH(OCH_2Ph)CH_2*C*H_2), 18.3 (C=C^x*C*H_3) ppm.

HRMS (ESI^+): m/z calc'd for $C_{28}H_{29}N_4O_3$ $[M+H]^+$: 469.2234, found: 469.2224.

IR (film): ν_{max} 3033, 2942, 1711 (C=O), 1501, 1402, 1092, 1070 cm^{-1}.

3'-(Benzyloxy)-9'-phenyl-3a1',5a'-dihydro-1'*H*,3'*H*,4'*H*,6'*H*,8'*H*-spiro[cyclohexane-1,2'-[6,11a]etheno[3a,5]methanopyrrolo[2,3,4-*de*][1,2,4]triazolo[1,2-*a*]cinnoline]-8',10'(9'*H*)-dione: 5j

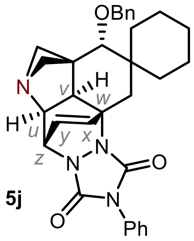

5j

Synthesised according to **General Procedure H** from: **2j** (69.5 mg, 0.200 mmol, 1.00 equiv), $HBF_4{\cdot}OEt_2$ (28.6 µL, 1.05 equiv) and PTAD (38.5 mg, 1.10 eq.). The residue was precipitated from Et_2O with HCl (2.0 M in Et_2O, 0.20 mL, 2.0 equiv) to separate from impurities before revealing the free-base upon washing with sat. aq. $NaHCO_3$ and concentrated under reduced pressure to afford **5j** (61.9 mg, 0.118 mmol, 59%) as a white solid.

NMR Spectroscopy (*see spectra*):

^{1}H NMR (400 MHz, $CDCl_3$): δ_H 7.45 – 7.29 (m, 10H, ArC*H*), 6.51 (d, *J* = 8.3 Hz, 1H, HC=C^x*H*), 6.33 (dd, *J* = 8.3, 5.9 Hz, 1H, *H*C^y=CH), 5.37 (dd, *J* = 5.9, 4.4 Hz, 1H, C^z*H*), 4.50 (d, *J* = 11.3 Hz, 1H, OC*H*$_2$Ph), 4.41 (d, *J* = 11.3 Hz, 1H, OC*H*$_2$Ph), 3.80 (dd, *J* = 8.1, 4.4 Hz, 1H, C^u*H*), 3.57 (dd, *J* = 10.8, 7.4 Hz, 1H, NC*H*$_2$), 3.50 (s, 1H, CHOCH$_2$Ph), 3.20 (d, *J* = 5.6 Hz, 1H, NC*H*$_2$), 3.03 (d, *J* = 8.1 Hz, 1H, C^v*H*), 2.85 (d, *J* = 13.8 Hz, 1H, (cy-C)C*H*$_2$), 2.72 (d, *J* = 7.4 Hz, 1H, NC*H*$_2$), 2.42 – 2.33 (m, 2H, NC*H*$_2$, (cy-C)C*H*$_2$)), 1.72 – 1.23 (m, 10H, cy-C*H*$_2$) ppm;

^{13}C NMR (126 MHz, $CDCl_3$): δ_C 157.1 (*C*=O), 156.2 (*C*=O), 138.4 (Ar*C*), 135.9 (C=*C*^{x}H), 131.4 (Ar*C*), 129.2 (Ar*C*H), 128.6 (Ar*C*H), 128.4 (Ar*C*H), 127.9 (Ar*C*H), 127.5 (Ar*C*H), 125.8 (C=*C*^{y}H), 125.7 (Ar*C*H), 79.3 (*C*HOCH$_2$Ph), 74.1 (O*C*H$_2$Ph), 71.2 (N*C*H$_2$), 61.9 (*C*^{u}H), 61.5 (*C*^{w}), 60.4 (*spiro*-*C*), 57.7 (*C*^{z}H), 56.1 (N*C*H$_2$), 44.0 (*C*^{v}H), 42.2 (cy-(*C*)), 37.3 ((cy-C)*C*H$_2$), 36.4 (cy-*C*H$_2$), 34.3 (cy-*C*H$_2$), 26.3 (cy-*C*H$_2$), 21.7 (cy-*C*H$_2$) ppm.

HRMS (ESI$^+$): m/z calc'd for $C_{32}H_{35}N_4O_3$ $[M+H]^+$: 523.2704 found: 523.2704.

IR (film): ν_{max} 2930, 2857, 1714 (C=O), 1500, 1402, 1071 cm^{-1}.

2.9. Unsuccessful substrates

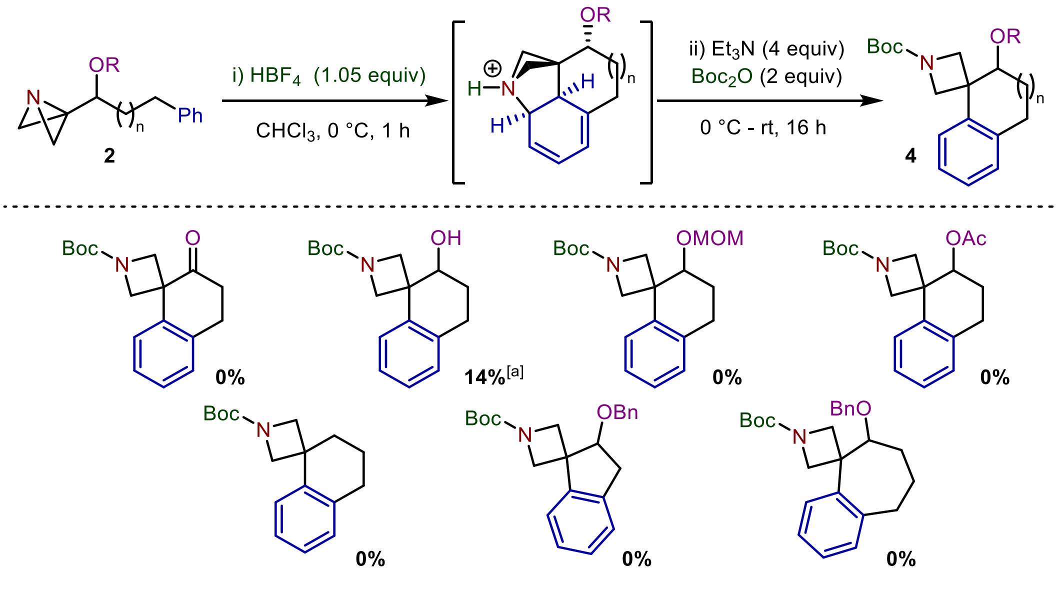

All reactions were carried out using **2** (0.20 mmol) in $CHCl_3$ (2.0 mL) according to **General Procedure E** (see above). Full consumption of starting material observed, complex mixture formed. [a] Yield was determined by ^{1}H NMR analysis using dibromomethane as an internal standard

Scheme S1: Unsuccessful substrates in the Friedel-Crafts spirocyclisation reaction.

3. STUDIES OF THE INTERRUPTED FRIEDEL-CRAFTS MECHANISM

3.1. Mechanistic studies of the interrupted Friedel-Crafts mechanism

The interrupted Friedel-Crafts mechanism was initially proposed to explain the observation of dearomatised diene species **3b**. This presumably forms from the direct interaction of the newly formed azetidine to the Wheland intermediate generated upon the Friedel-Crafts spirocylisation of the ABB-tethered aromatic ring. To determine whether rearomatisation to the Friedel-Crafts product was triggered upon addition of Et_3N or electrophilic activation with Boc_2O, a reaction under standard conditions was ran in parallel to one in which no Boc_2O was added (Scheme S2).

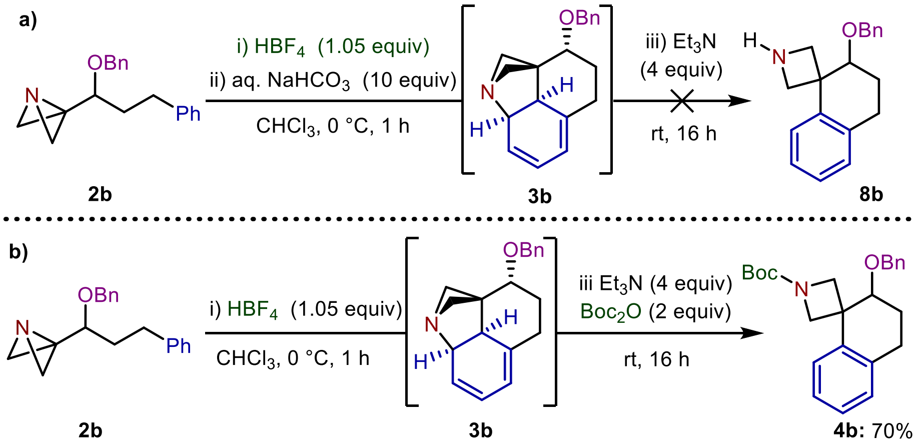

Scheme S2: Determination of the trigger for rearomatisation in the interrupted Friedel-Crafts spirocyclisation of 2b.

No Friedel-Crafts product was detected after stirring intermediate **3b** with Et_3N in the absence of Boc_2O, with the reaction in which Boc_2O was present giving exclusive formation of spiro-tetralin **4b**. This demonstrates that electrophilic activation of dearomatised diene **3b** is required to facilitate the collapse of the azabicyclo[2.1.1]hexane scaffold.

However, we predicted that an alternative mechanism which does not involve an azabicyclo[2.1.1]hexane species may be in operation in the case of substrates which cannot feasibly generate the corresponding intermediate. To demonstrate this, we subjected **2m** to our interrupted Friedel-Crafts procedure and observed that N–H azetidine **8m** was the only detectable species (Scheme S3). In this instance, C–N bond formation is presumably strongly disfavoured as this would require the complete dearomatisation of the naphthalene ring system. As a result, rearomatisation to form the Friedel-Crafts product occurs before the addition of Boc_2O and the N–H azetidine is simply protected in the second step.

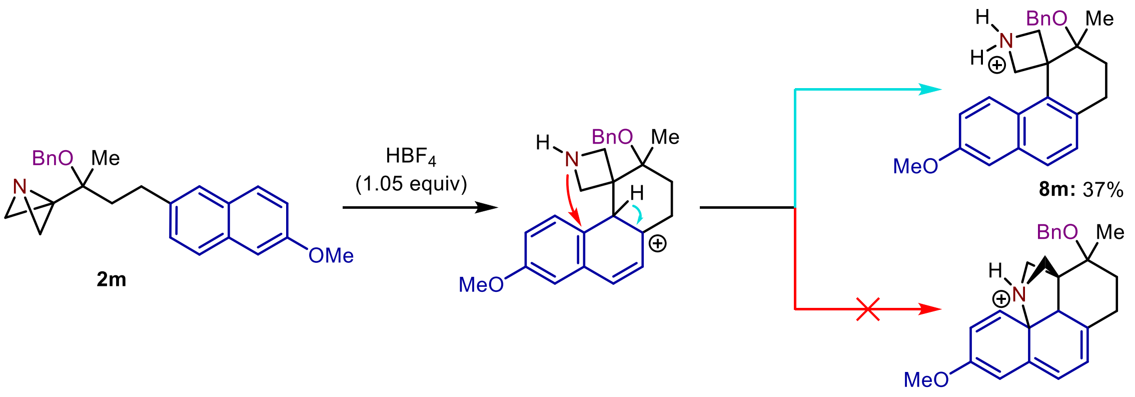

Scheme S3: Friedel-Crafts spirocyclisation of 2m.

3.2. Determination of the structure and stereochemistry of dearomatised intermediate 3

3-(Benzyloxy)-2a1,4,5,8a-tetrahydro-2H,3H-1,2a-methanobenzo[cd]indole: 3b

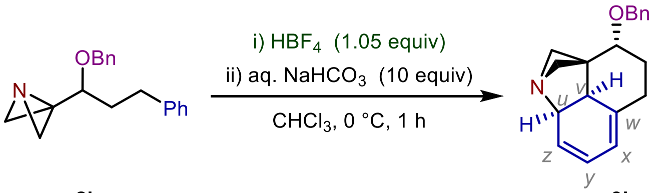

2b **3b**

Synthesised according to a modified **General Procedure G** from: **2b** (55.9 mg, 0.200 mmol, 1.00 equiv) and $HBF_4{\cdot}OEt_2$ (28.6 µL, 1.05 equiv). After work up, the yield of **3b** was determined to be 55% by ^{1}H NMR analysis using dibromomethane as an internal standard. The product was not purified further, and NMR spectroscopic data is provided below for the crude sample of **3b**.

NMR Spectroscopy (*see spectra*):

^{1}H NMR (500 MHz, $CDCl_3$): δ_H 7.39 – 7.28 (m, 5H, ArC*H*), 5.98 (dd, *J* = 9.8, 5.5 Hz, 1H, C=C^y*H*), 5.75 (dd, *J* = 9.8, 4.5 Hz, 1H, C=C^z*H*), 5.64 (d, *J* = 5.5 Hz, 1H, C=C^x*H*), 4.61 (d, *J* = 12.2 Hz, 1H, OC*H*$_2$Ph), 4.48 (d, *J* = 12.2 Hz, 1H, OC*H*$_2$Ph), 3.89 – 3.79 (m, 2H, C^u*H*, C*H*OCH$_2$Ph), 3.14 (d, *J* = 10.7 Hz, 1H, C^v*H*), 2.92 (d, *J* = 5.9 Hz, 1H, NC*H*$_2$), 2.80 (d, *J* = 6.1 Hz, 1H, NC*H*$_2$), 2.55 – 2.45 (m, 1H, C*H*$_2$(C=C)), 2.36 (dd, *J* = 9.7, 6.1 Hz, 1H, NC*H*$_2$), 2.29 (dd, *J* = 9.7, 5.9 Hz, 1H, NC*H*$_2$), 2.15 – 1.99 (m, 2H, C*H*$_2$(C=C), CH(OCH$_2$Ph)C*H*$_2$), 1.30 – 1.21 (m, 1H, CH(OCH$_2$Ph)C*H*$_2$) ppm;

^{13}C NMR (126 MHz, $CDCl_3$): δ_C 140.2 (C=*C*^{w}),138.9 (Ar*C*), 128.6 (Ar*C*H), 127.9 (Ar*C*H), 127.8 (C=*C*^{y}H), 127.4 (Ar*C*H), 122.7 (C=*C*^{z}H), 115.9 (C=*C*^{x}H), 73.3 (*C*HOCH$_2$Ph), 70.9 (O*C*H$_2$Ph), 65.3 (*spiro-C*), 62.4 (*C*^{u}H), 61.4 (N*C*H$_2$), 58.5 (N*C*H$_2$), 39.4 (*C*^{v}H), 29.6 (C=C*C*H$_2$,) 29.4 (CH(OCH$_2$Ph)*C*H$_2$) ppm.

HRMS (ESI$^+$): m/z calc'd for $C_{19}H_{22}NO$ $[M+H]^+$: 280.1696 found: 280.1694.

IR (film): ν_{max} 3030, 2928, 2855, 1496, 1454, 1093, 1070 cm^{-1}.

The structure of the observed interrupted Friedel-Crafts intermediate can be evidenced from the NMR

spectroscopic data obtained from the pure sample of **3j** in combination with HRMS. Heteronuclear multiple bond correlation (HMBC) spectroscopy provided evidence for the connectivity as the correlation of signals between the azetidine methylenes and allylic methine/alkenyl fragment was clearly observed (Figure S1). This same correlation is not seen in similar structures in which the key C-N bond is absent.

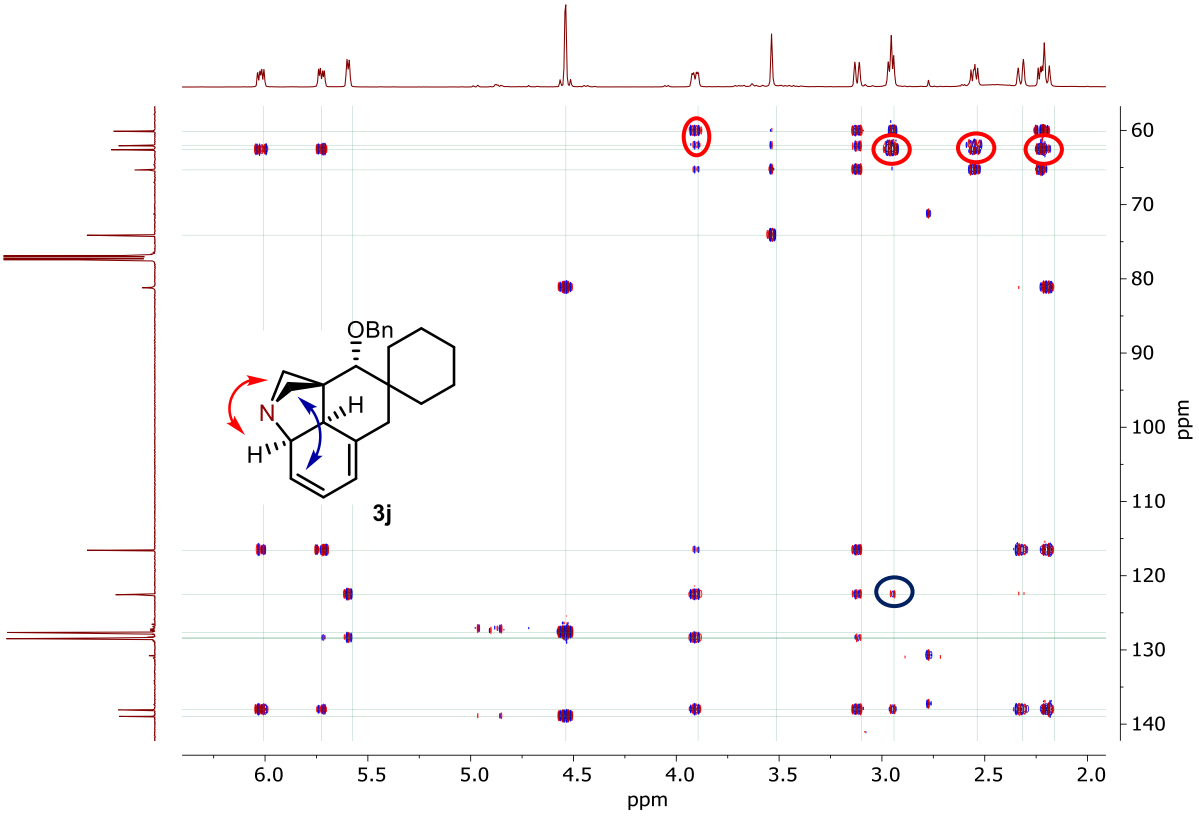

Figure S1: HMBC spectrum of 3j with key signal correlations highlighted.

To determine the relative stereochemistry and further confirm the structure of the proposed dearomatised intermediate, Diels-Alder adduct **5b** was formed in which the key azabicyclo[2.1.1]hexane scaffold is retained (Scheme S4). This substrate was selected as the coupling constants between the α-benzyloxy proton and the adjacent methylene protons can help elucidate the stereochemistry in this rigid structure.

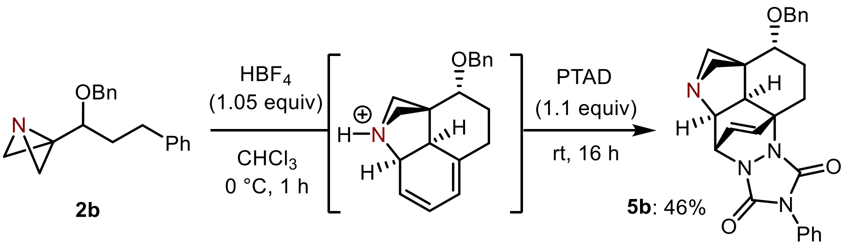

Scheme S4: Interrupted Friedel-Crafts/Diels-Alder dearomatisation reaction for the synthesis of 5b.

Initial evidence of the relative stereochemistry of **5b** was obtained by analysis of the coupling constants of the methine proton of the key stereocentre (**H**^{26}). Energy minimised structures of the two possible diastereomers

show that in diastereomer **A**, the protons of the adjacent methylene signal (**H**^{24}, **H**^{25}) have a dihedral angle of approximately 180° and 60° to **H**^{26} (Figure S2). This relationship would therefore result in a large (>10 Hz) and a small (<5 Hz) coupling constant to **H**^{26} according to the Karplus equation. In diastereomer **B** both protons lie at approximately 60° from **H**^{26}, resulting in two similar, small coupling constants. This matches what is observed spectroscopically for **5b**, with coupling constants of 2.62 and 2.63 Hz (see spectra), supporting the formation of diastereomer **B**.

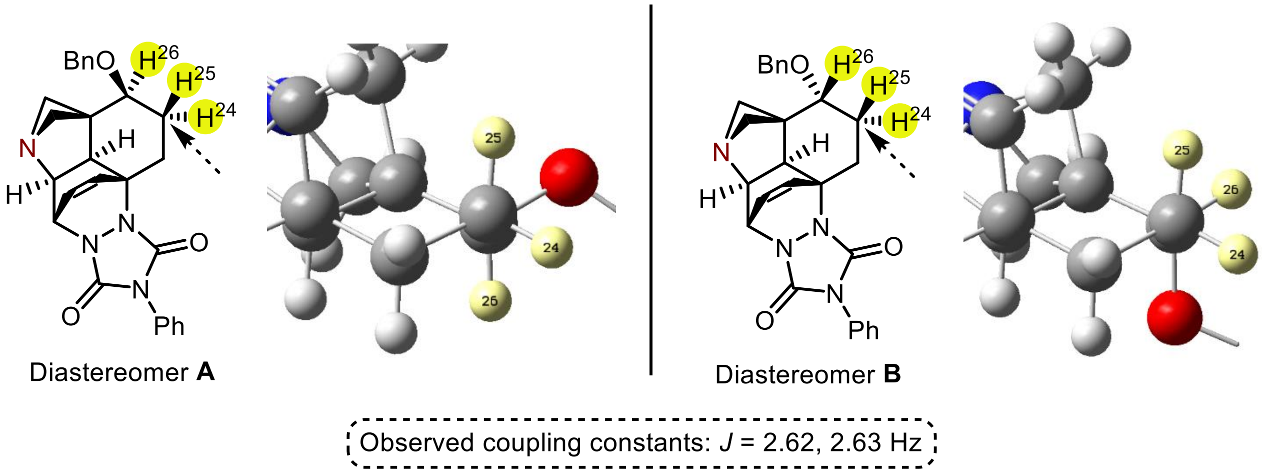

Diastereomer **A** Diastereomer **B**

Observed coupling constants: J = 2.62, 2.63 Hz

DFT at the B3LYPD3/6-311+G** level with the integral equation formalism variant of the polarisable continuum model of solvation (IEFPCM: chloroform).

Figure S2: Coupling constant analysis for the stereochemical assignment of 5b.

Further evidence to support that the correct stereochemistry of **5b** was that of diastereomer **B** was obtained through the use of 1D NOESY experimentation (Figure S3). Irradiation of the **H**^{26} signal showed a similar degree of through space coupling to the adjacent methylene protons (**H**^{24}, **H**^{25}). A much smaller through space coupling is observed to **H**^{27}. As this interaction directly scales with interatomic distance, the data indicates that **H**^{26} must be a similar distance away from **H**^{24} and **H**^{25}, and should show a larger interatomic distance to **H**^{27}. This fits with diastereomer **B** which displays calculated interatomic distances of 2.51 Å and 2.43 Å to **H**^{24} and **H**^{25}, respectively, and 3.81 Å to **H**^{27}. Diastereomer **A** shows calculated interatomic distances of 2.49 Å and 3.06 Å to **H**^{24} and **H**^{25}, respectively, and 2.61 Å to hydrogen **H**^{27}. In this case, the NOE signal of **H**^{24} and **H**^{27} should be similar, whereas the interaction to **H**^{25} should be noticeably weaker, which does not match the observed results.

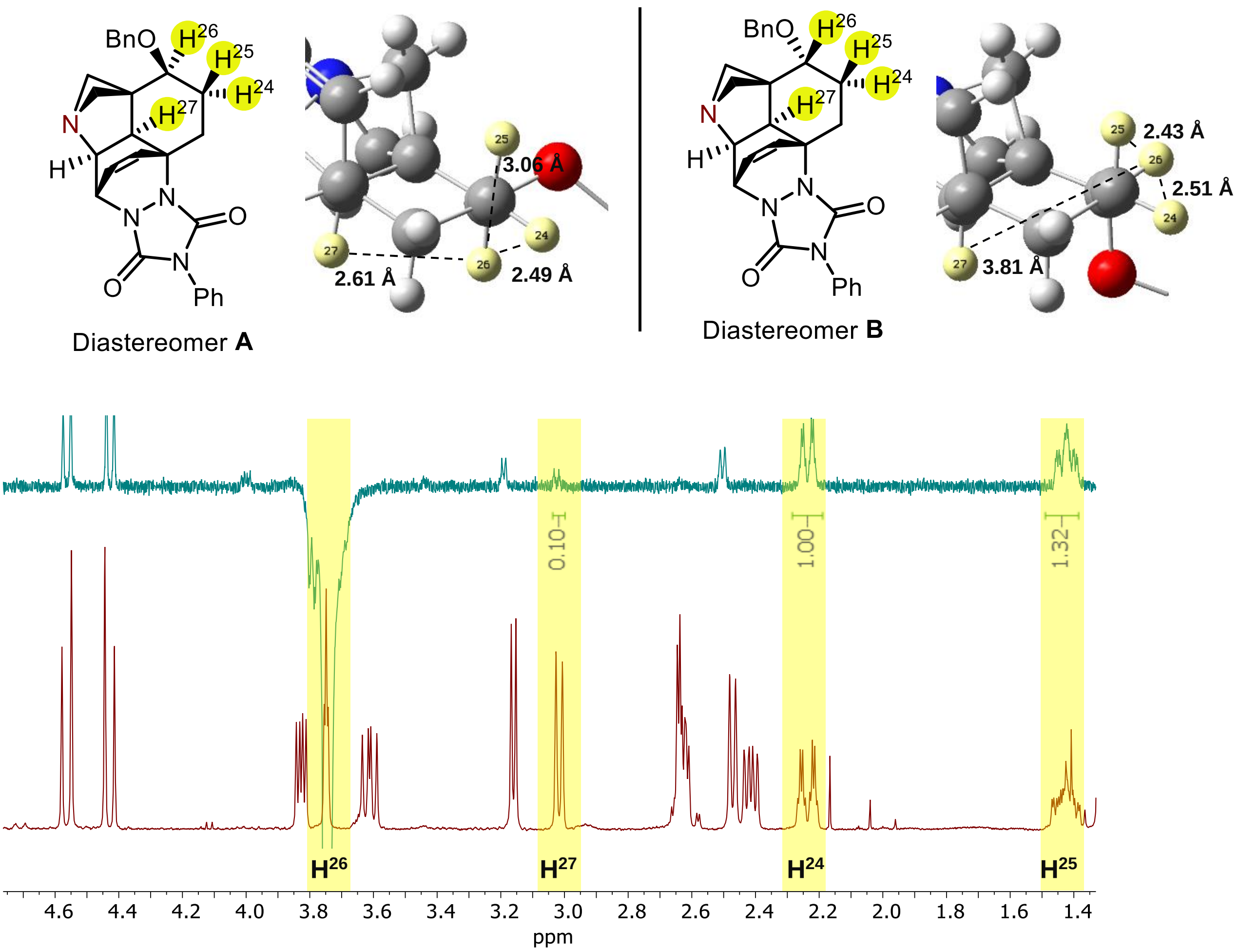

DFT at the B3LYPD3/6-311+G** level with the integral equation formalism variant of the polarisable continuum model of solvation (IEFPCM: chloroform).

Figure S3: 1D NOESY analysis for the stereochemical assignment of 5b.

3.3. Stereochemistry of terminal alkene spirocycle 4p

The stereochemistry of spirocycle **4p** was initially assigned by analogy to Friedel-Crafts intermediates **3**. However, to confirm this we determined the stereochemistry of azabicyclo[2.1.1]hexane intermediate **3p** in the spirocyclisation reaction (Scheme S5). The rigidity of this scaffold allows the elucidation of the relative stereochemistry of the newly formed stereocentre (which remains unchanged in the final product) through an analysis of the ^{1}H NMR coupling constants.

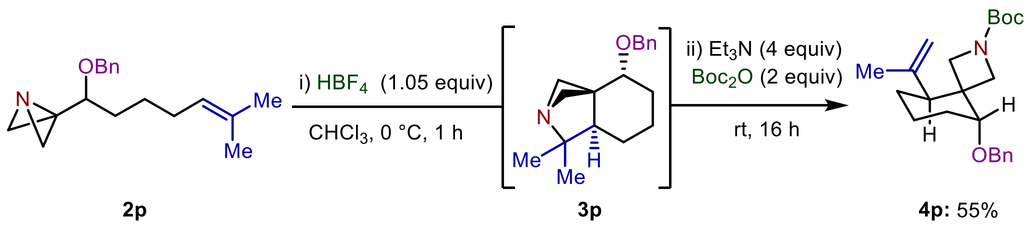

Scheme S5: Observed azabicyclo[2.1.1]hexane intermediate in the spirocyclisation of 2p.

Energy minimised structures of the two possible diastereomers show that in diastereomer **A**, protons **H**^{23} and **H**^{24} have dihedral angles of approximately 60° to **H**^{25} (Figure S4). This relationship would therefore result in two similar, small coupling constants (<5 Hz) according to the Karplus equation. This matches what is observed for **3p**, with coupling constants of 2.75 and 2.71 Hz (see spectra), suggesting diastereomer **A** is the correct stereochemistry. In diastereomer **B**, protons **H**^{23} and **H**^{24} have dihedral angles of approximately 35° and 80° to **H**^{25}, which would give one small coupling constant (<5 Hz) and another in the range of 6-9 Hz.

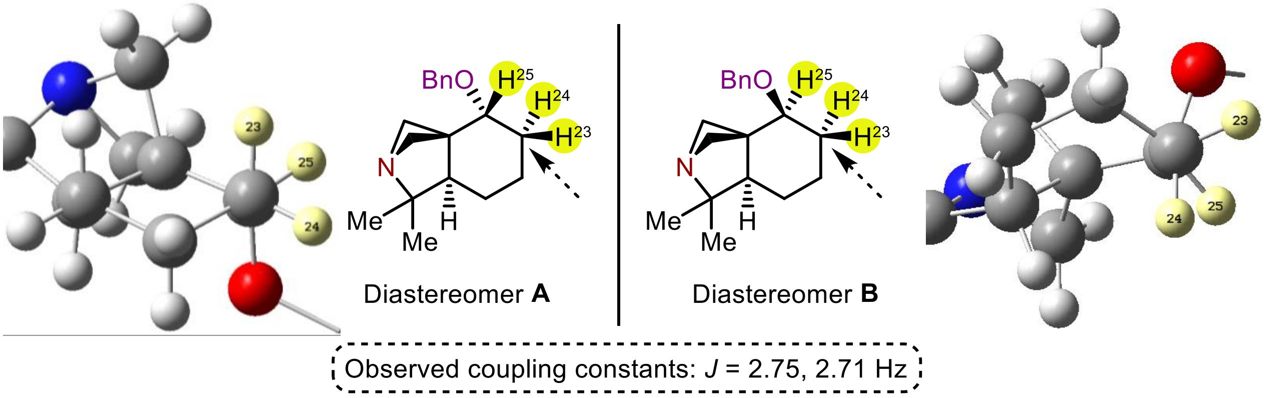

DFT at the B3LYPD3/6-311+G** level with the integral equation formalism variant of the polarisable continuum model of solvation (IEFPCM: chloroform). Coupling constants quoted are those observed for the free-base of **3p**.

Figure S4: Coupling constant analysis for the stereochemical assignment of 3p.

The same analysis can also be performed on methine proton **H**^{11}. In diastereomer **A**, protons **H**^{19} and **H**^{20} have dihedral angles of approximately 170° and 60° to **H**^{11} (Figure S5). This relationship would result in a large (>10 Hz) and a small (<5 Hz) coupling constant according to the Karplus equation. This matches what is observed for **3p**, with coupling constants of 12.5 and 4.2 Hz (see spectra), suggesting diastereomer **A** is the correct stereochemistry. In diastereomer **B**, protons **H**^{19} and **H**^{20} have dihedral angles of 140° and 20° to **H**^{11}. This relationship would result in two coupling constants in the range of 7-10 Hz.

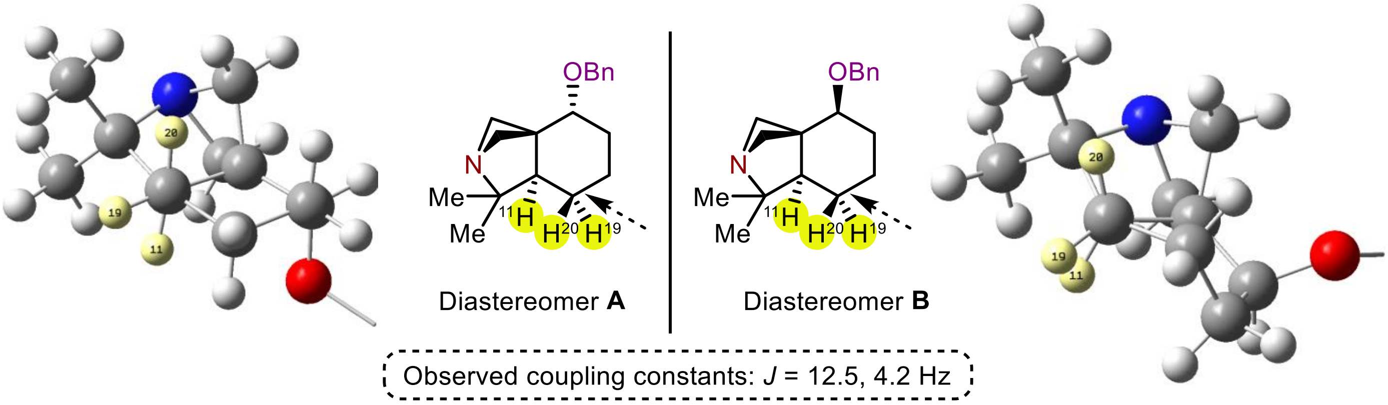

DFT at the B3LYPD3/6-311+G** level with the integral equation formalism variant of the polarisable continuum model of solvation (IEFPCM: chloroform). Coupling constants quoted are those observed for the free-base of **3p**.

Figure S5: Further coupling constant analysis for the stereochemical assignment of 3p.

3.4. Unsuccessful derivatisation of dearomatised intermediate 3b

As well as [4+2] cycloaddition reactions, dearomatized intermediate **3b** was also submitted to hydrogenation, oxidation, dihydroxylation and epoxidation conditions (Scheme S6). However, under these conditions no desired product which retains the key azabicyclo[2.1.1]hexane framework could be observed. In all cases Friedel-Crafts products could be detected in minor amounts, showing that rearomatisation/elimination was taking place. However, the reactions returned complex mixtures from which no individual species could be isolated.

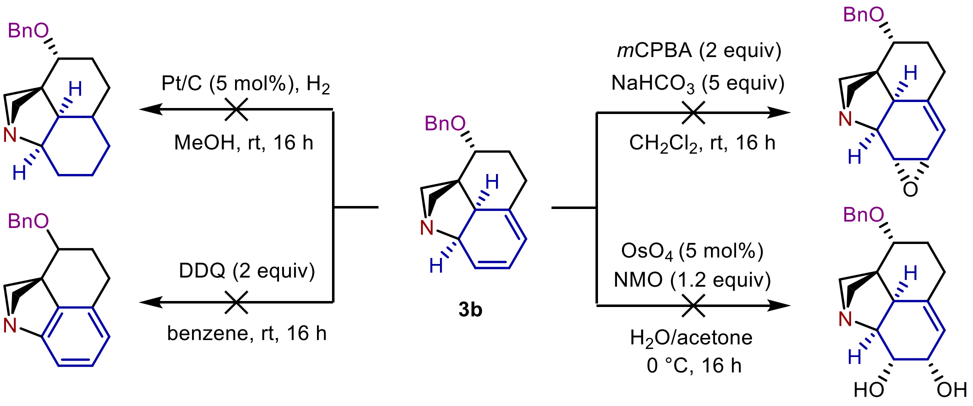

Scheme S6: Attempted derivatisation reactions of dearomatised intermediate 3b.

4. X-RAY CRYSTALLOGRAPHIC ANALYSIS

4.1. 4r (CCDC number: 2110073) and 8m (CCDC number: 2112703)

X-ray diffraction experiments on **4r** were carried out at 100(2) K on a Bruker D8 Venture diffractometer using Mo-K_α radiation (λ = 0.71073 Å), while **8m** was carried out at 100(2) K on a Bruker D8 Venture diffractometer using Cu-K_α radiation (λ = 1.54178 Å). Data collections were performed using a Bruker CPAD detector. Intensities were integrated in SAINT[14] and absorption corrections based on equivalent reflections were applied using SADABS.[15] The structure was solved using ShelXT[16] and refined by full matrix least squares against F^2 in ShelXL[17,18] using Olex2.[19] All of the hydrogen atoms were located geometrically and refined using a riding model, apart from the N-H protons in **8m** which were located in the difference map. In the case of **8m** the counterion displayed disorder and was modelled in two positions with a refined occupancy ratio of 0.75:0.25(4), SADI and SIMU were used to maintain sensible geometries and thermal parameters. The crystal structure and refinement data are given in Table S5. Crystallographic data for compounds **4r** and **8m** has been deposited with the Cambridge Crystallographic Data Centre as supplementary publications CCDC 2110073 and 2112703, respectively. Copies of the data can be obtained free of charge on application to CCDC, 12 Union Road, Cambridge CB2 1EZ, UK [fax(+44) 1223 336033, e-mail: deposit@ccdc.cam.ac.uk].

**Compound**	**4r**	**8m**
CCDC number	2110073	2112703
Empirical formula	$C_{31}H_{35}NO_3S$	$C_{25}H_{28}BF_4NO_2$
Formula weight	501.66	461.29
Temperature/K	100.0	100.0
Crystal system	orthorhombic	triclinic
Space group	$Pca2_1$	P-1
a/Å	18.1702(9)	9.3356(3)
b/Å	10.7306(5)	10.1259(3)
c/Å	13.1412(7)	13.0958(4)
α/°	90	82.8910(10)
β/°	90	79.3670(10)
γ/°	90	63.3710(10)
Volume/Å^3	2562.2(2)	1086.46(6)
Z	4	2
ρ_{calc}g/cm^3	1.300	1.410
μ/mm^{-1}	0.160	0.935
F(000)	1072.0	484.0
Crystal size/mm^3	0.341 × 0.16 × 0.08	0.349 × 0.161 × 0.14
Radiation	MoKα (λ = 0.71073)	CuKα (λ = 1.54178)
2Θ range for data collection/°	4.408 to 50.692	6.874 to 136.486
Index ranges	-21 ≤ h ≤ 17<br>-12 ≤ k ≤ 11<br>-15 ≤ l ≤ 15	-11 ≤ h ≤ 11<br>-12 ≤ k ≤ 11<br>-15 ≤ l ≤ 15
Reflections collected	26397	14448
Independent reflections	4652 [R_{int} = 0.0716, R_{sigma} = 0.0639]	3964 [R_{int} = 0.0351, R_{sigma} = 0.0331]
Data/restraints/parameters	4652/1/326	3964/220/354
Goodness-of-fit on F^2	1.058	1.038
Final R indexes [I>=2σ (I)]	R_1 = 0.0395, wR_2 = 0.0770	R_1 = 0.0446, wR_2 = 0.1214
Final R indexes [all data]	R_1 = 0.0633, wR_2 = 0.0859	R_1 = 0.0476, wR_2 = 0.1239
Largest diff. peak/hole / e Å^{-3}	0.21/-0.32	0.40/-0.38

Table S5: Crystal data and structure refinement for 4r and 8m.

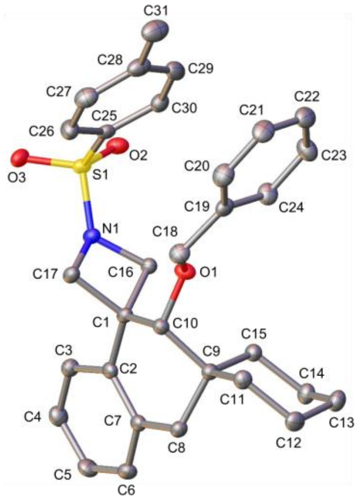

Figure S6: Crystal structure of 4r with the anisotropic displacement parameters depicted at the 50% probability level and hydrogens omitted for clarity.

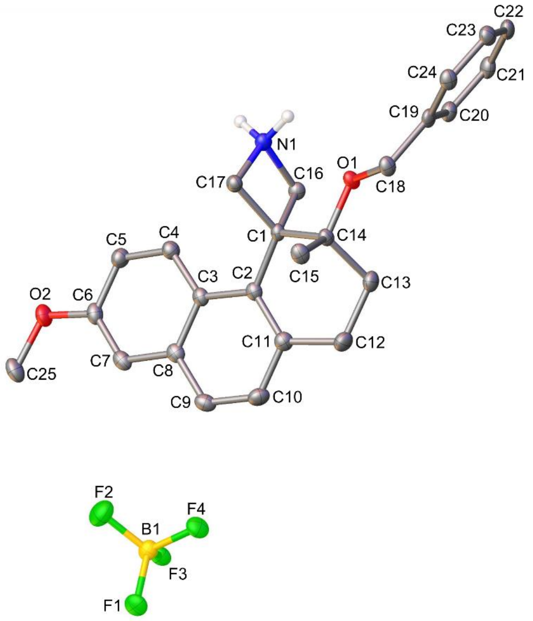

Figure S7. Crystal structure of 8m with the anisotropic displacement parameters depicted at the 50% probability level. Disorder on the counterion and hydrogens, except those on the heteroatom, omitted for clarity.

5. SPECTROSCOPIC DATA

^{1}H NMR (400 MHz, $CDCl_3$) of **7f** (*see procedure*)

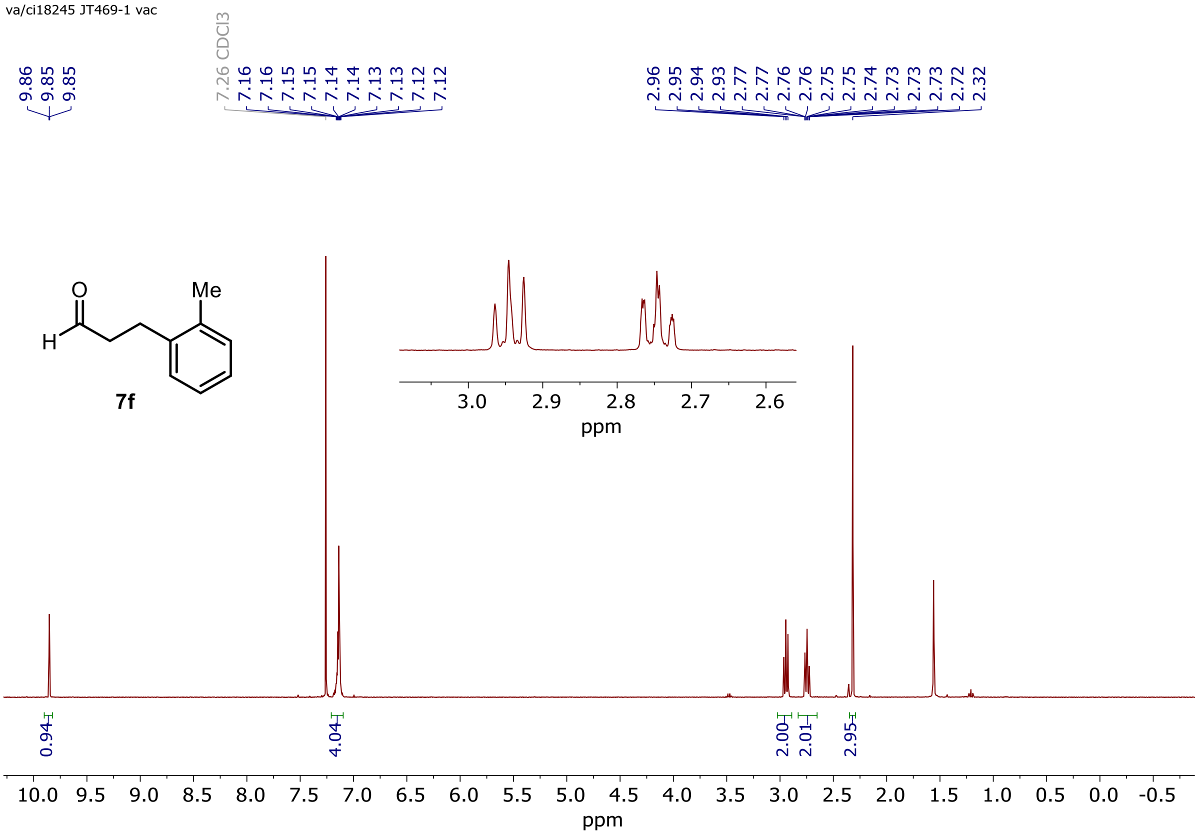

^{13}C NMR (126 MHz, $CDCl_3$) of **7f**

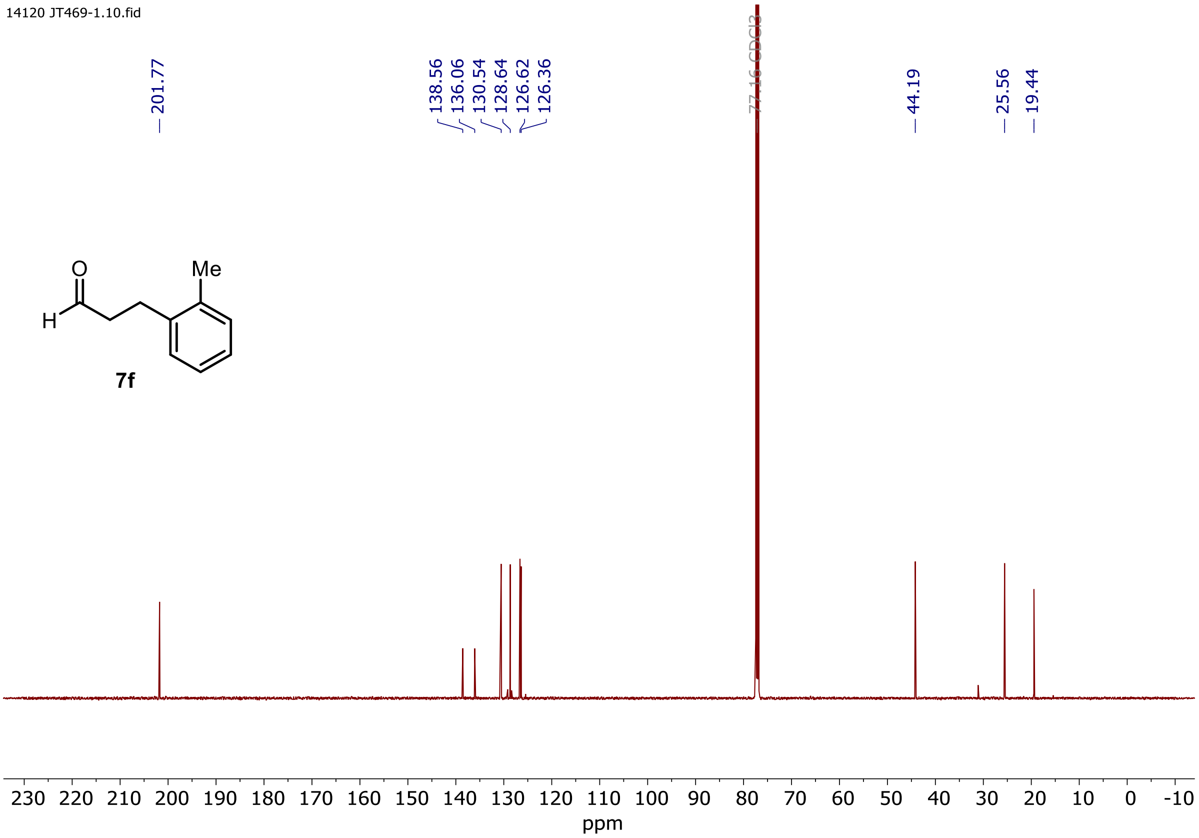

^{1}H NMR (400 MHz, $CDCl_3$) of **7g** (*see procedure*)

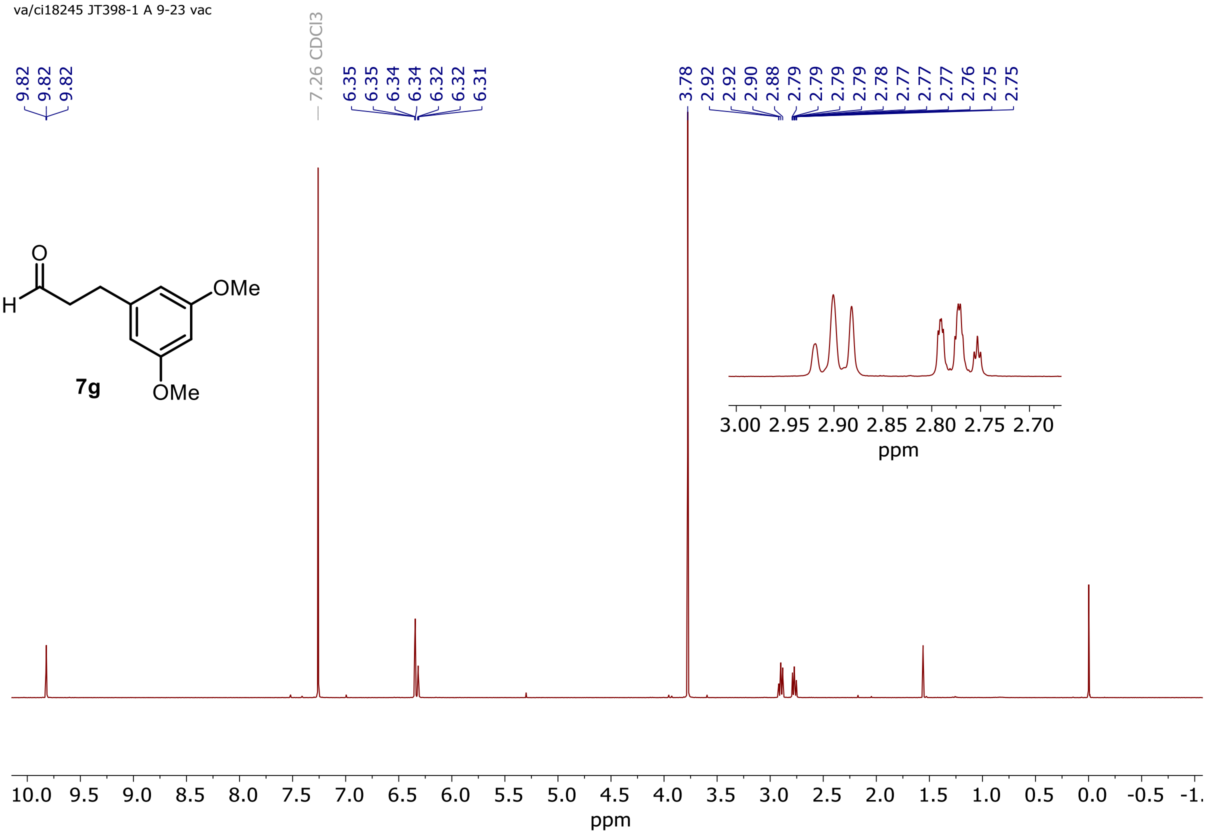

^{13}C NMR (126 MHz, $CDCl_3$) of **7g**

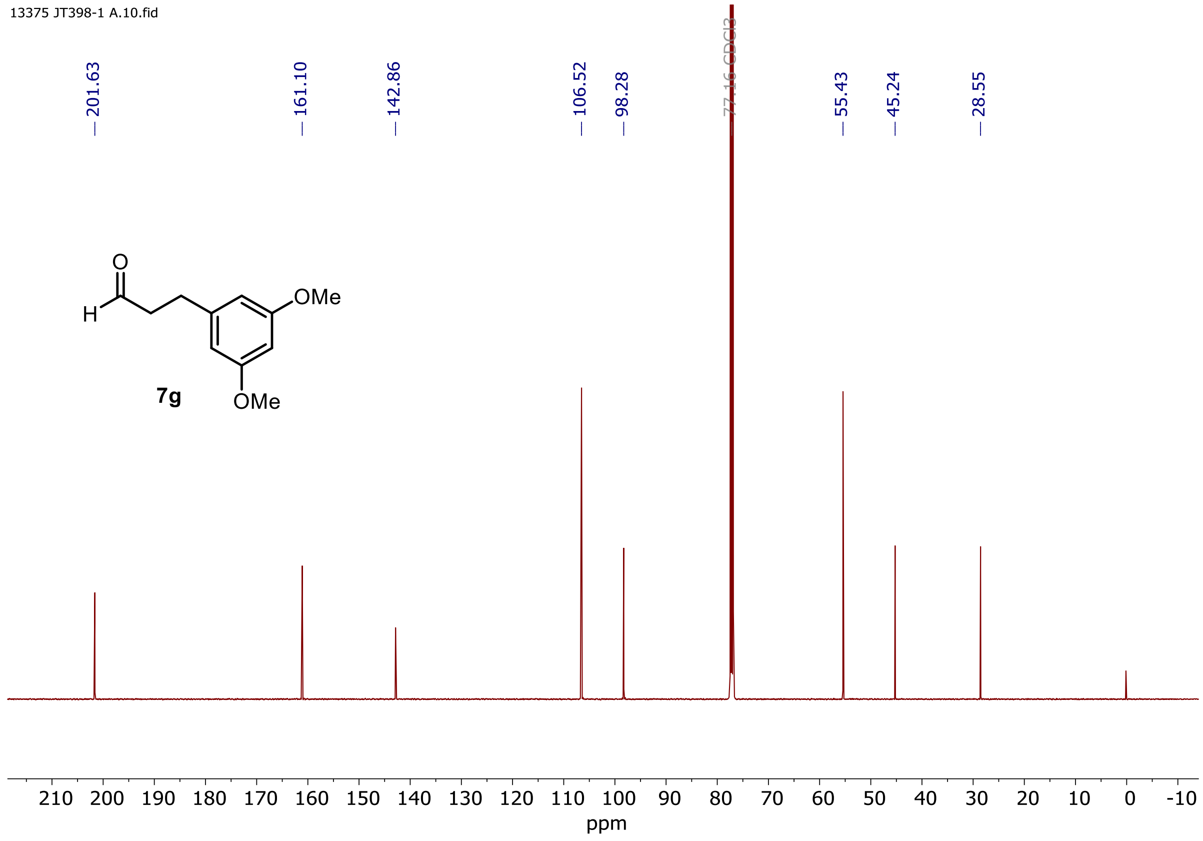

^{1}H NMR (400 MHz, $CDCl_3$) of **7h** (see procedure)

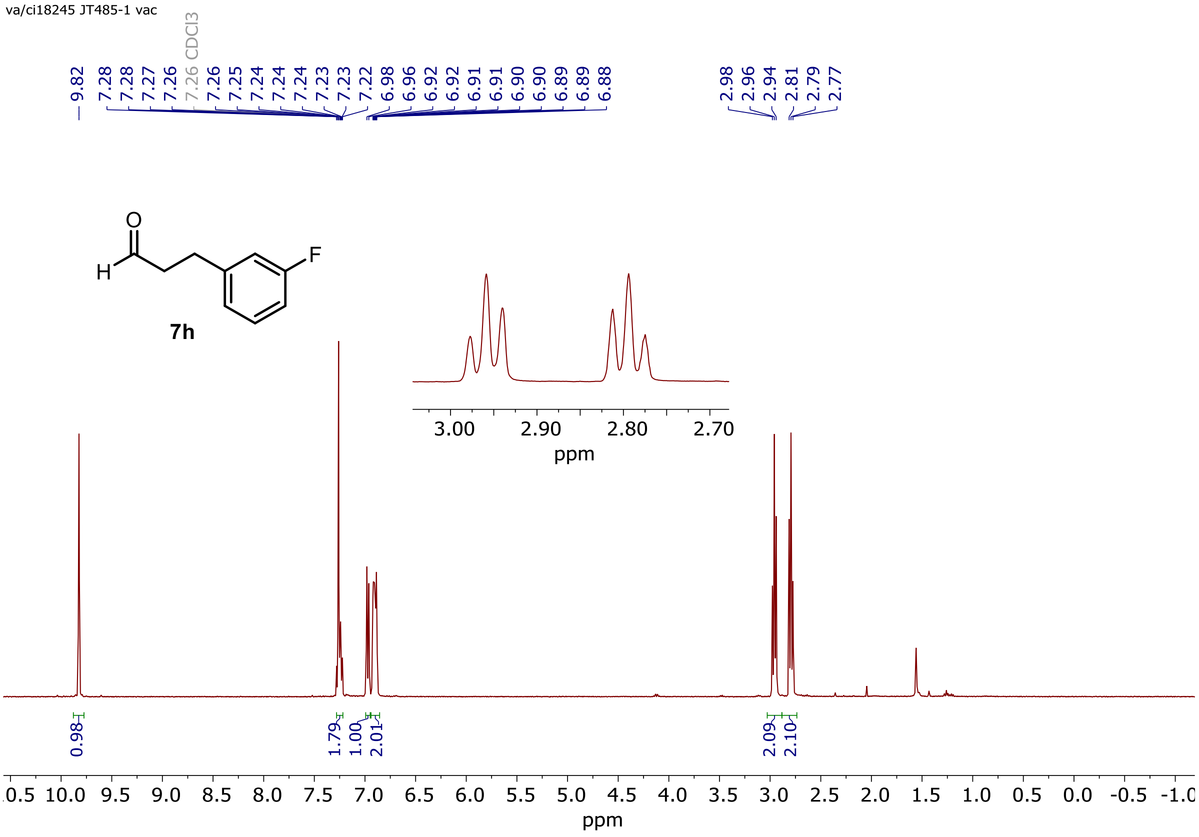

^{13}C NMR (126 MHz, $CDCl_3$) of **7h**

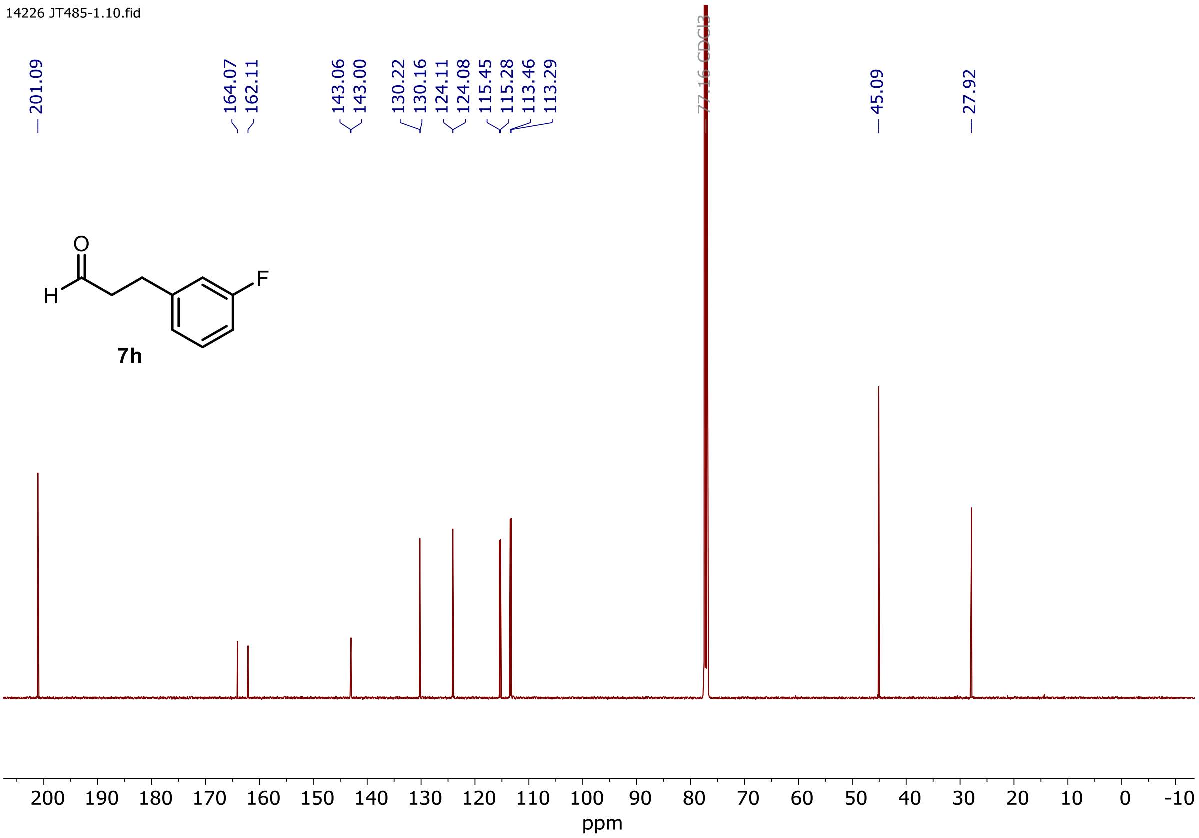

^{1}H NMR (500 MHz, $CDCl_3$) of **7j** (*see procedure*)

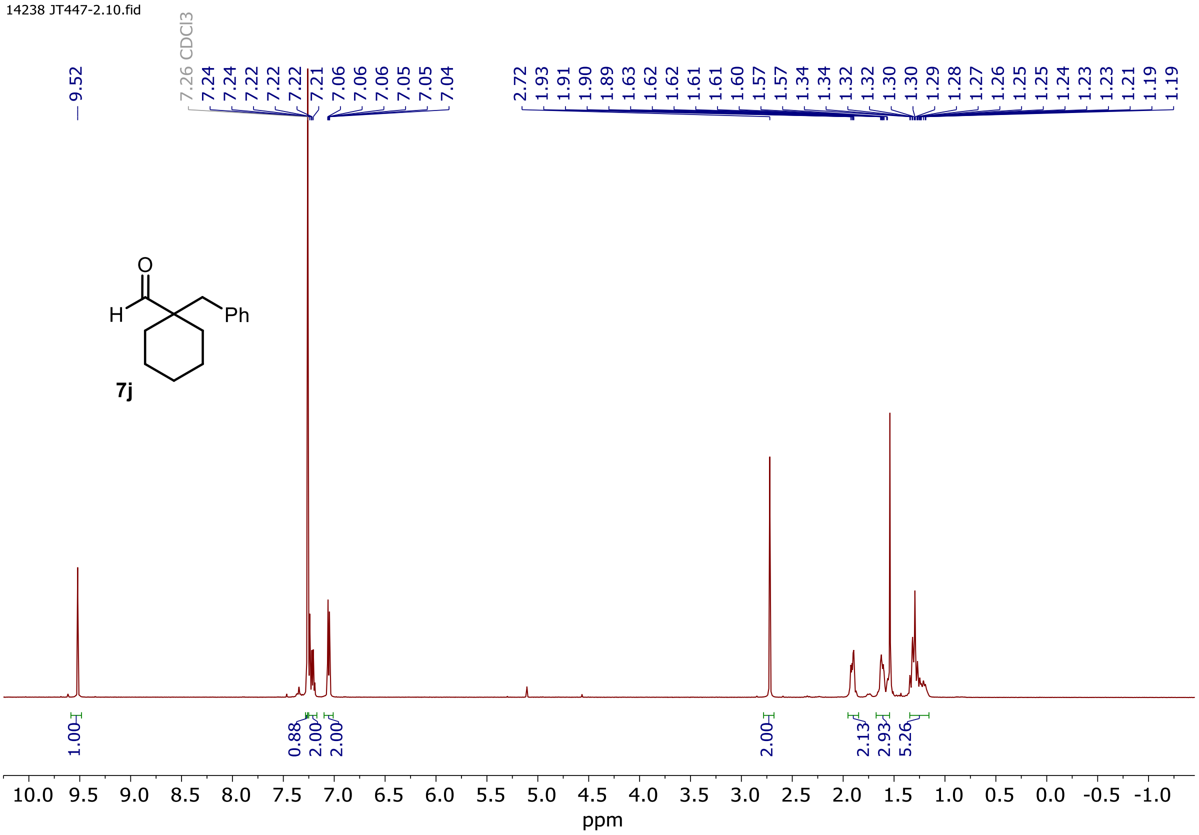

^{13}C NMR (126 MHz, $CDCl_3$) of **7j**

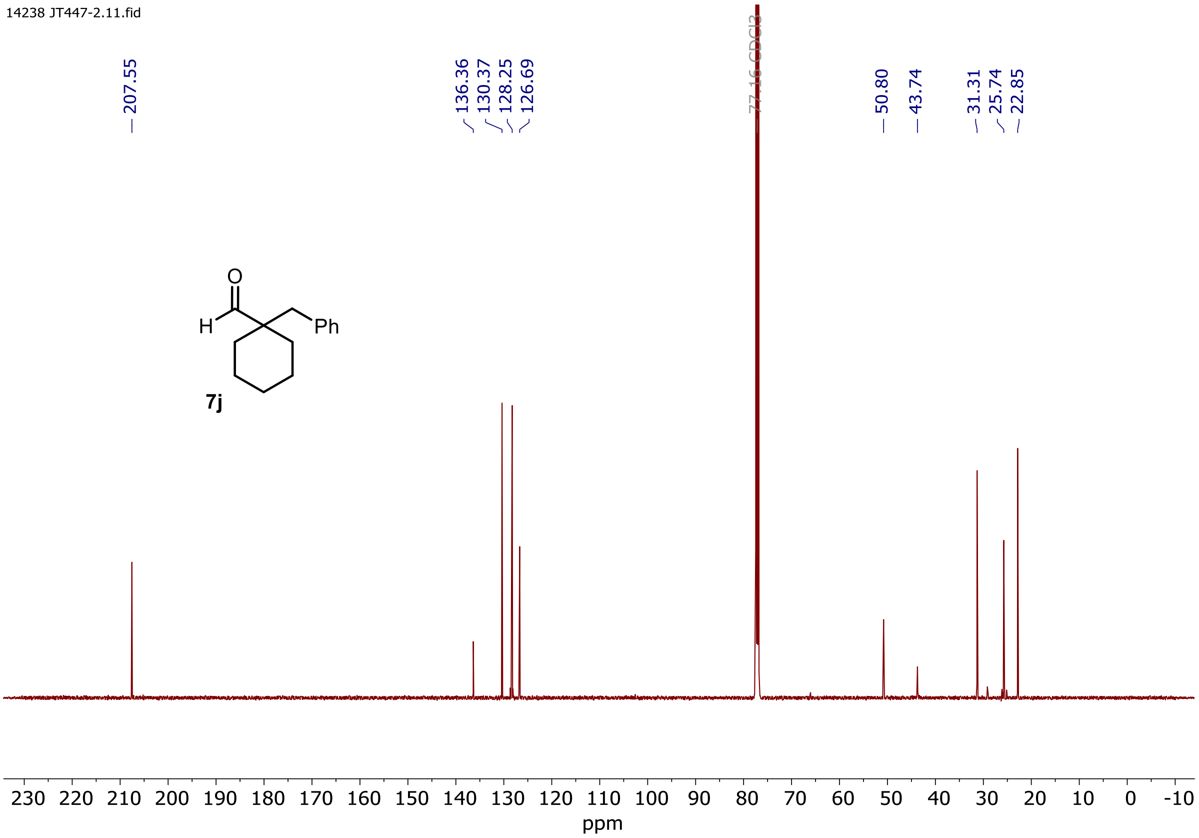

^{1}H NMR (400 MHz, $CDCl_3$) of **7k** (*see procedure*)

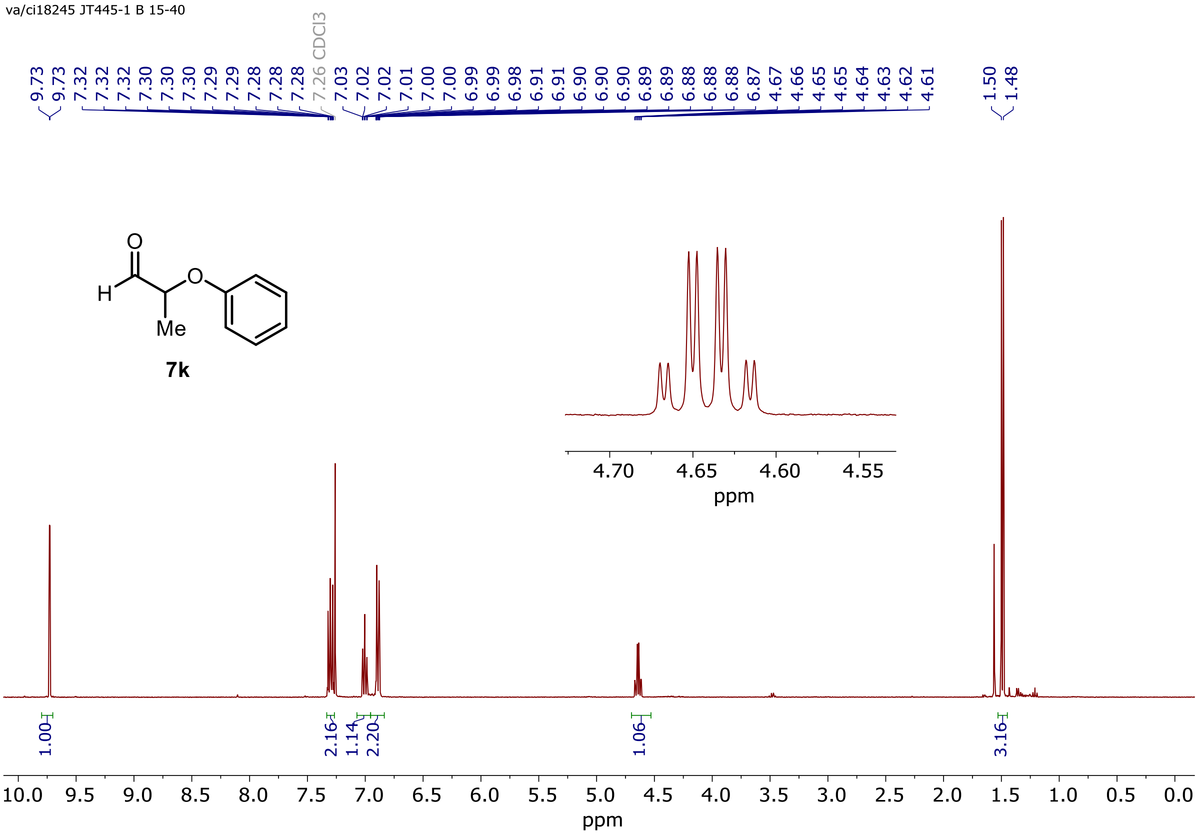

^{13}C NMR (126 MHz, $CDCl_3$) of **7k**

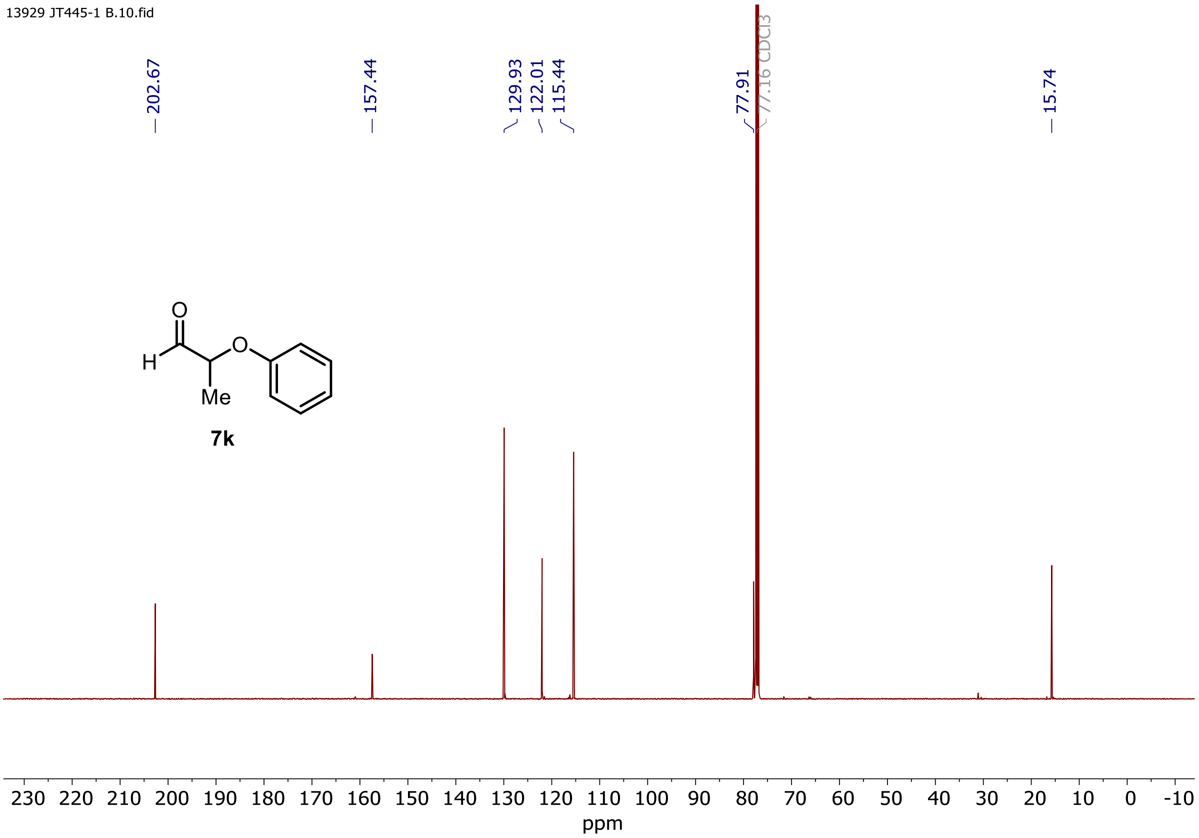

^{1}H NMR (400 MHz, $CDCl_3$) of **7l** (*see procedure*)

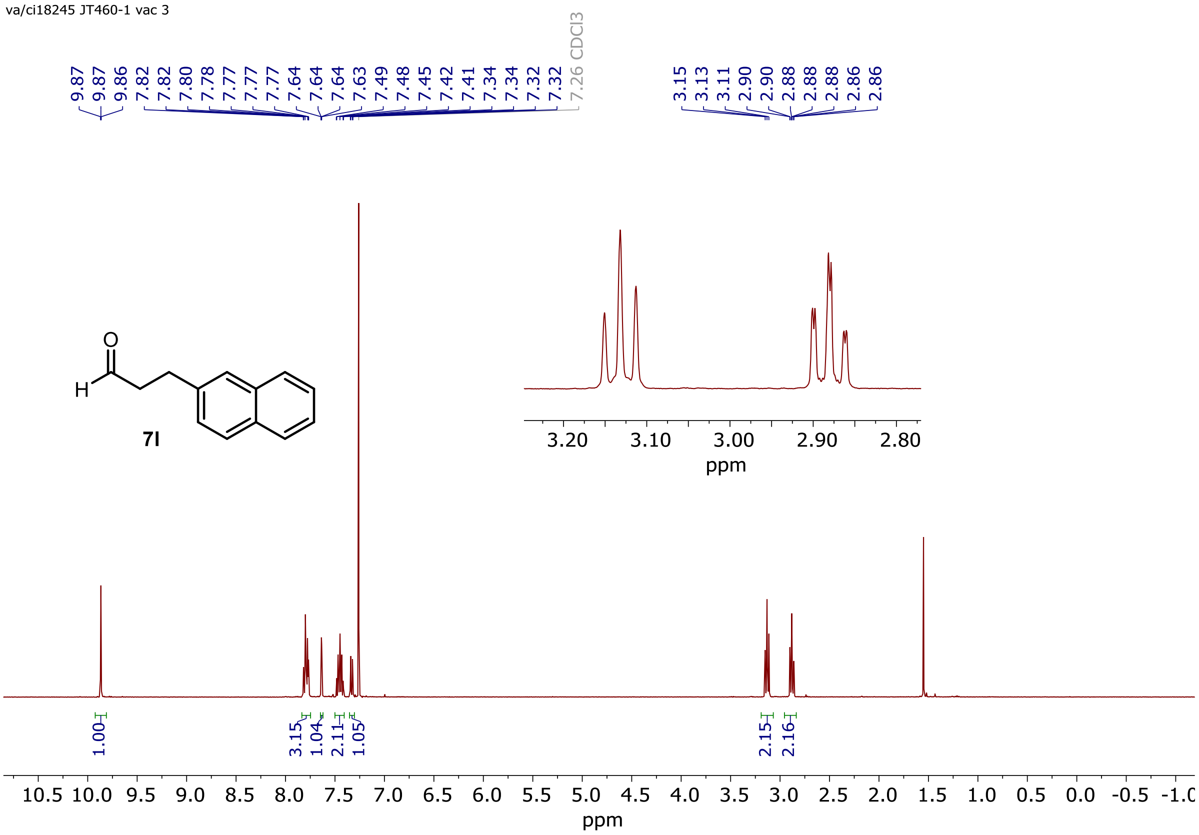

^{13}C NMR (126 MHz, $CDCl_3$) of **7l**

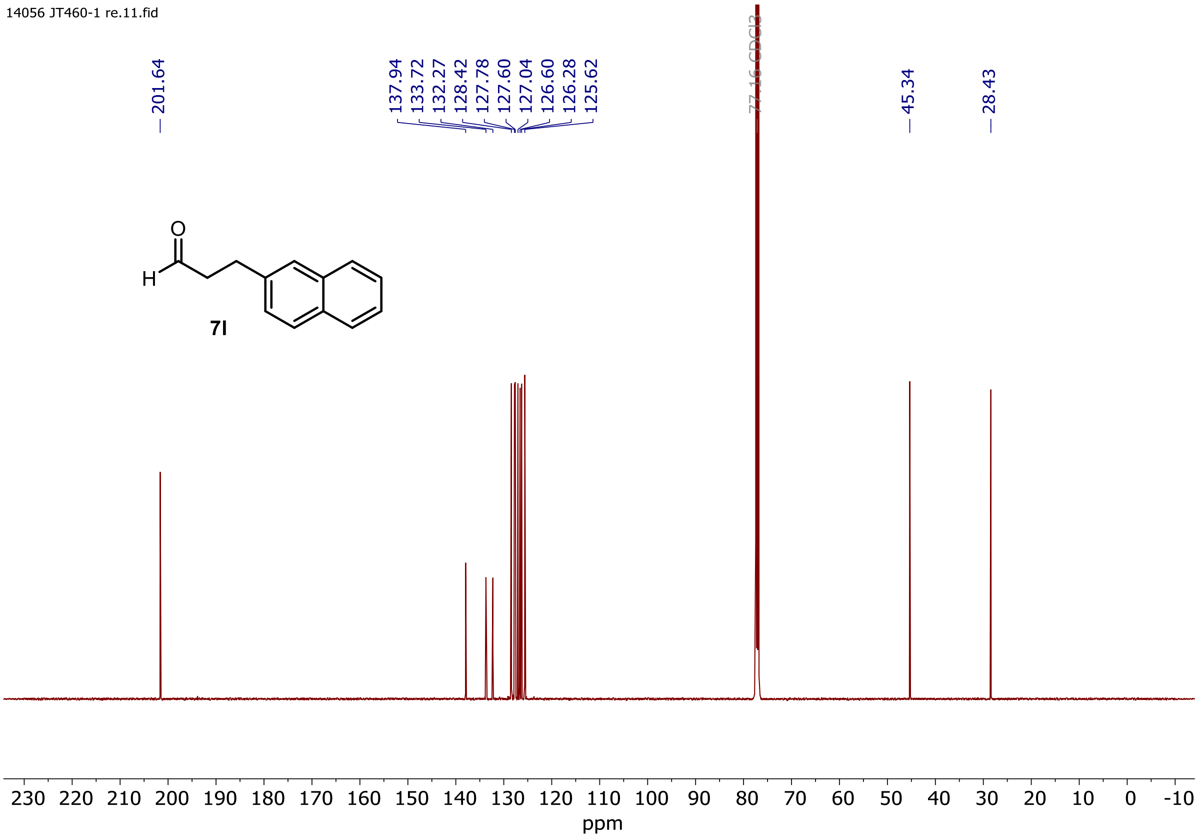

^{1}H NMR (400 MHz, $CDCl_3$) of **7o** (*see procedure*)

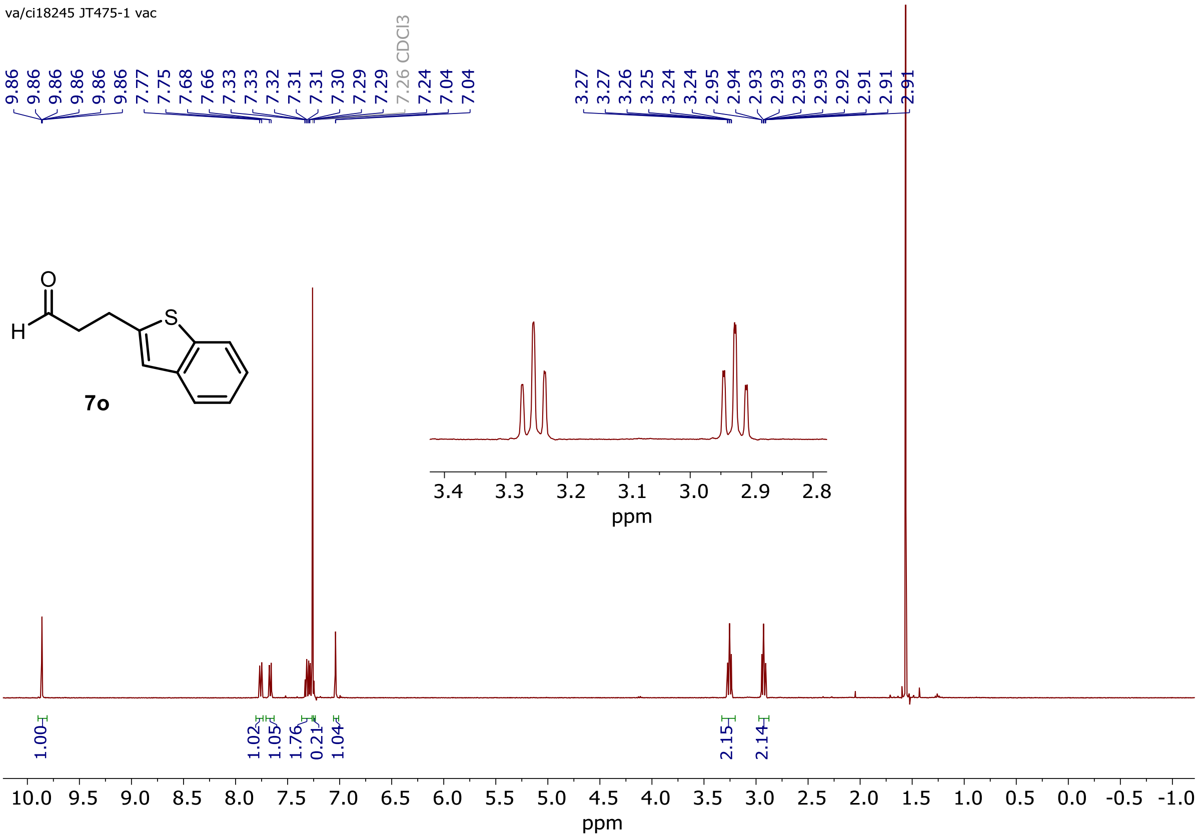

^{13}C NMR (126 MHz, $CDCl_3$) of **7o**

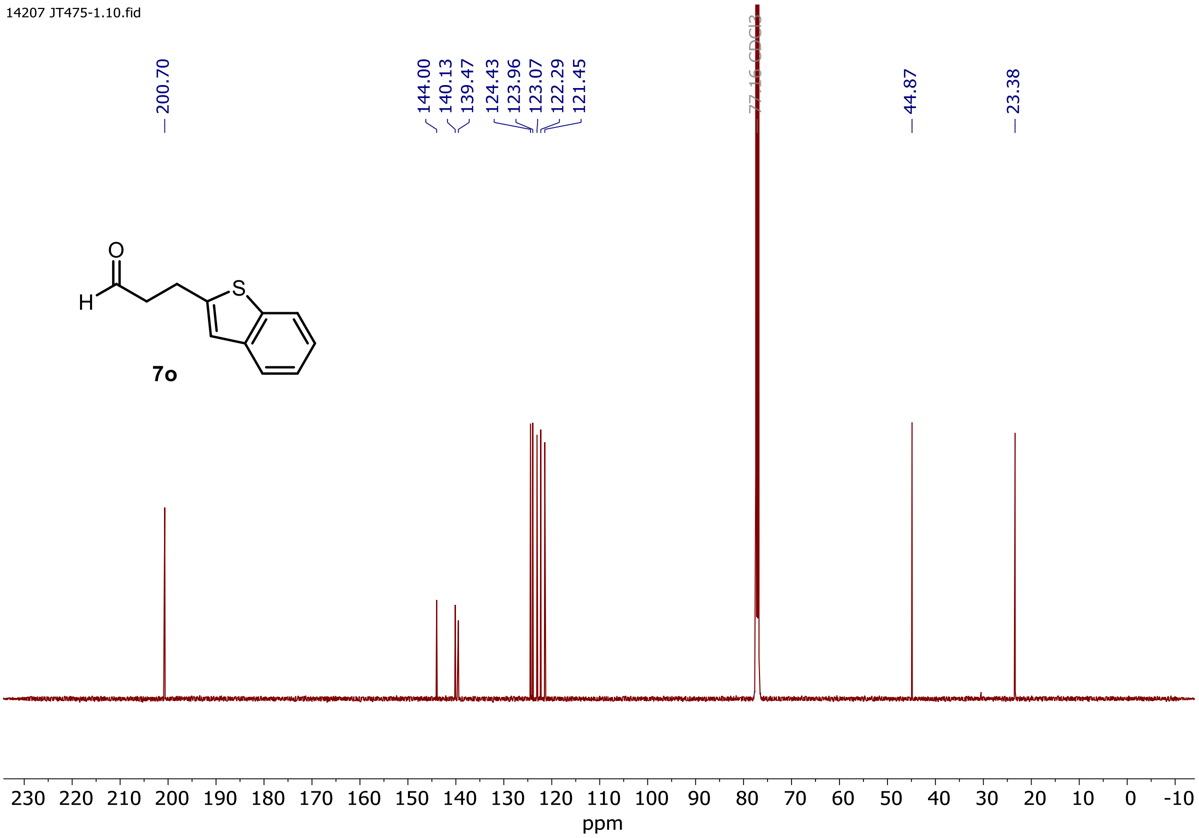

^{1}H NMR (400 MHz, $CDCl_3$) of **7p** (*see procedure*)

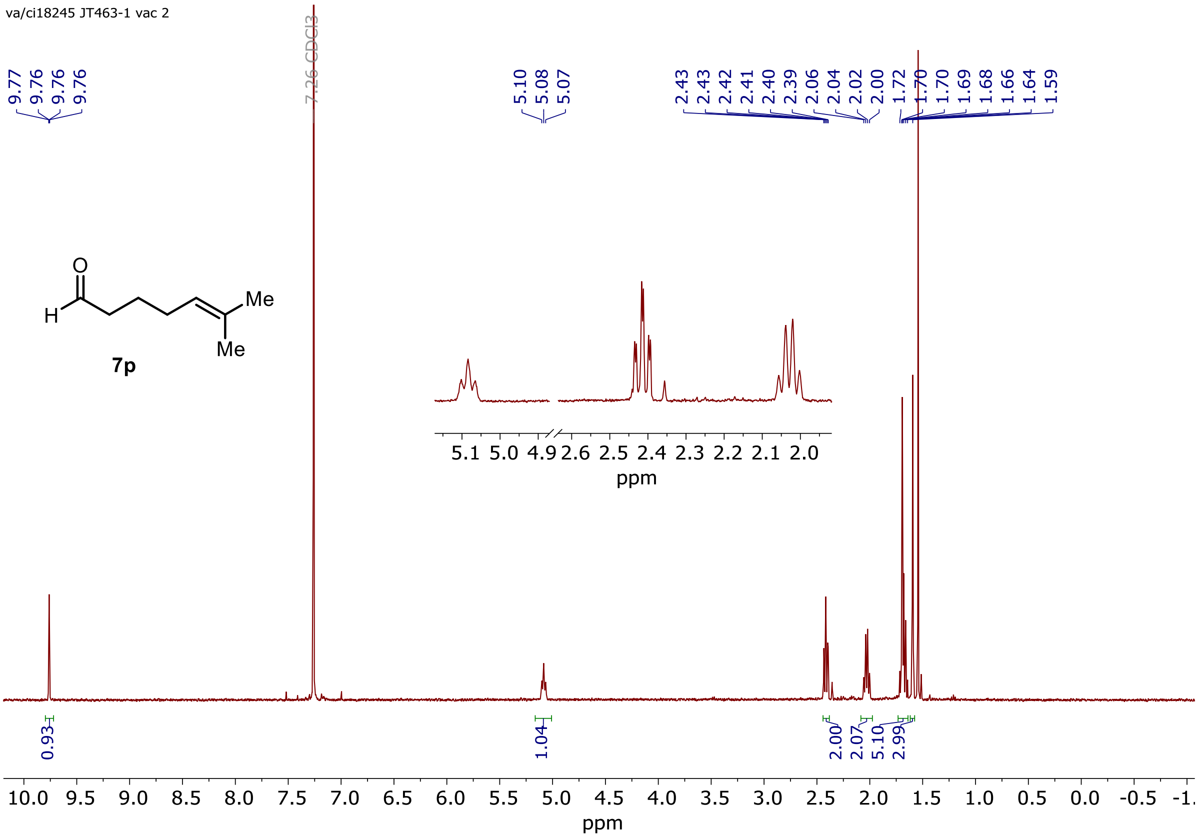

^{13}C NMR (126 MHz, $CDCl_3$) of **7p**

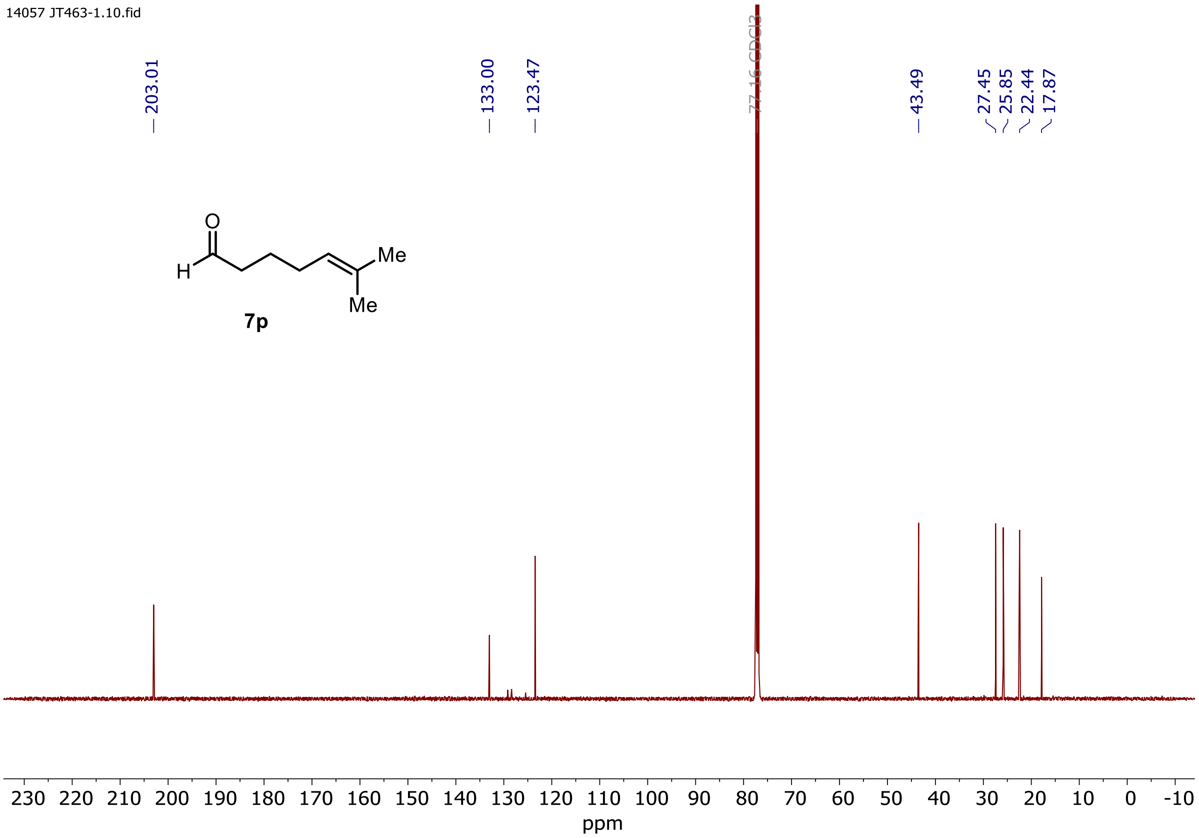

^{1}H NMR (400 MHz, $CDCl_3$) of **4-Phenylbutanal** (*see procedure*)

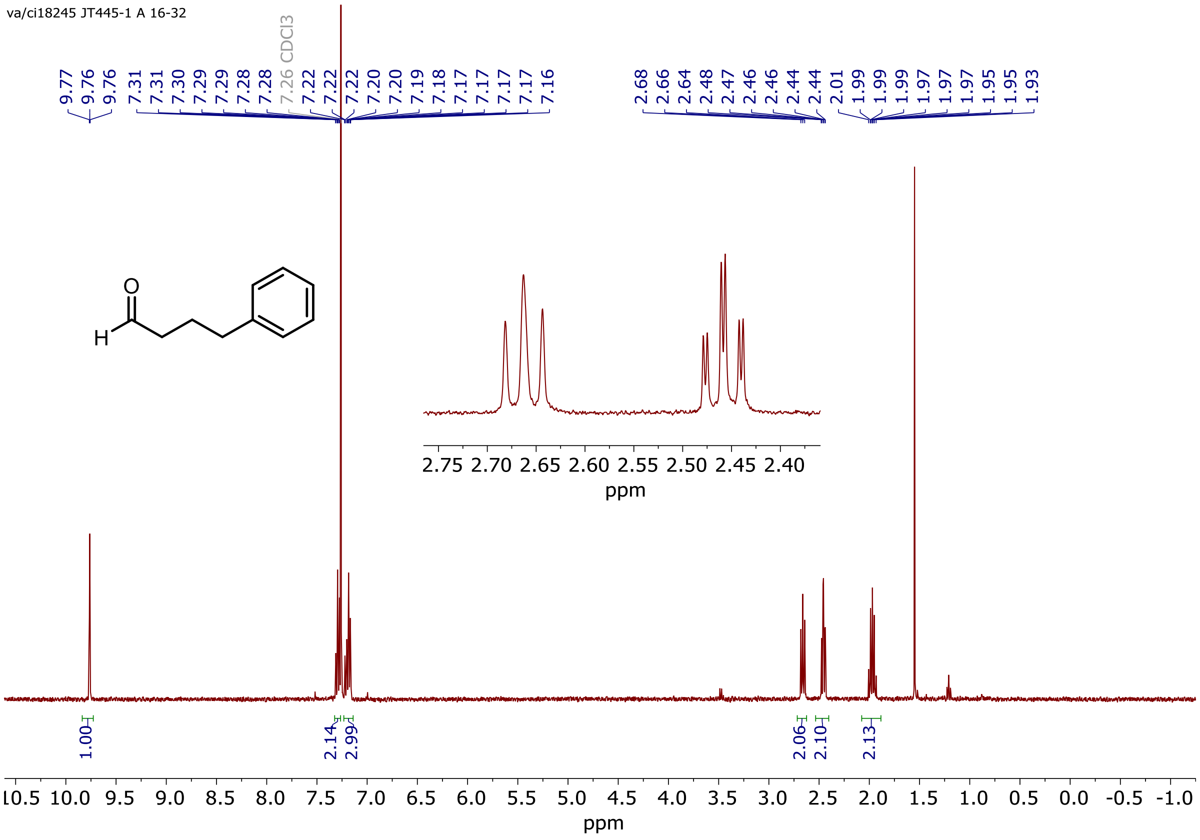

^{13}C NMR (126 MHz, $CDCl_3$) of **4-Phenylbutanal**

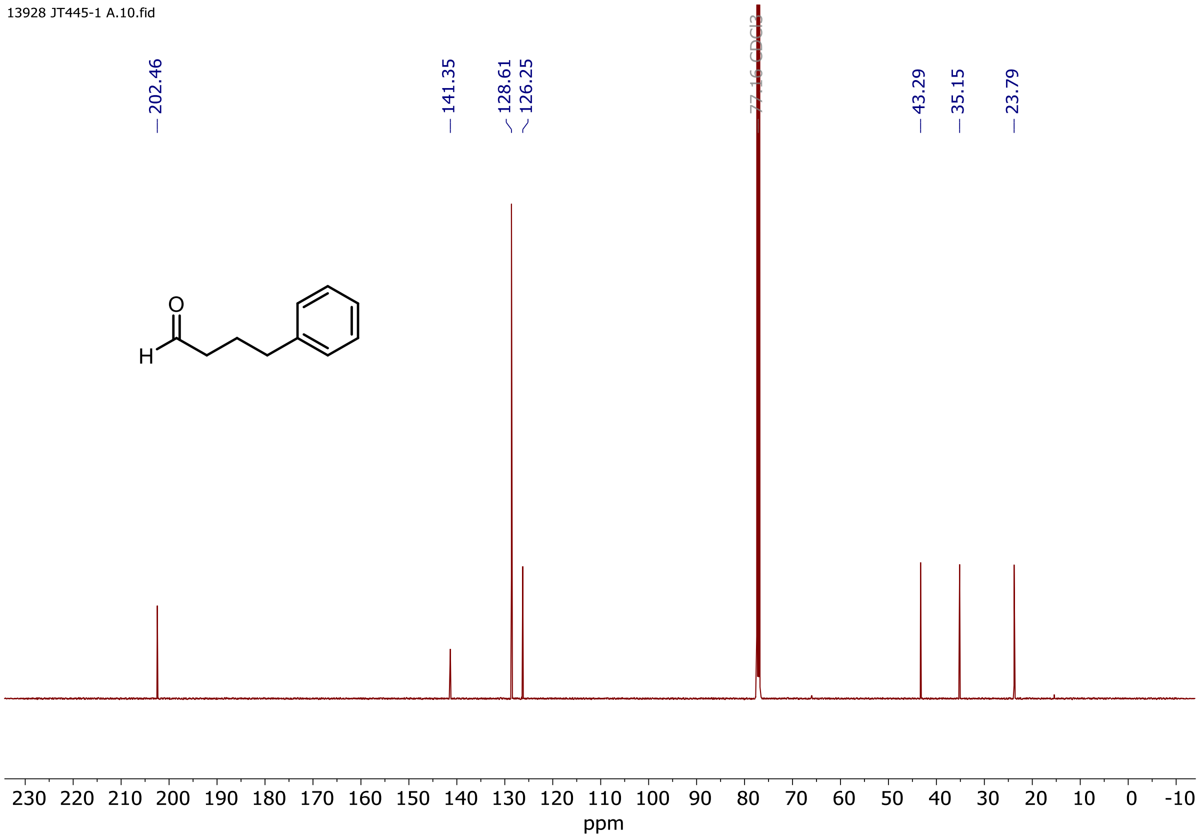

^{1}H NMR (500 MHz, $CDCl_3$) of **1-(1-Azabicyclo[1.1.0]butan-3-yl)-3-phenylpropan-1-one** (*see procedure*)

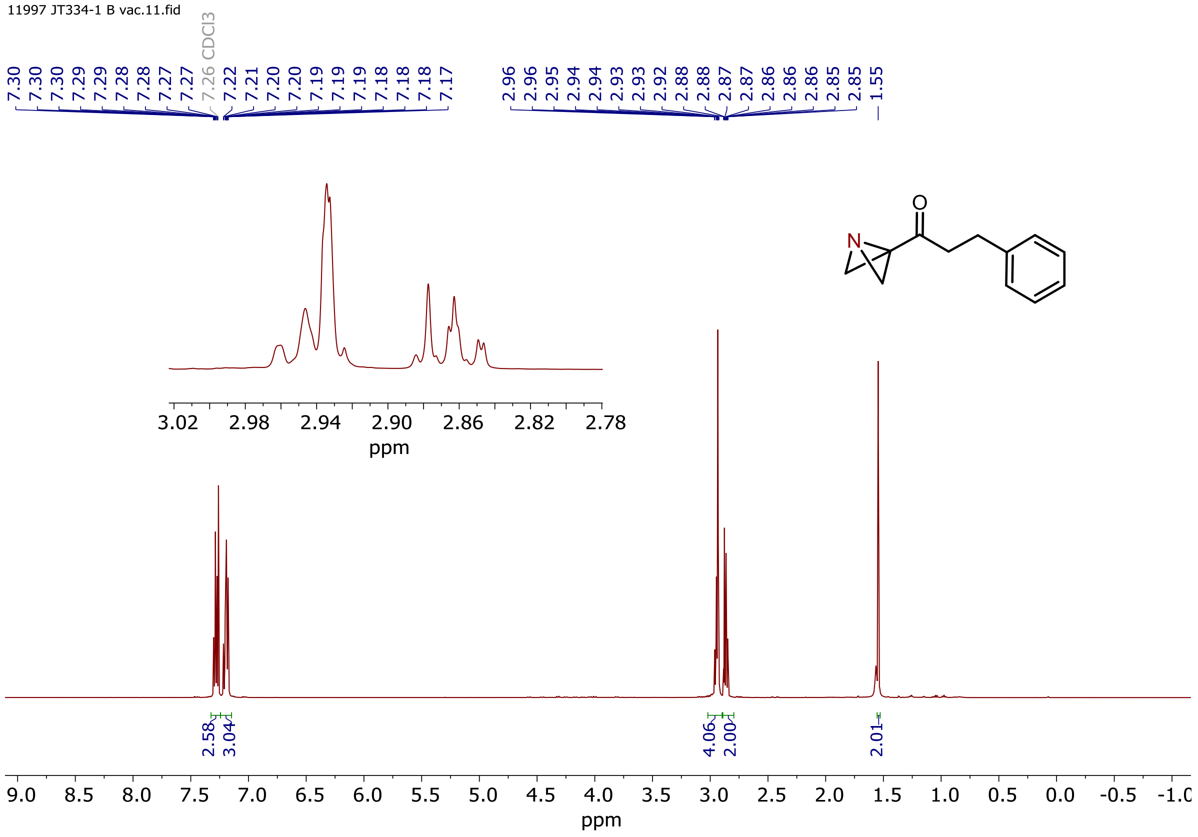

^{13}C NMR (126 MHz, $CDCl_3$) of **1-(1-Azabicyclo[1.1.0]butan-3-yl)-3-phenylpropan-1-one**

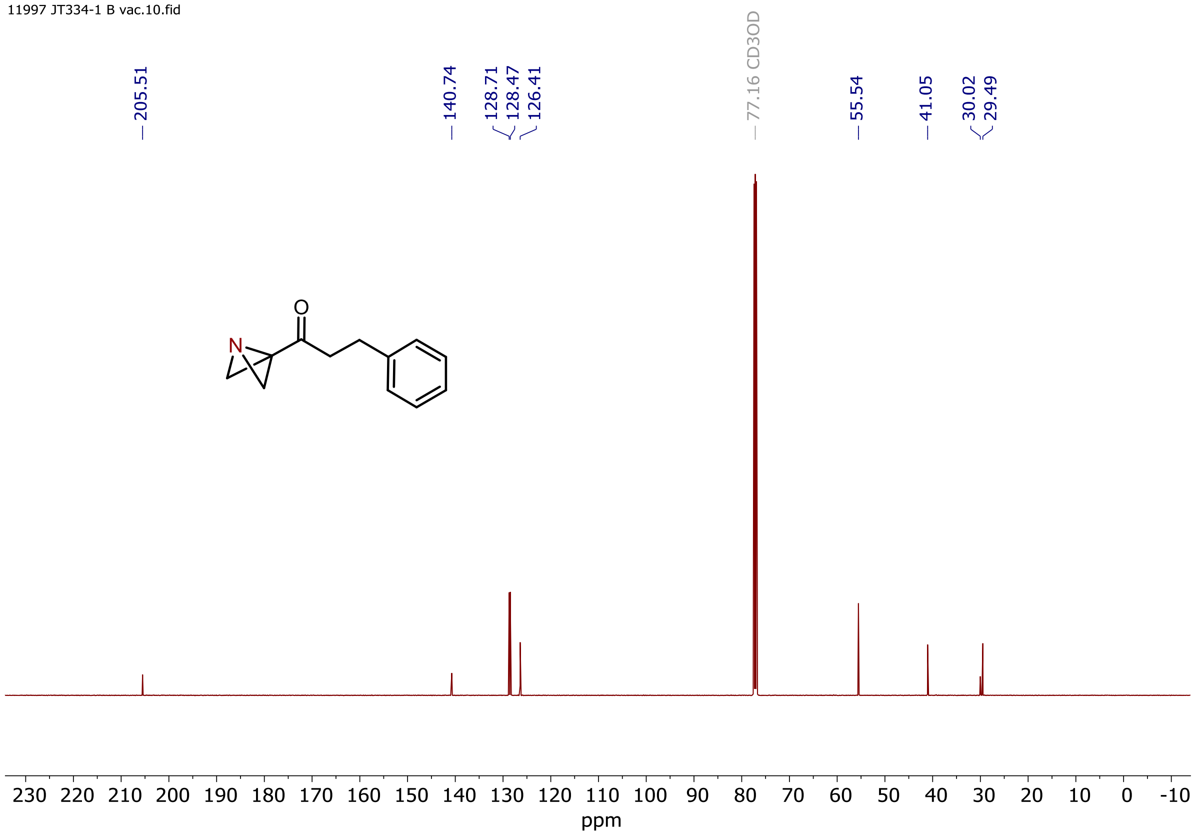

^{1}H NMR (500 MHz, $CDCl_3$) of **2z** (*see procedure*)

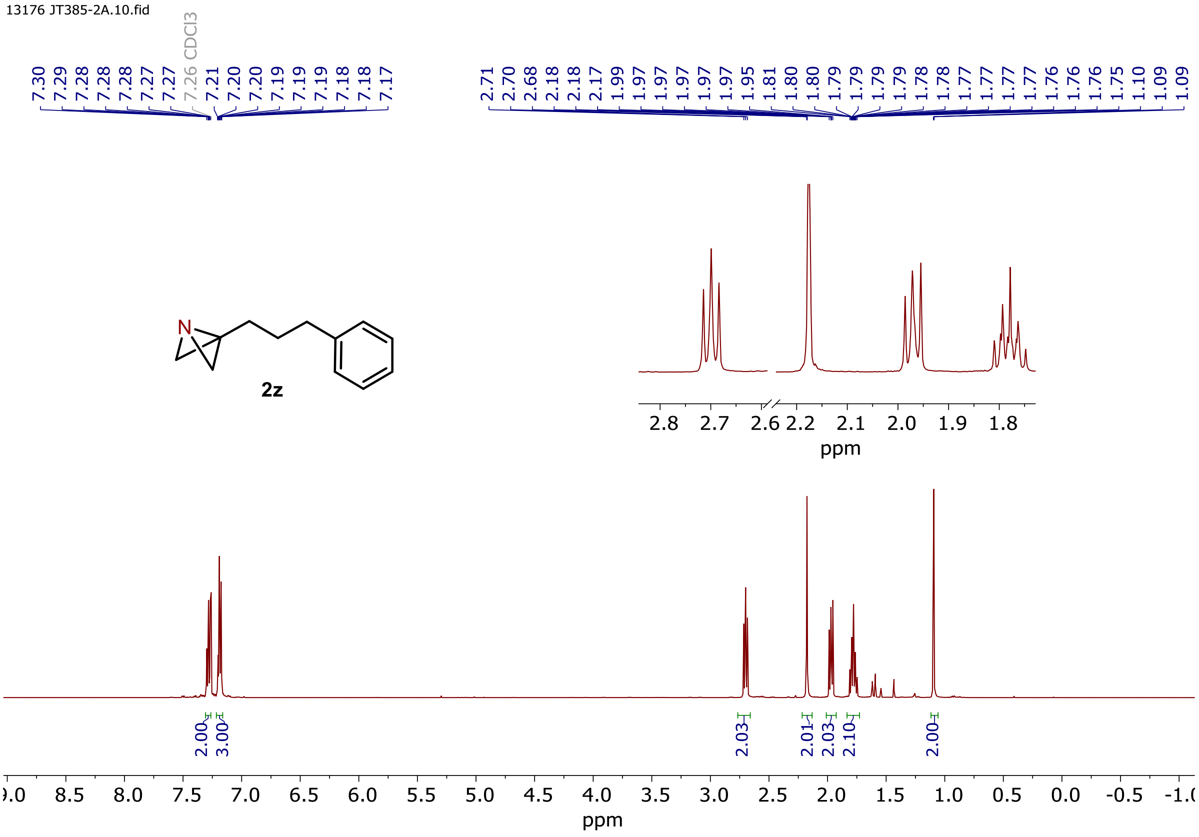

^{13}C NMR (126 MHz, $CDCl_3$) of **2z**

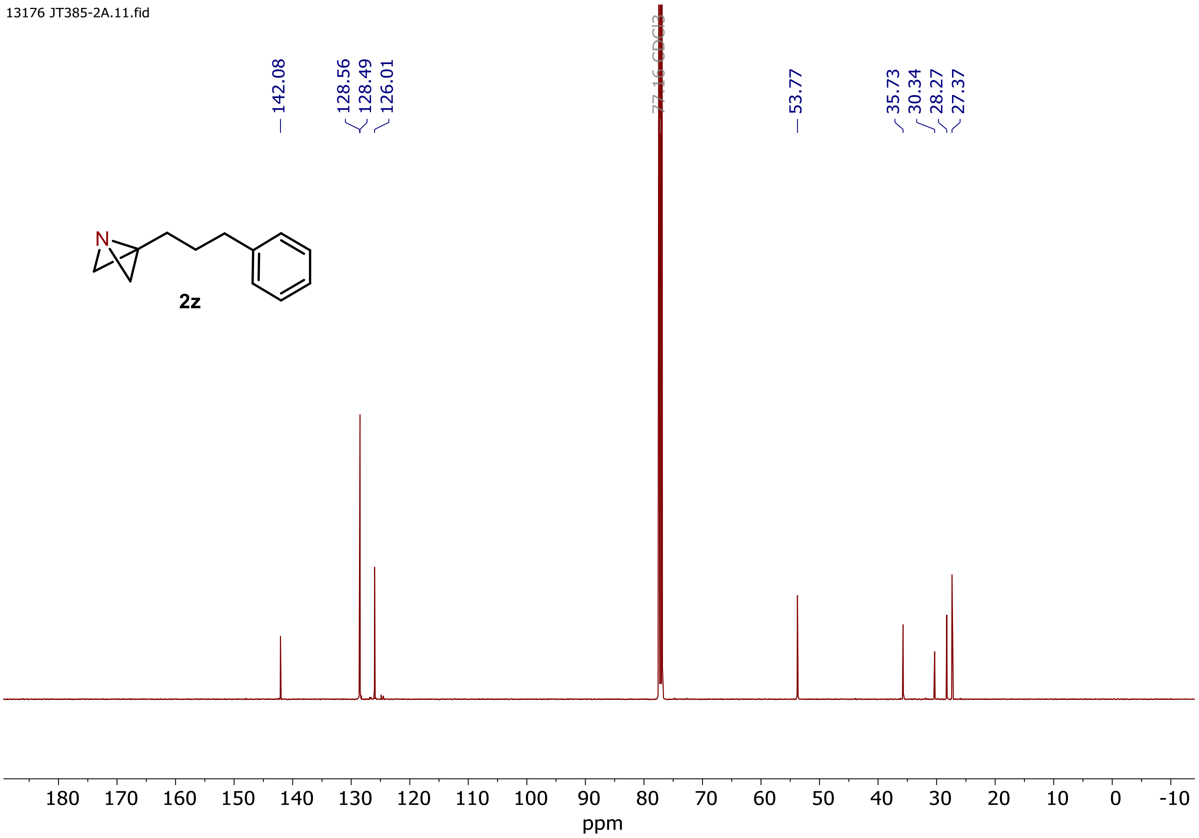

^{1}H NMR (500 MHz, $CDCl_3$) of **2a** (*see procedure*)

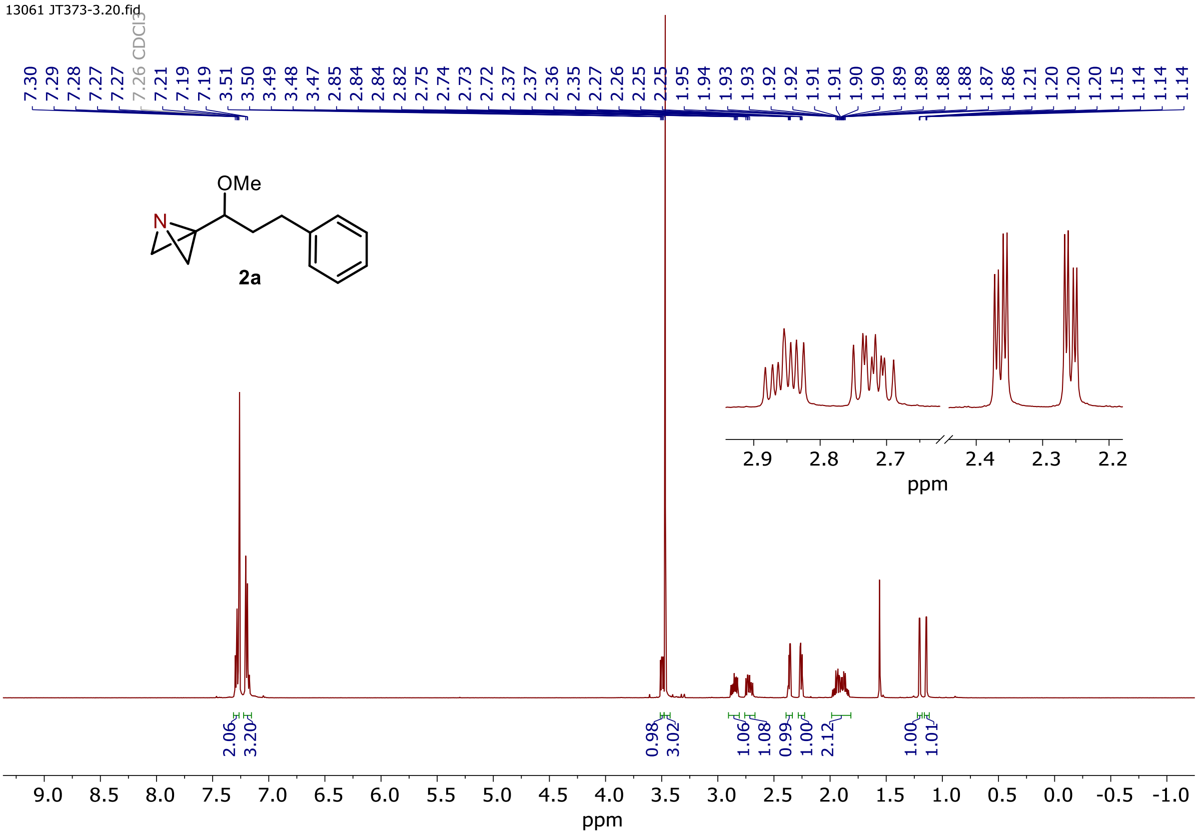

^{13}C NMR (126 MHz, $CDCl_3$) of **2a**

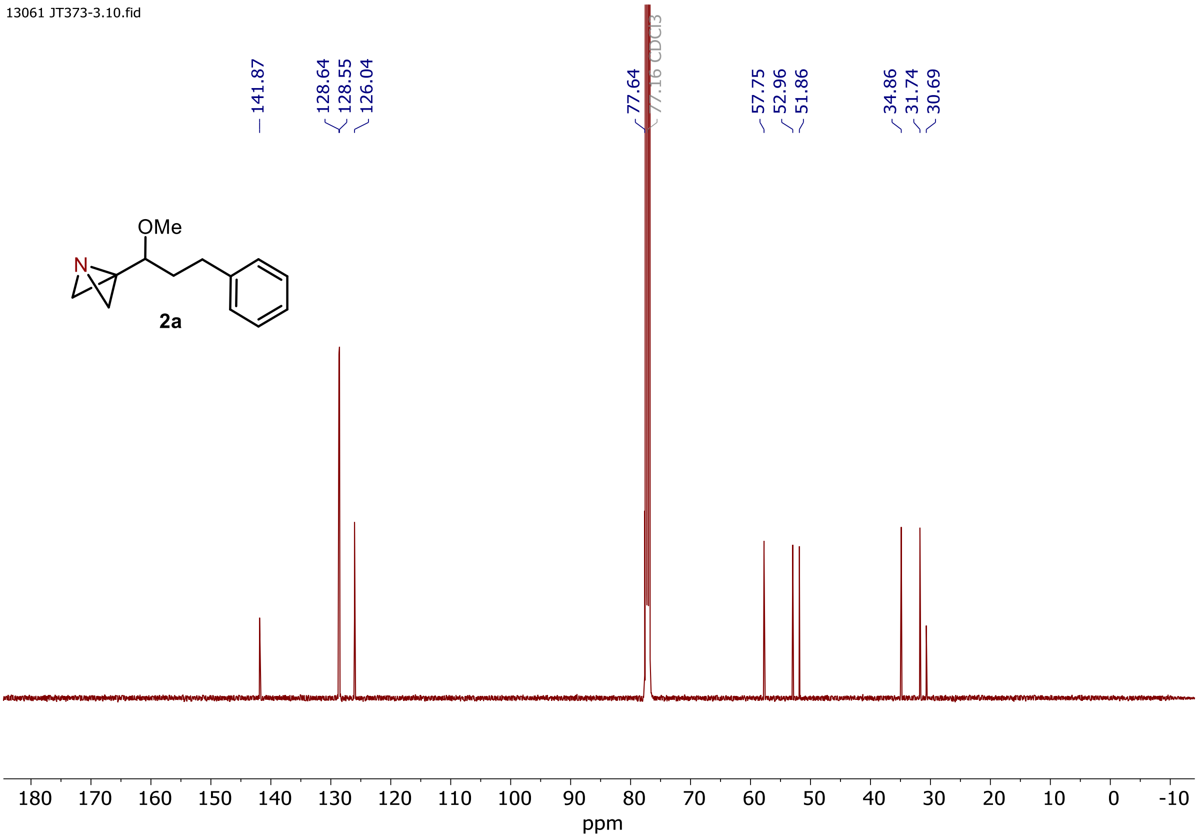

^{1}H NMR (400 MHz, $CDCl_3$) of **2b** (see procedure)

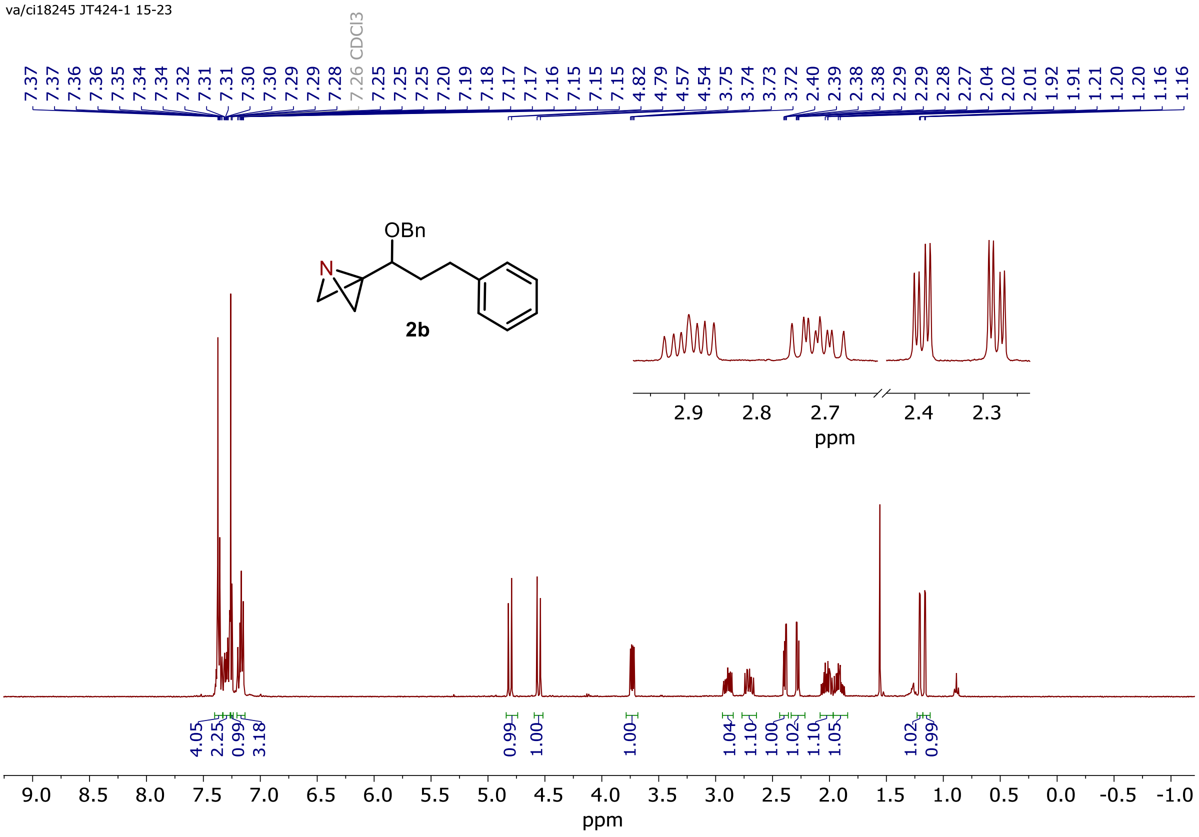

^{13}C NMR (126 MHz, $CDCl_3$) of **2b**

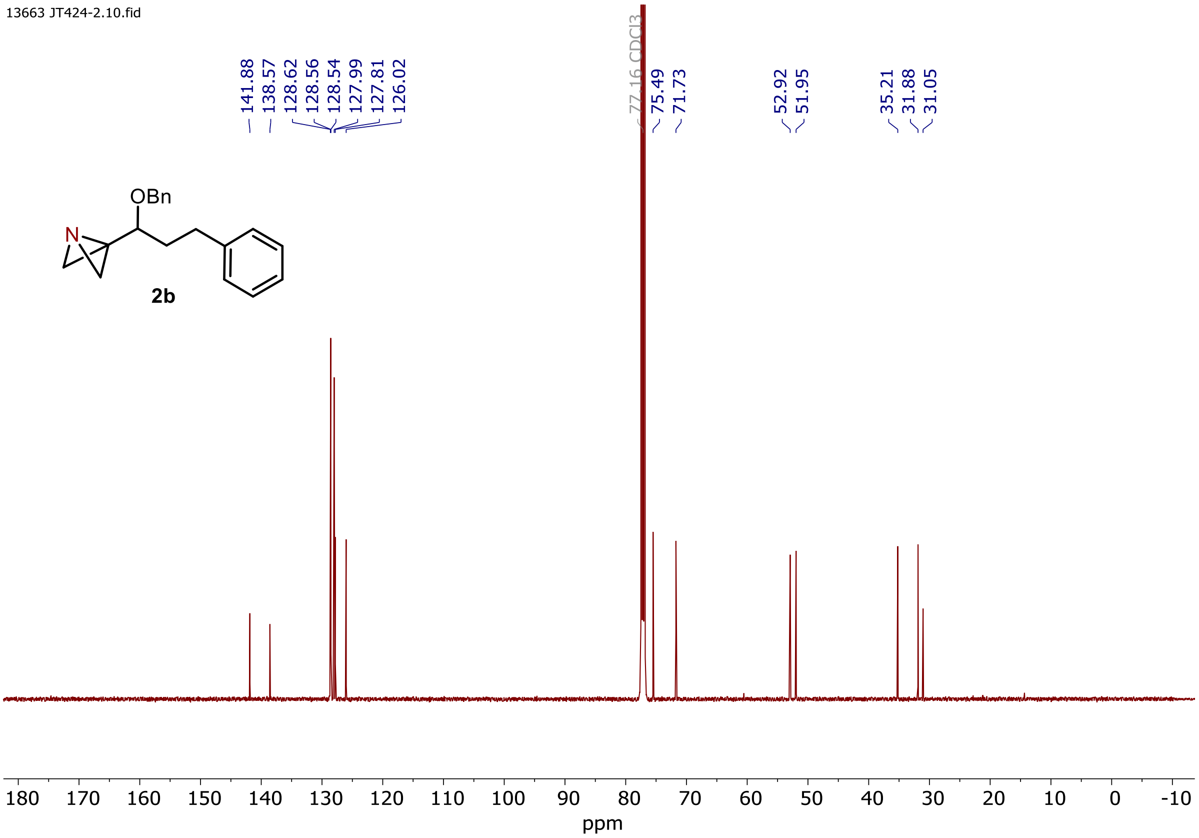

^{1}H NMR (400 MHz, $CDCl_3$) of **2c** (*see procedure*)

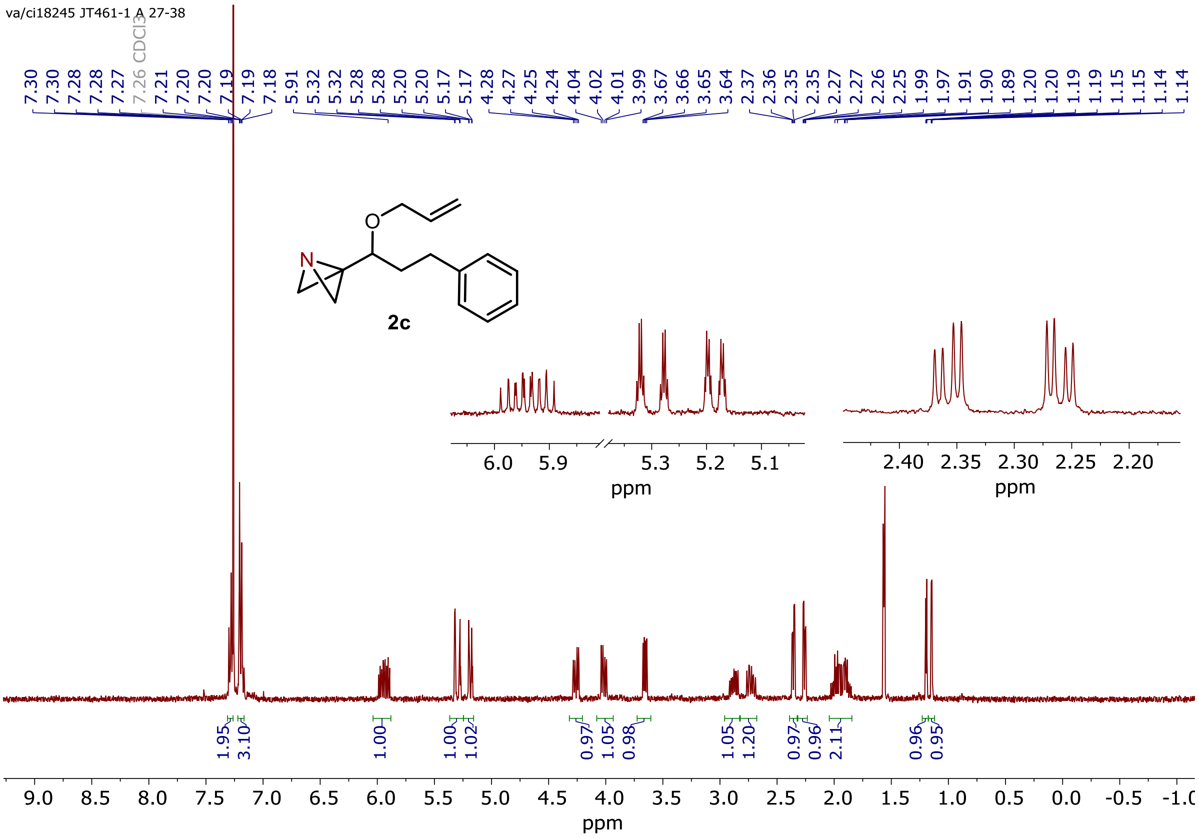

^{13}C NMR (126 MHz, $CDCl_3$) of **2c**

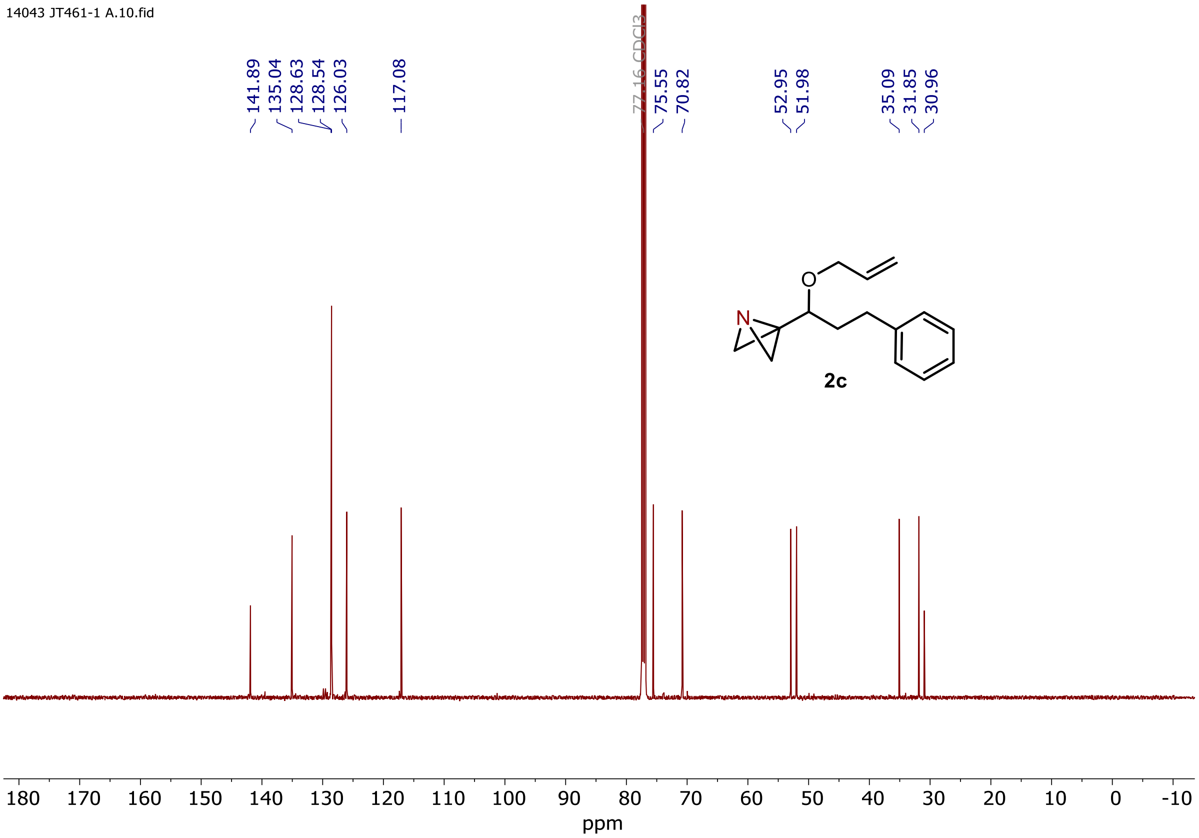

^{1}H NMR (400 MHz, $CDCl_3$) of **2d** (*see procedure*)

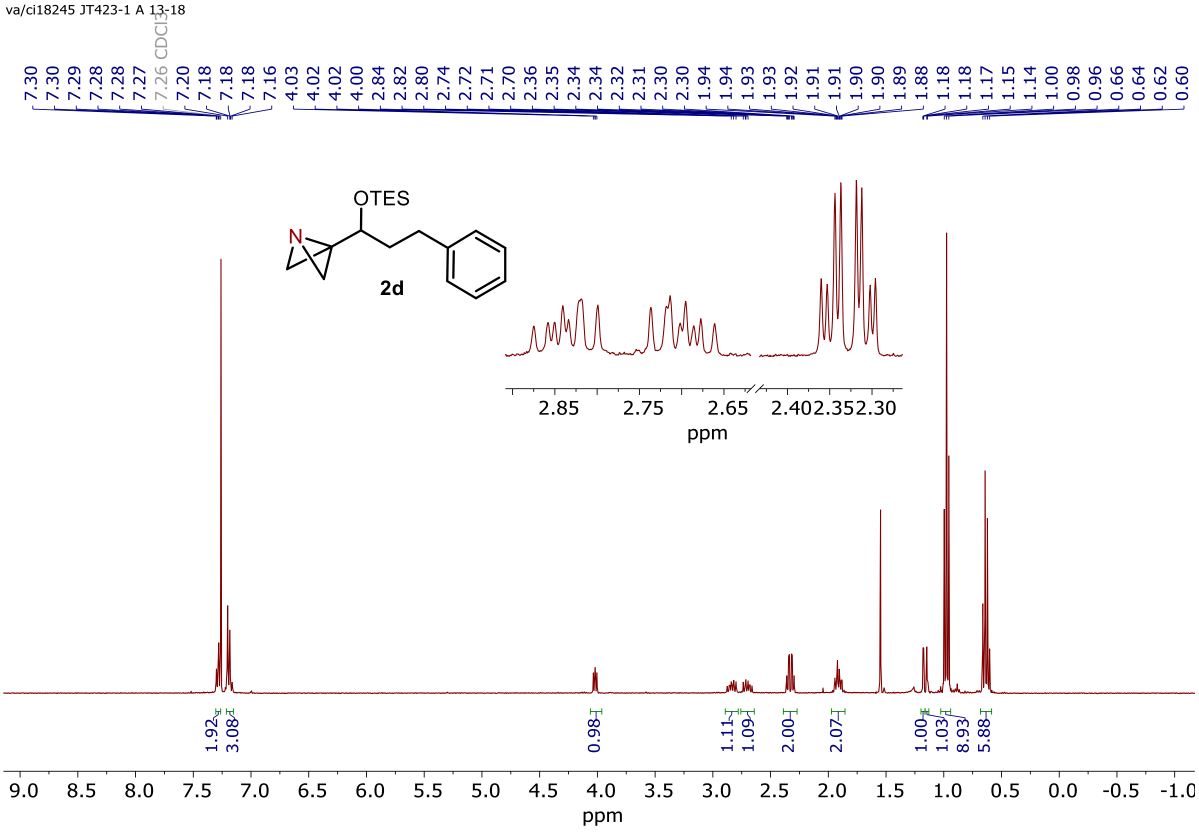

^{13}C NMR (126 MHz, $CDCl_3$) of **2d**

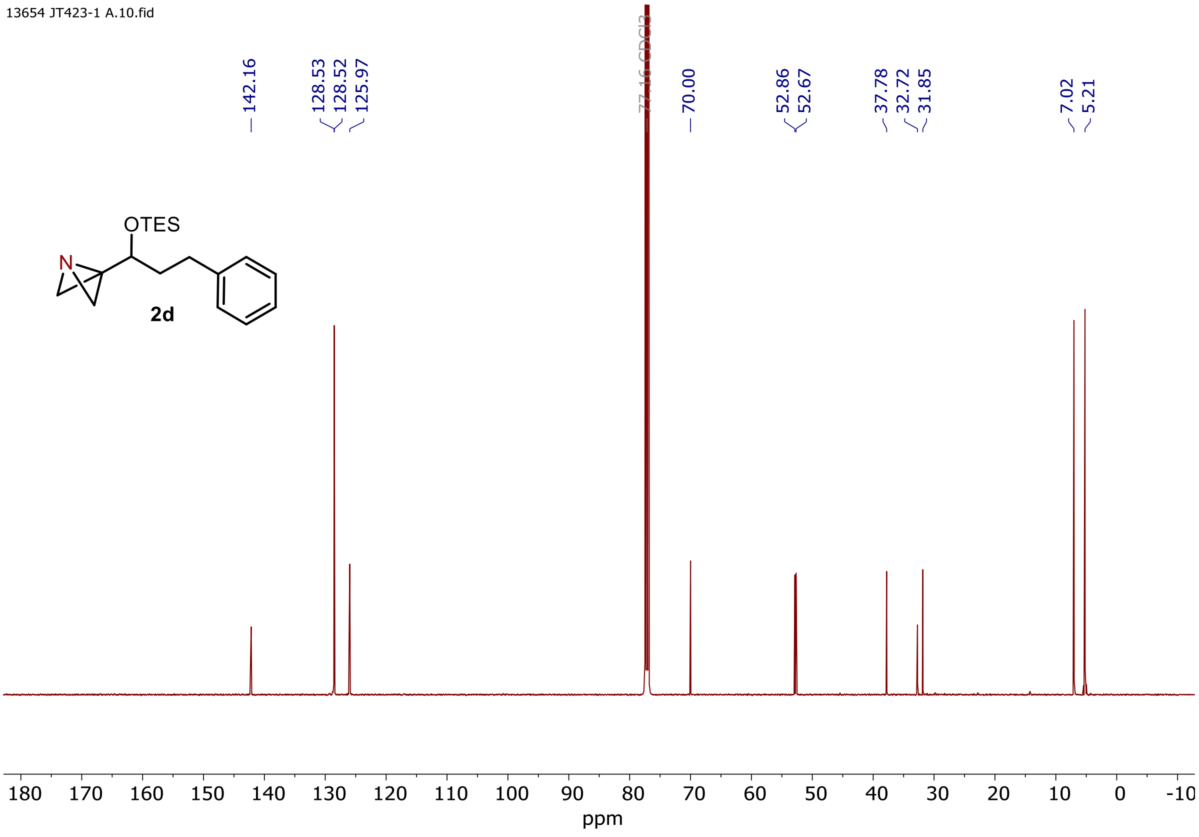

^{1}H NMR (400 MHz, $CDCl_3$) of **2e** (*see procedure*)

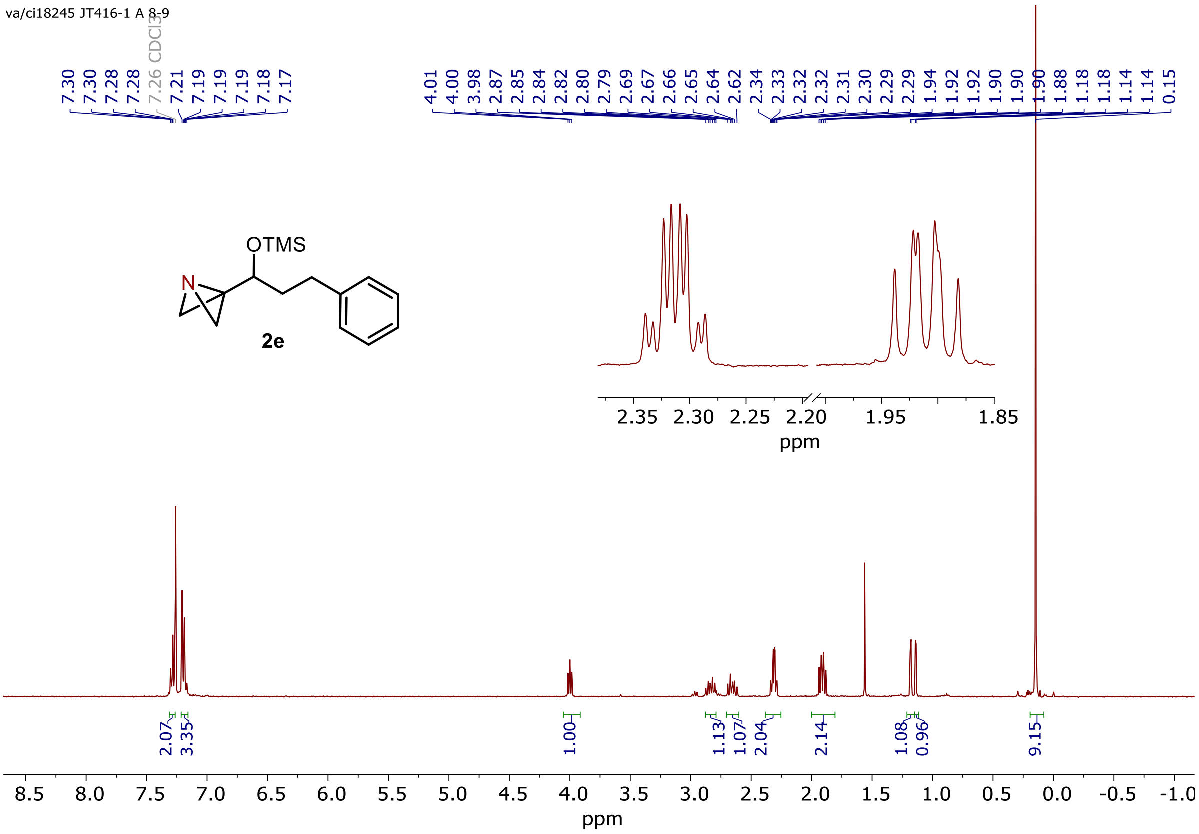

^{13}C NMR (126 MHz, $CDCl_3$) of **2e**

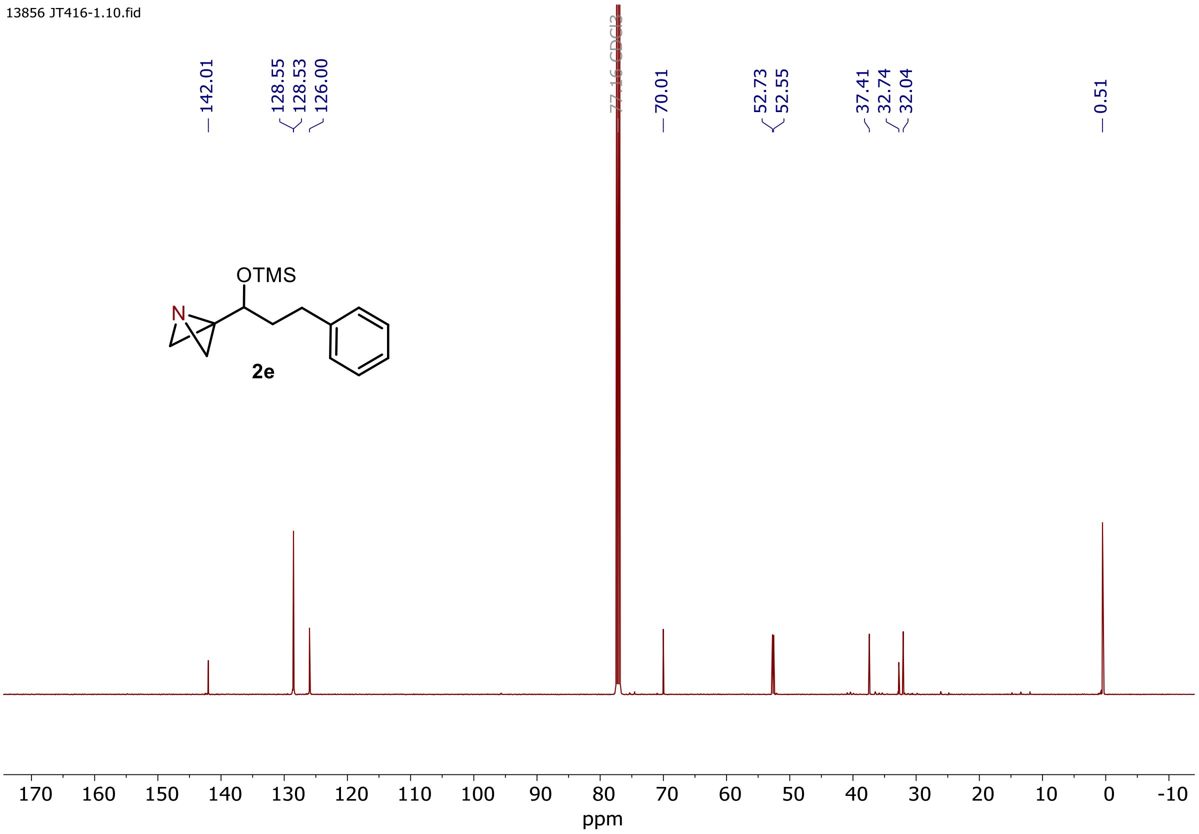

^{1}H NMR (400 MHz, $CDCl_3$) of **2f** (*see procedure*)

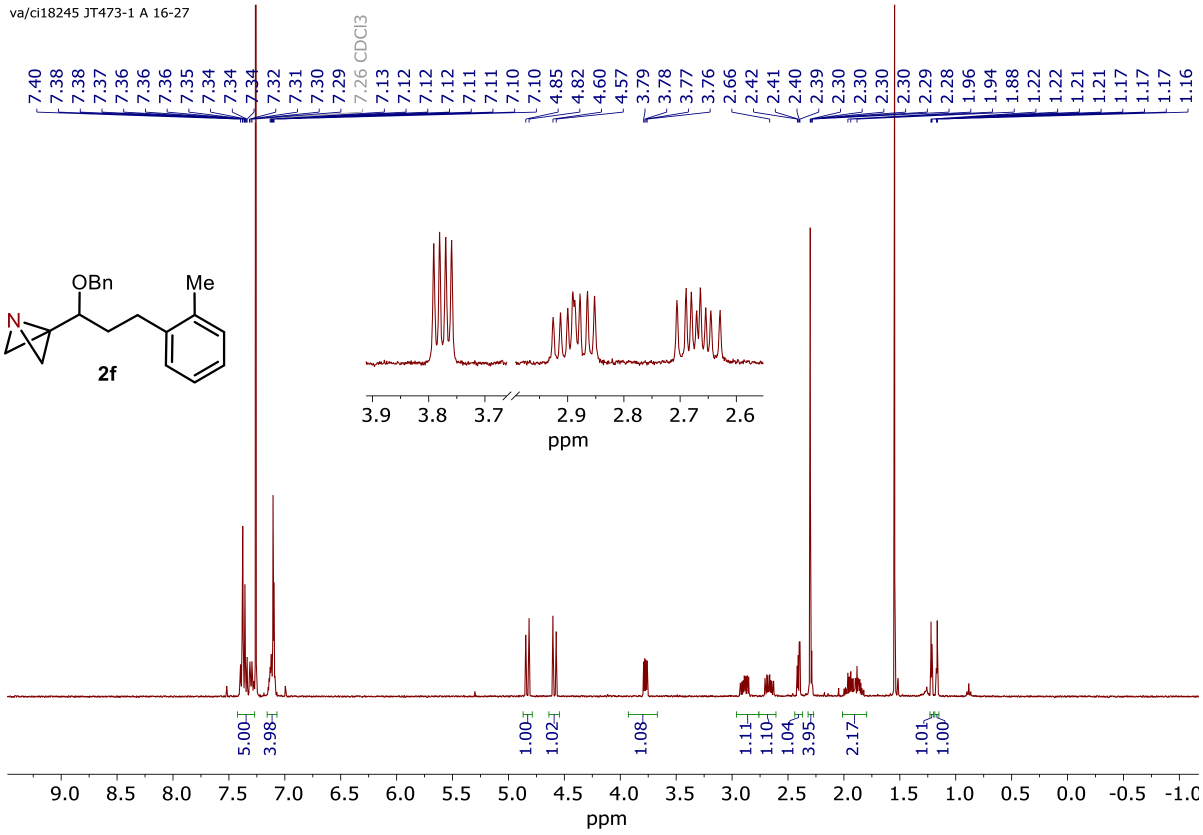

^{13}C NMR (126 MHz, $CDCl_3$) of **2f**

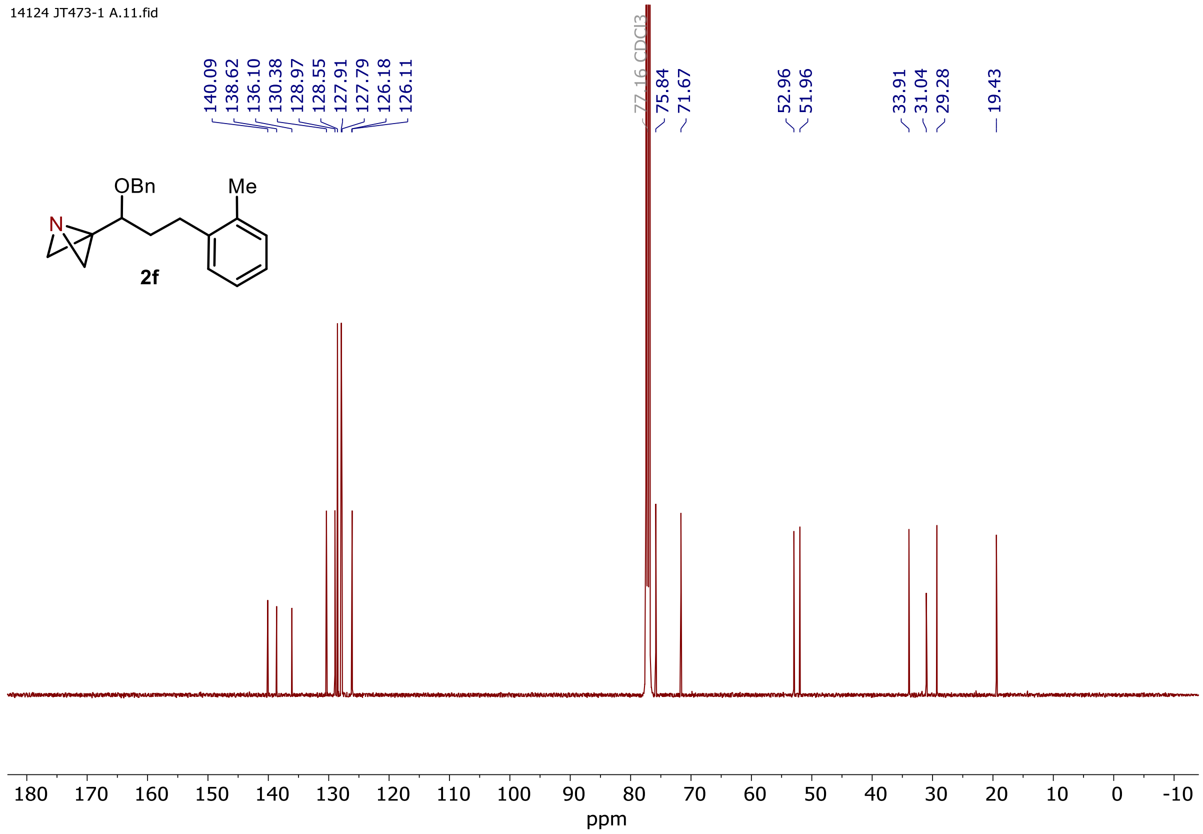

^{1}H NMR (400 MHz, $CDCl_3$) of **2g** (*see procedure*)

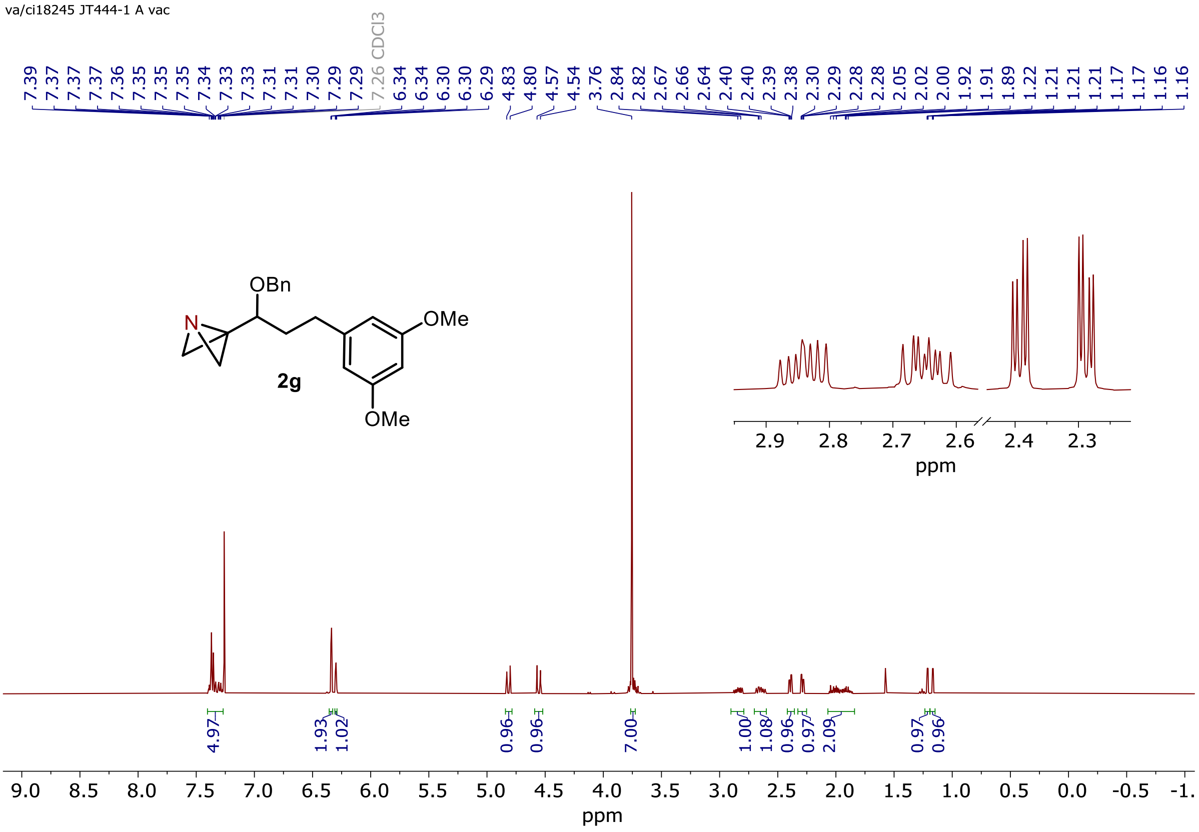

^{13}C NMR (126 MHz, $CDCl_3$) of **2g**

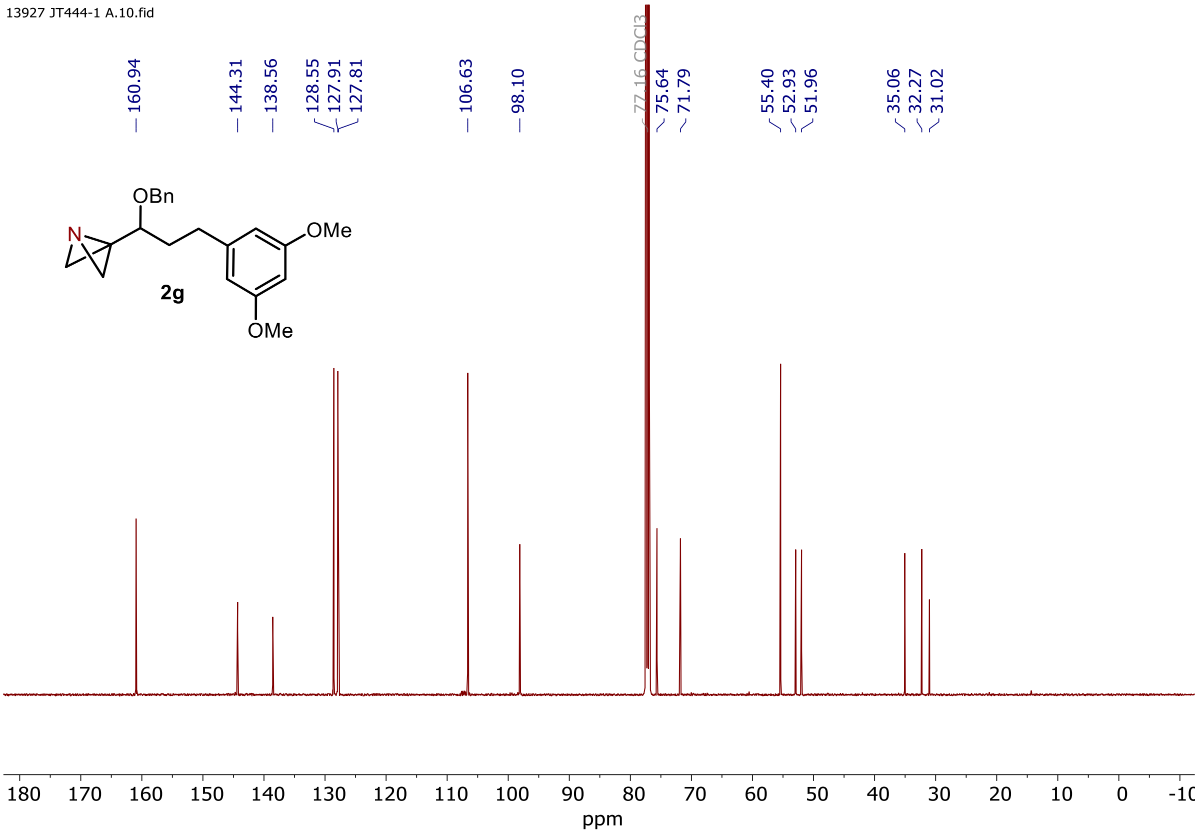

^{1}H NMR (500 MHz, $CDCl_3$) of **2h** (*see procedure*)

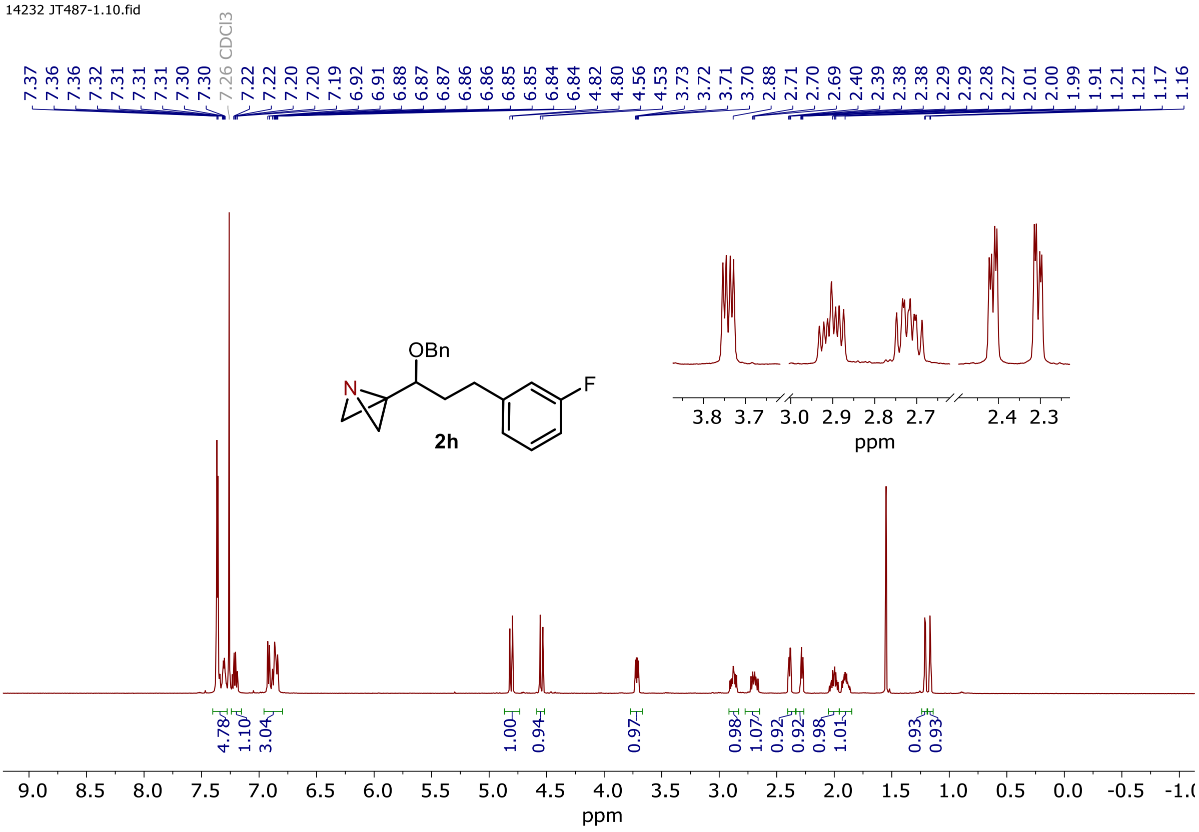

^{13}C NMR (126 MHz, $CDCl_3$) of **2h**

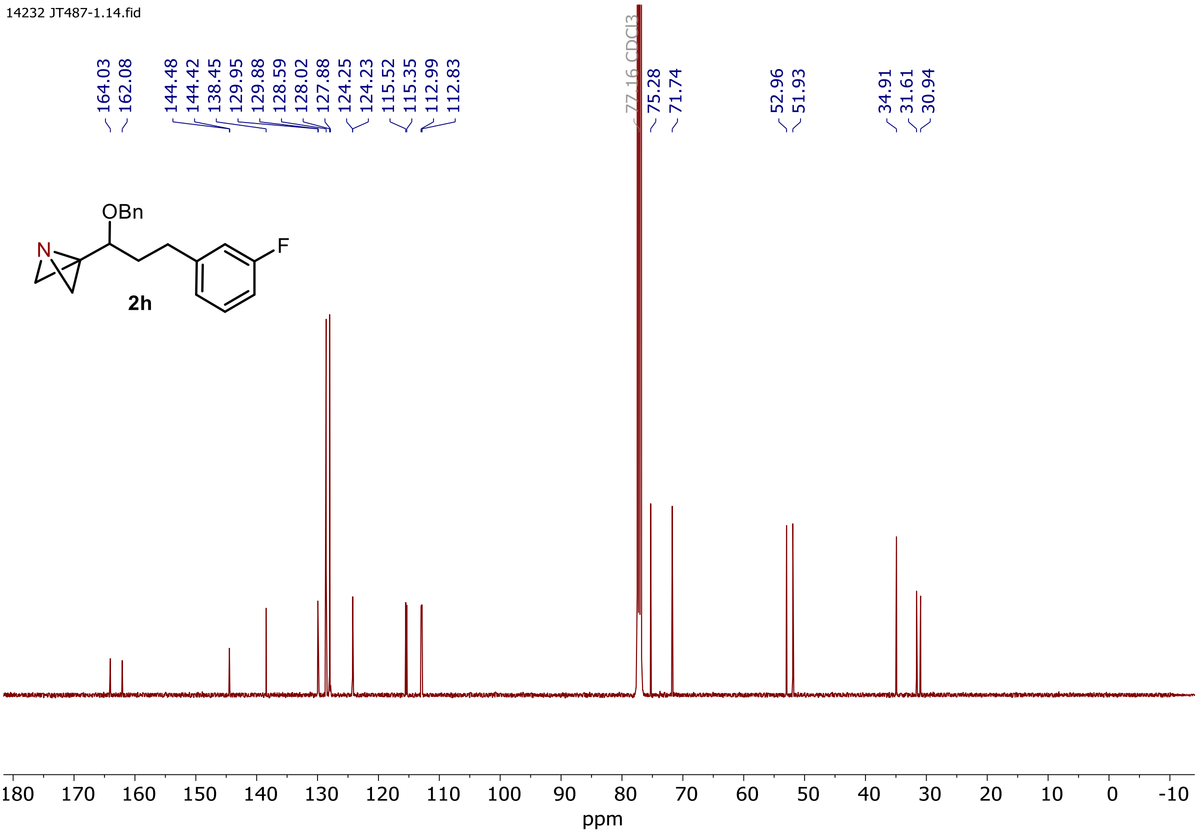

^{19}F NMR (376 MHz, $CDCl_3$) of **2h**

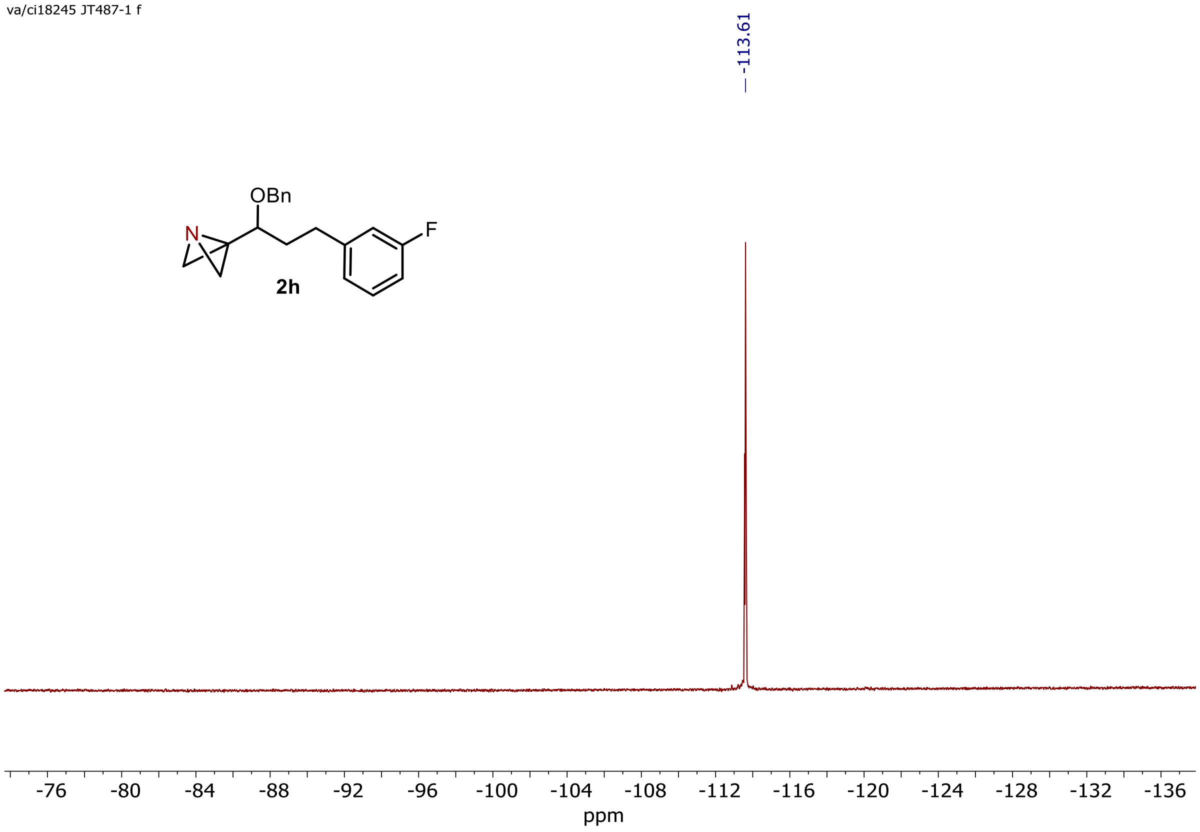

^{1}H NMR (400 MHz, $CDCl_3$) of **2i** (*see procedure*)

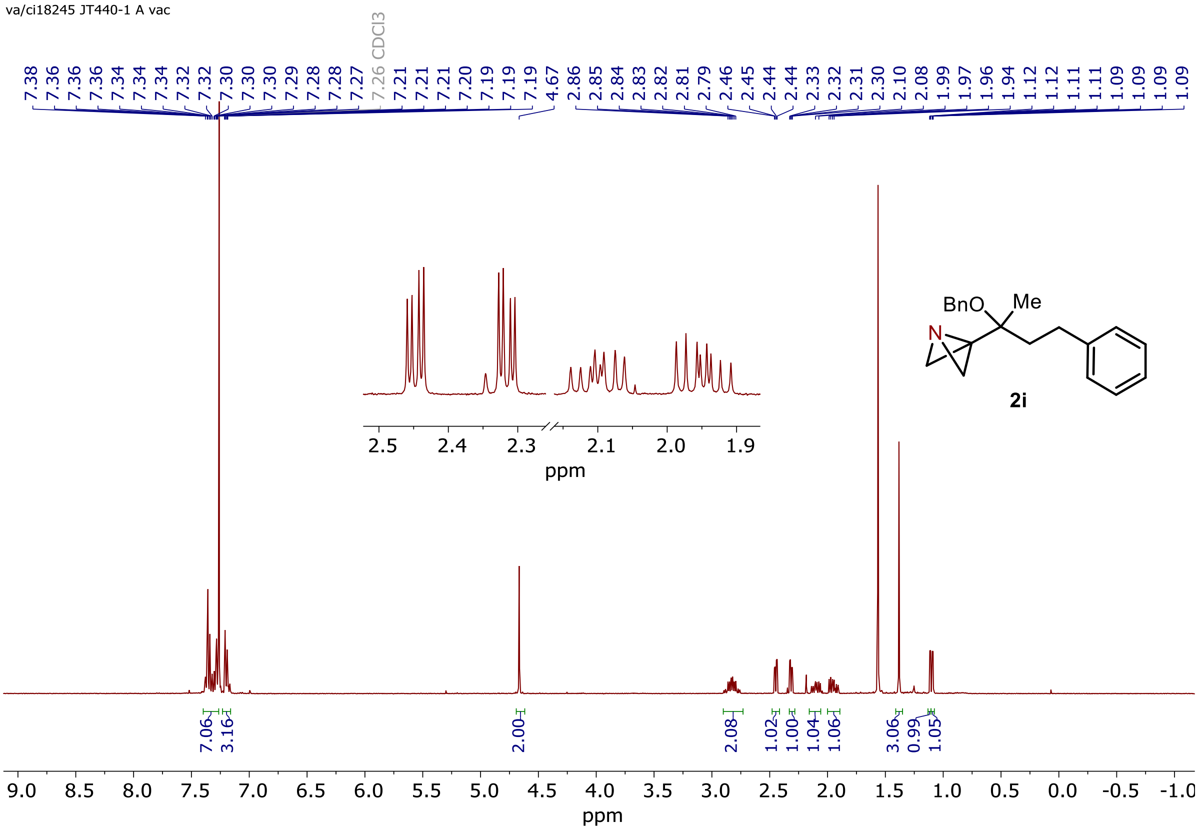

^{13}C NMR (126 MHz, $CDCl_3$) of **2i**

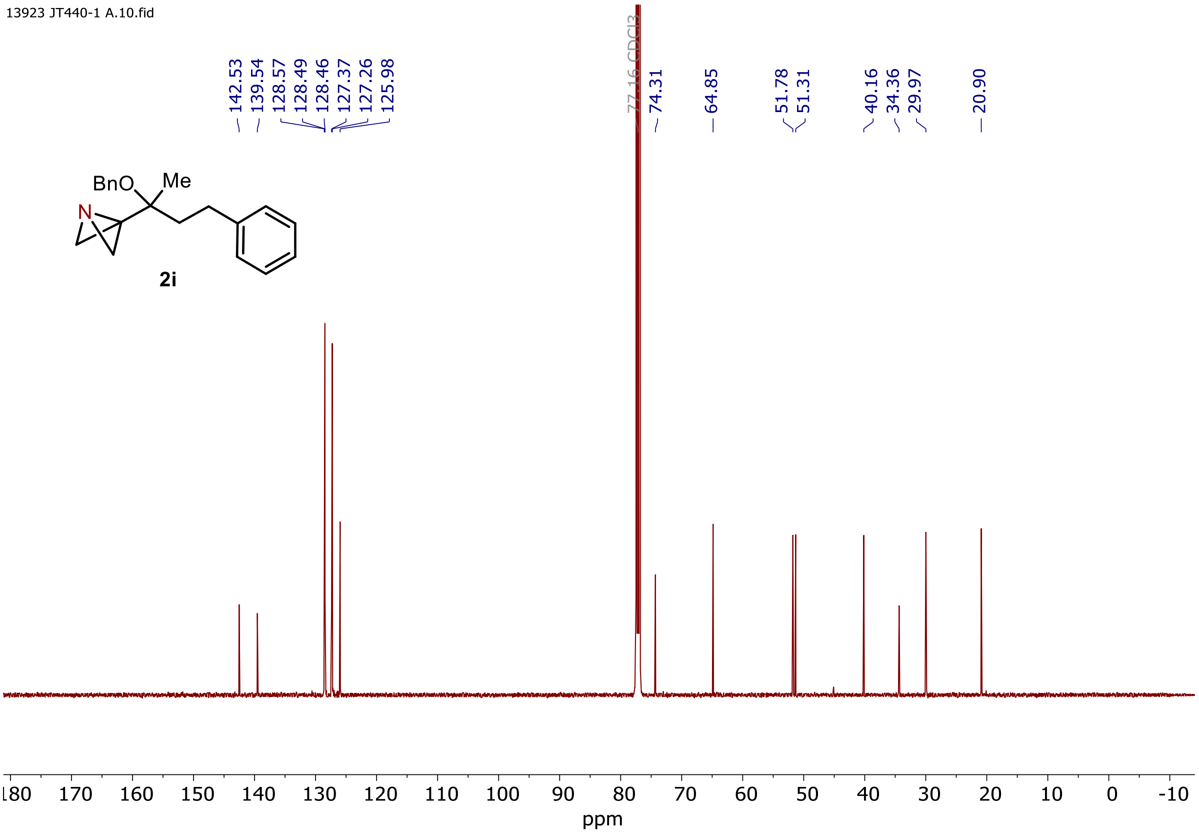

^{1}H NMR (400 MHz, $CDCl_3$) of **2j** (*see procedure*)

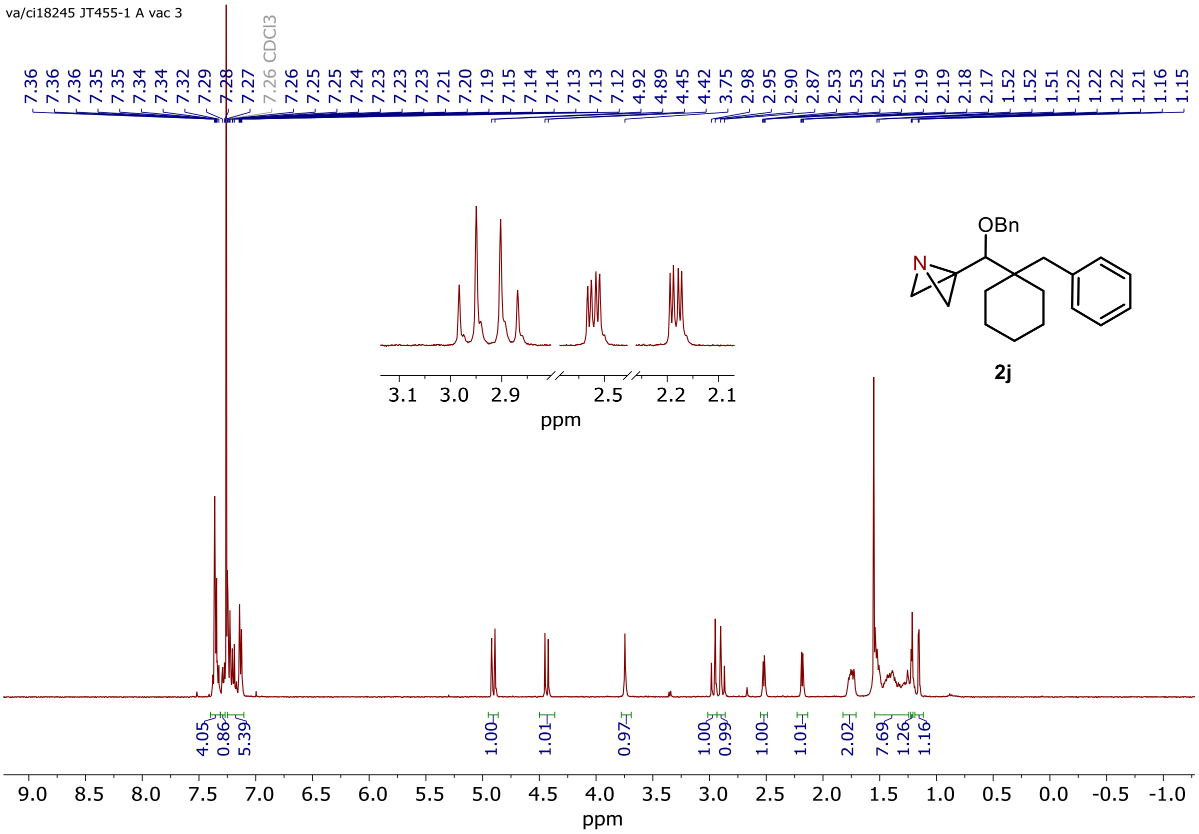

^{13}C NMR (126 MHz, $CDCl_3$) of **2j**

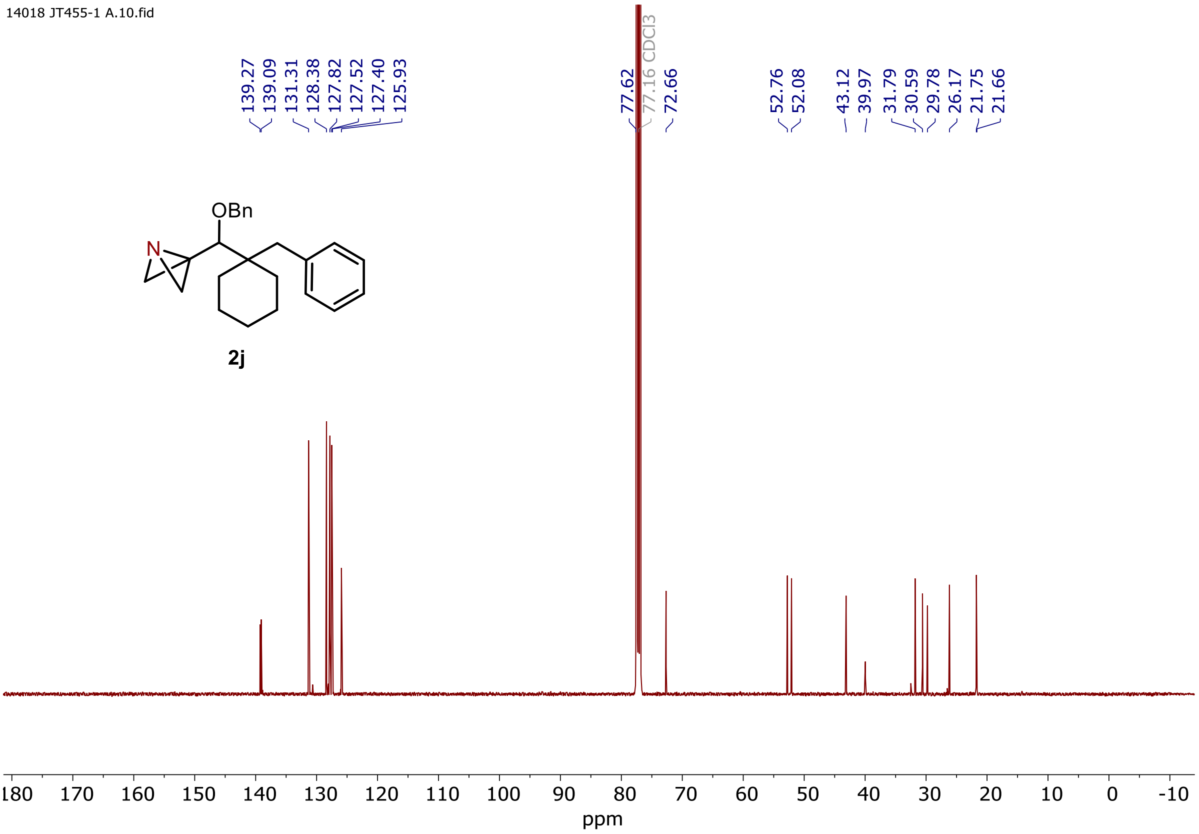

^{1}H NMR (500 MHz, $CDCl_3$) of **2k** (*see procedure*)

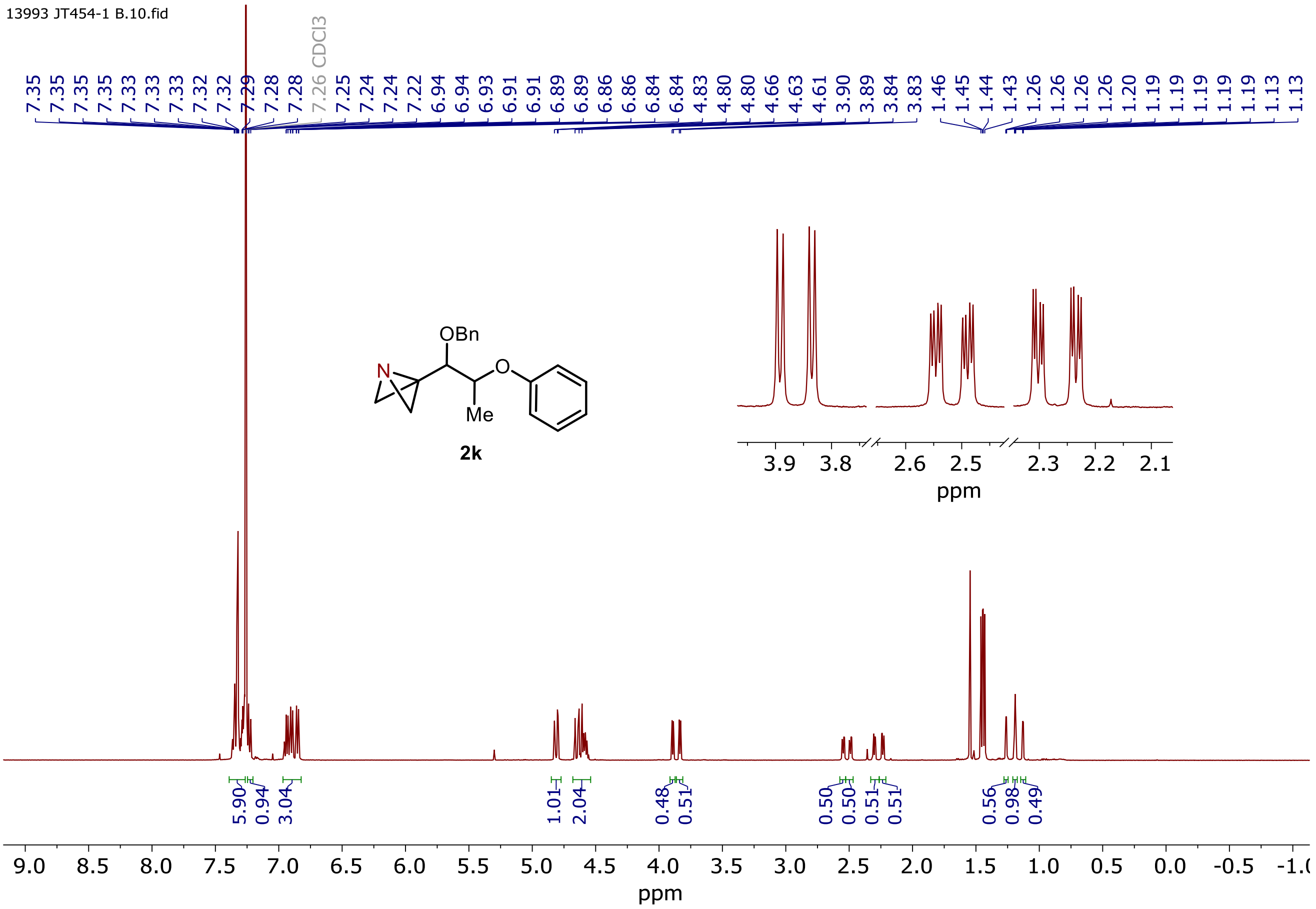

^{13}C NMR (126 MHz, $CDCl_3$) of **2k**

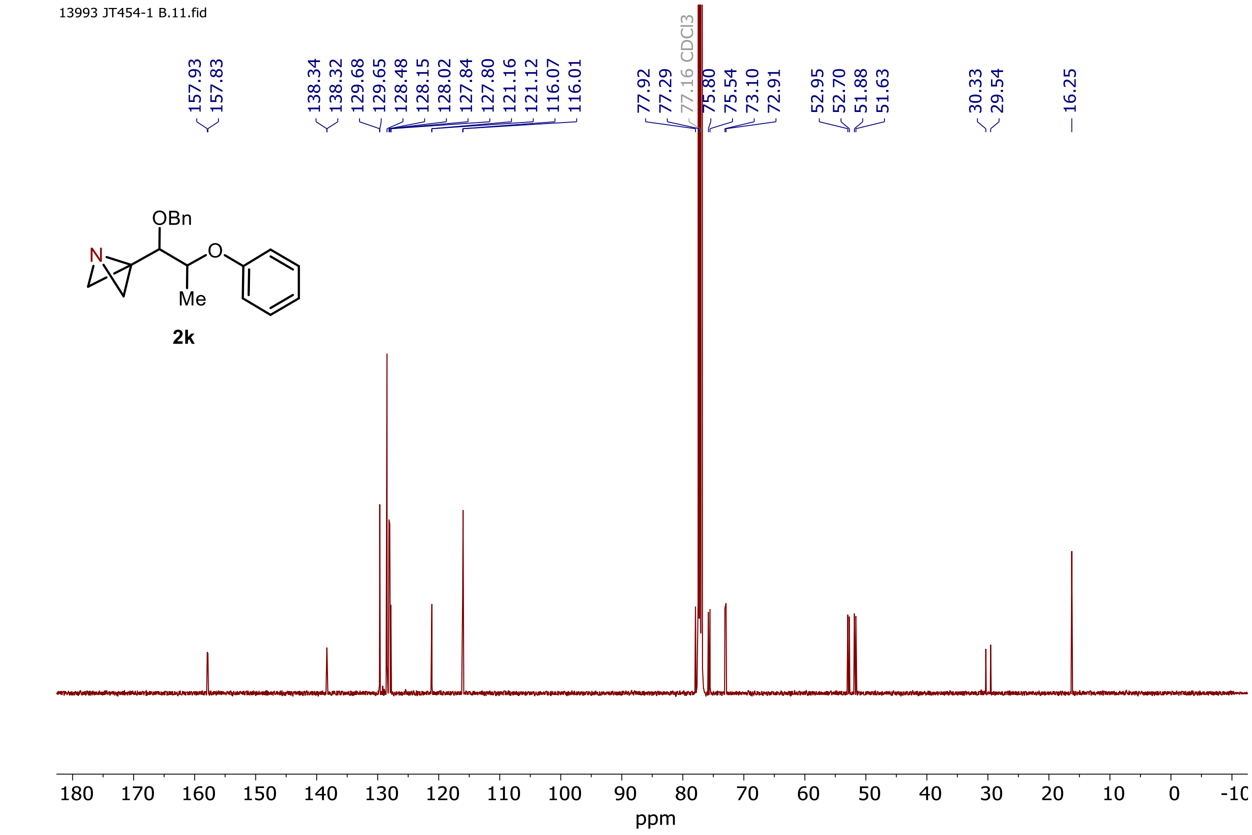

^{1}H NMR (500 MHz, $CDCl_3$) of **2l** (*see procedure*)

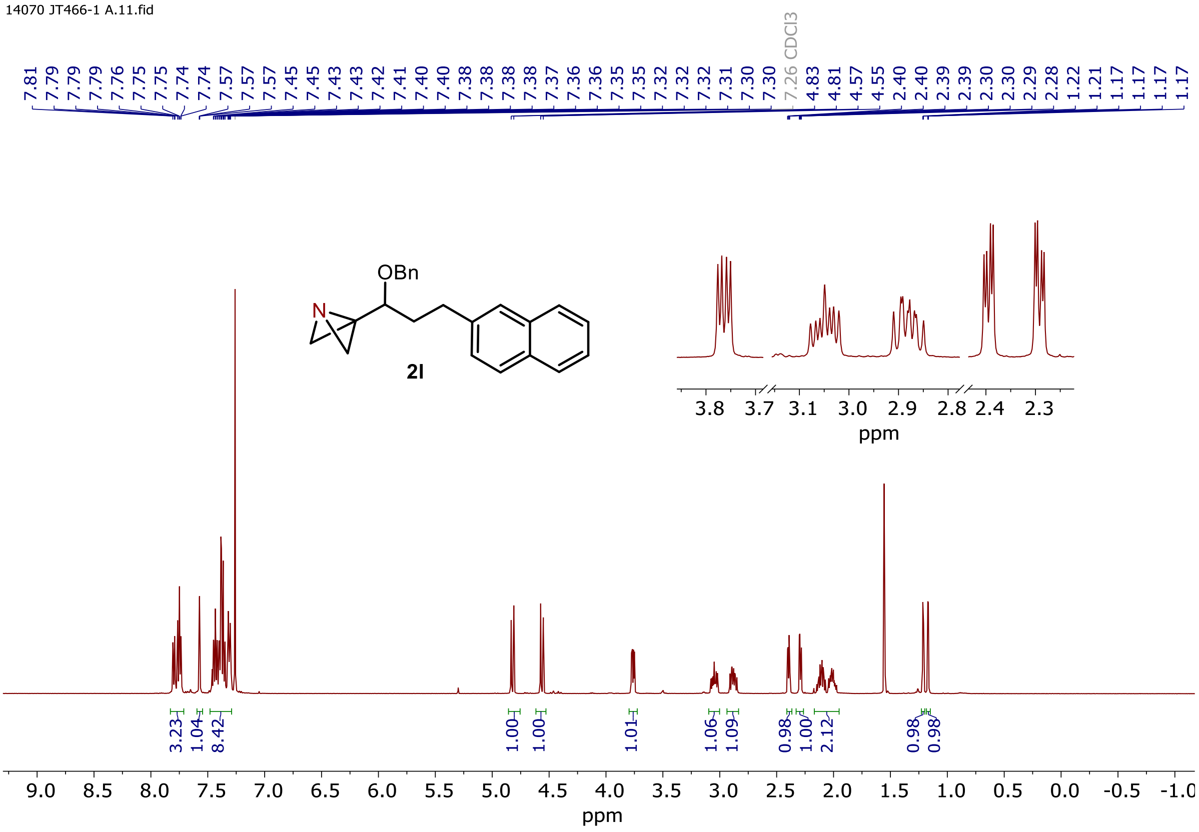

^{13}C NMR (126 MHz, $CDCl_3$) of **2l**

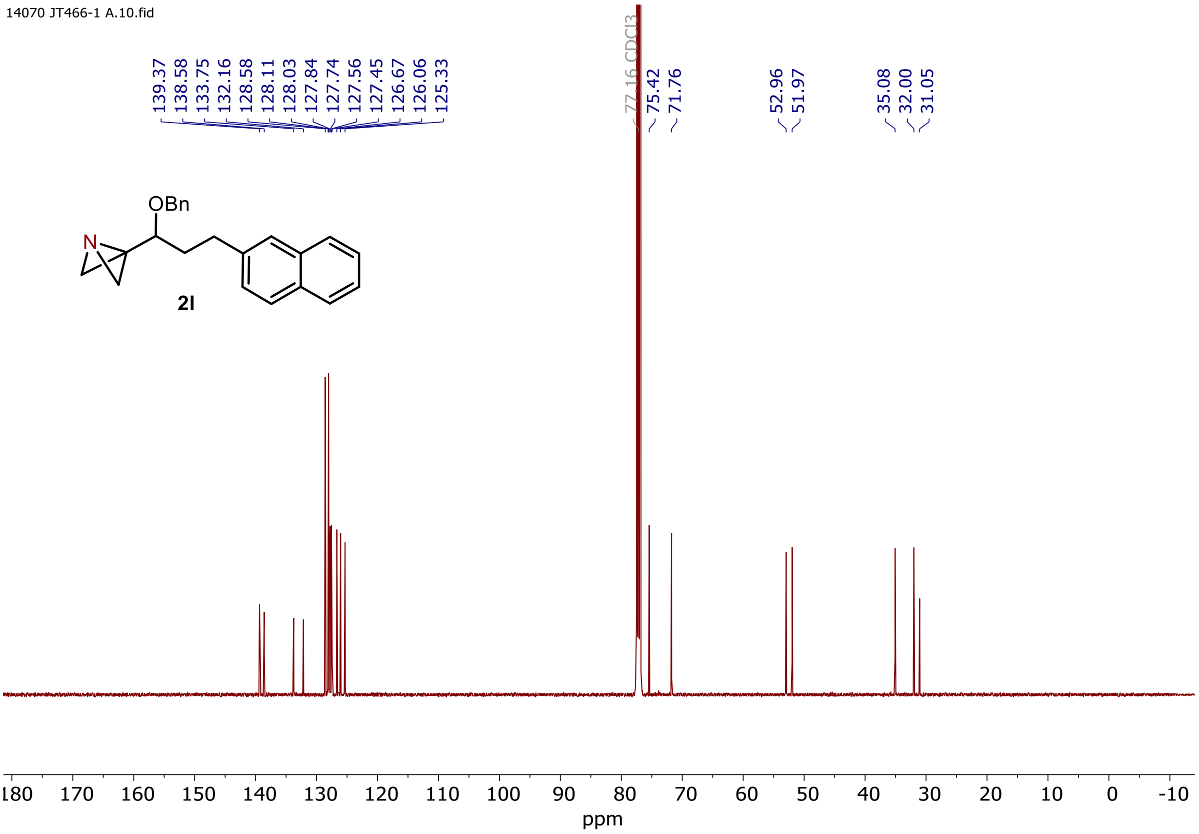

^{1}H NMR (500 MHz, $CDCl_3$) of **2m** (*see procedure*)

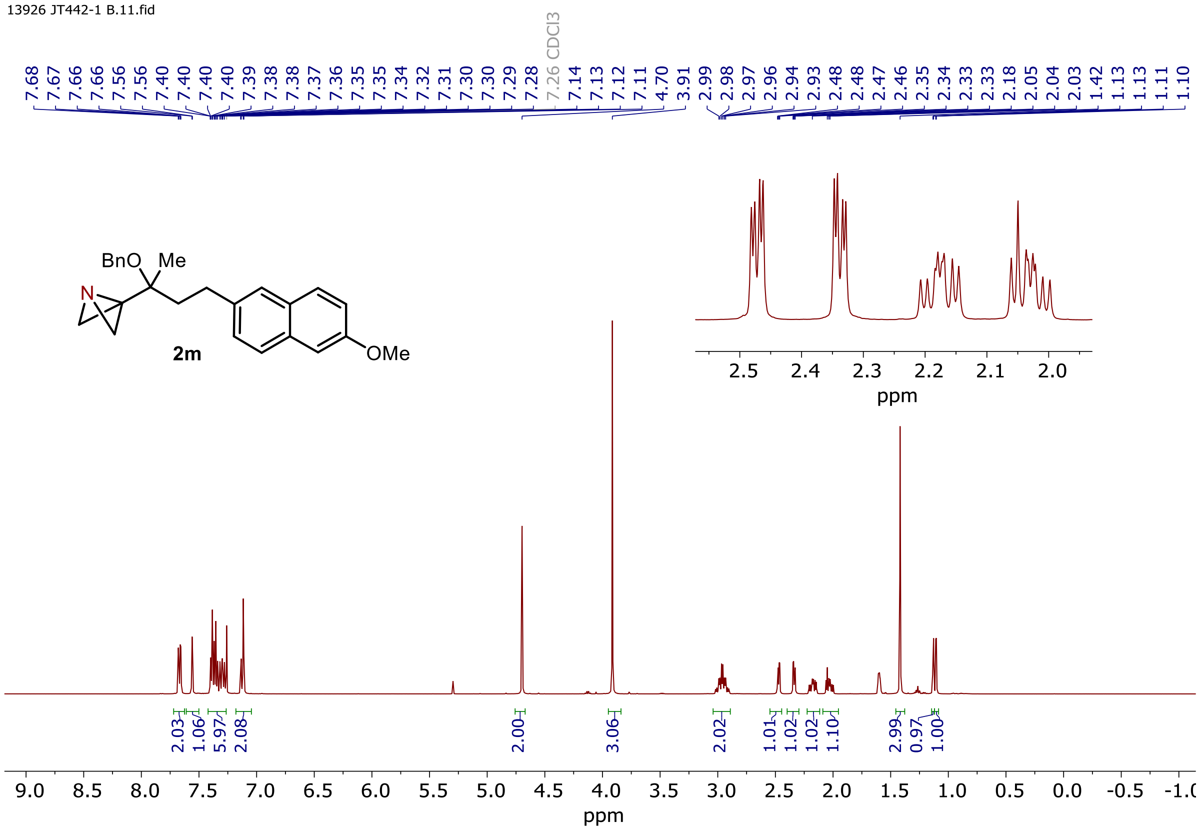

^{13}C NMR (126 MHz, $CDCl_3$) of **2m**

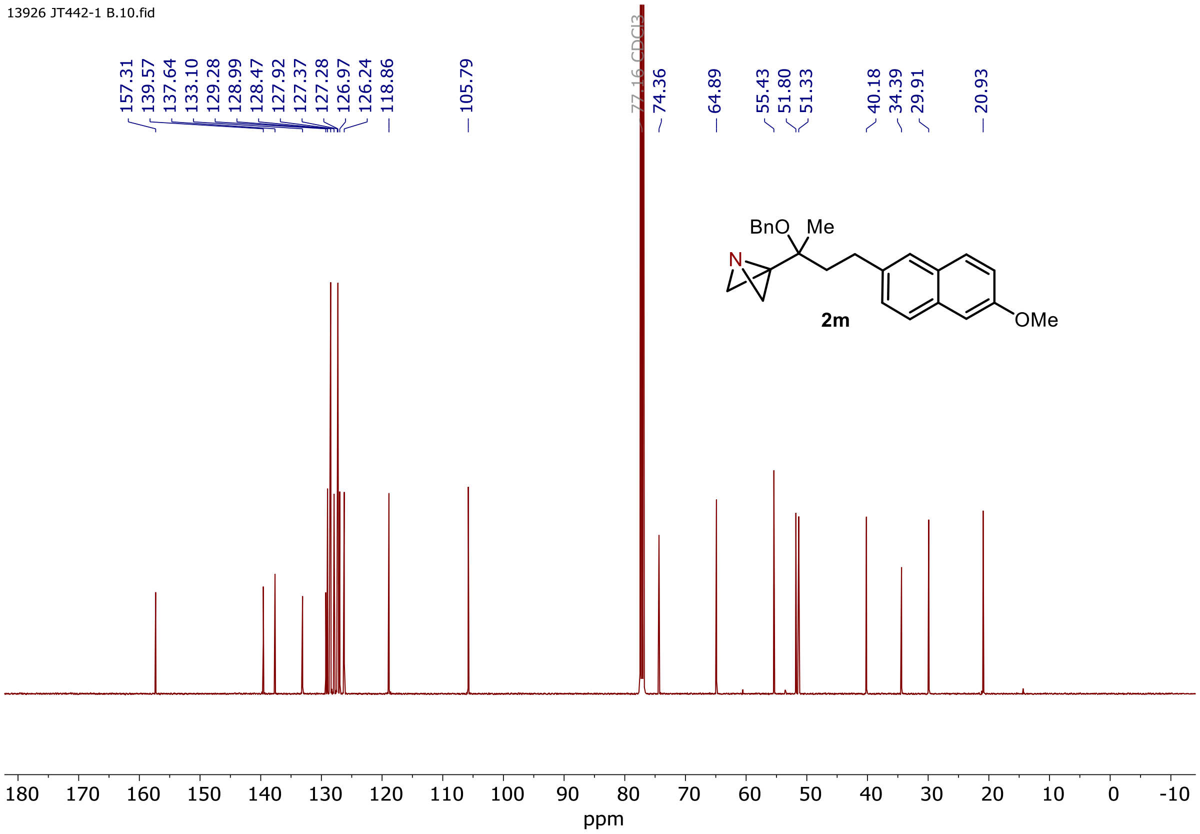

^{1}H NMR (400 MHz, $CDCl_3$) of **2n** (*see procedure*)

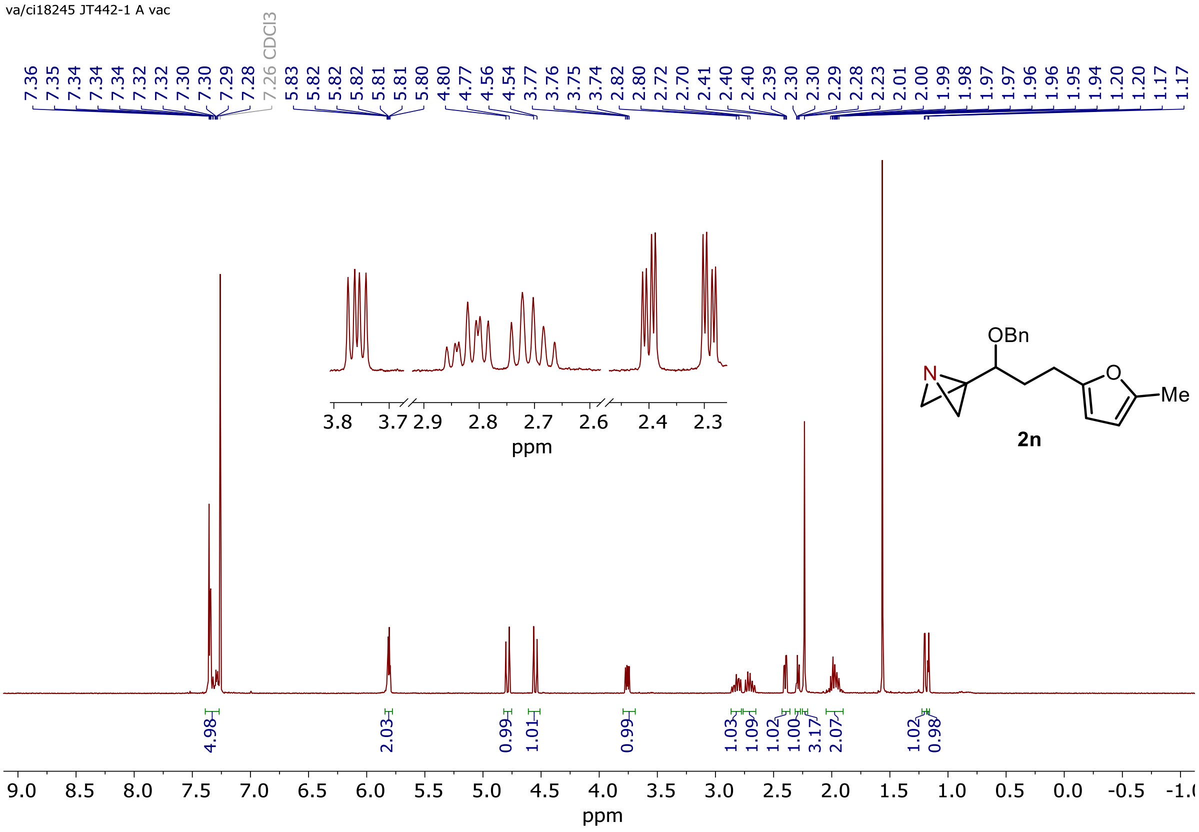

^{13}C NMR (126 MHz, $CDCl_3$) of **2n**

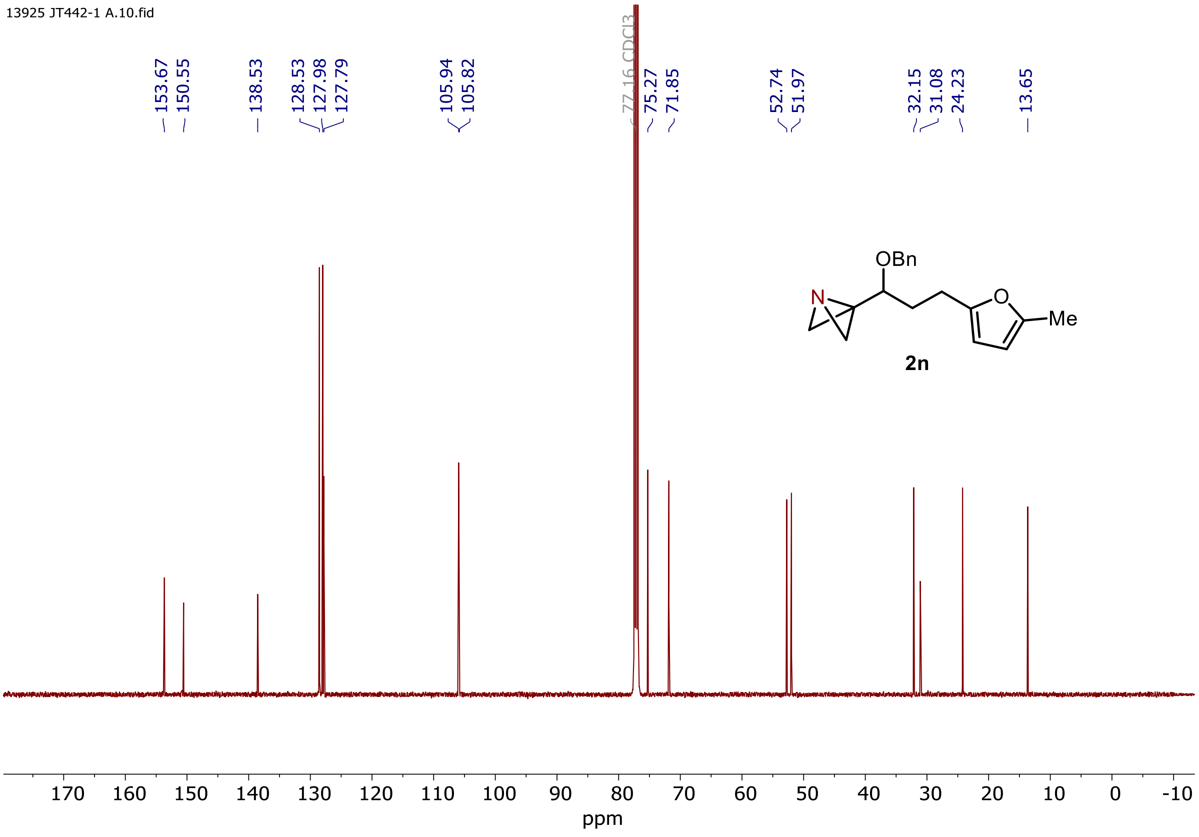

^{1}H NMR (500 MHz, $CDCl_3$) of **2o** (*see procedure*)

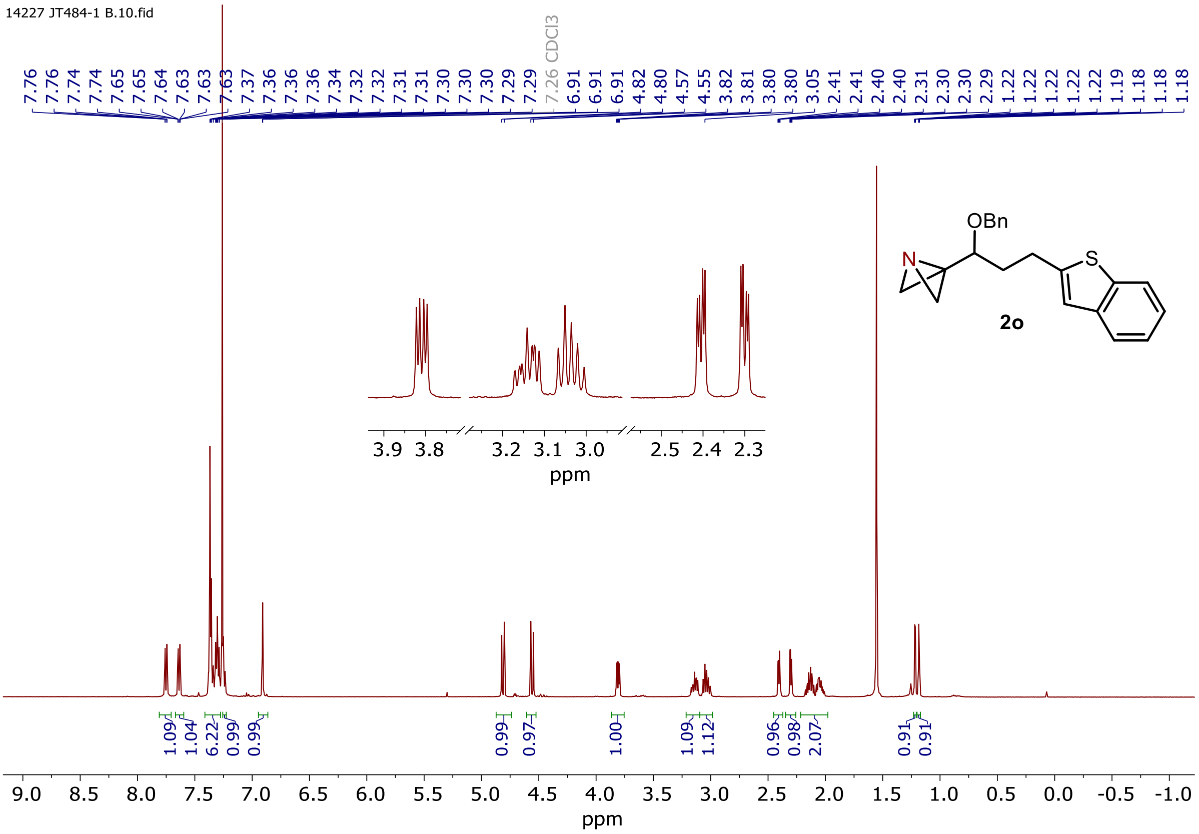

^{13}C NMR (126 MHz, $CDCl_3$) of **2o**

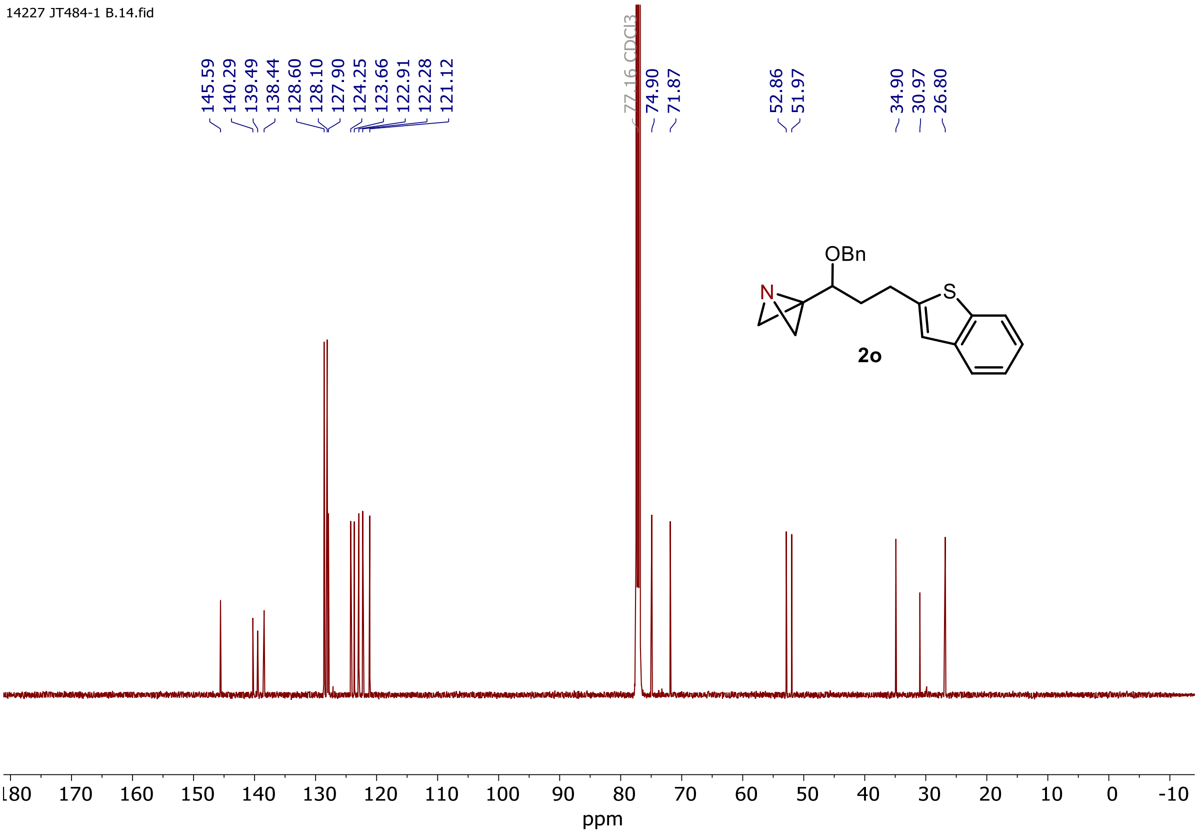

^{1}H NMR (500 MHz, $CDCl_3$) of **2p** (*see procedure*)

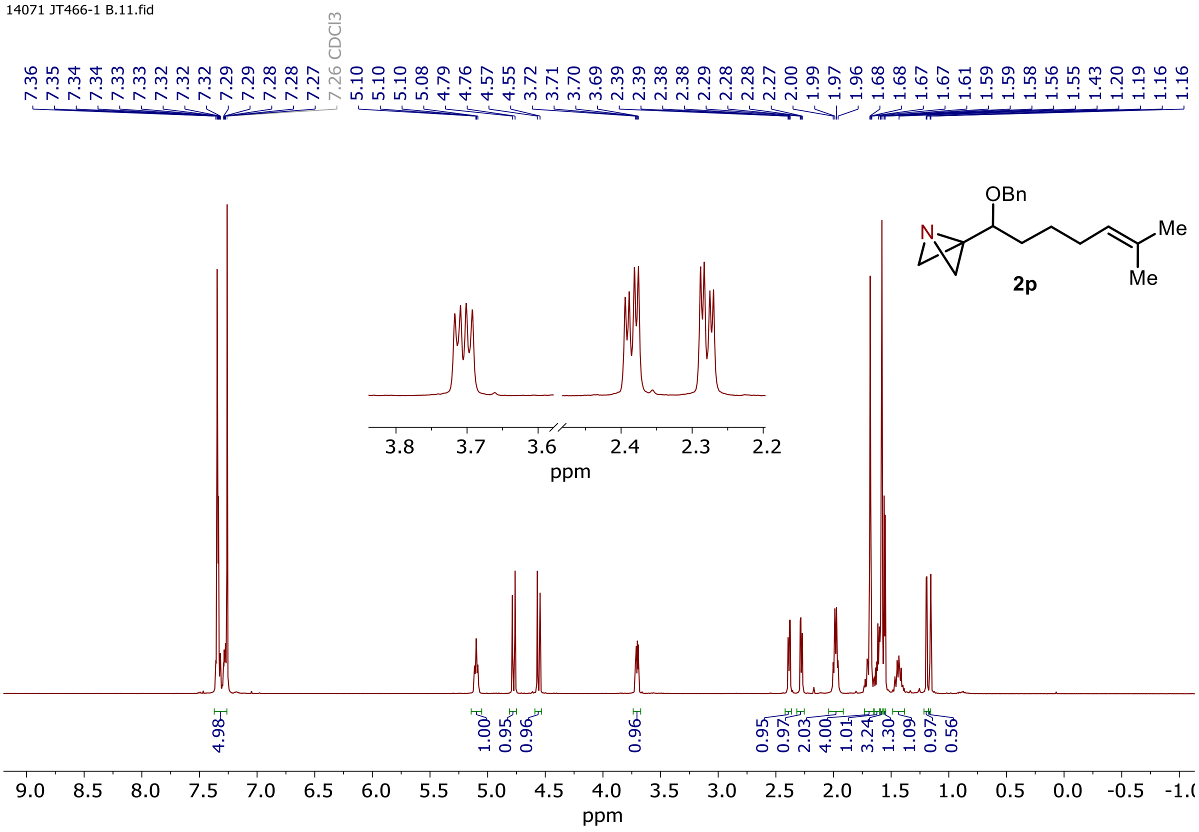

^{13}C NMR (126 MHz, $CDCl_3$) of **2p**

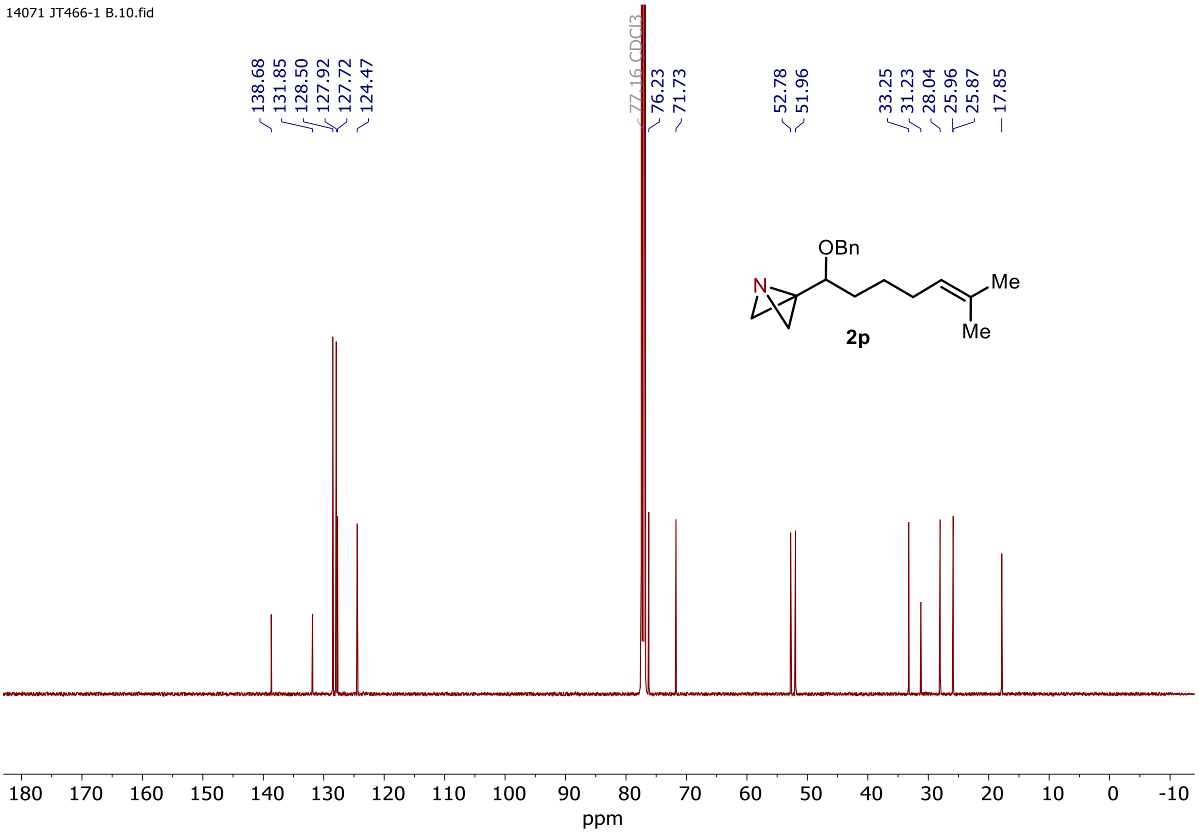

^{1}H NMR (500 MHz, $CDCl_3$) of **2q** (*see procedure*)

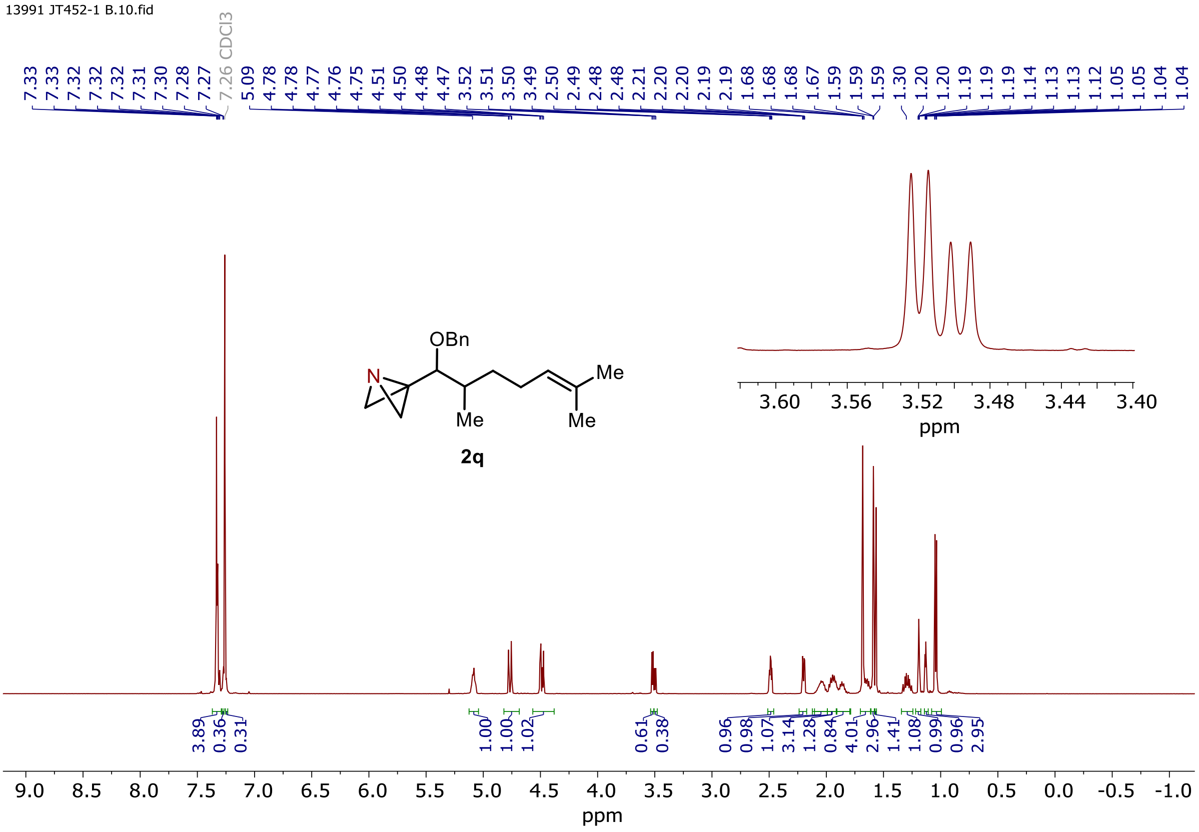

^{13}C NMR (126 MHz, $CDCl_3$) of **2q**

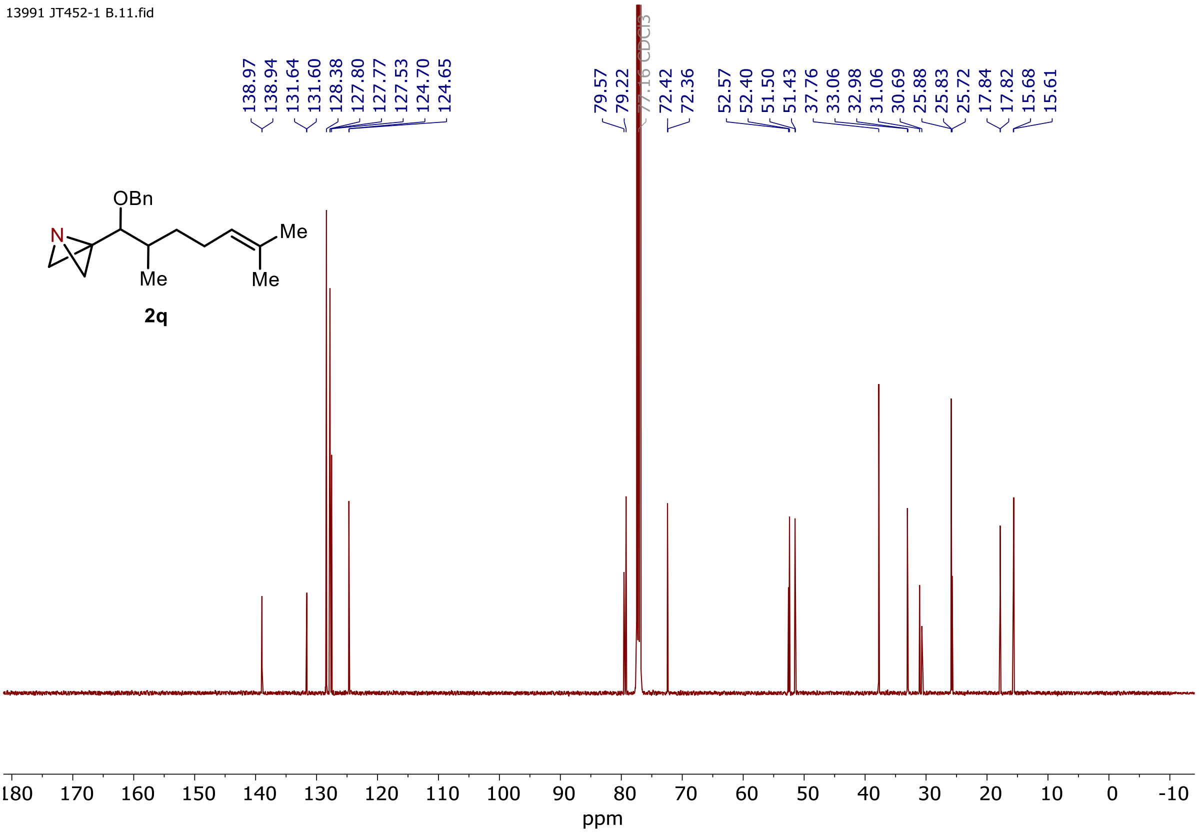

^{1}H NMR (400 MHz, $CDCl_3$) of **3-(1-(Methoxymethoxy)-3-phenylpropyl)-1-azabicyclo[1.1.0]butane** (see procedure)

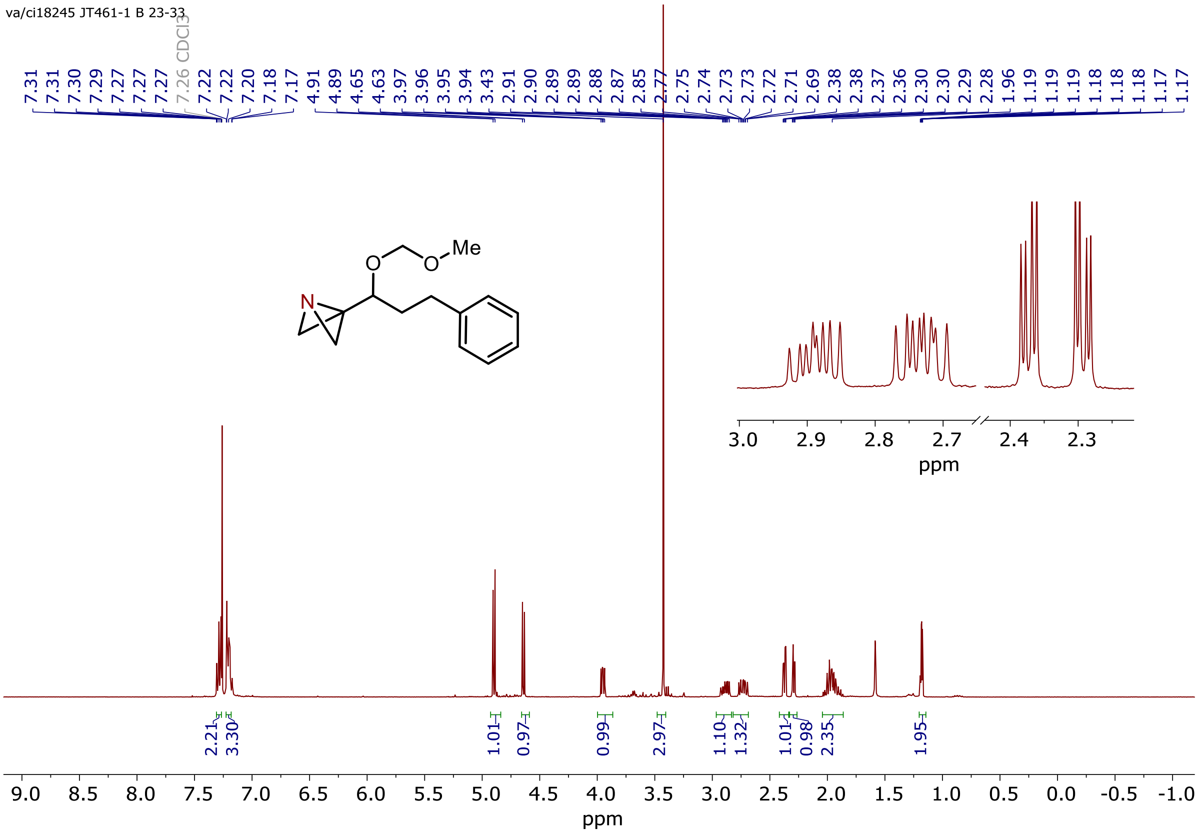

^{13}C NMR (126 MHz, $CDCl_3$) of **3-(1-(Methoxymethoxy)-3-phenylpropyl)-1-azabicyclo[1.1.0]butane**

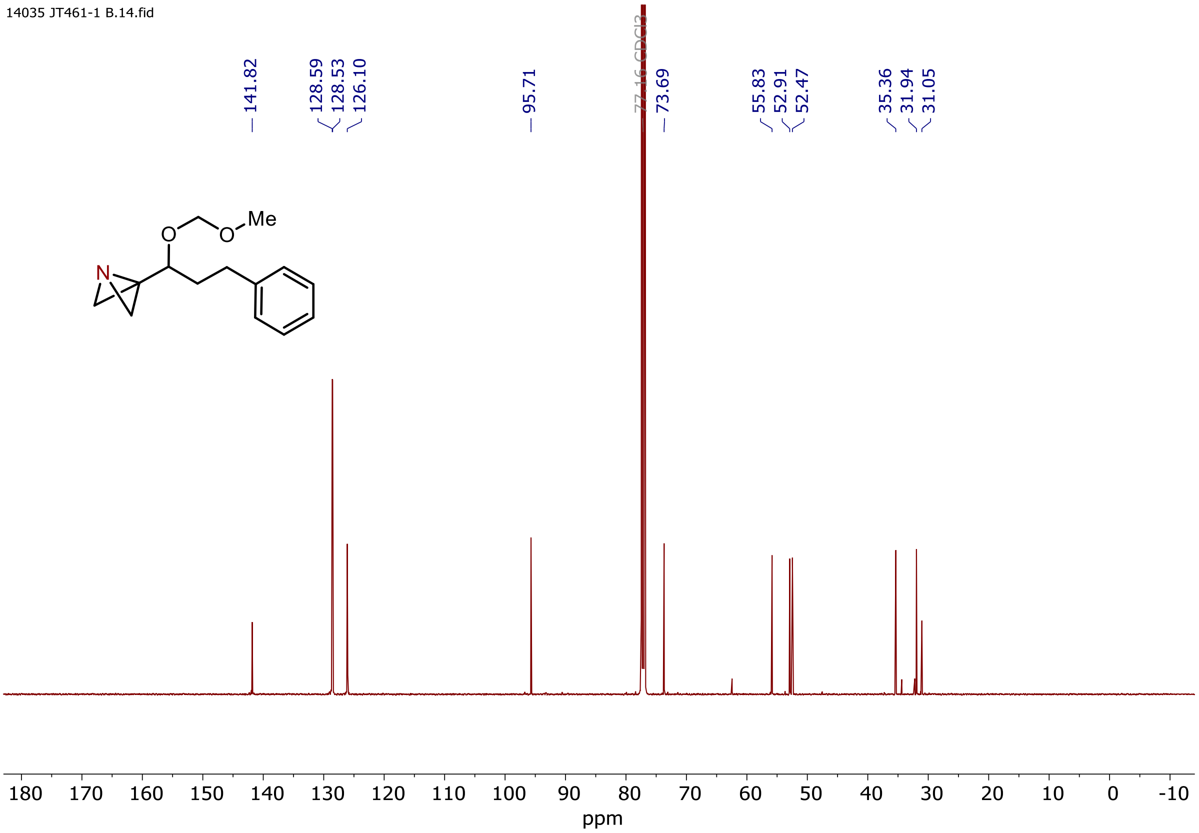

^{1}H NMR (400 MHz, $CDCl_3$) of **1-(1-Azabicyclo[1.1.0]butan-3-yl)-3-phenylpropyl acetate** (*see procedure*)

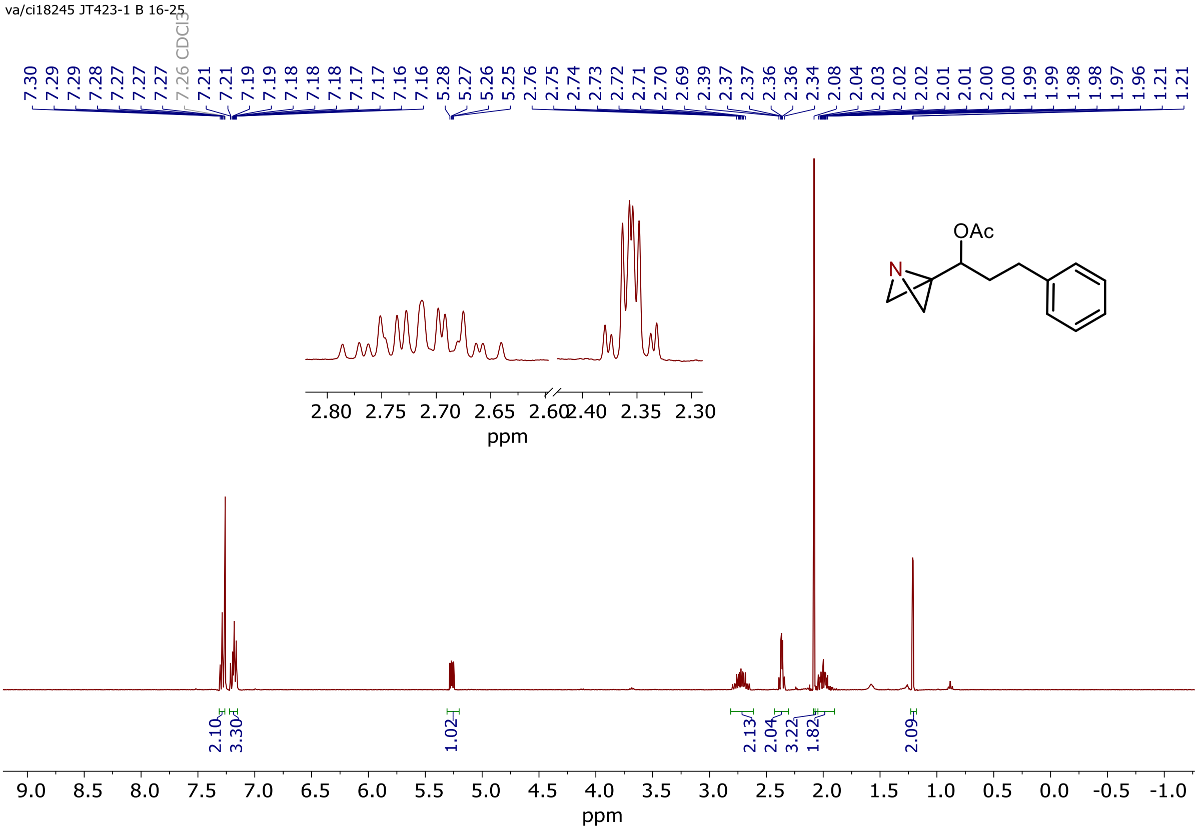

^{13}C NMR (126 MHz, $CDCl_3$) of **1-(1-Azabicyclo[1.1.0]butan-3-yl)-3-phenylpropyl acetate**

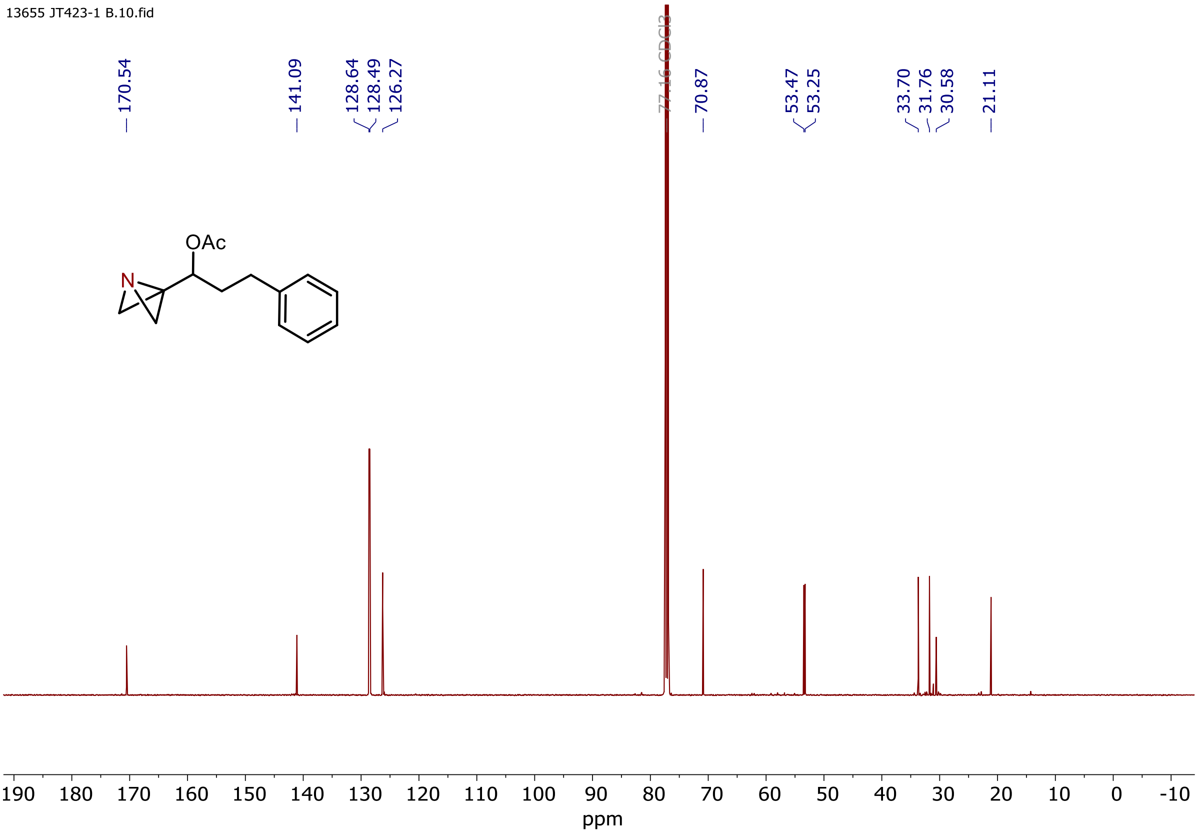

^{1}H NMR (400 MHz, $CDCl_3$) of **3-(1-(Benzyloxy)-2-phenylethyl)-1-azabicyclo[1.1.0]butane** (*see procedure*)

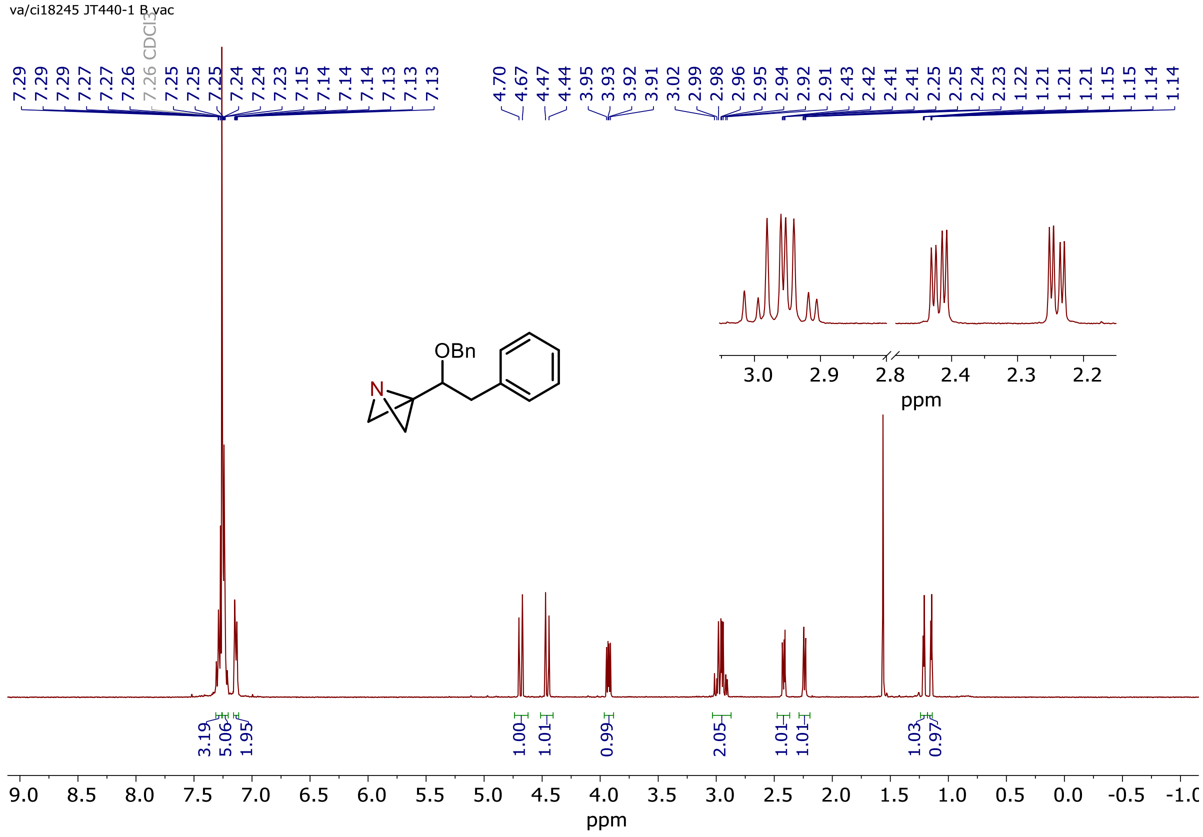

^{13}C NMR (126 MHz, $CDCl_3$) of **3-(1-(Benzyloxy)-2-phenylethyl)-1-azabicyclo[1.1.0]butane**

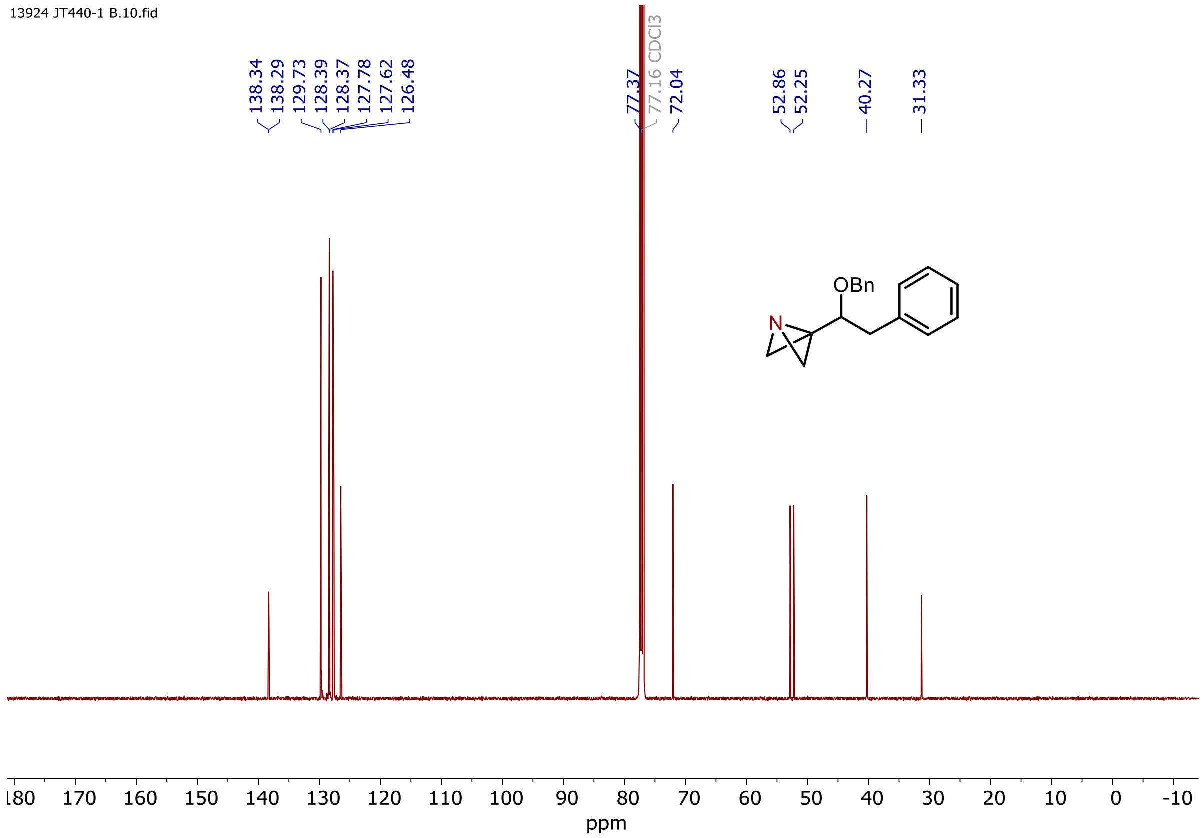

^{1}H NMR (500 MHz, $CDCl_3$) of **3-(1-(Benzyloxy)-4-phenylbutyl)-1-azabicyclo[1.1.0]butane** (*see procedure*)

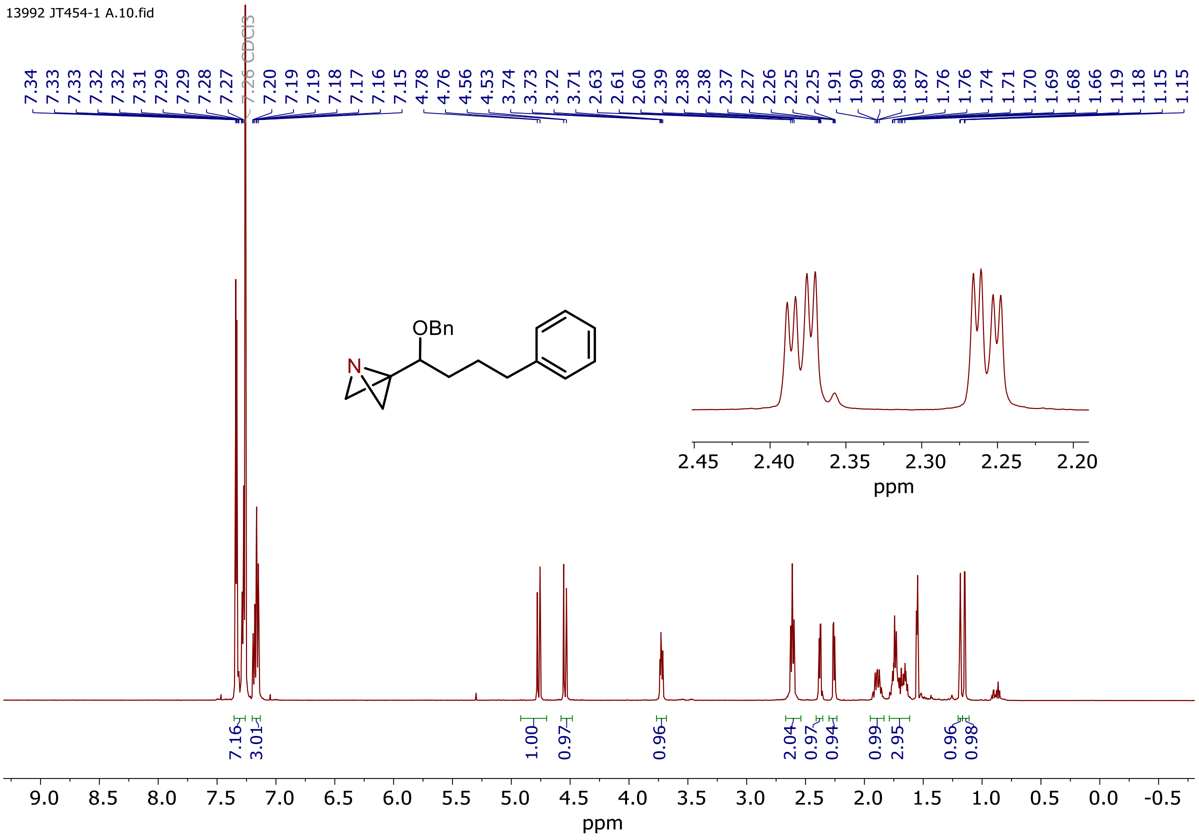

^{13}C NMR (126 MHz, $CDCl_3$) of **3-(1-(Benzyloxy)-4-phenylbutyl)-1-azabicyclo[1.1.0]butane**

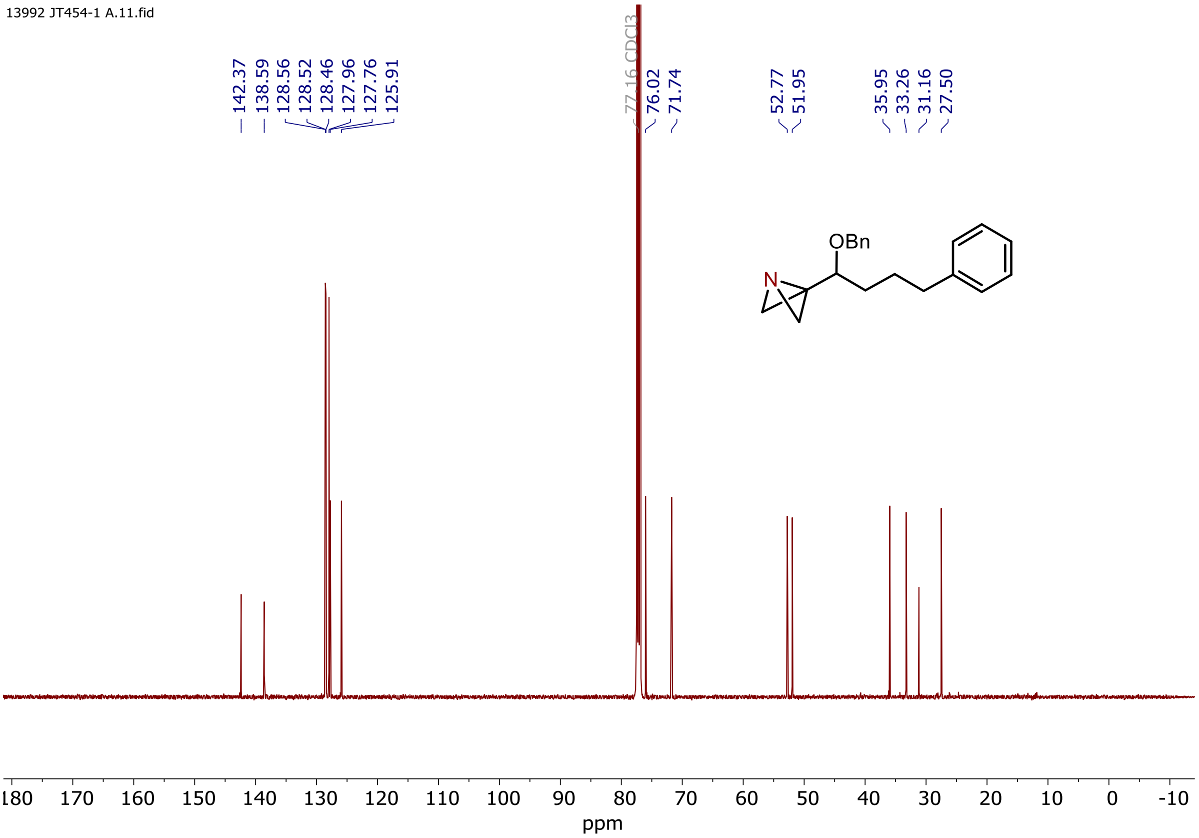

^{1}H NMR (500 MHz, $CDCl_3$) of **4a** (*see procedure*)

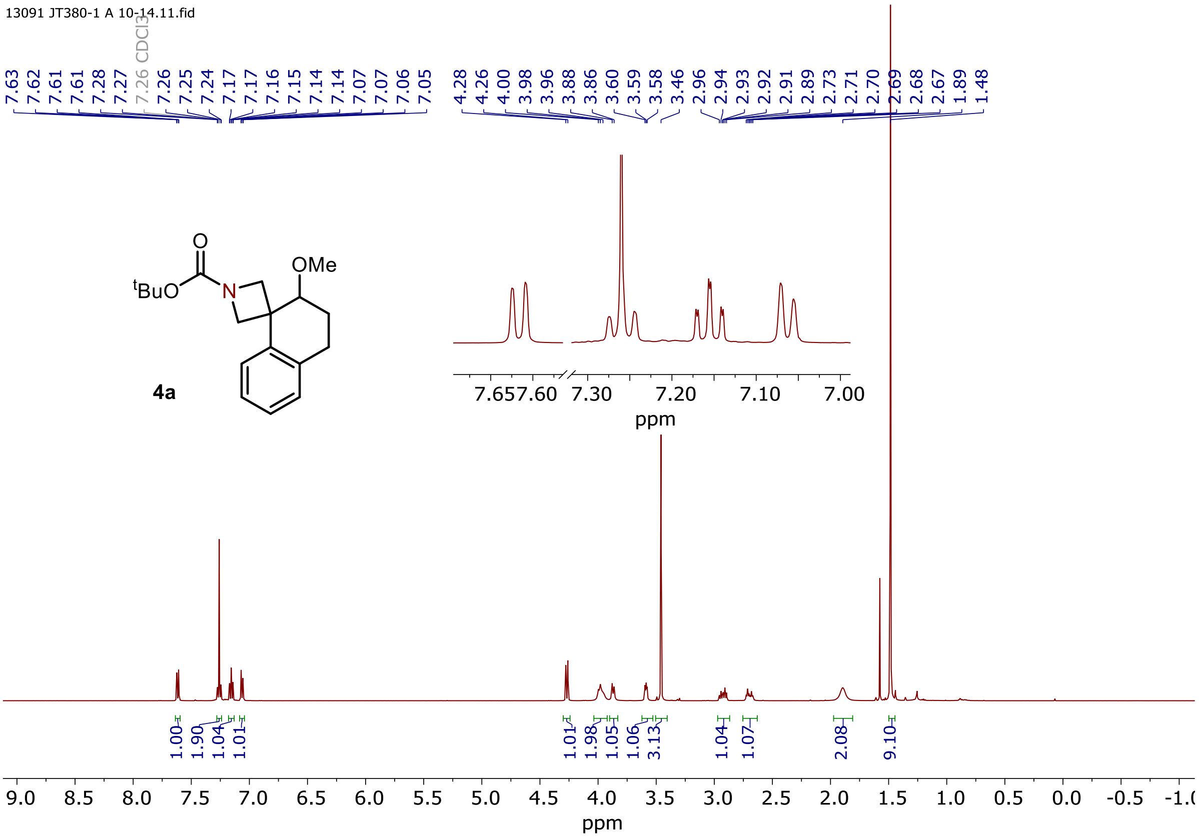

^{13}C NMR (126 MHz, $CDCl_3$) of **4a**

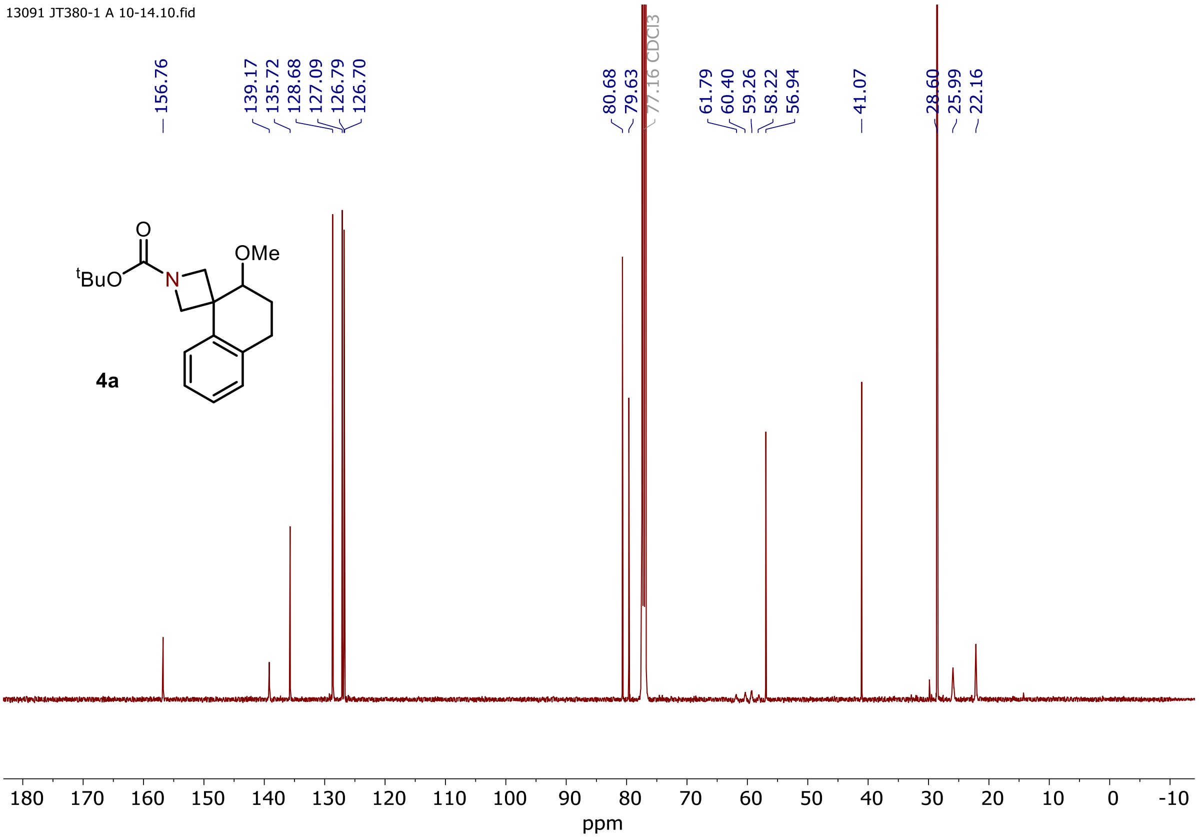

^{1}H NMR (400 MHz, $CDCl_3$) of **4b** (*see procedure*)

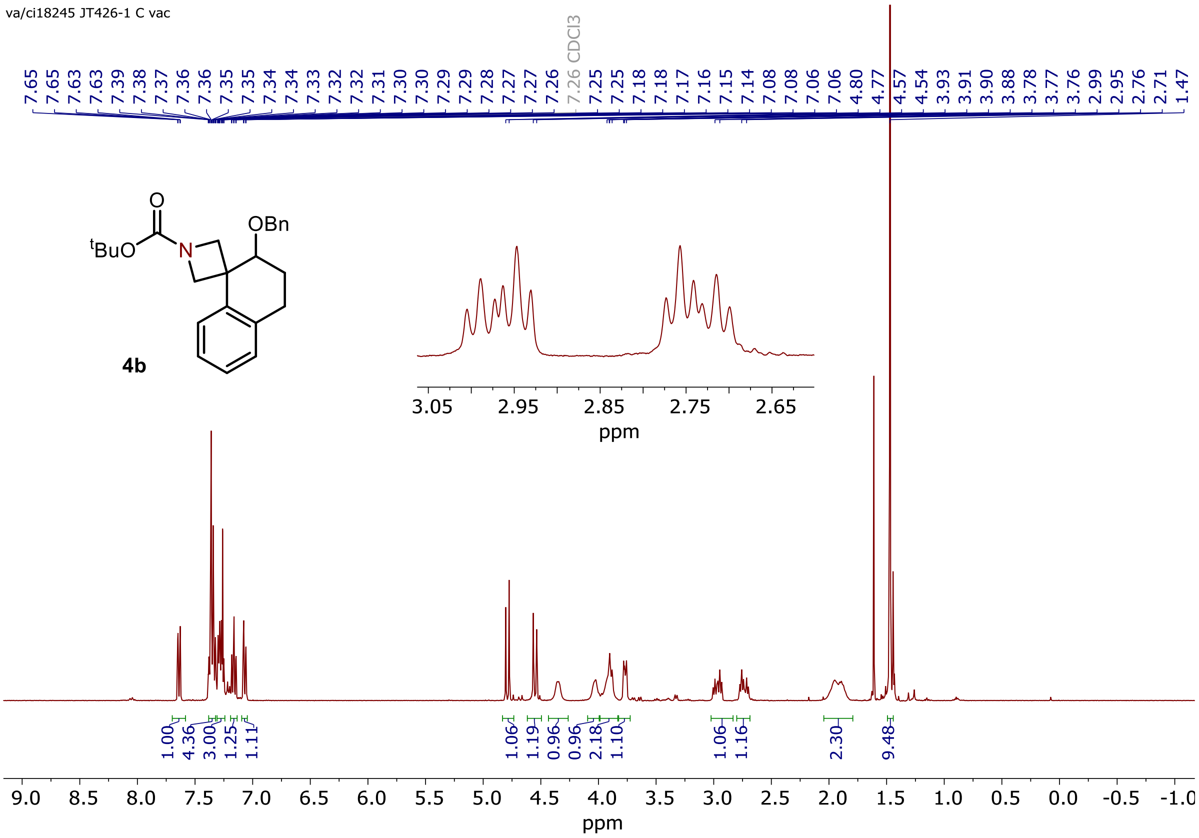

^{13}C NMR (126 MHz, $CDCl_3$) of **4b**

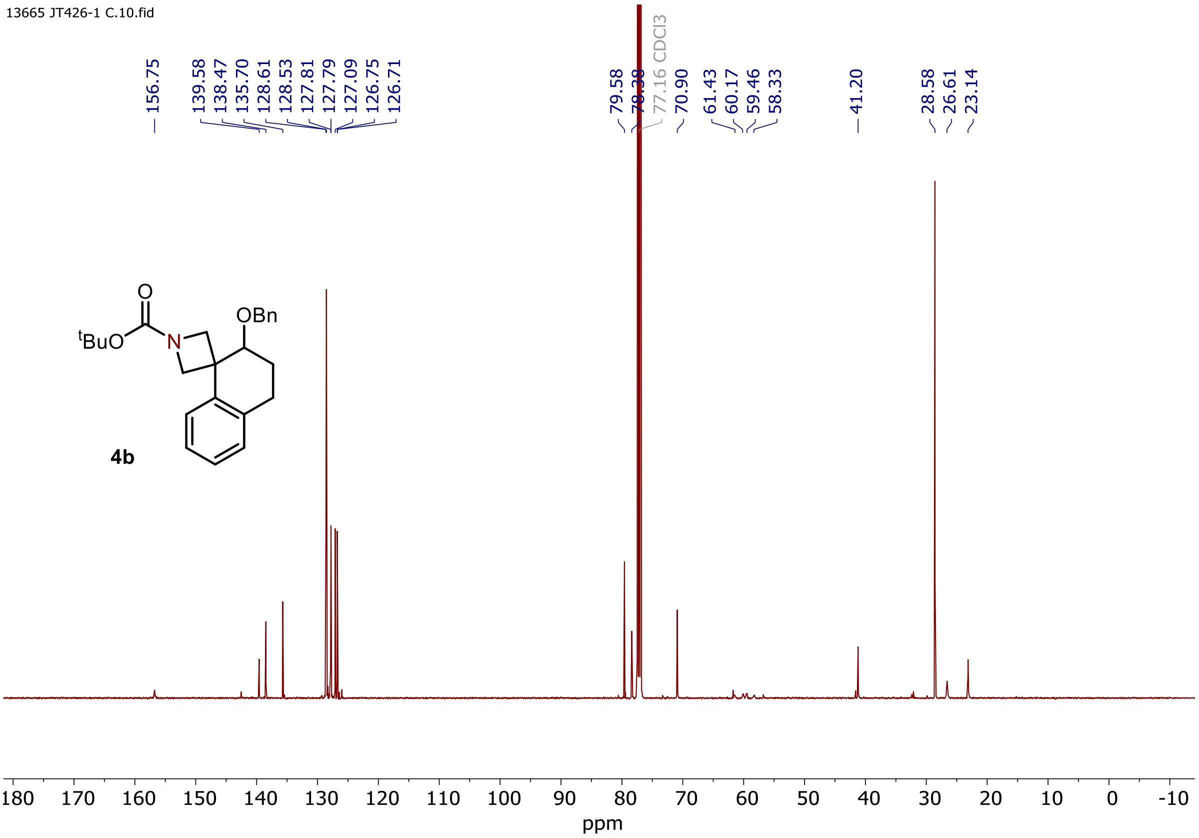

^{1}H NMR (400 MHz, $CDCl_3$) of **4c** (see procedure)

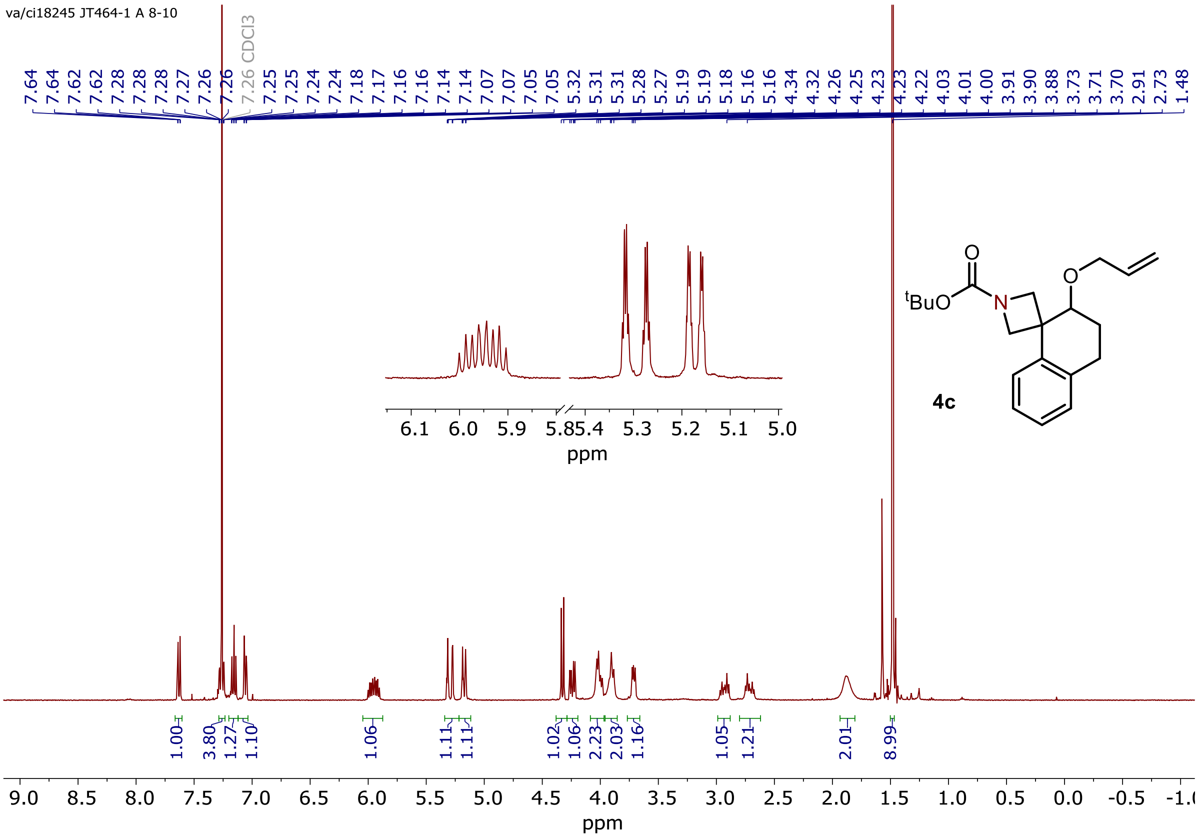

^{13}C NMR (126 MHz, $CDCl_3$) of **4c**

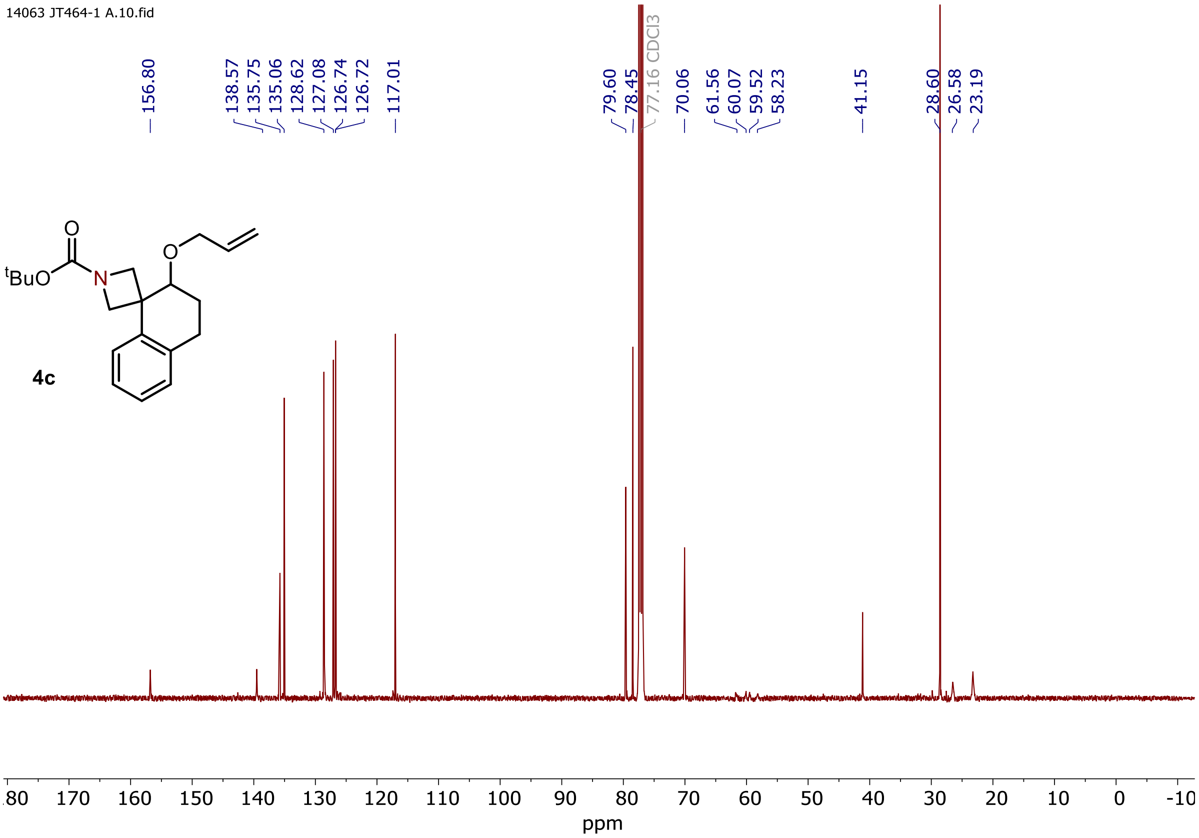

^{1}H NMR (400 MHz, $CDCl_3$) of **4d** (*see procedure*)

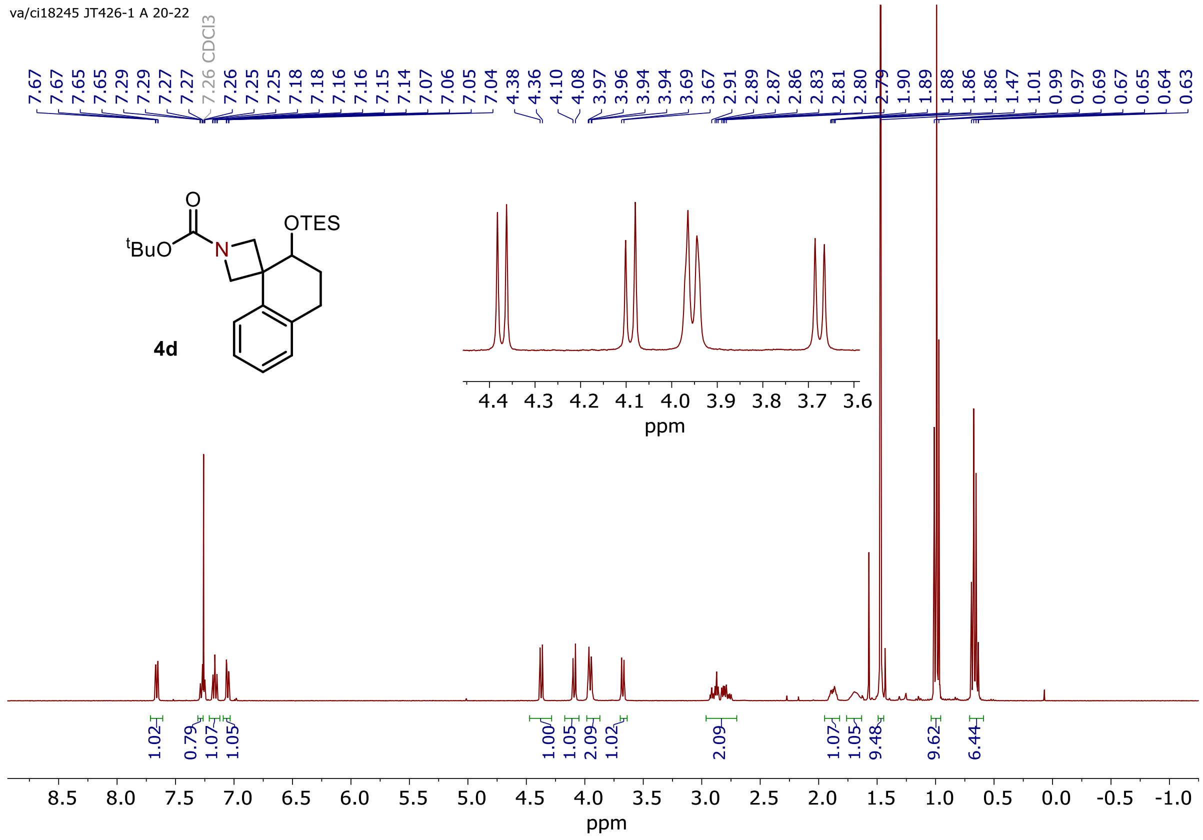

^{13}C NMR (126 MHz, $CDCl_3$) of **4d**

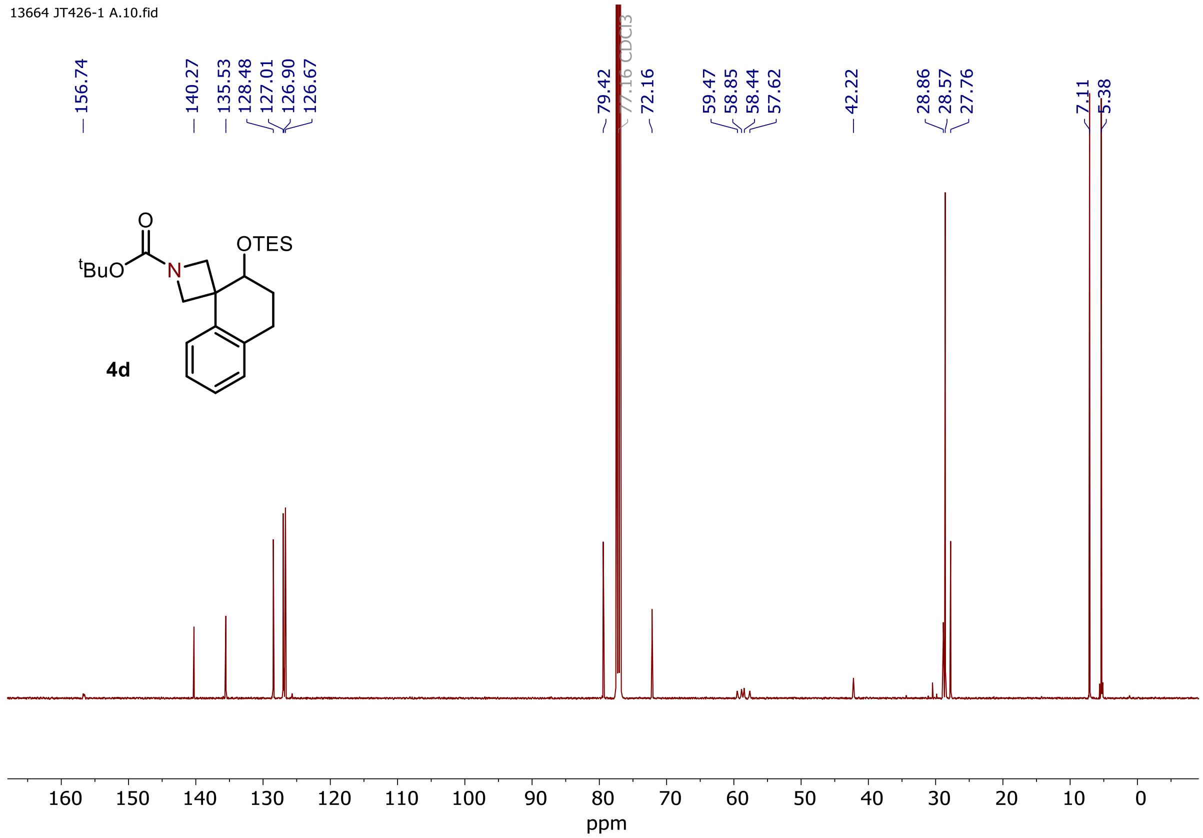

^{1}H NMR (400 MHz, $CDCl_3$) of **4e** (*see procedure*)

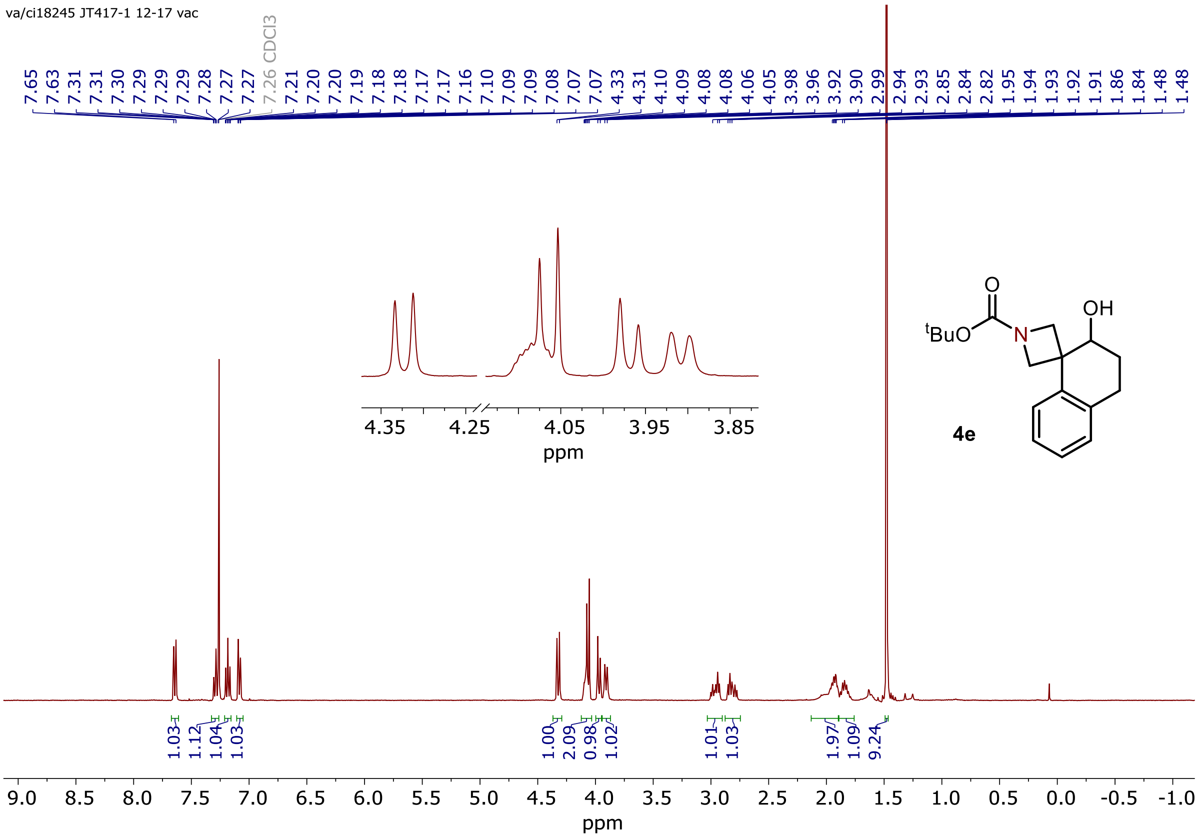

^{13}C NMR (126 MHz, $CDCl_3$) of **4e**

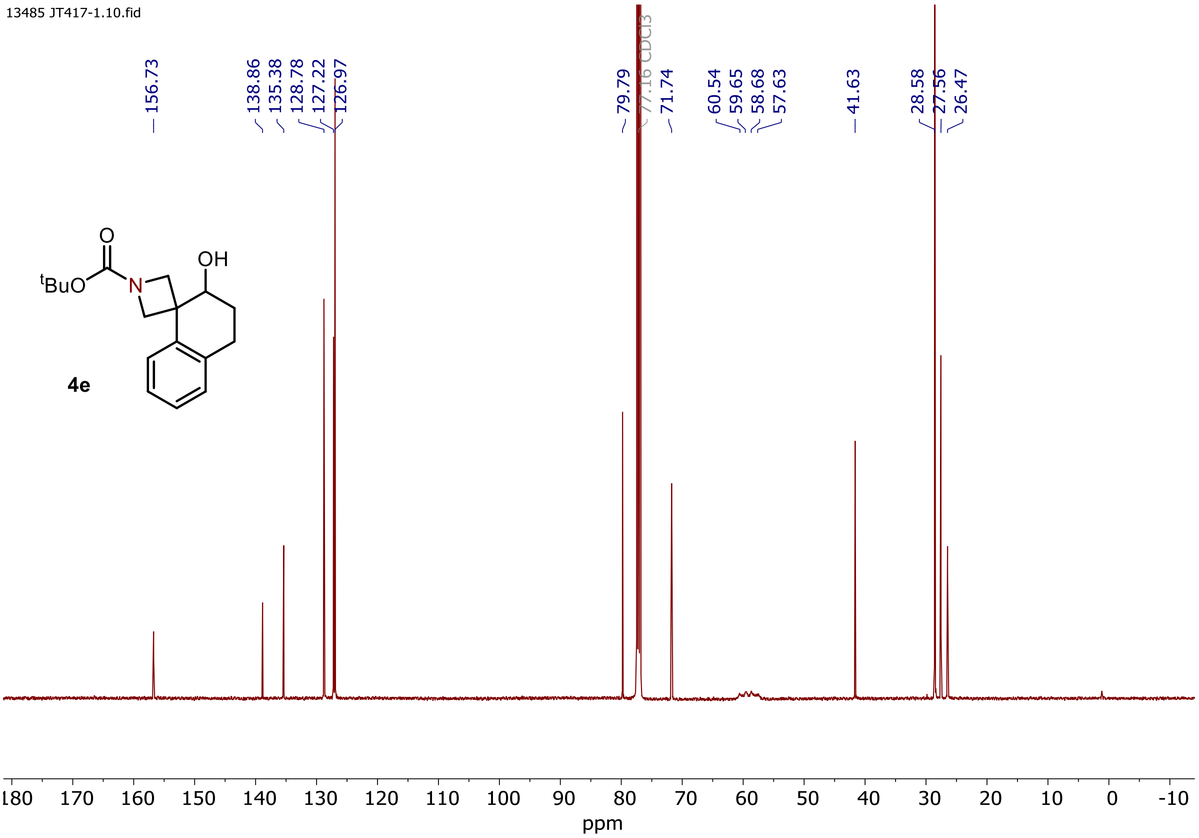

^{1}H NMR (400 MHz, $CDCl_3$) of **4f** (*see procedure*)

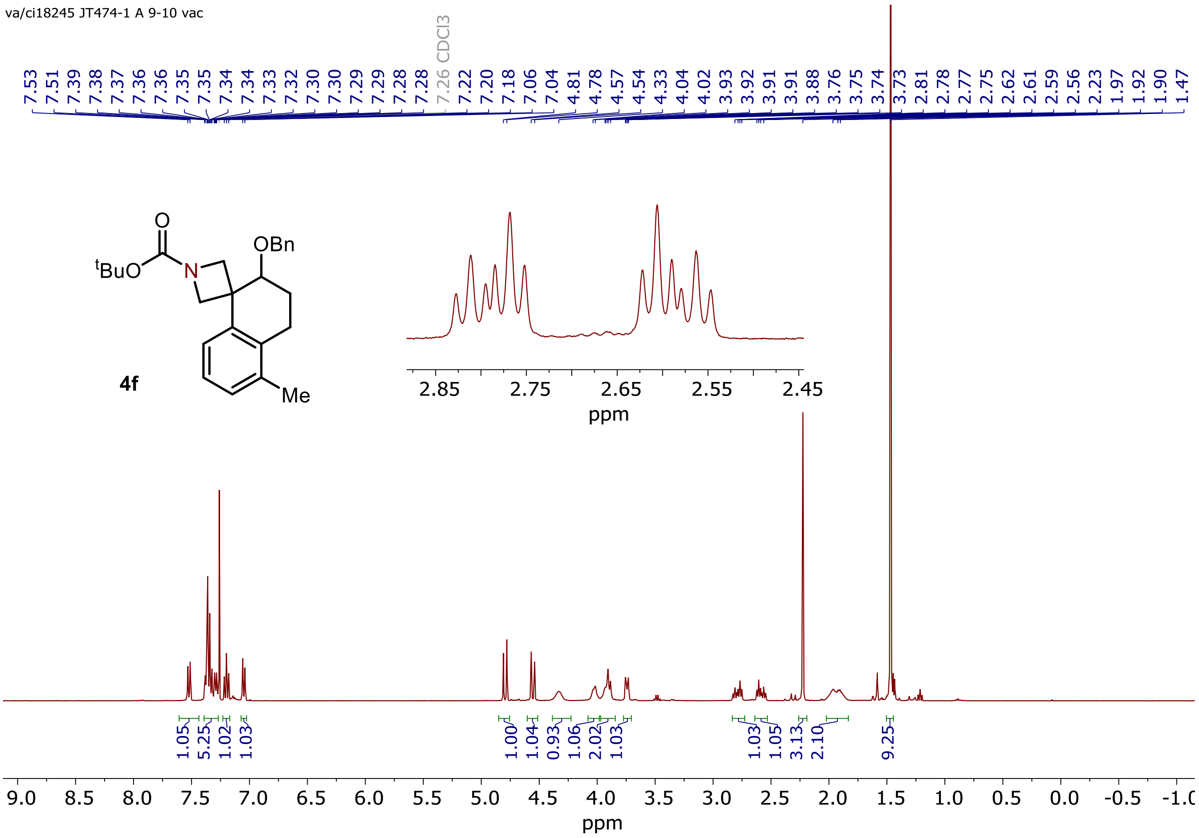

^{13}C NMR (126 MHz, $CDCl_3$) of **4f**

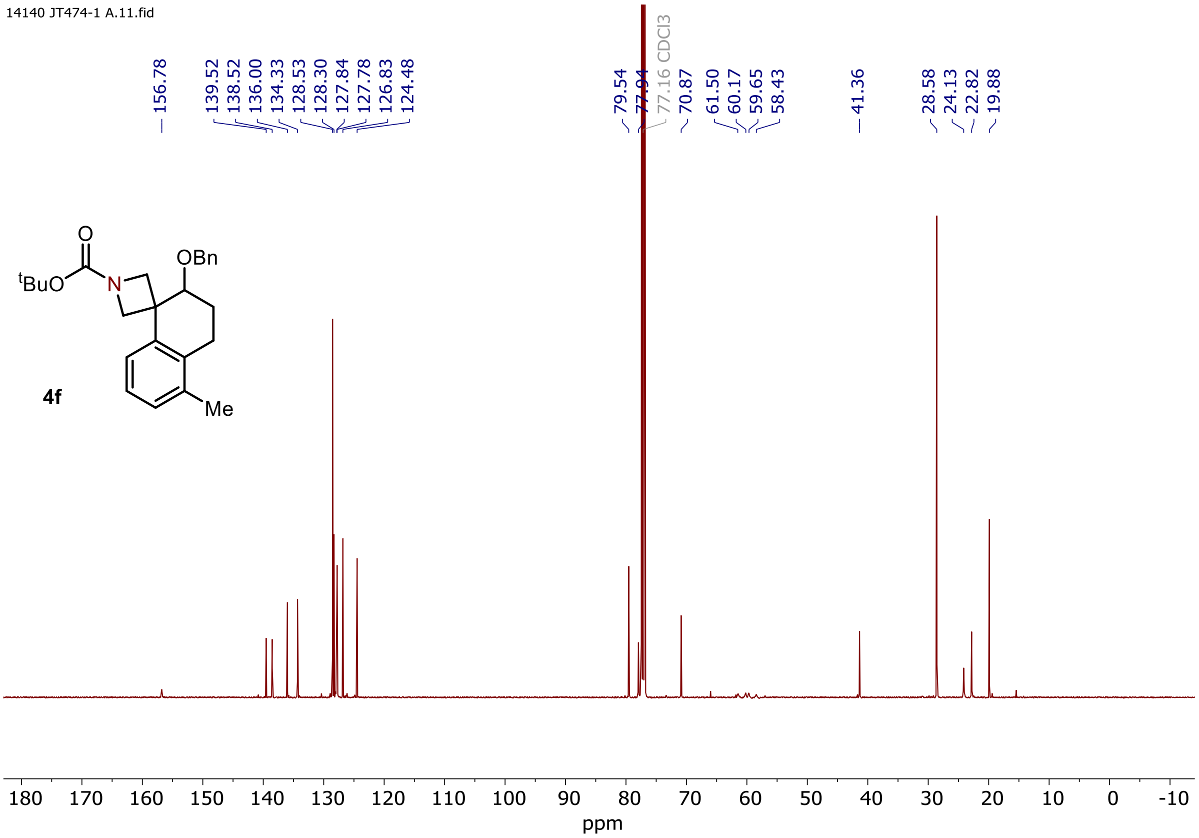

^{1}H NMR (500 MHz, $CDCl_3$) of **4g** (*see procedure*)

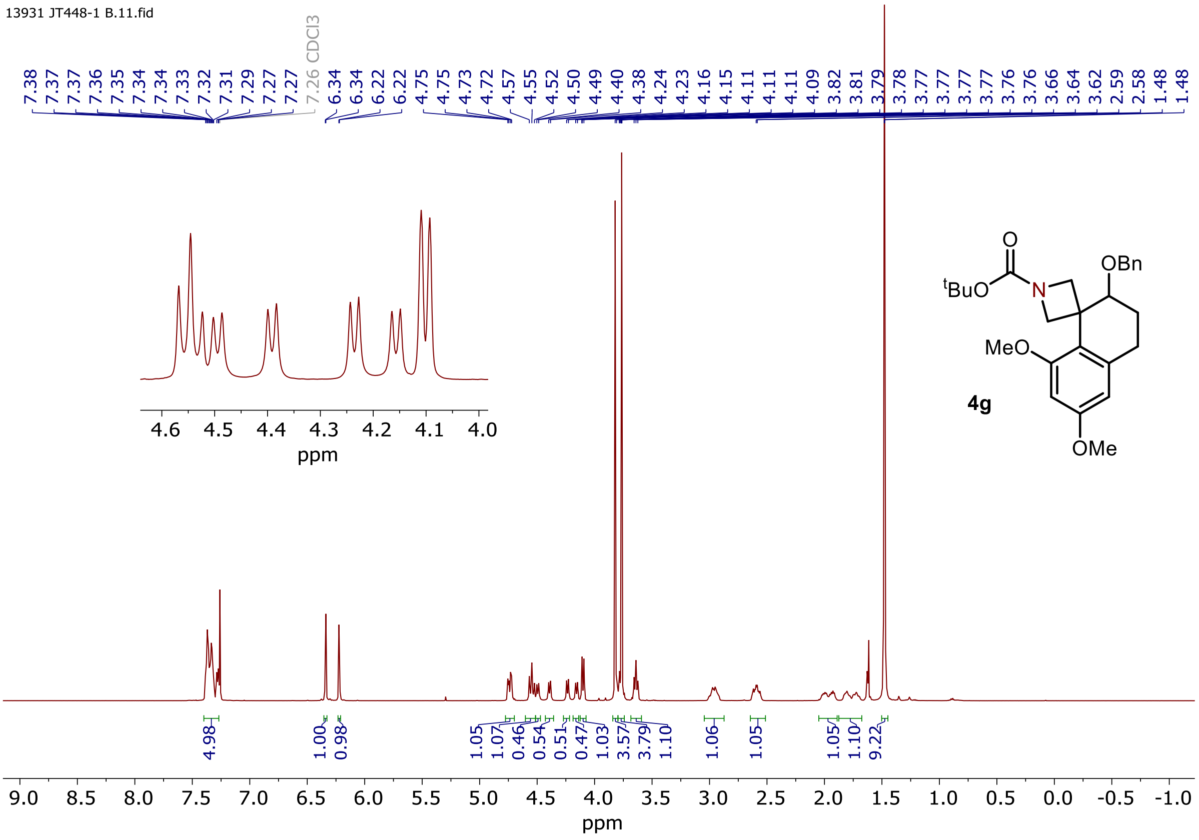

^{13}C NMR (126 MHz, $CDCl_3$) of **4g**

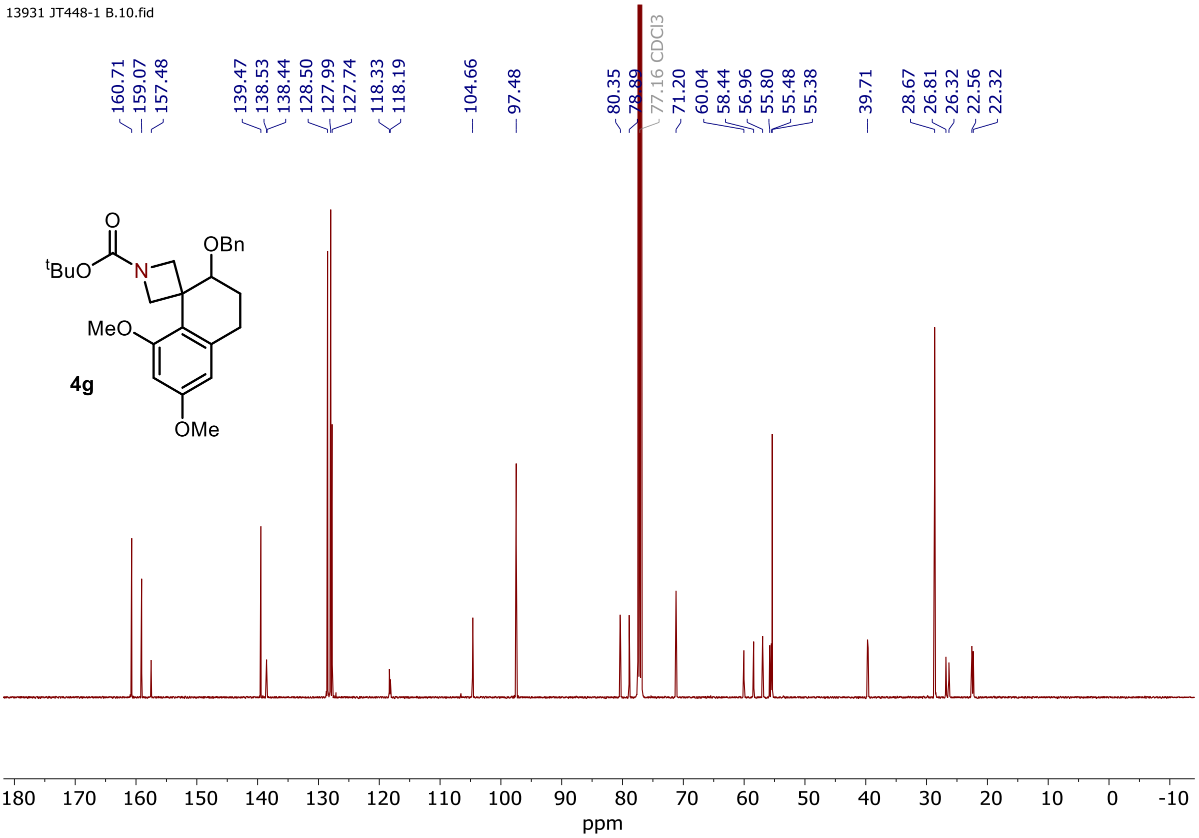

^{1}H NMR (500 MHz, $CDCl_3$) of **4h** (*see procedure*)

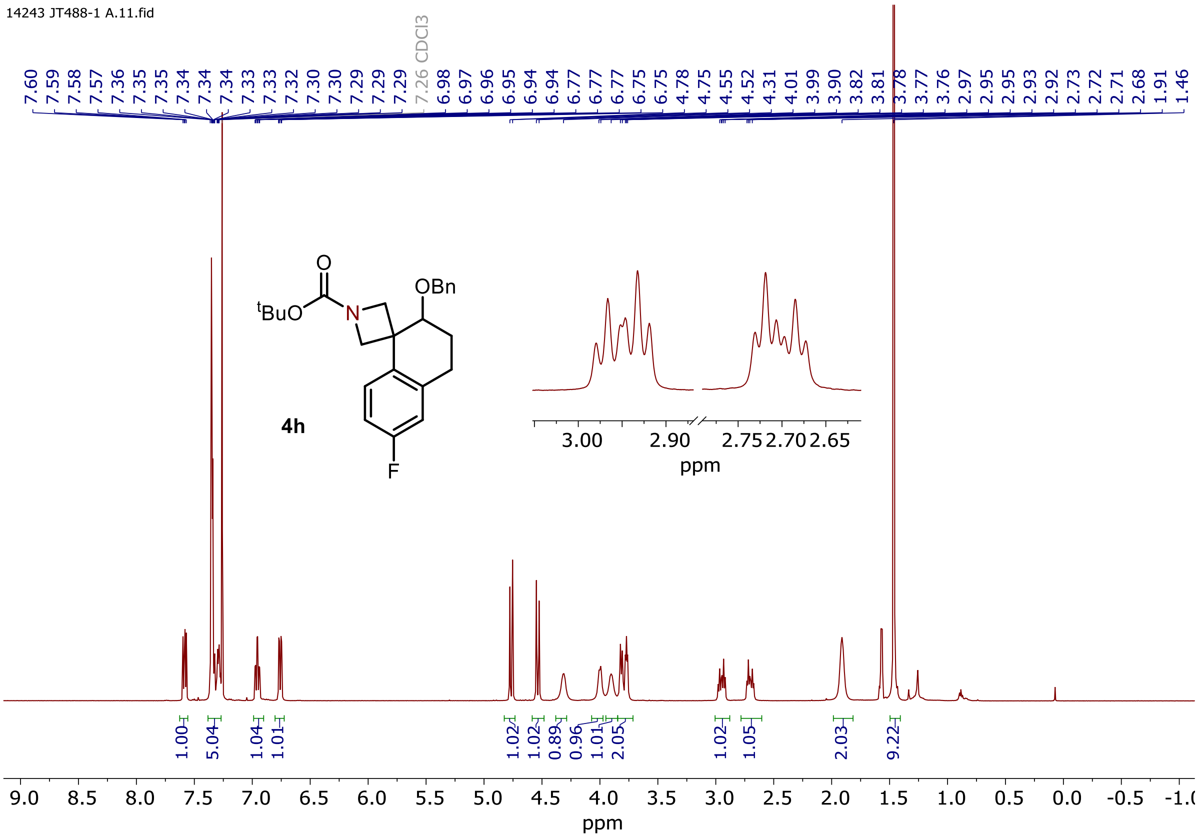

^{13}C NMR (126 MHz, $CDCl_3$) of **4h**

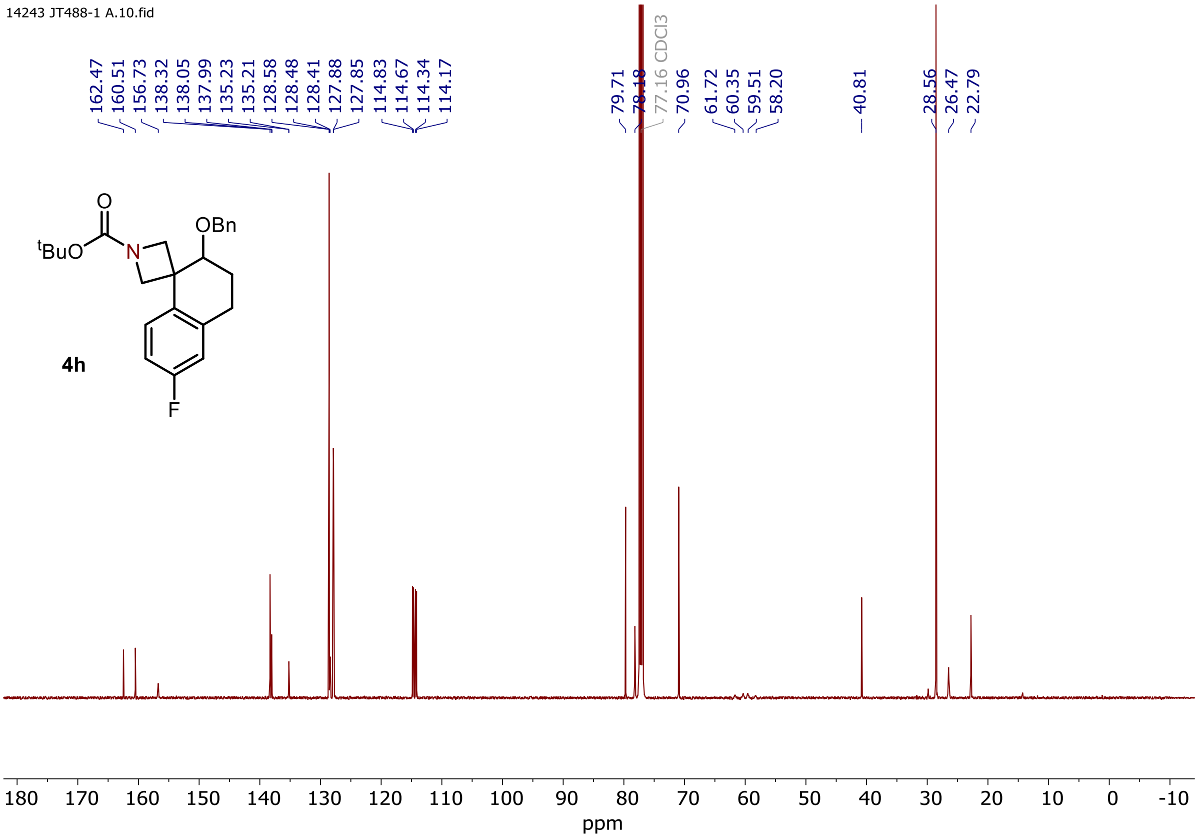

^{19}F NMR (376 MHz, $CDCl_3$) of **4h**

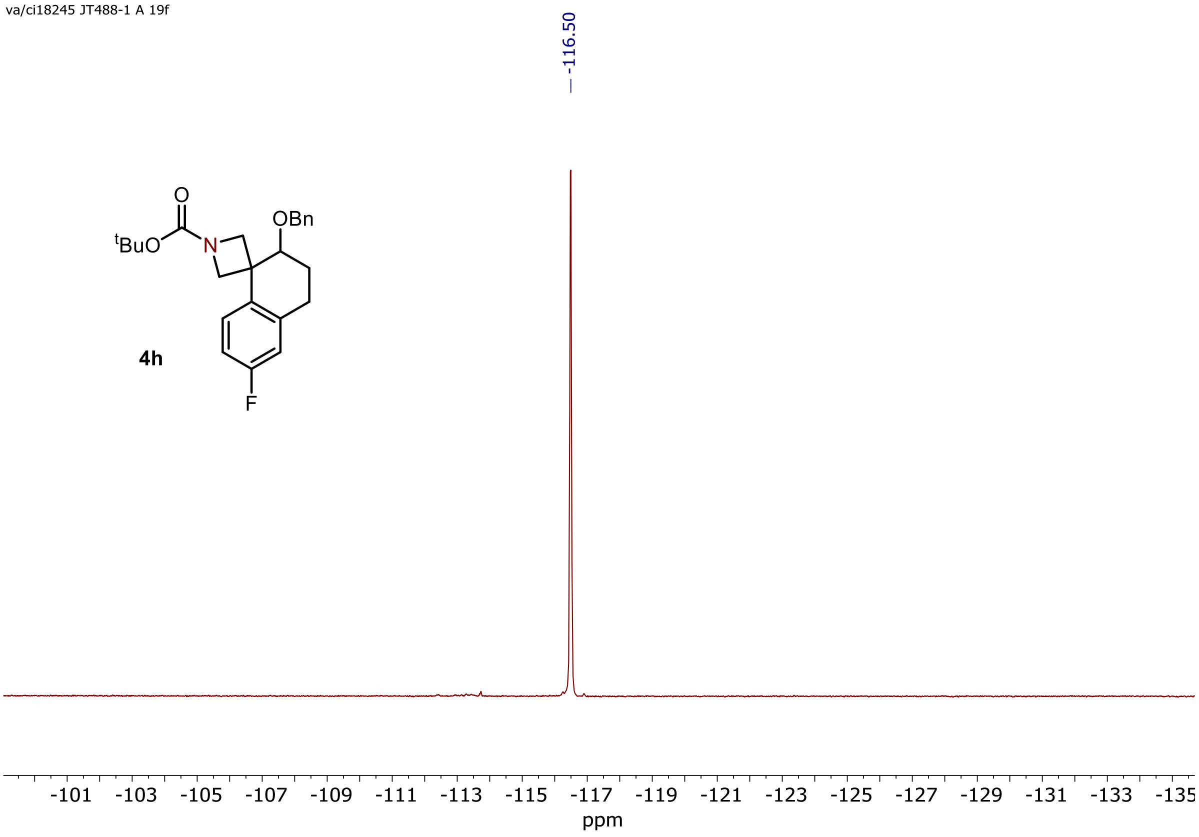

^{1}H NMR (400 MHz, $CDCl_3$) of **4i** (*see procedure*)

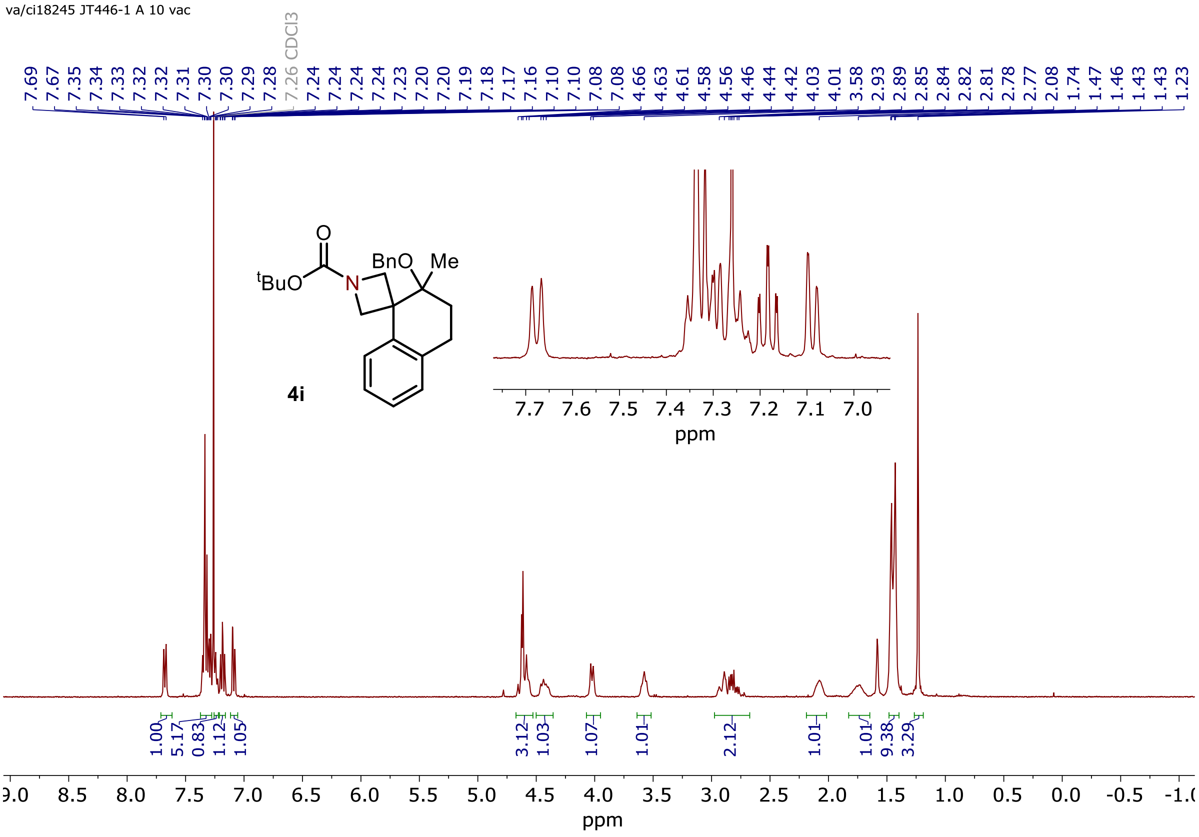

^{13}C NMR (126 MHz, $CDCl_3$) of **4i**

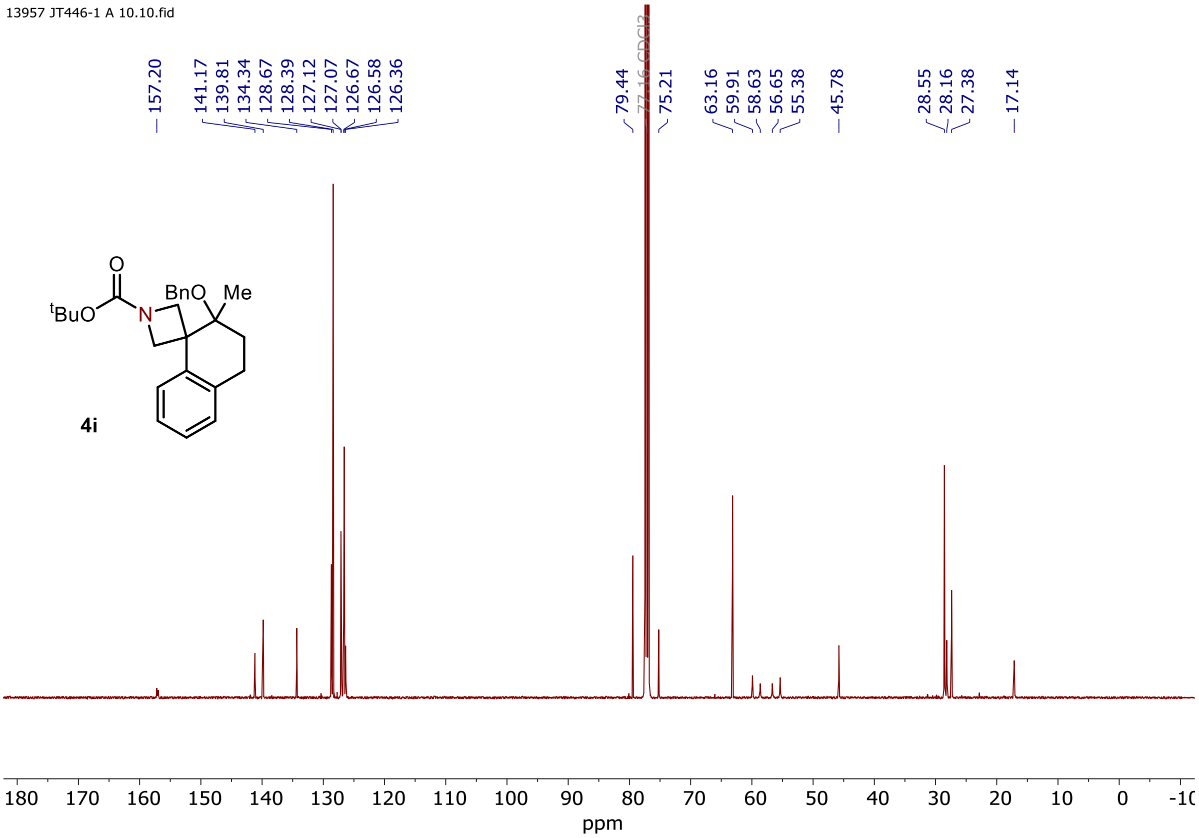

^{1}H NMR (400 MHz, $CDCl_3$) of **4j** (*see procedure*)

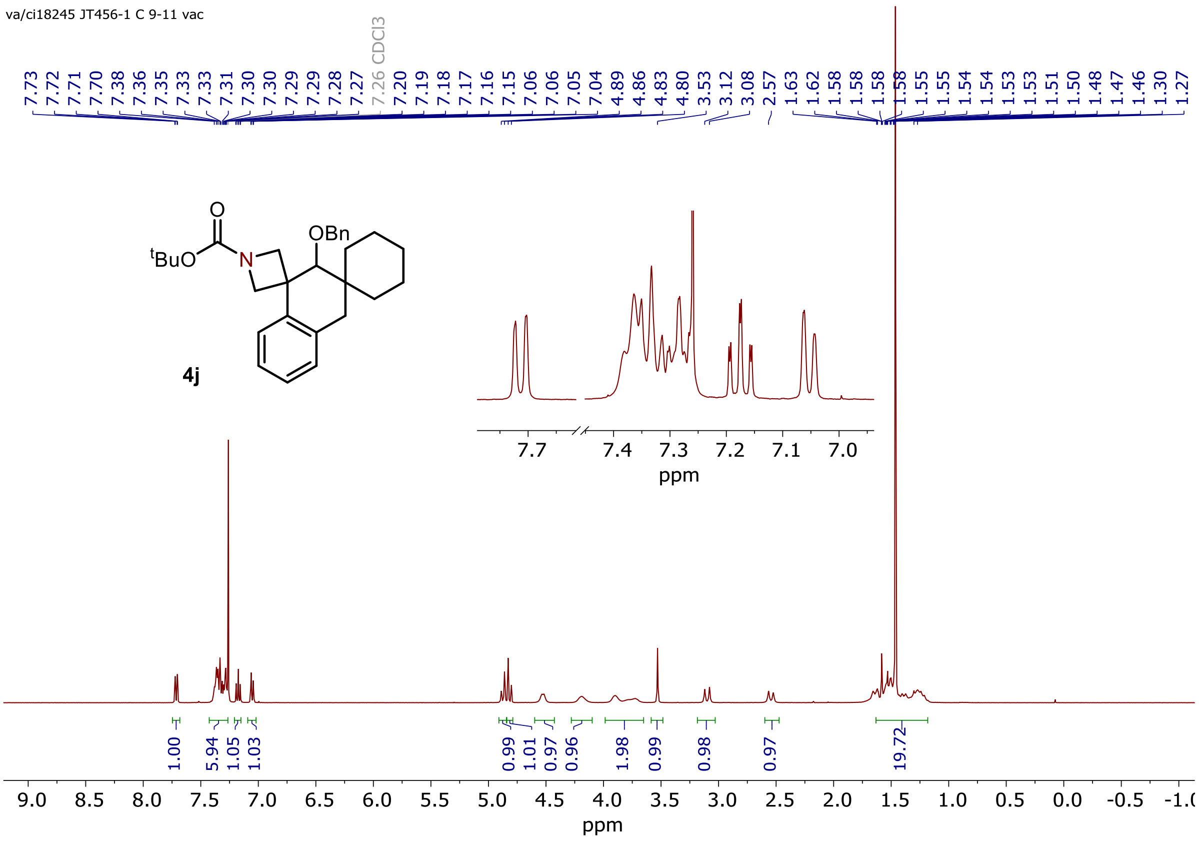

^{13}C NMR (126 MHz, $CDCl_3$) of **4j**

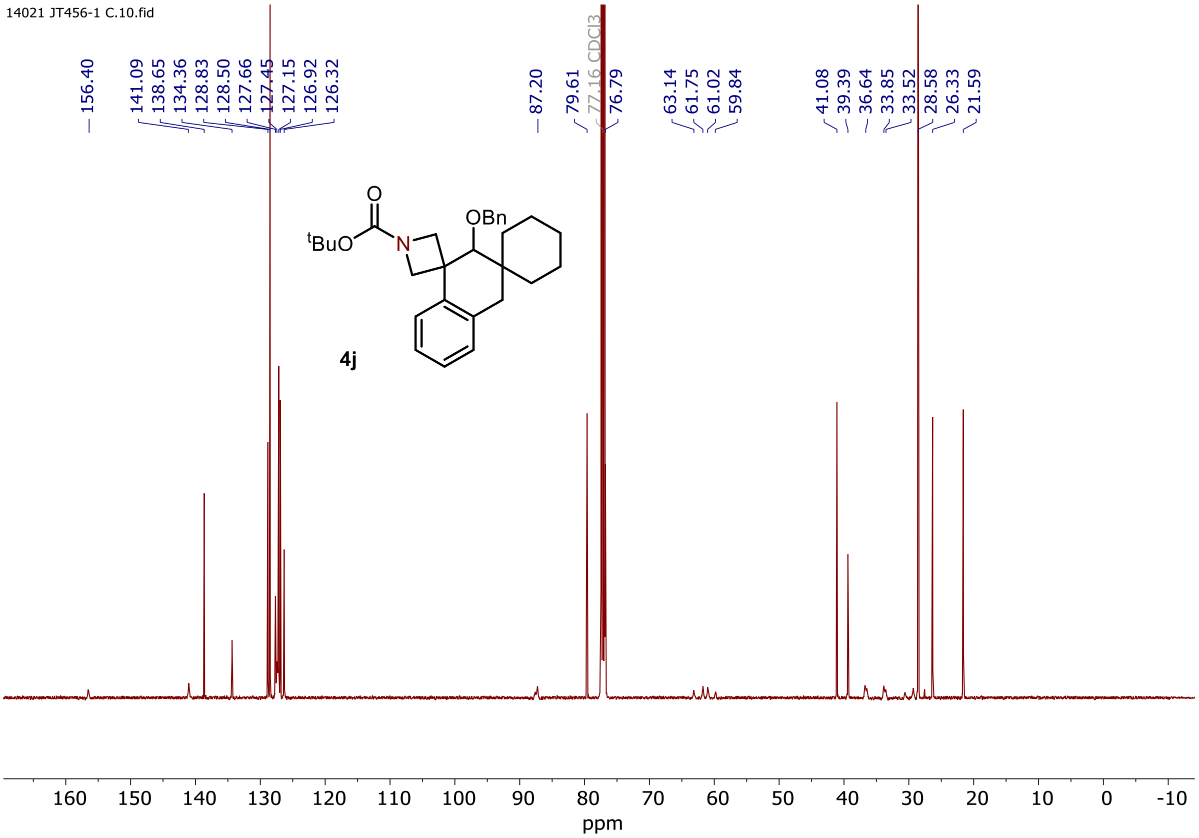

^{1}H NMR (400 MHz, $CDCl_3$) of **4k** (*see procedure*)

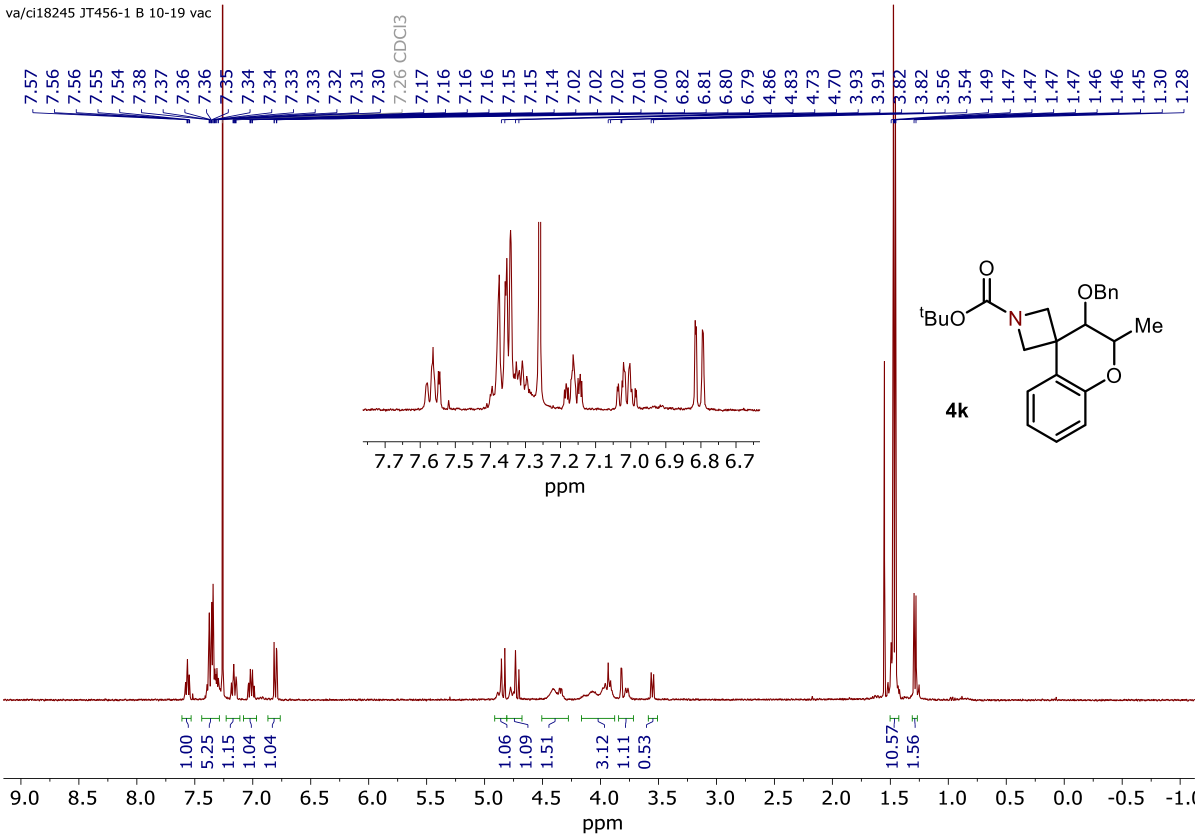

^{13}C NMR (126 MHz, $CDCl_3$) of **4k**

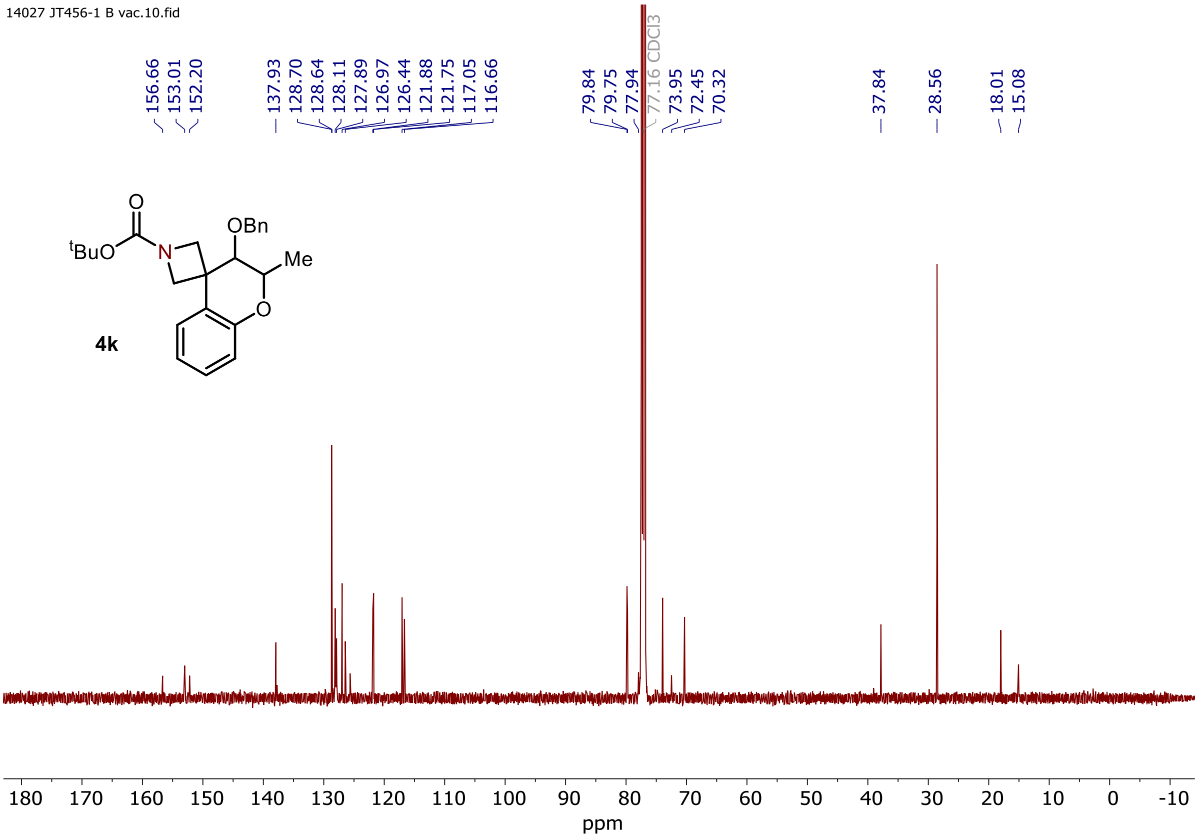

^{1}H NMR (500 MHz, $CDCl_3$) of **4l** (*see procedure*)

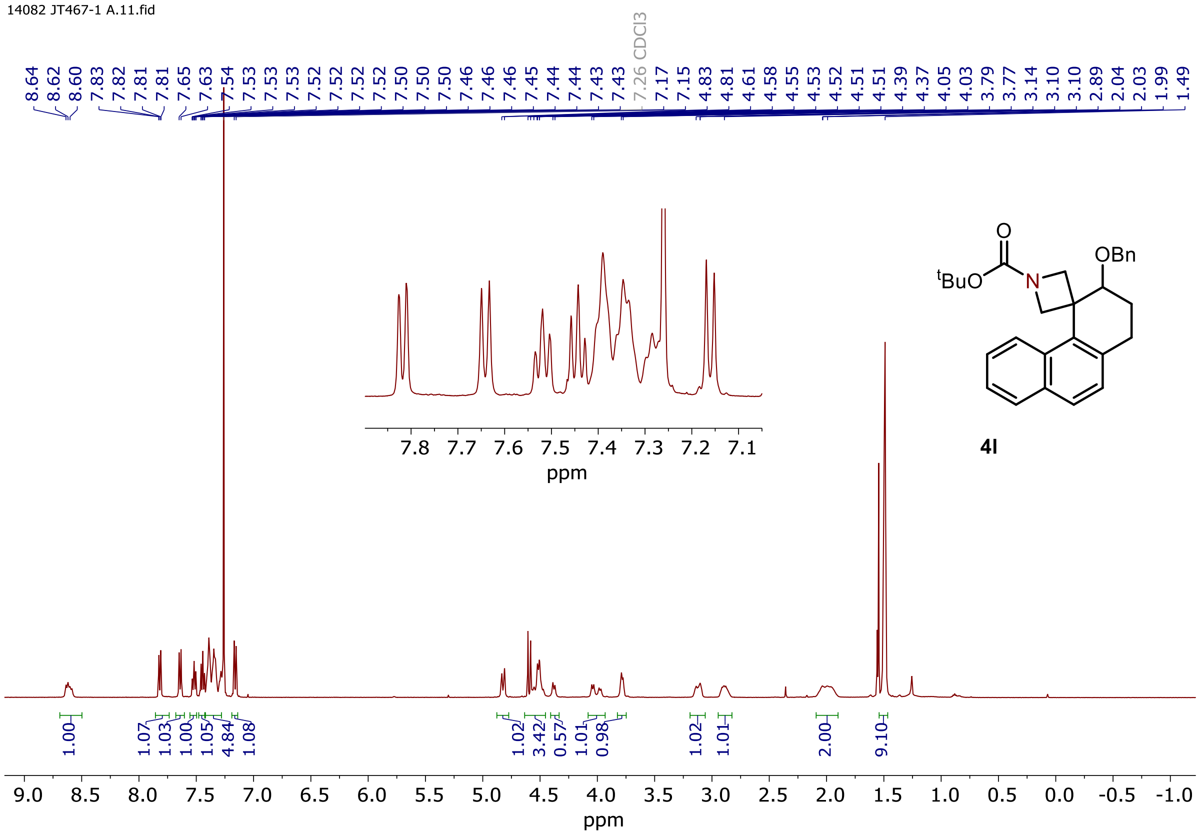

^{13}C NMR (126 MHz, $CDCl_3$) of **4l**

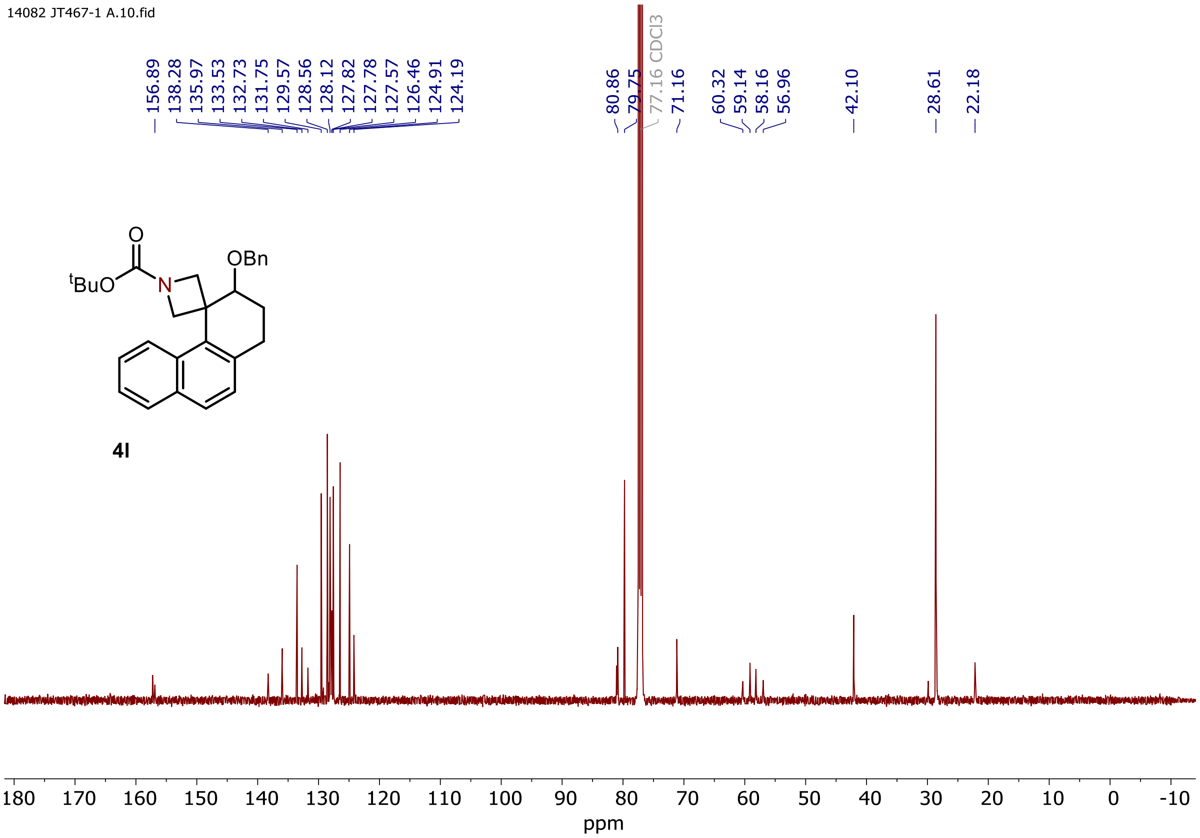

^{1}H NMR (400 MHz, $CDCl_3$) of **4m** (see procedure)

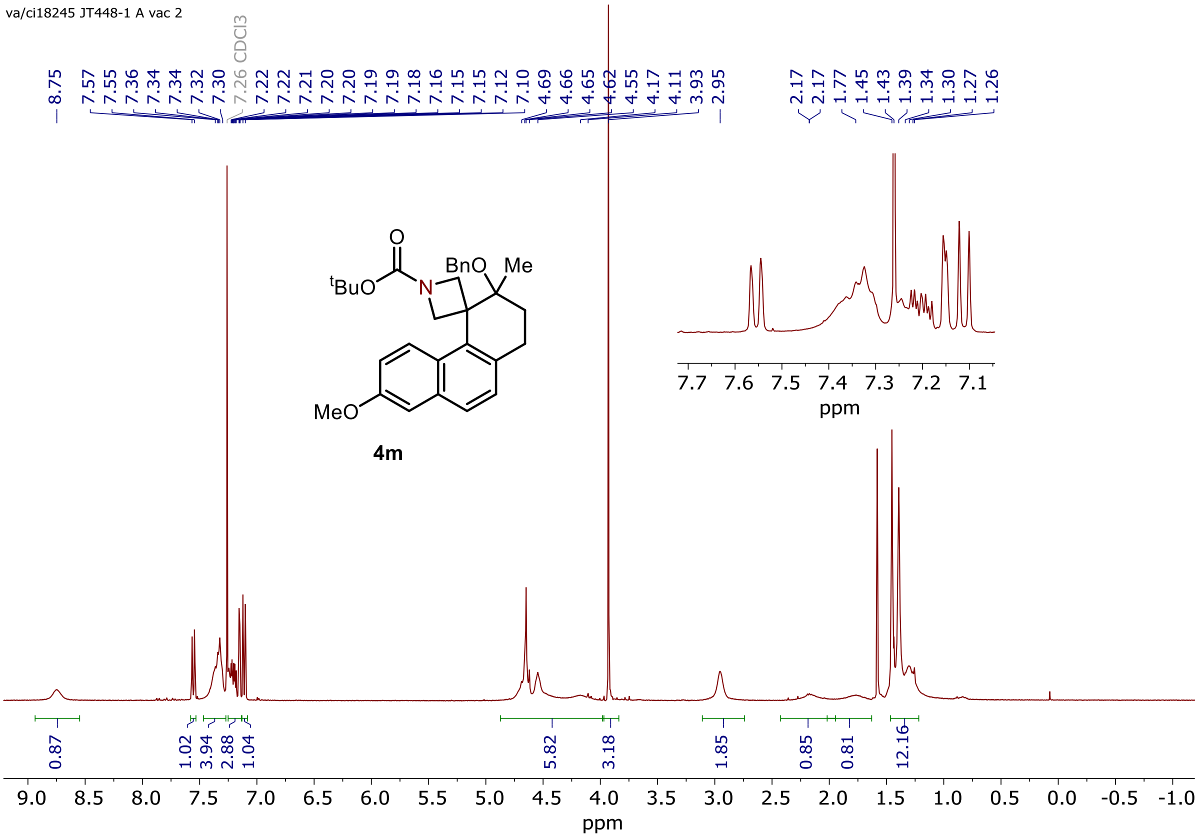

^{13}C NMR (126 MHz, $CDCl_3$) of **4m**

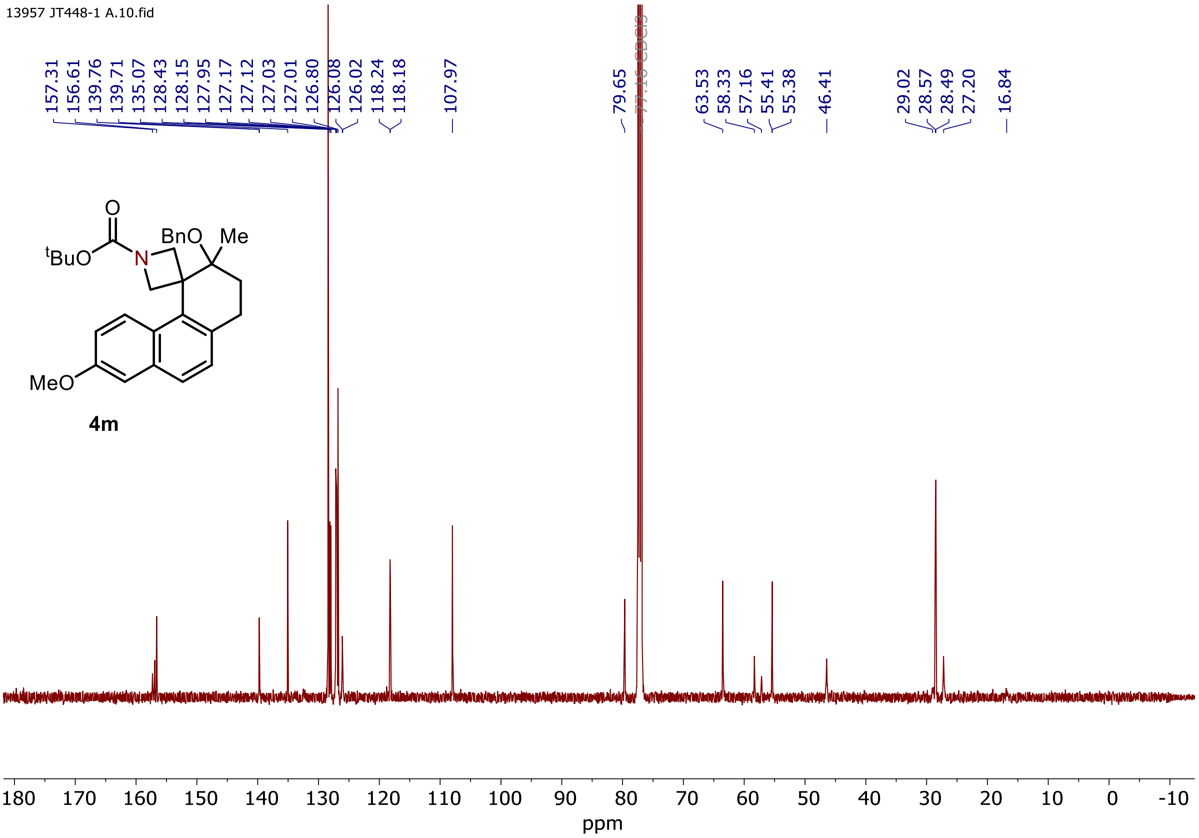

^{1}H NMR (400 MHz, $CDCl_3$) of **4n** (*see procedure*)

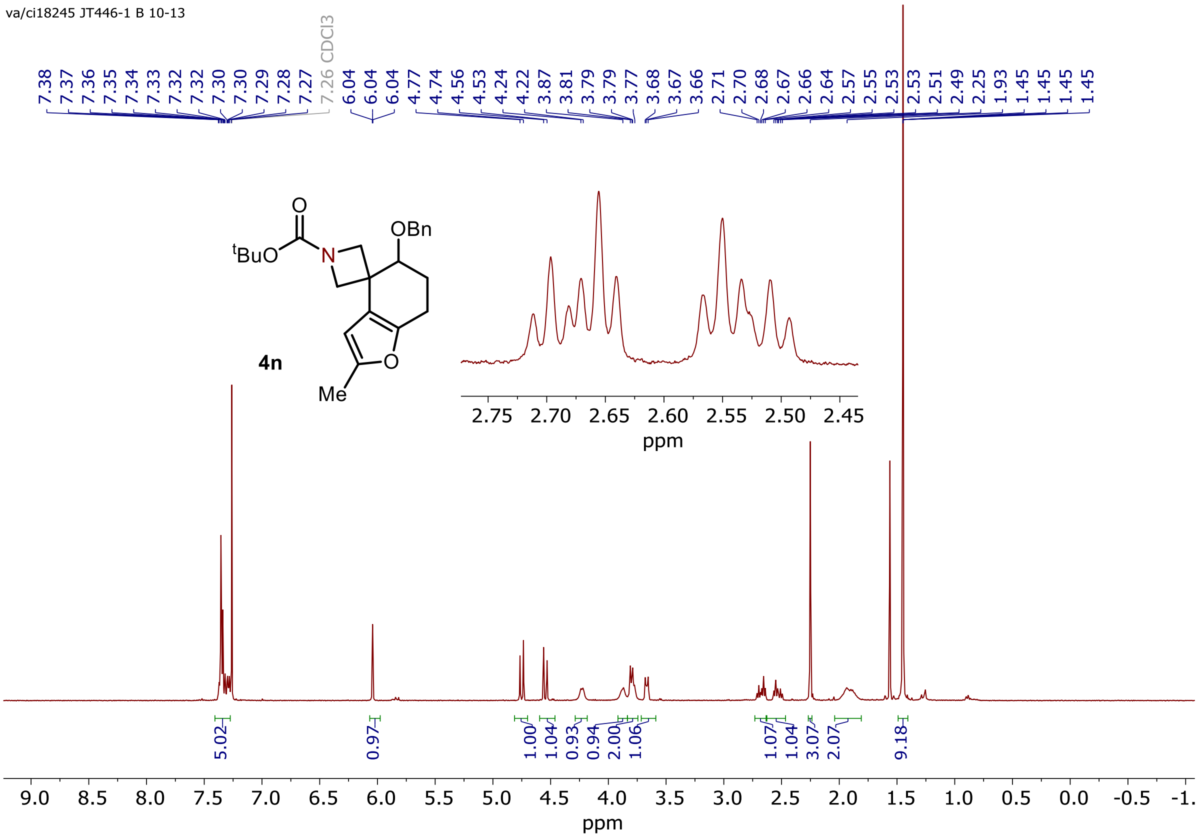

^{13}C NMR (126 MHz, $CDCl_3$) of **4n**

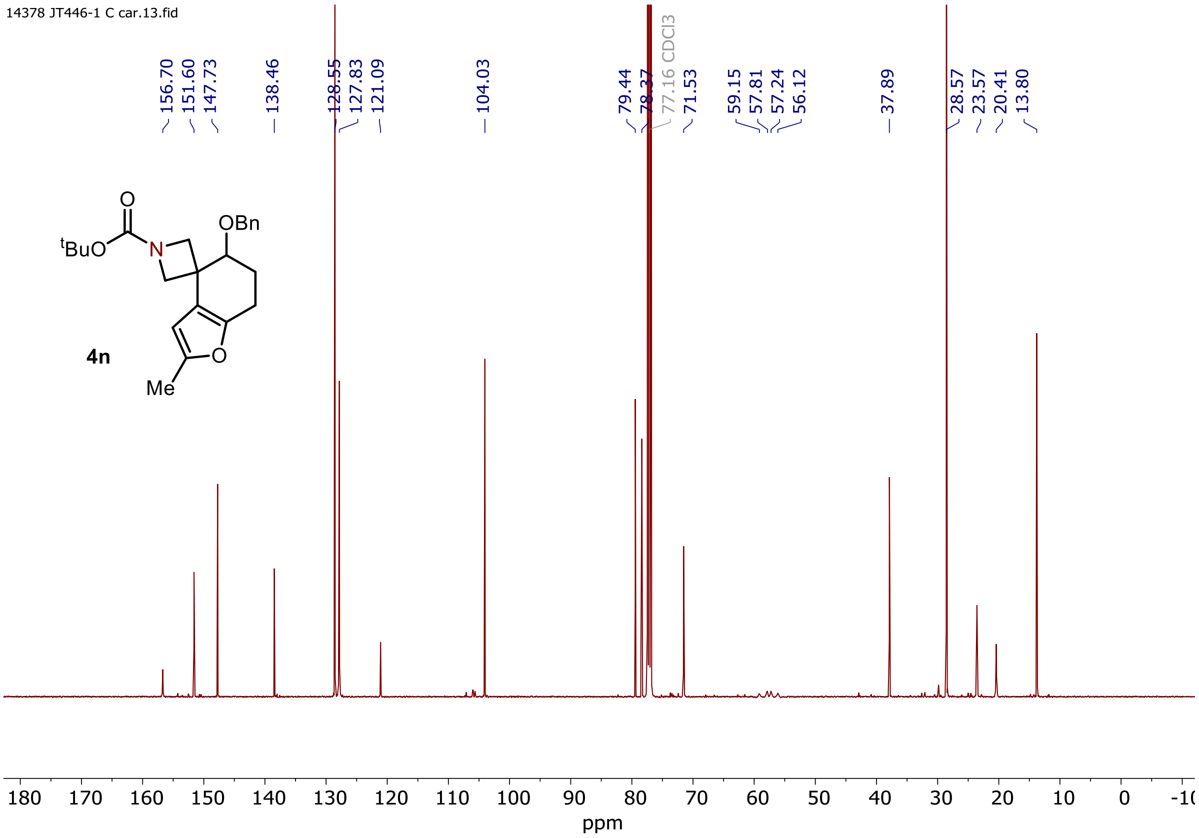

^{1}H NMR (500 MHz, $CDCl_3$) of **4o** (*see procedure*)

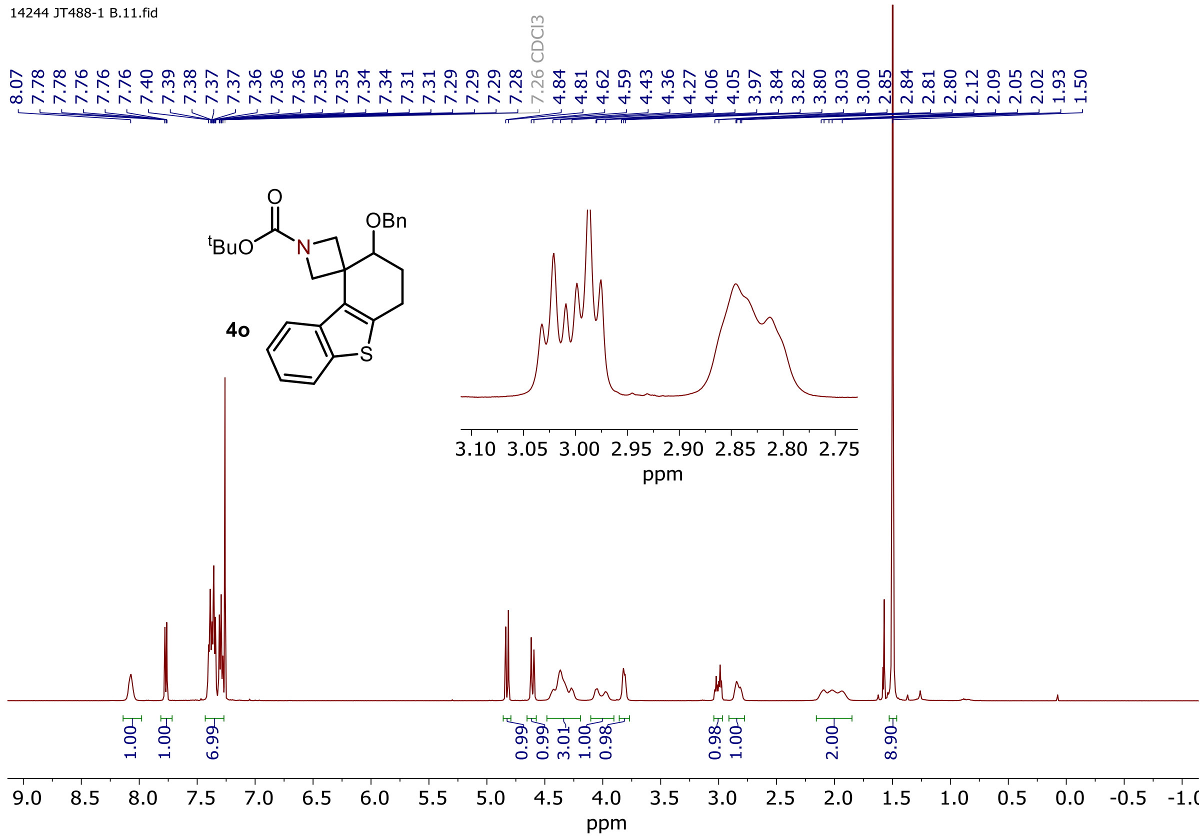

^{13}C NMR (126 MHz, $CDCl_3$) of **4o**

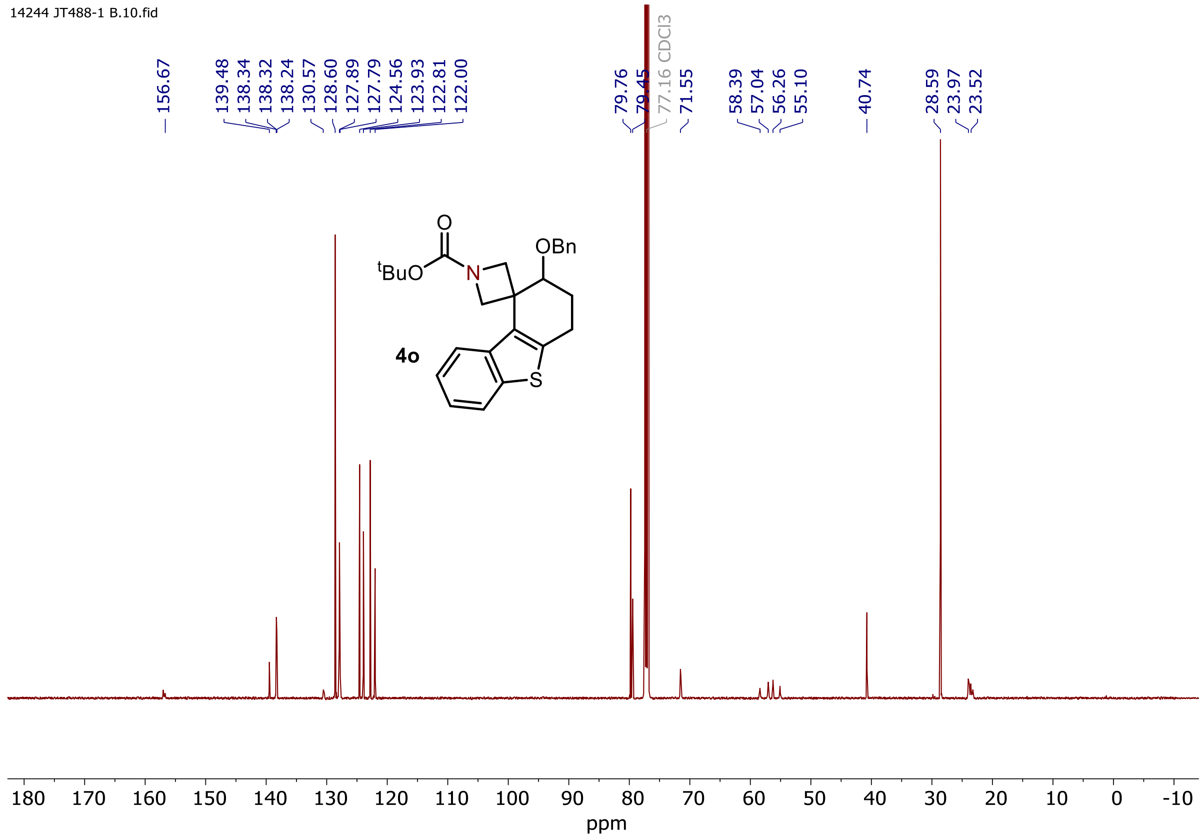

^{1}H NMR (500 MHz, $CDCl_3$) of **4p** (*see procedure*)

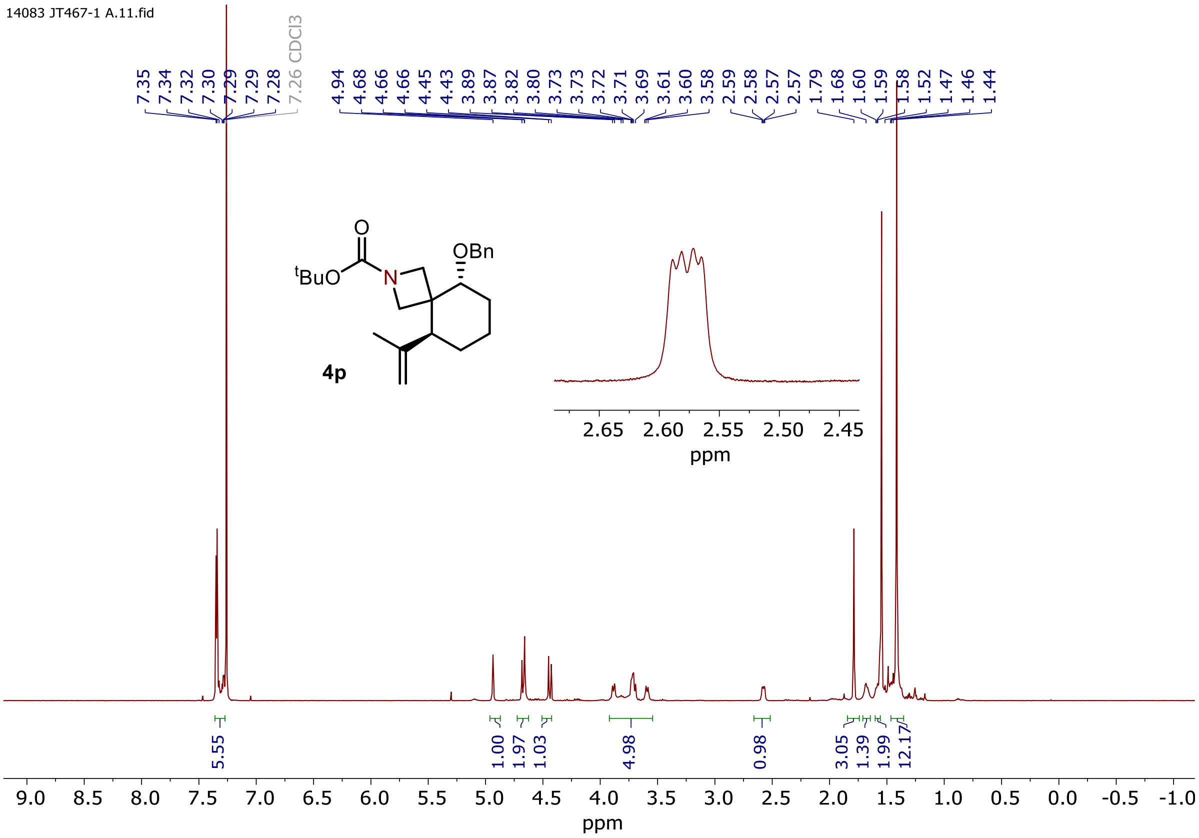

^{13}C NMR (126 MHz, $CDCl_3$) of **4p**

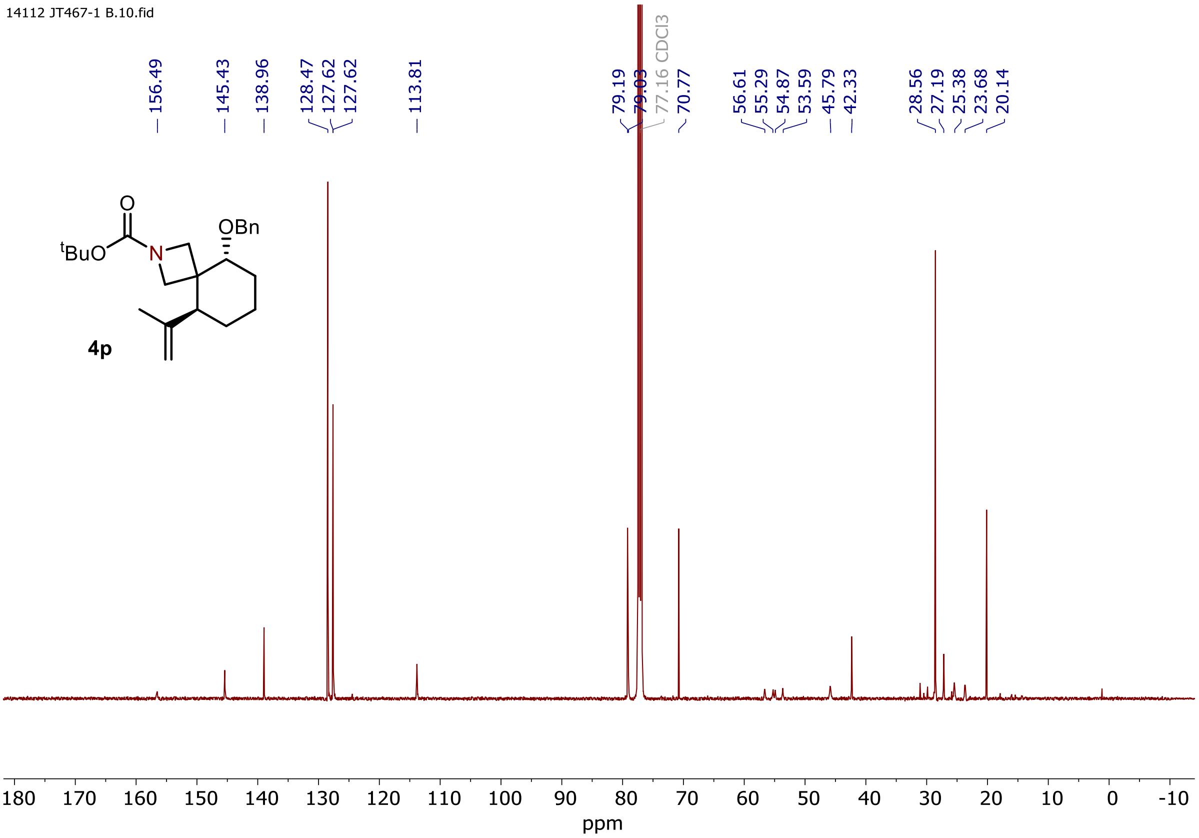

^{1}H NMR (500 MHz, $CDCl_3$) of **4q** (*see procedure*)

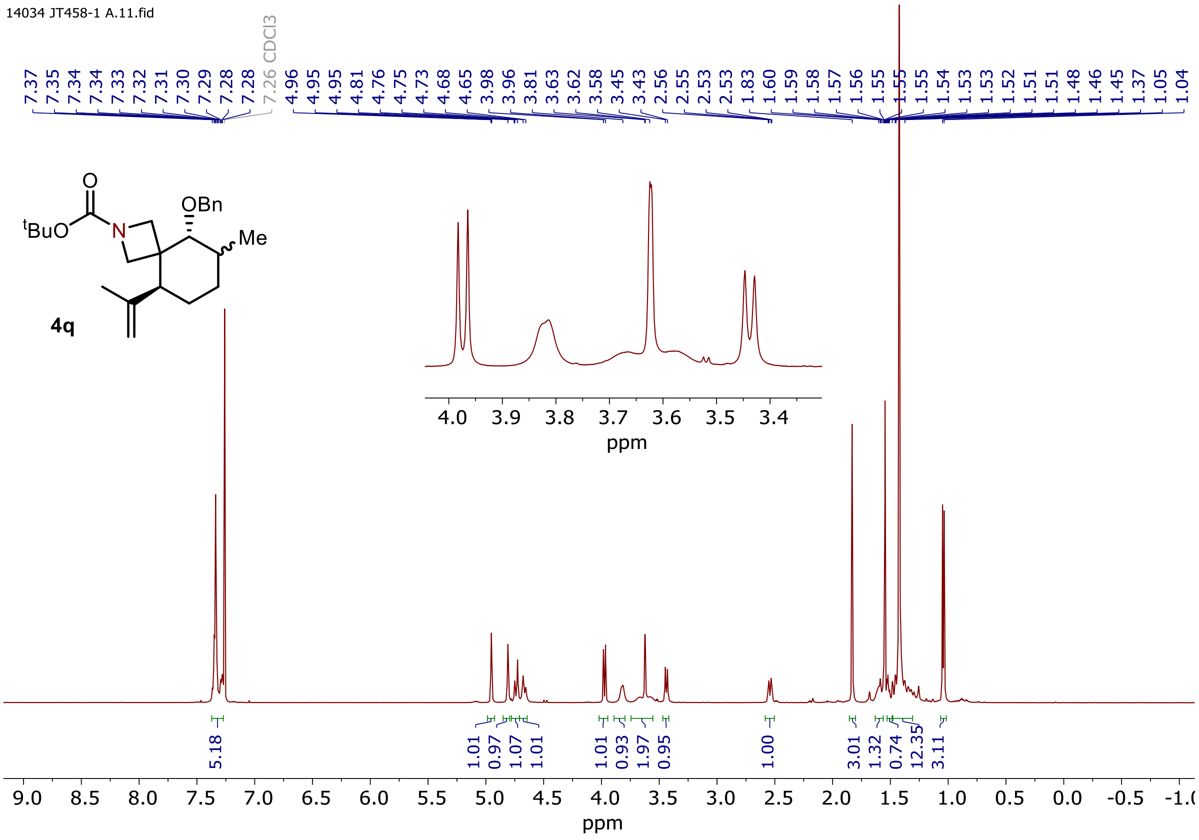

^{13}C NMR (126 MHz, $CDCl_3$) of **4q**

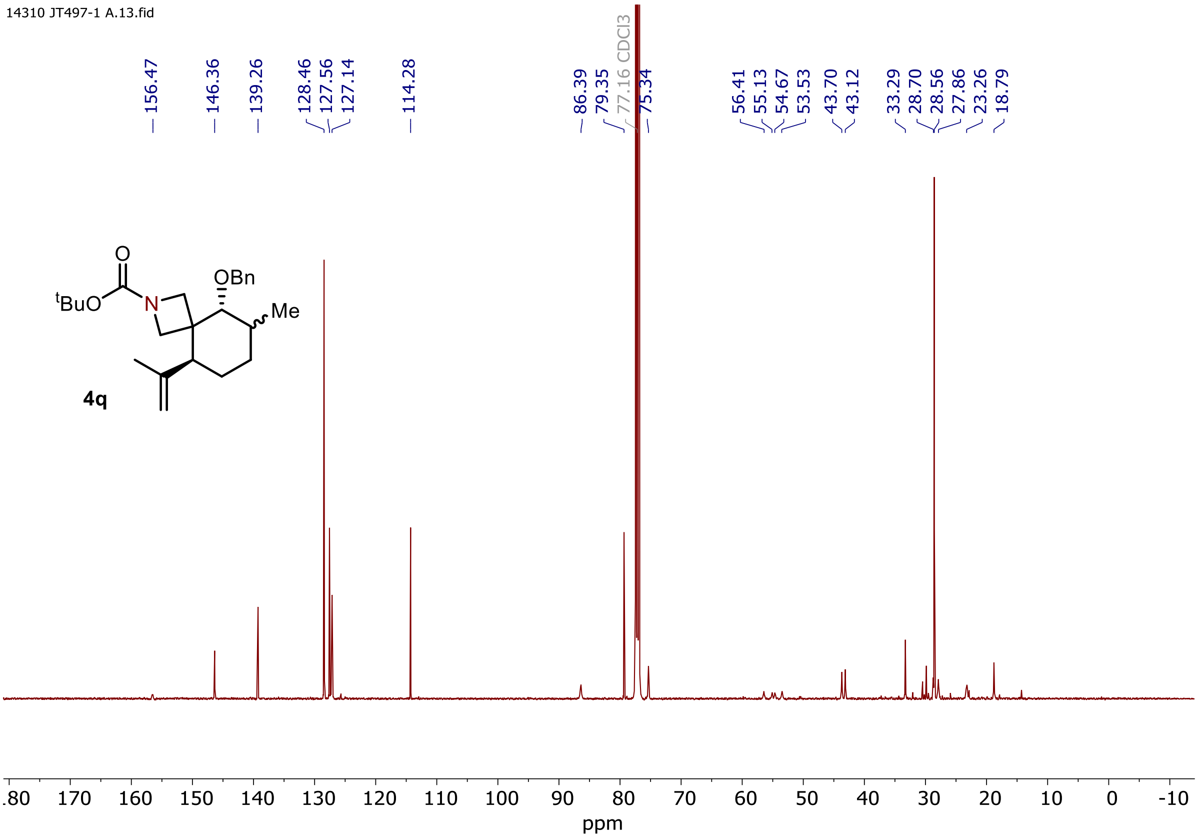

^{1}H NMR (500 MHz, $CDCl_3$) of **4r** (*see procedure*)

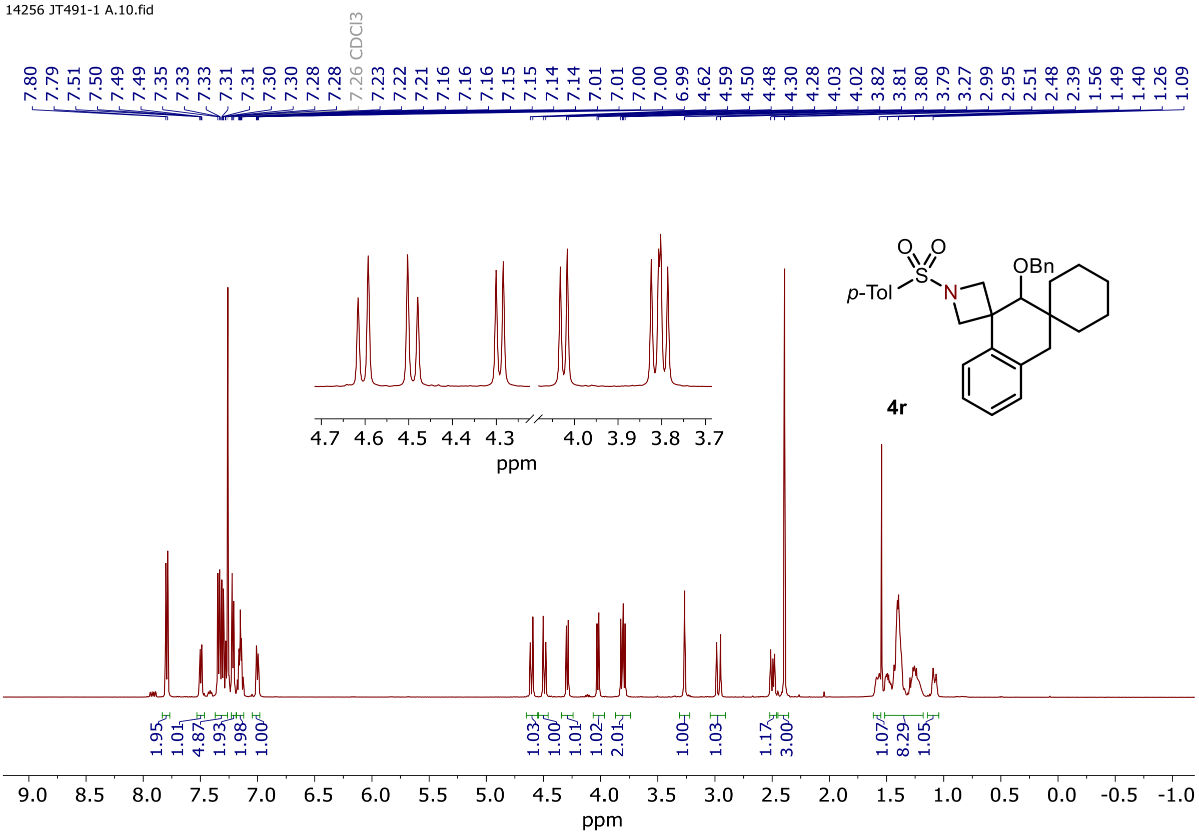

^{13}C NMR (126 MHz, $CDCl_3$) of **4r**

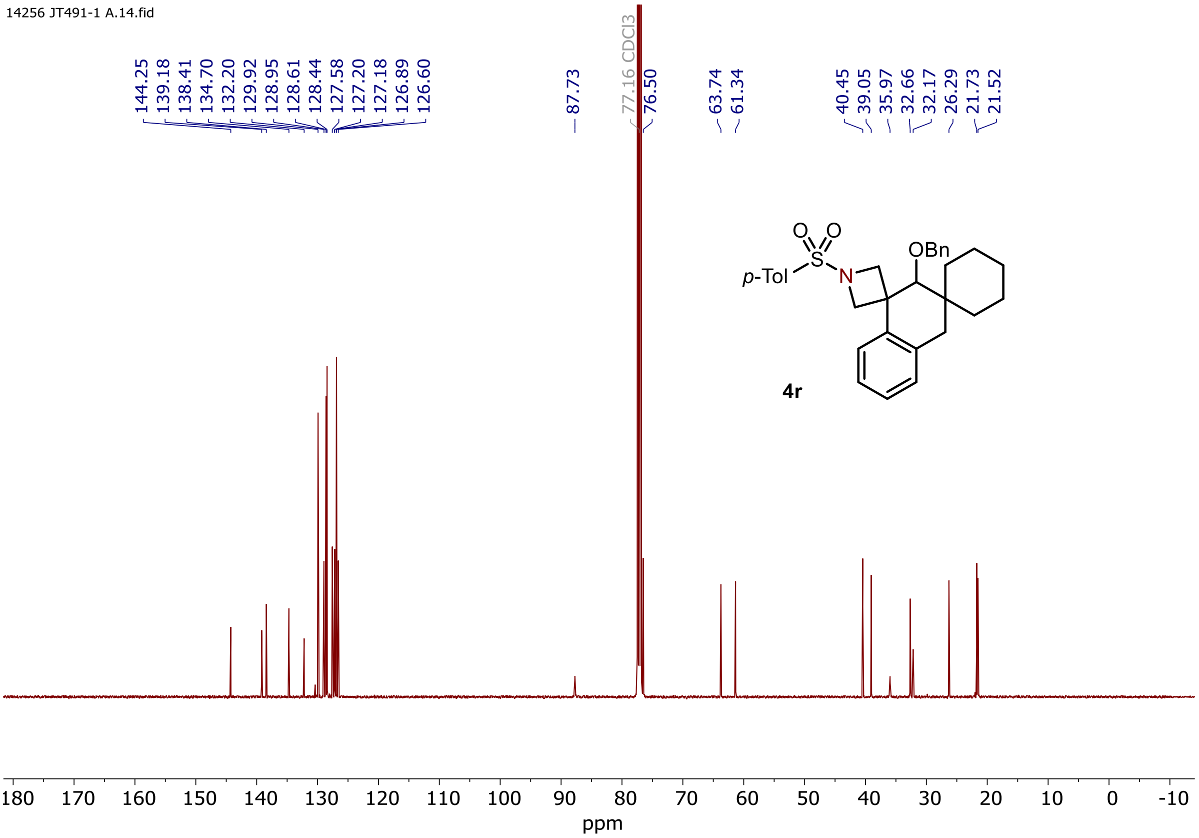

^{1}H NMR (500 MHz, $CDCl_3$) of **4s** (*see procedure*)

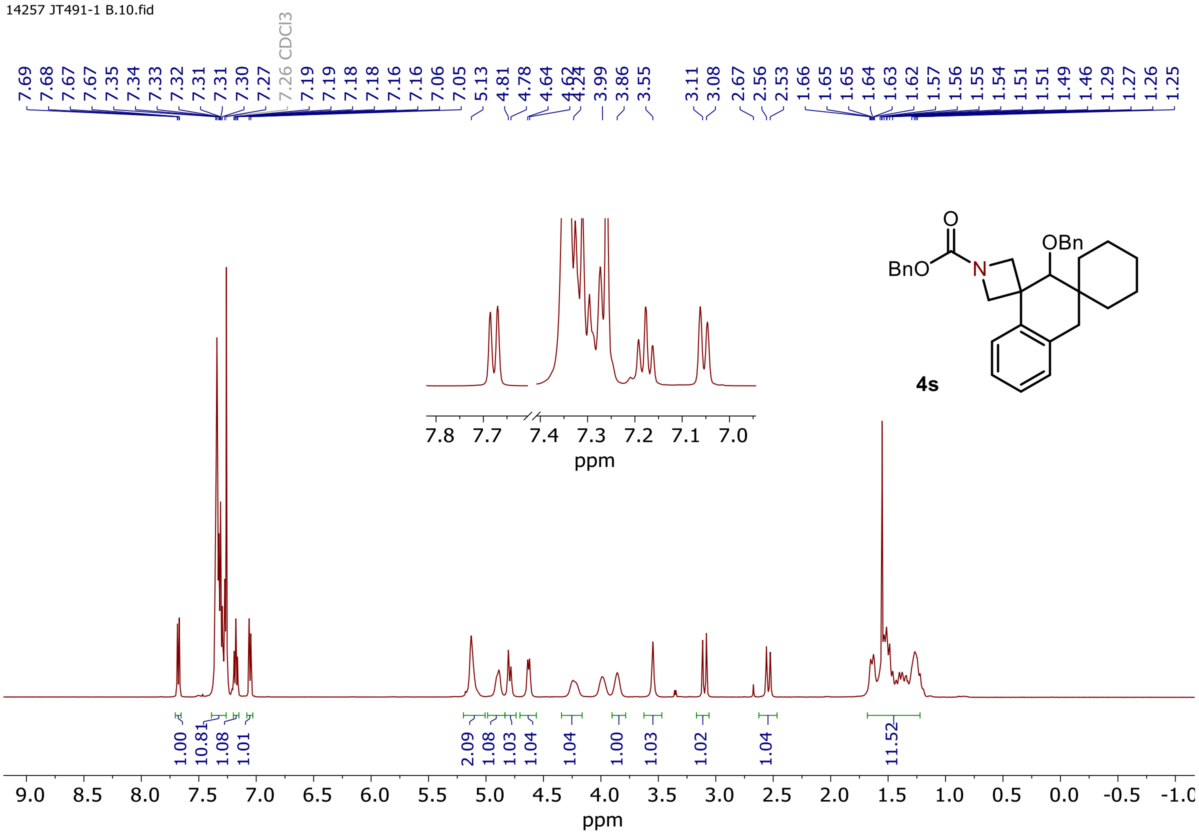

^{13}C NMR (126 MHz, $CDCl_3$) of **4s**

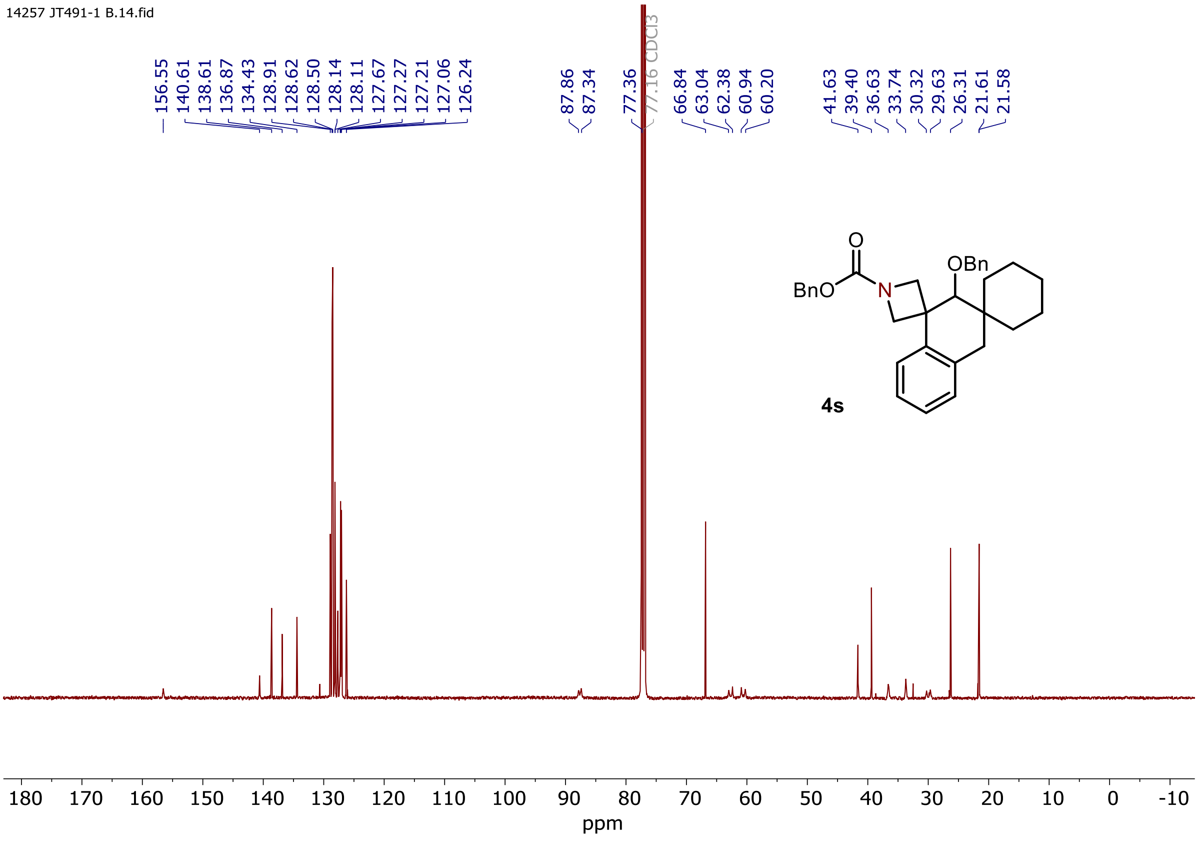

^{1}H NMR (500 MHz, $CDCl_3$) of **4t** (*see procedure*)

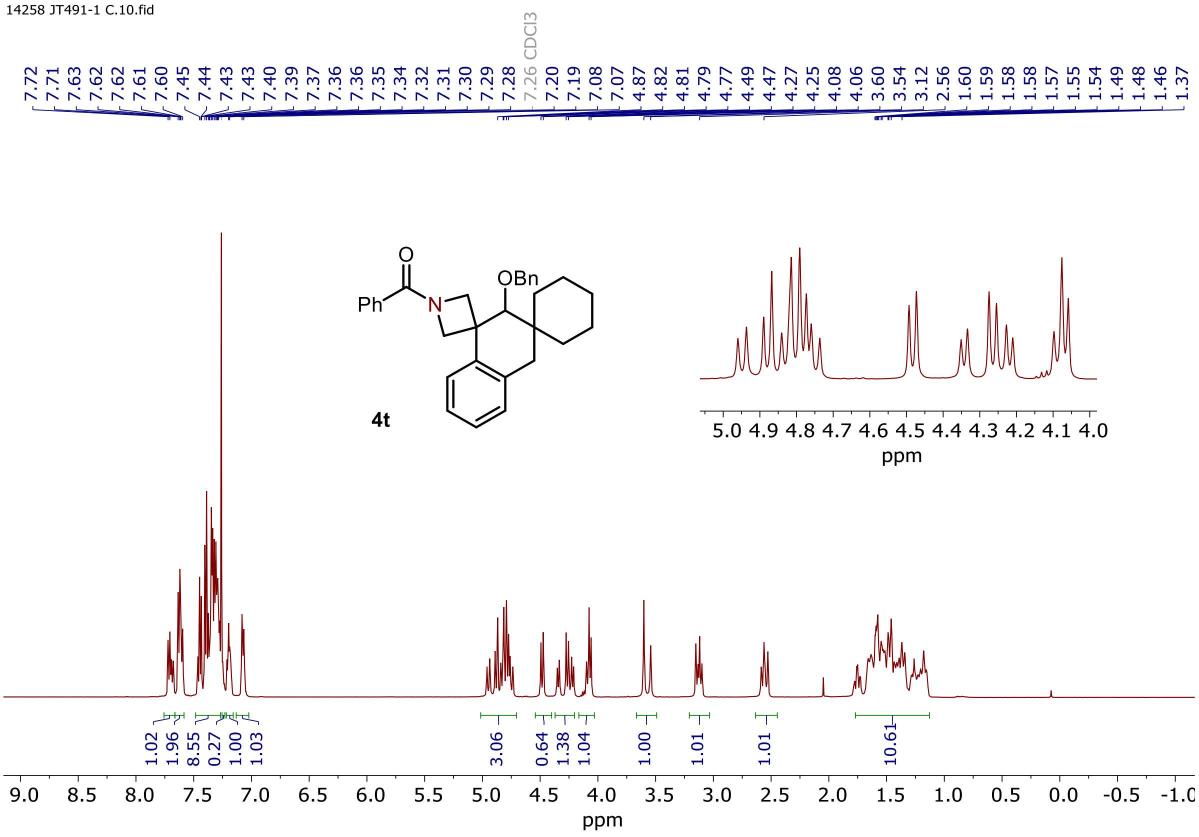

^{13}C NMR (126 MHz, $CDCl_3$) of **4t**

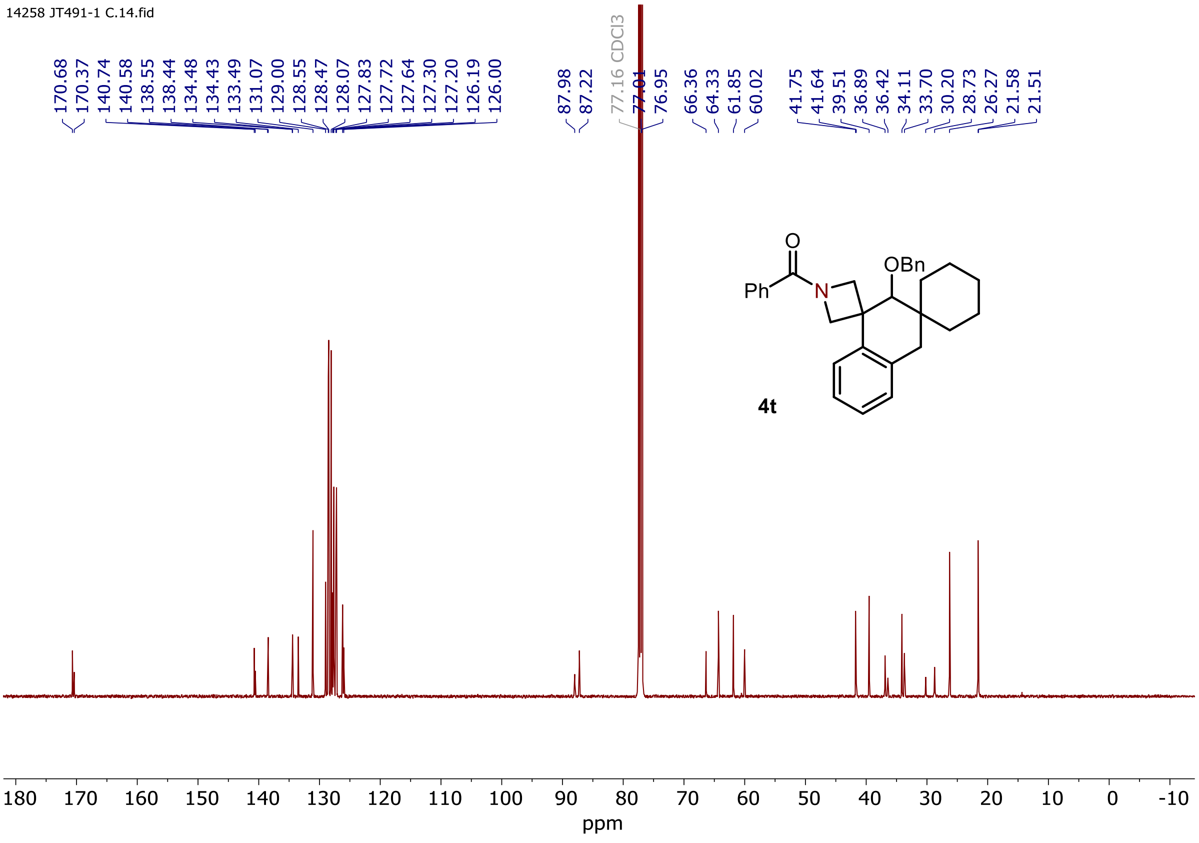

^{1}H NMR (500 MHz, $CDCl_3$) of **4u** (*see procedure*)

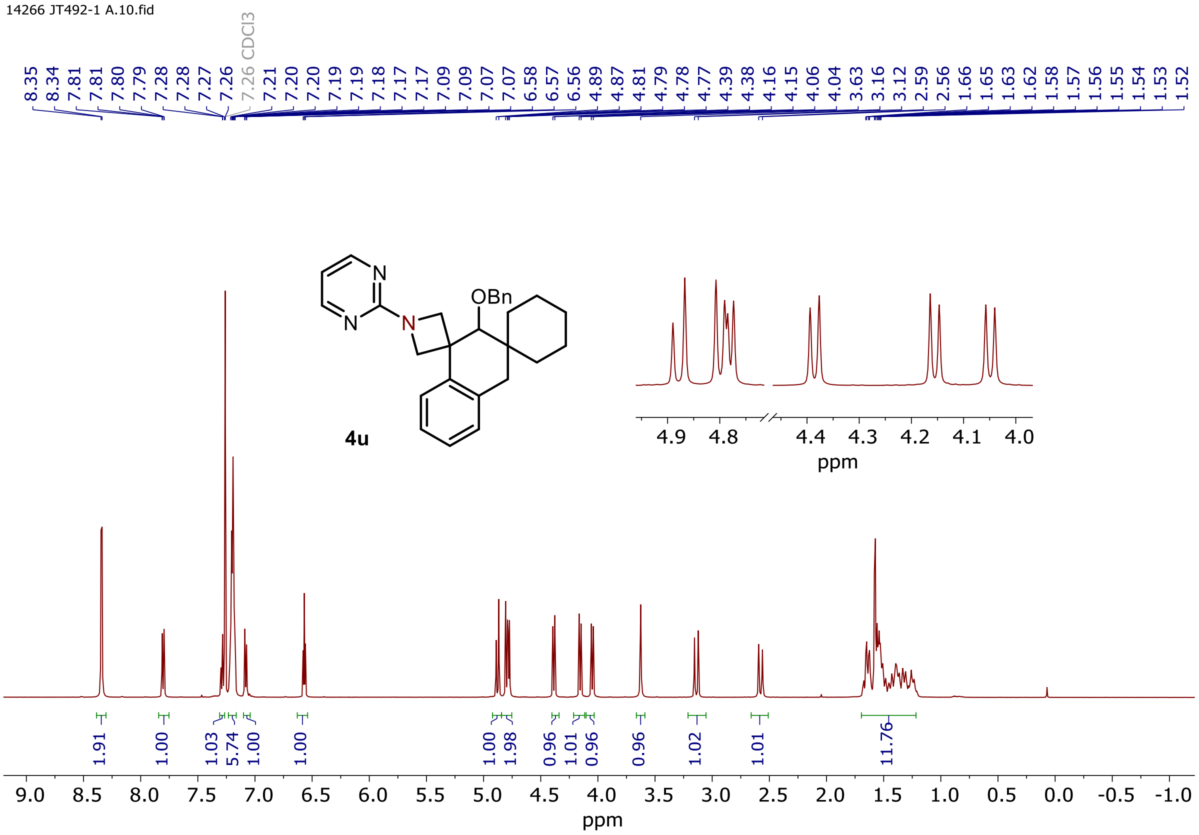

^{13}C NMR (126 MHz, $CDCl_3$) of **4u**

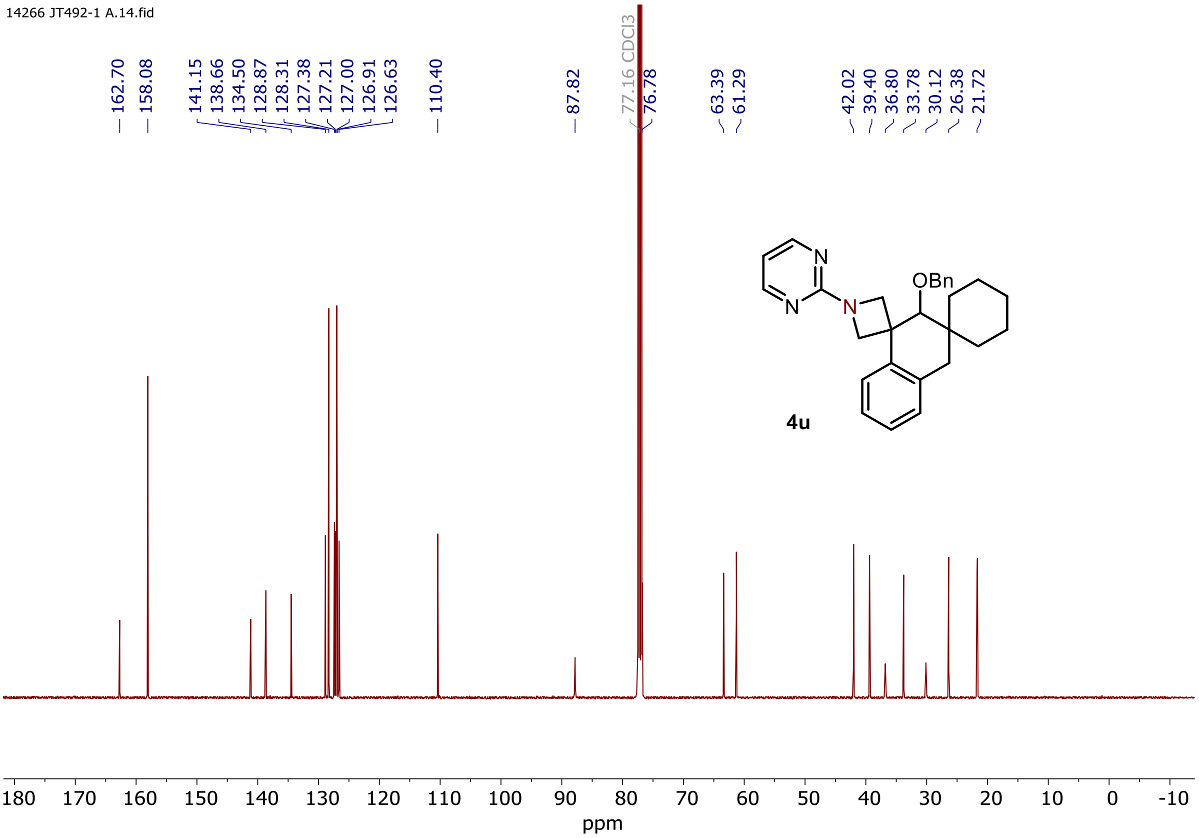

^{1}H NMR (400 MHz, $CDCl_3$) of **4v** (*see procedure*)

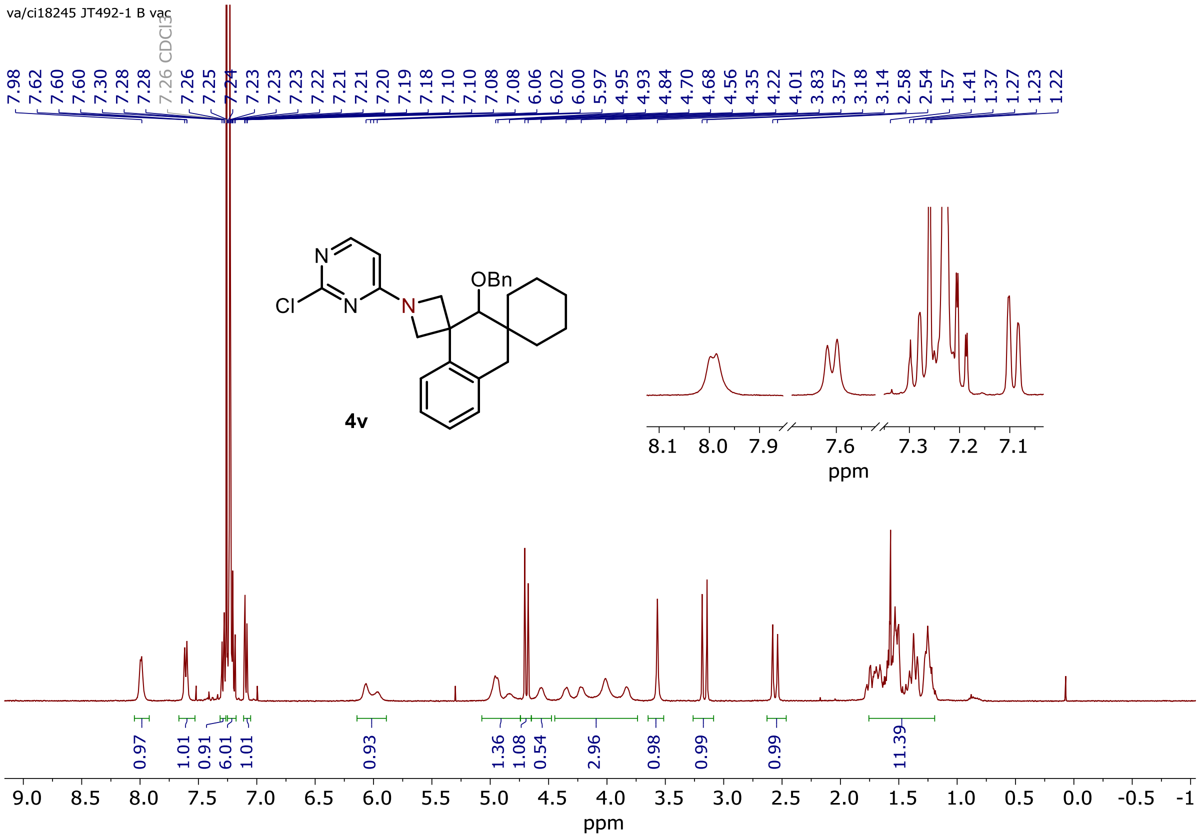

^{13}C NMR (126 MHz, $CDCl_3$) of **4v**

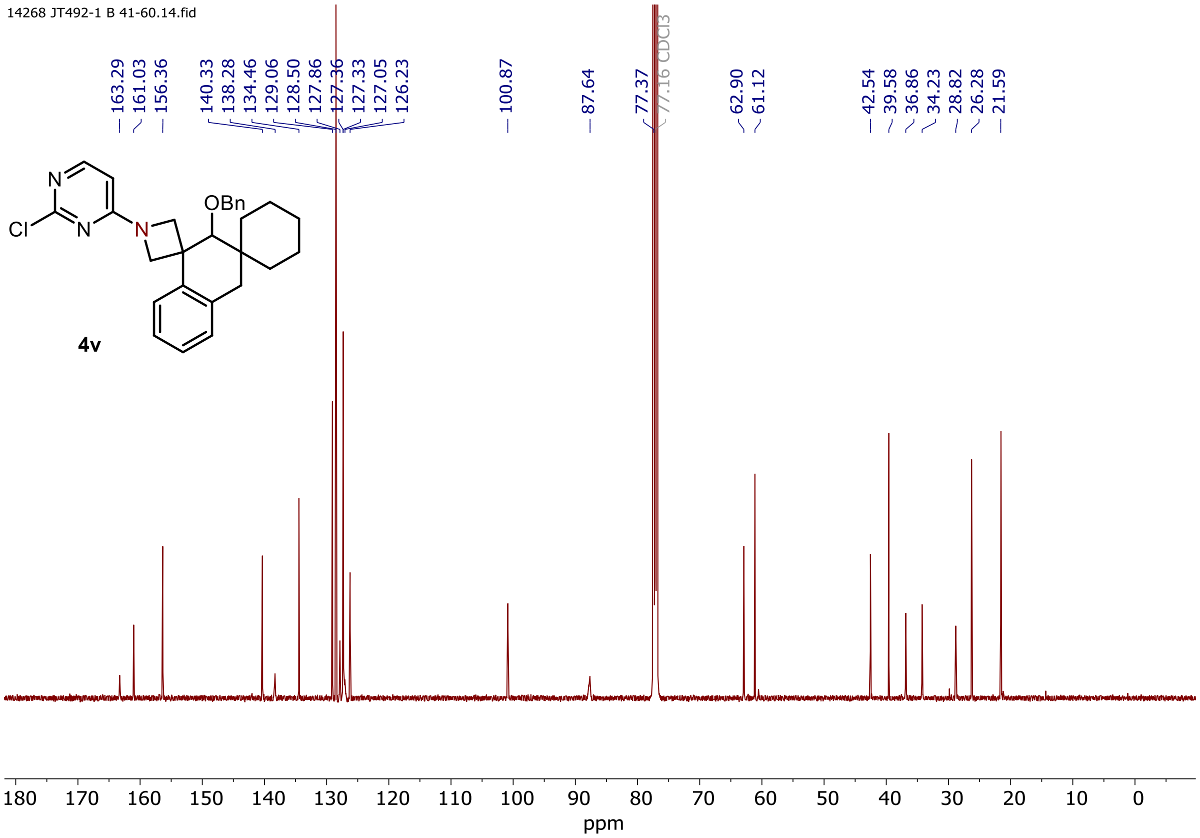

^{1}H NMR (400 MHz, $CDCl_3$) of **4w** (*see procedure*)

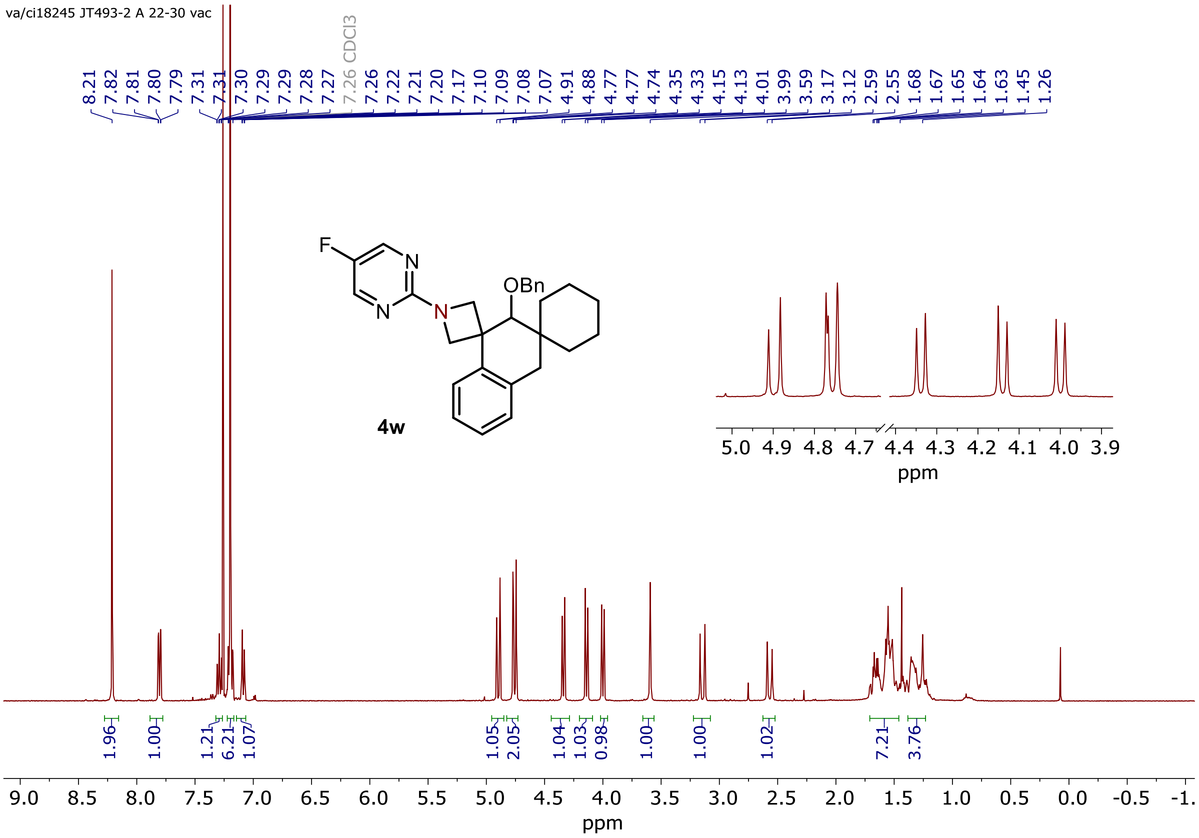

^{13}C NMR (126 MHz, $CDCl_3$) of **4w**

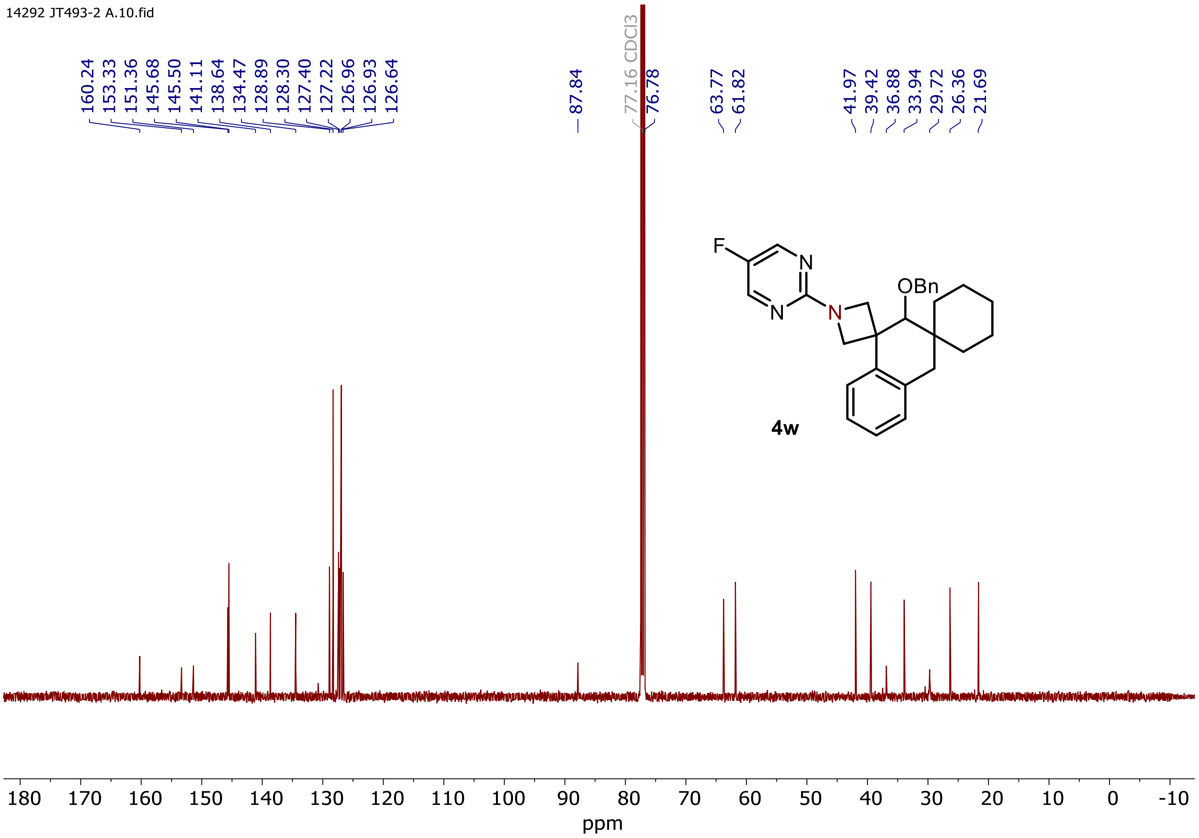

^{19}F NMR (376 MHz, $CDCl_3$) of **4w**

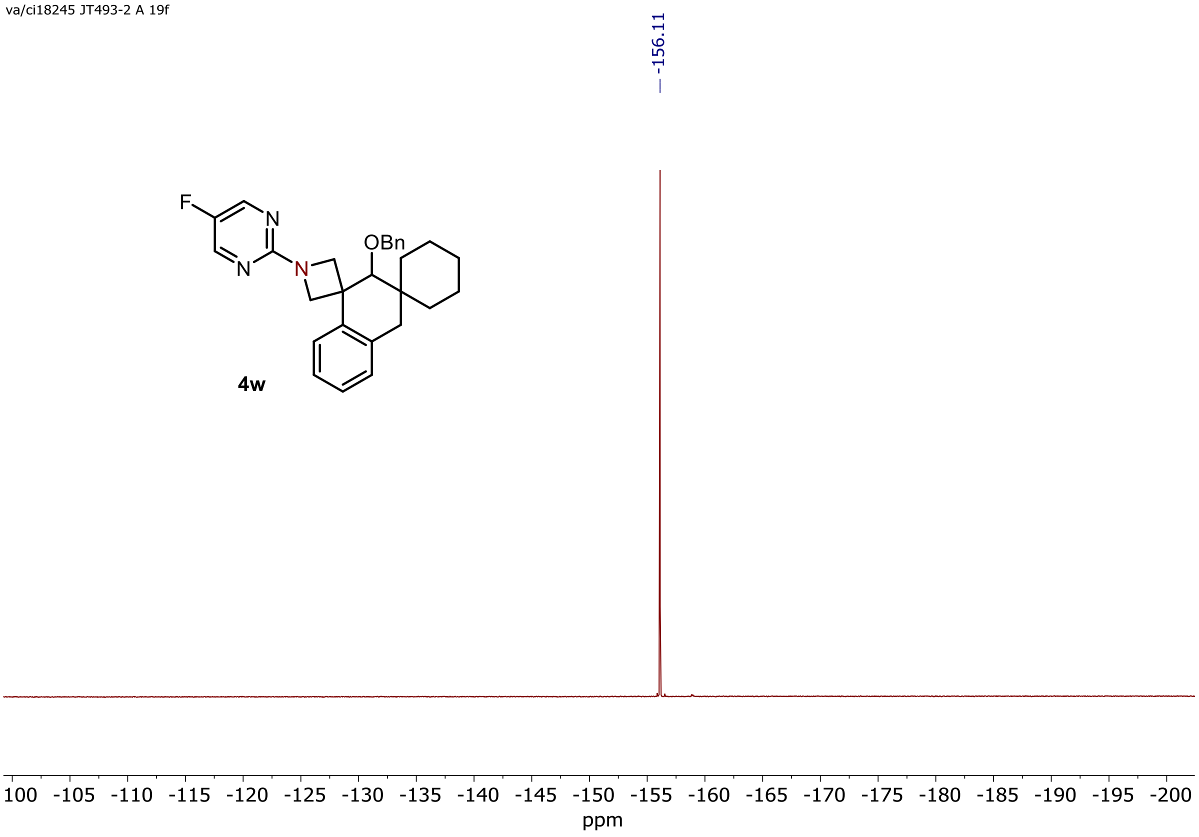

^{1}H NMR (500 MHz, $CDCl_3$) of **4x** (*see procedure*)

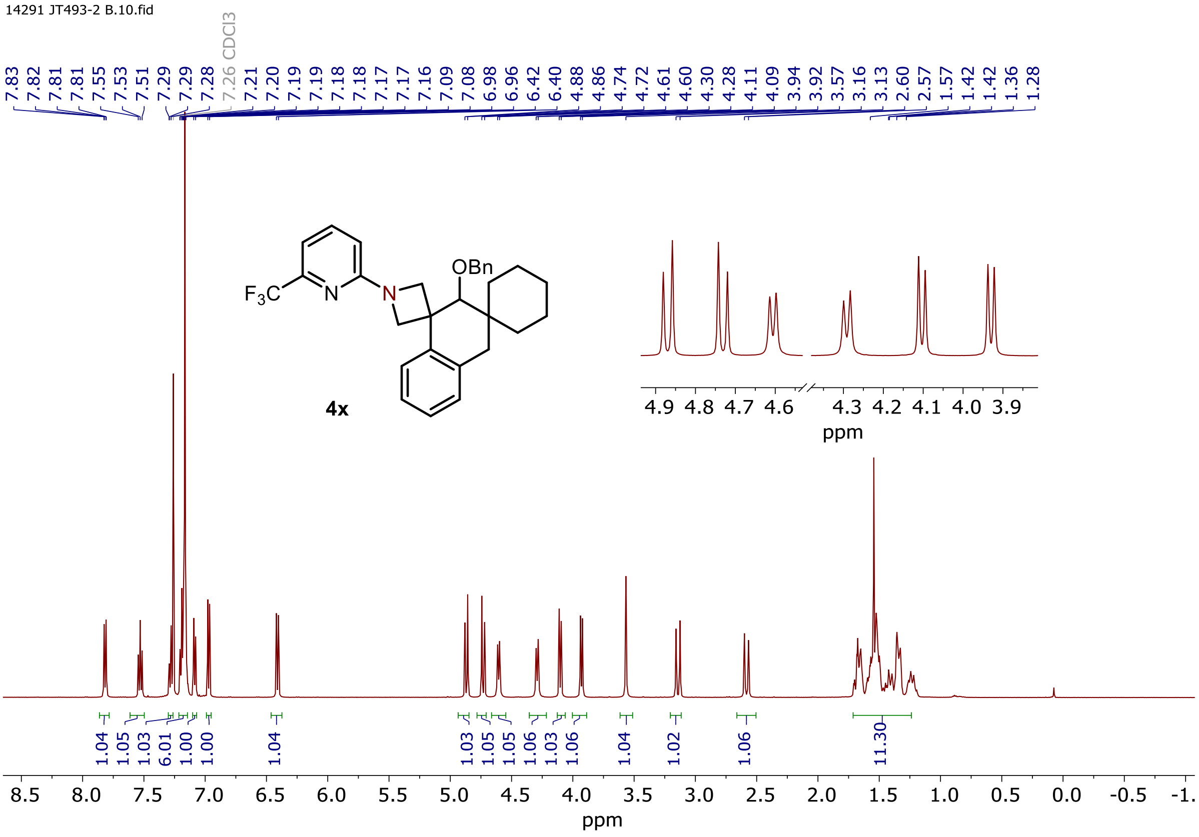

^{13}C NMR (126 MHz, $CDCl_3$) of **4x**

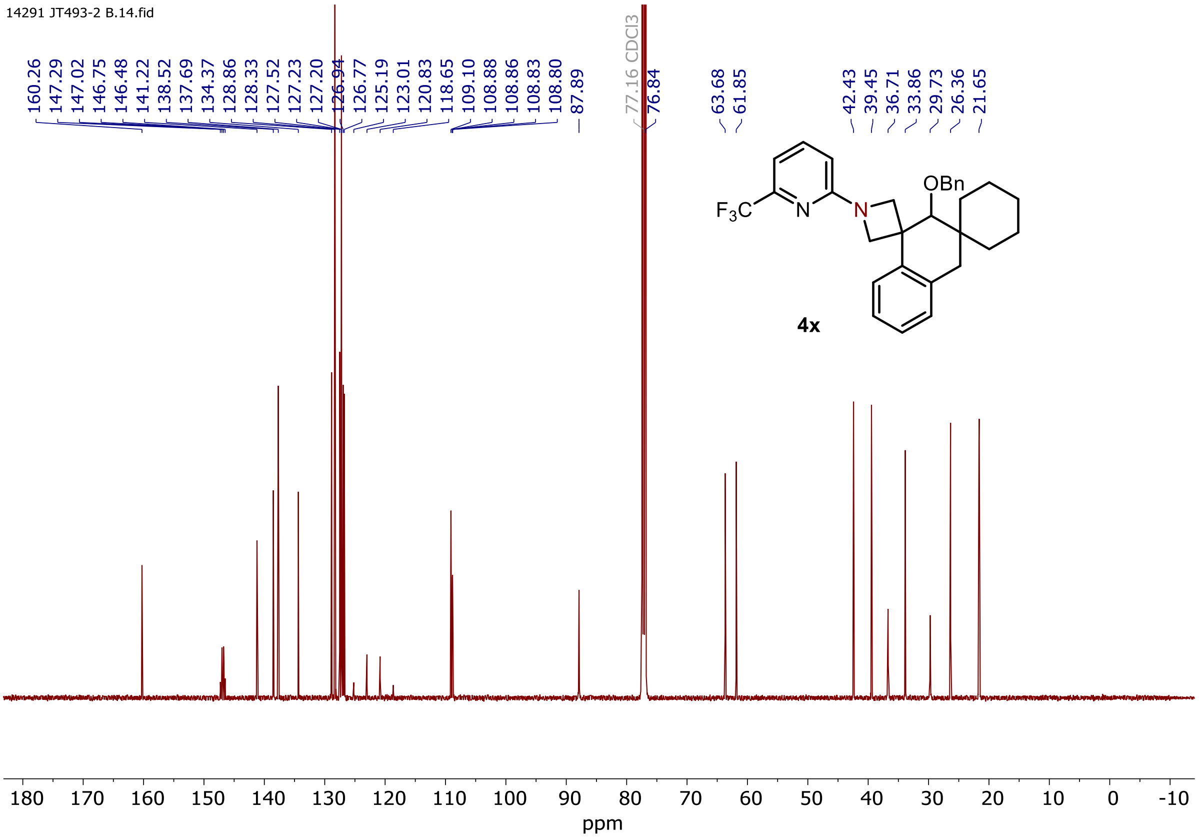

^{19}F NMR (376 MHz, $CDCl_3$) of **4x**

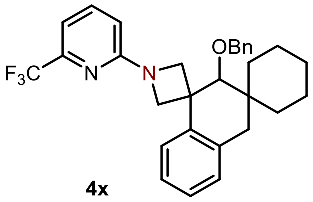

4x

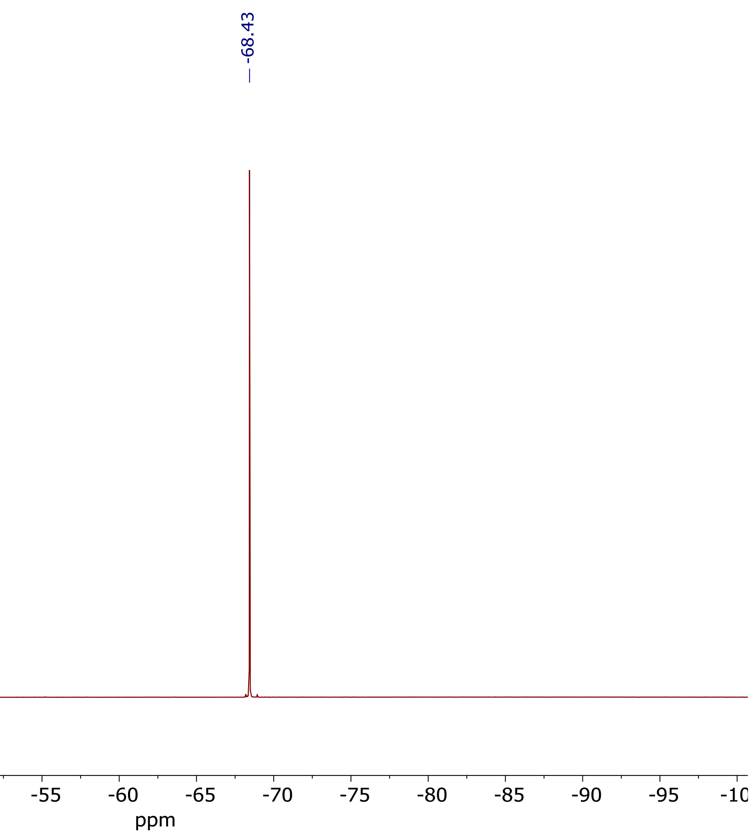

^{1}H NMR (500 MHz, $CDCl_3$) of **4y** (see procedure)

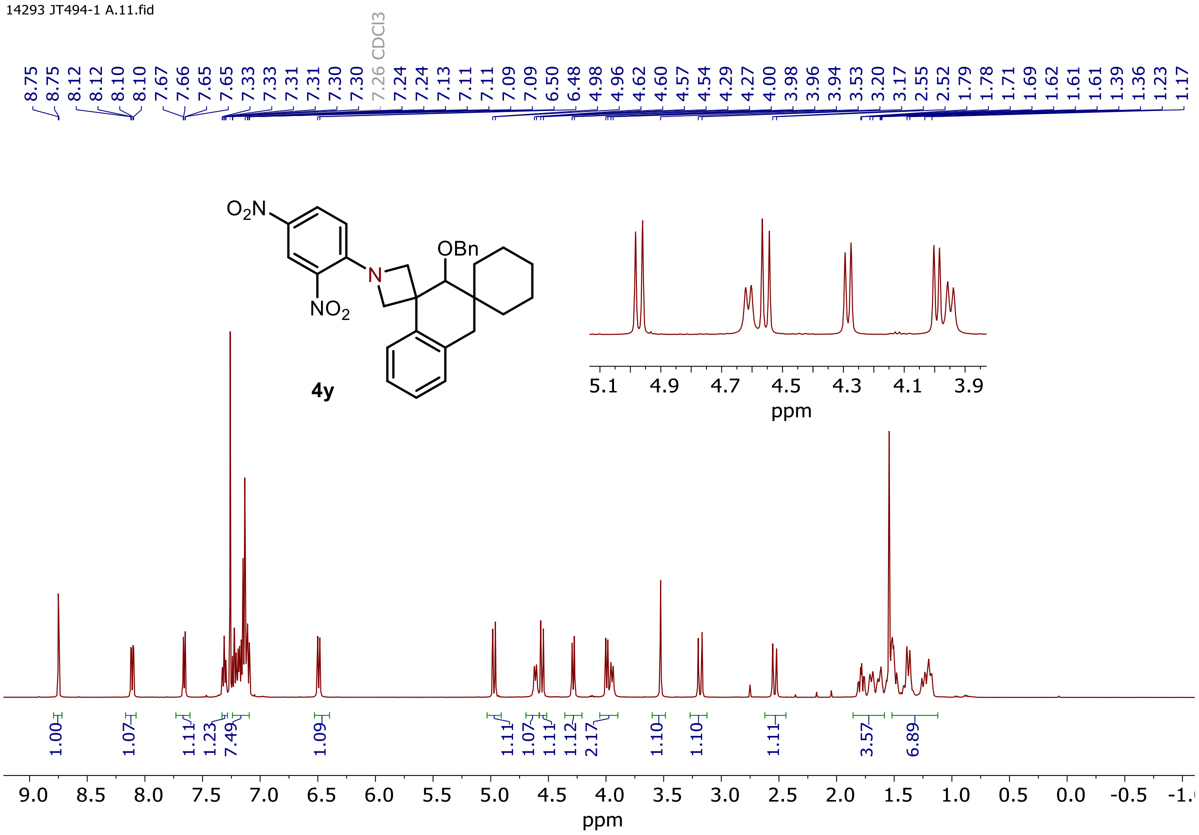

^{13}C NMR (126 MHz, $CDCl_3$) of **4y**

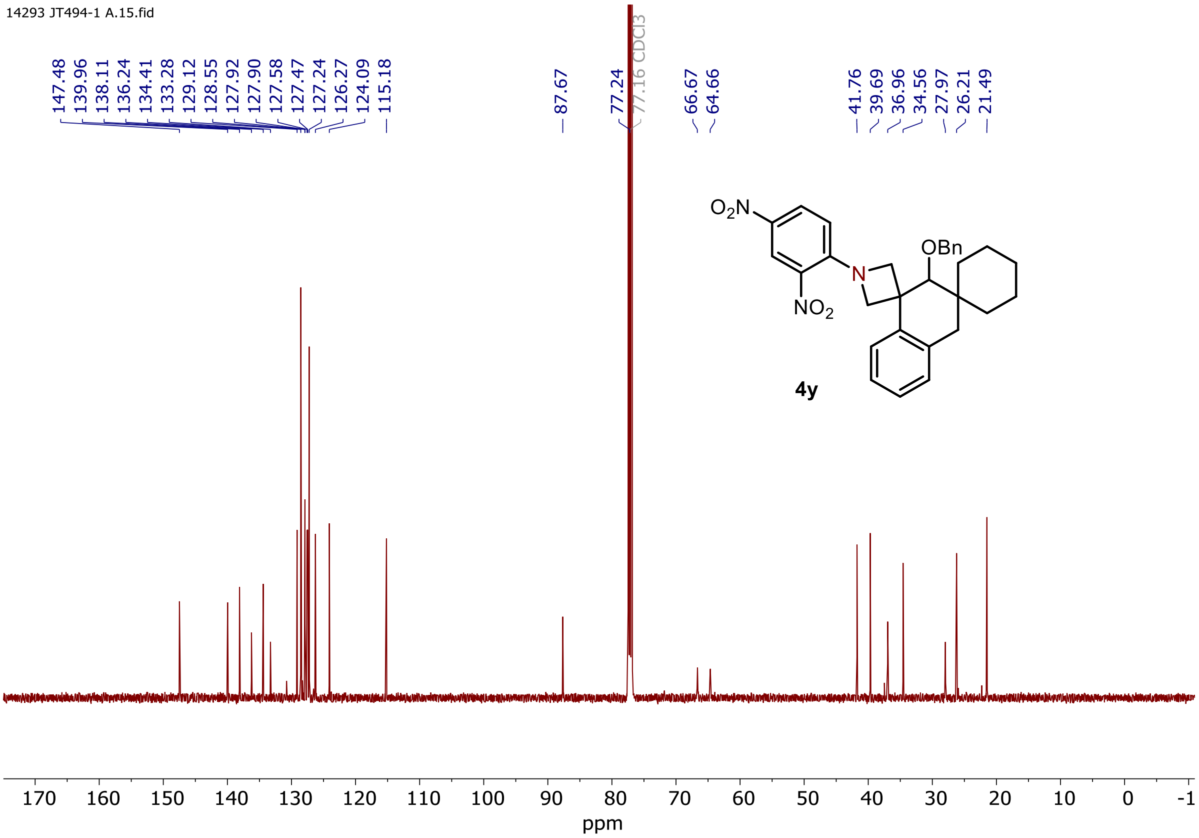

^{1}H NMR (500 MHz, $CDCl_3$) of **4a′** (<u>*see procedure*</u>)

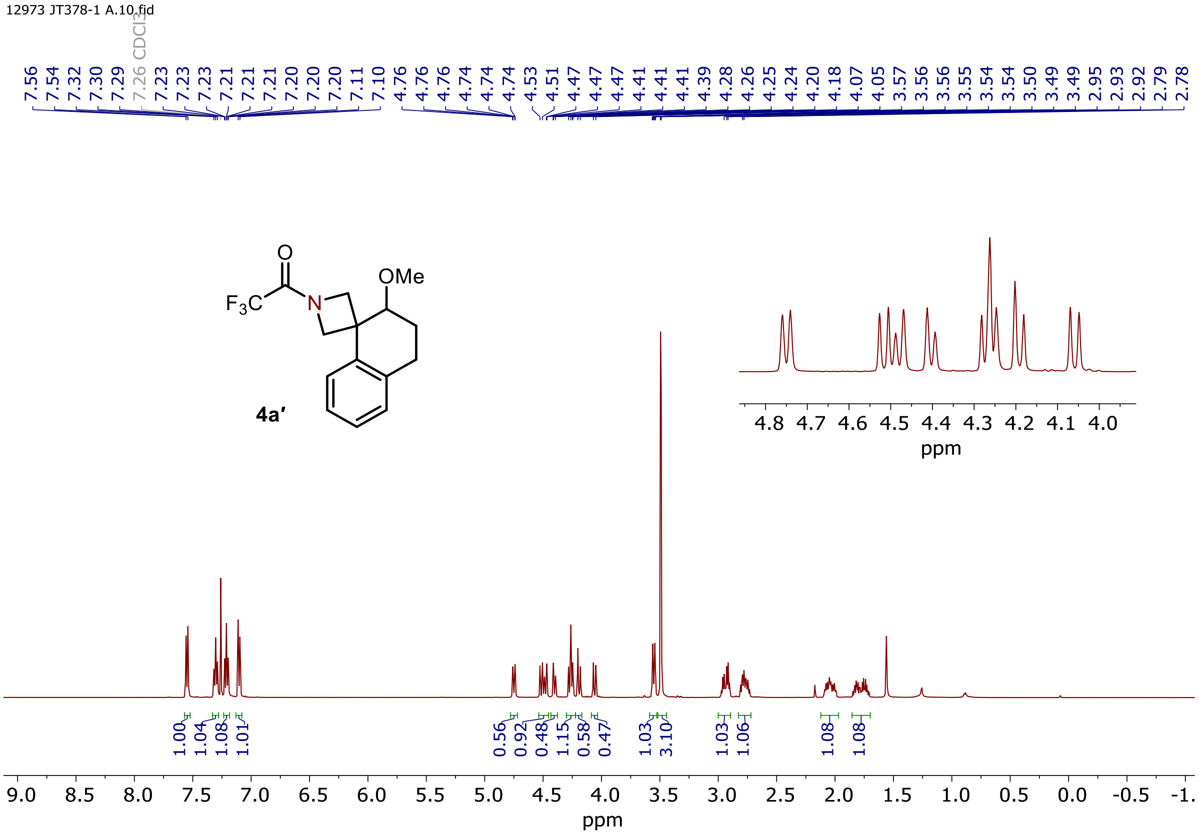

^{13}C NMR (126 MHz, $CDCl_3$) of **4a′**

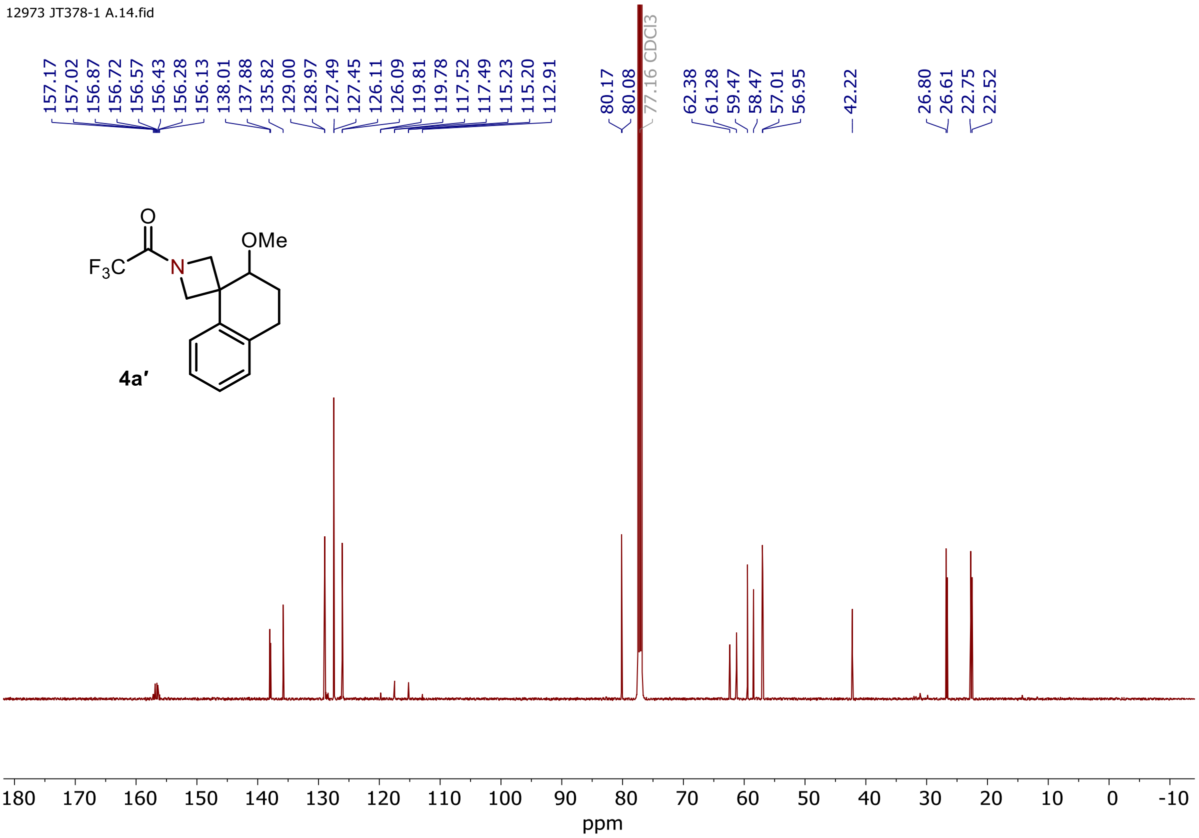

^{19}F NMR (376 MHz, $CDCl_3$) of **4a′**

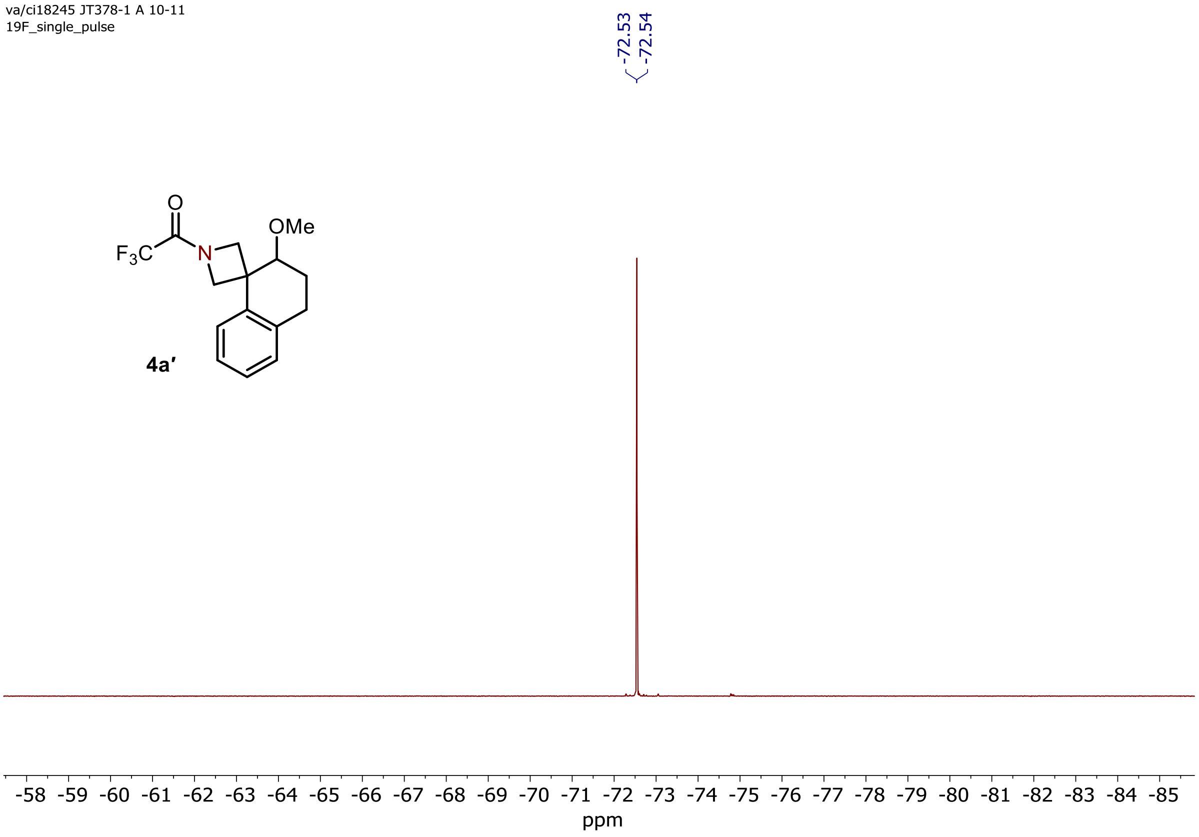

^{1}H NMR (500 MHz, $CDCl_3$) of **3j** (*see procedure*)

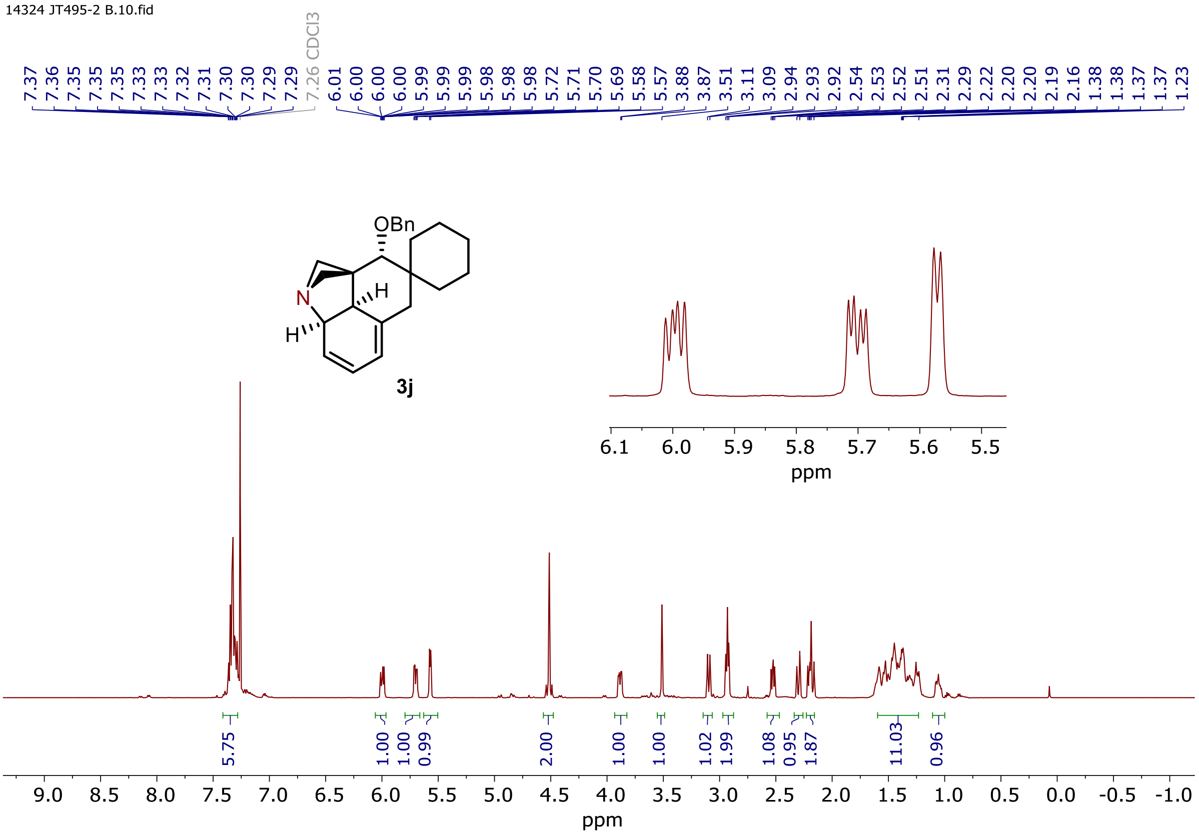

^{13}C NMR (126 MHz, $CDCl_3$) of **3j**

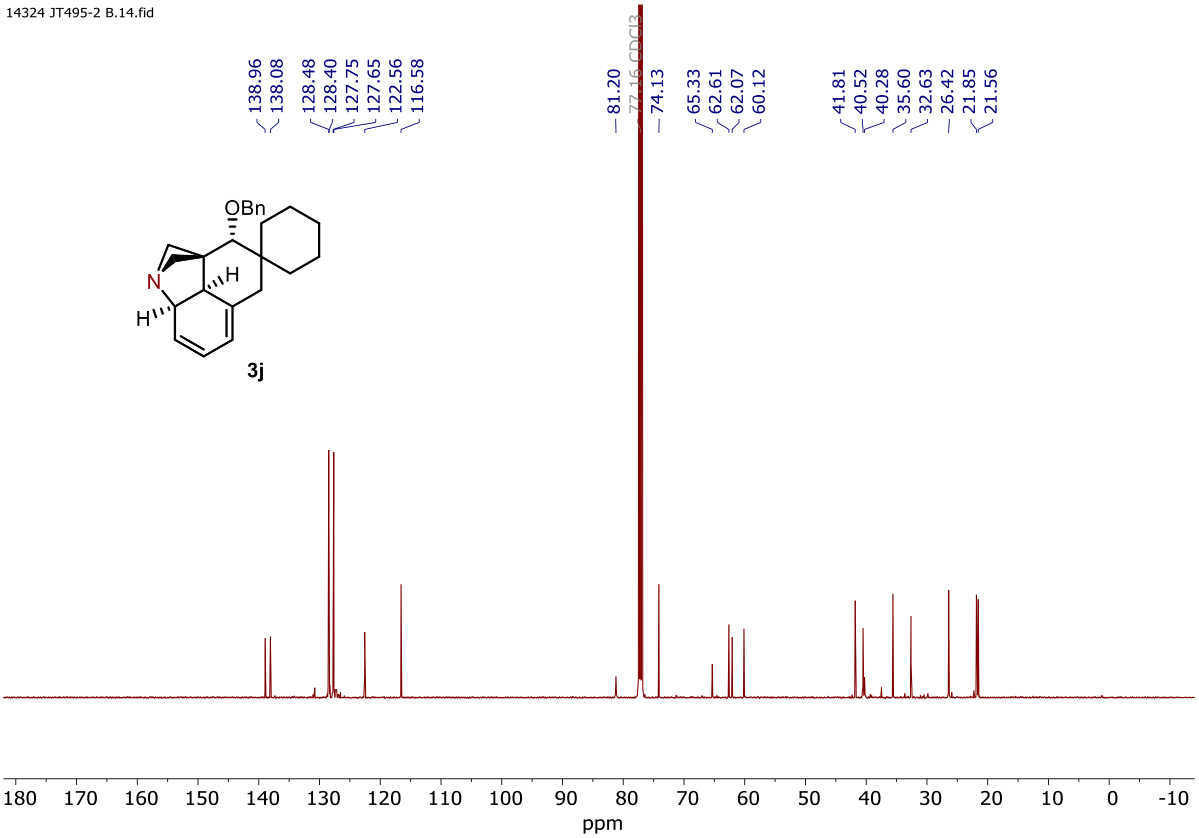

^{1}H NMR (500 MHz, $CDCl_3$) of **8m** (*see procedure*)

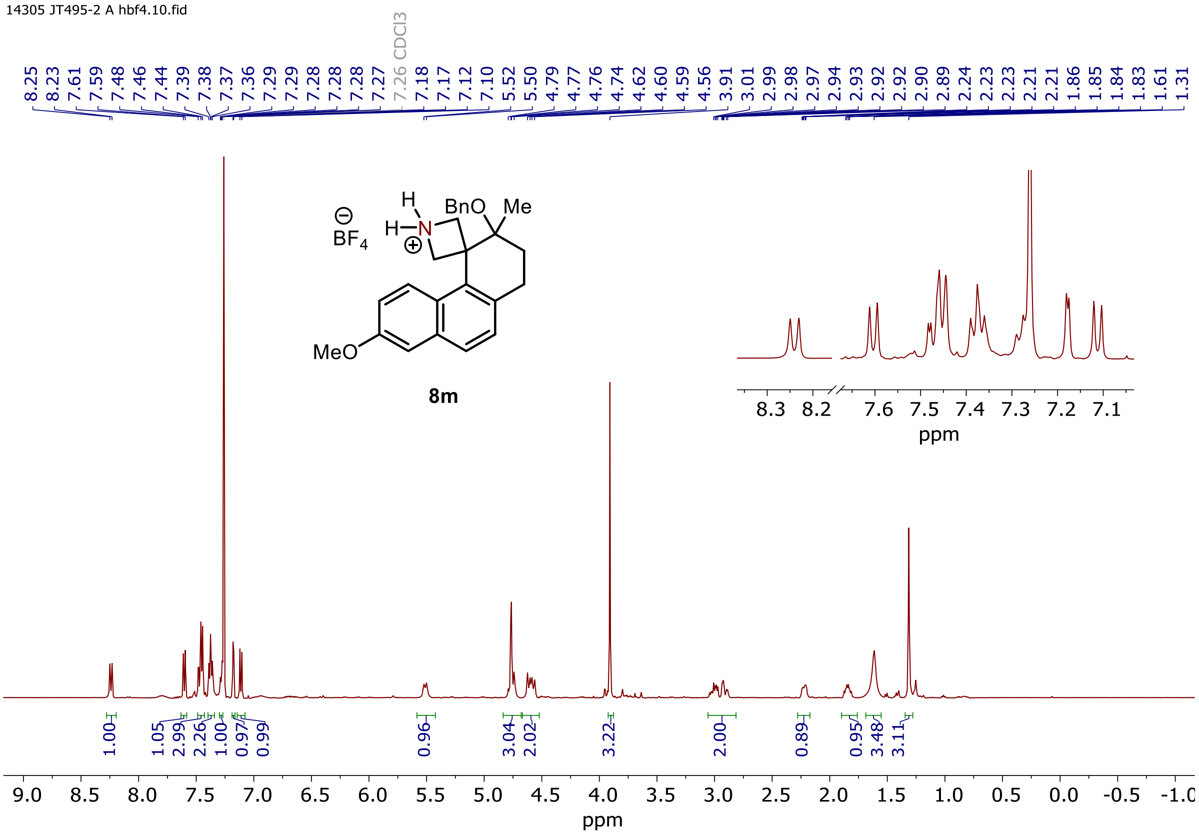

^{13}C NMR (126 MHz, $CDCl_3$) of **8m**

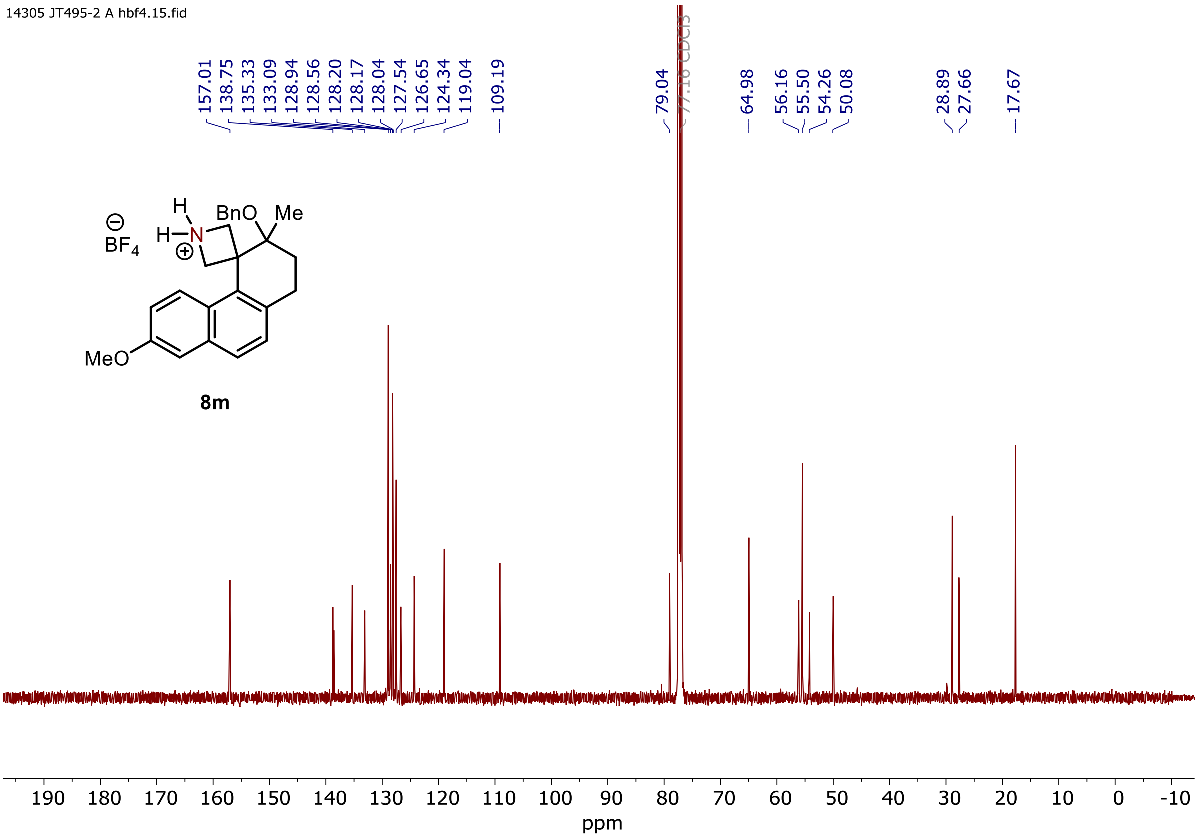

^{19}F NMR (376 MHz, $CDCl_3$) of **8m**

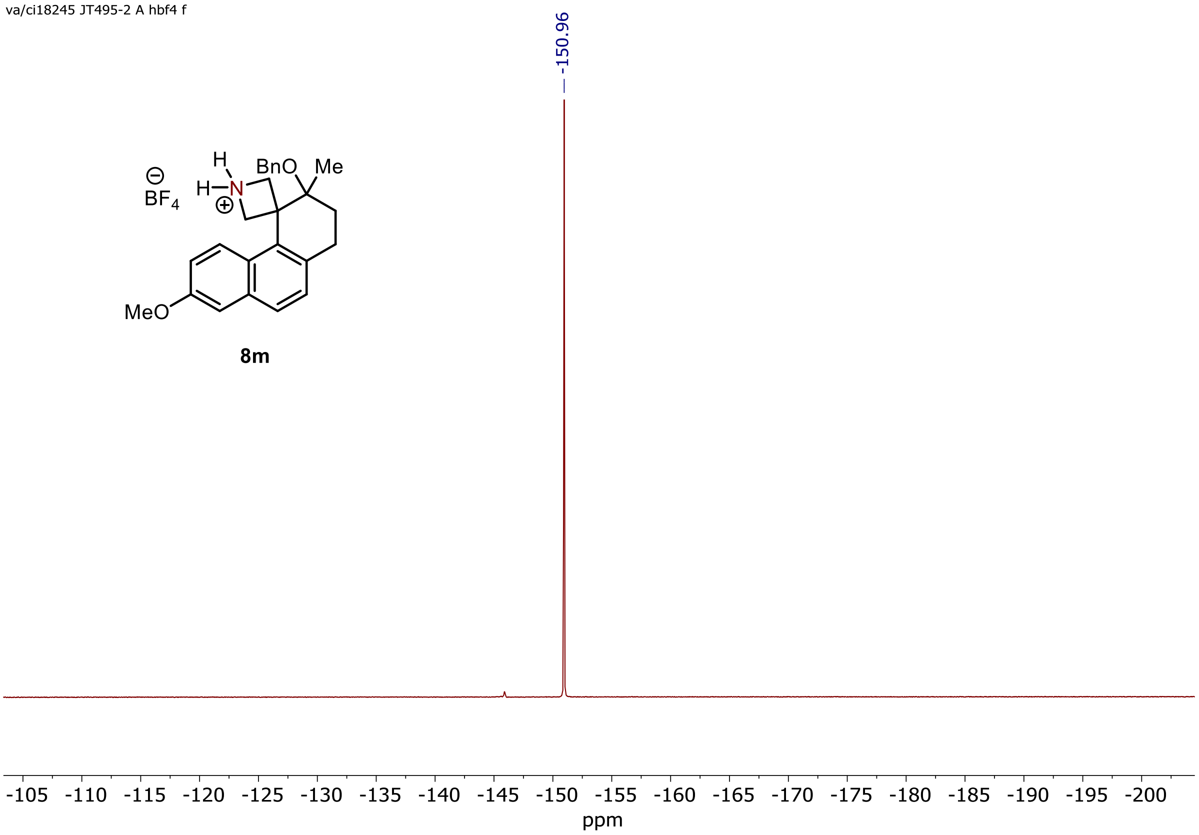

^{1}H NMR (500 MHz, CDCl$_3$) of **3p** (*see procedure*)

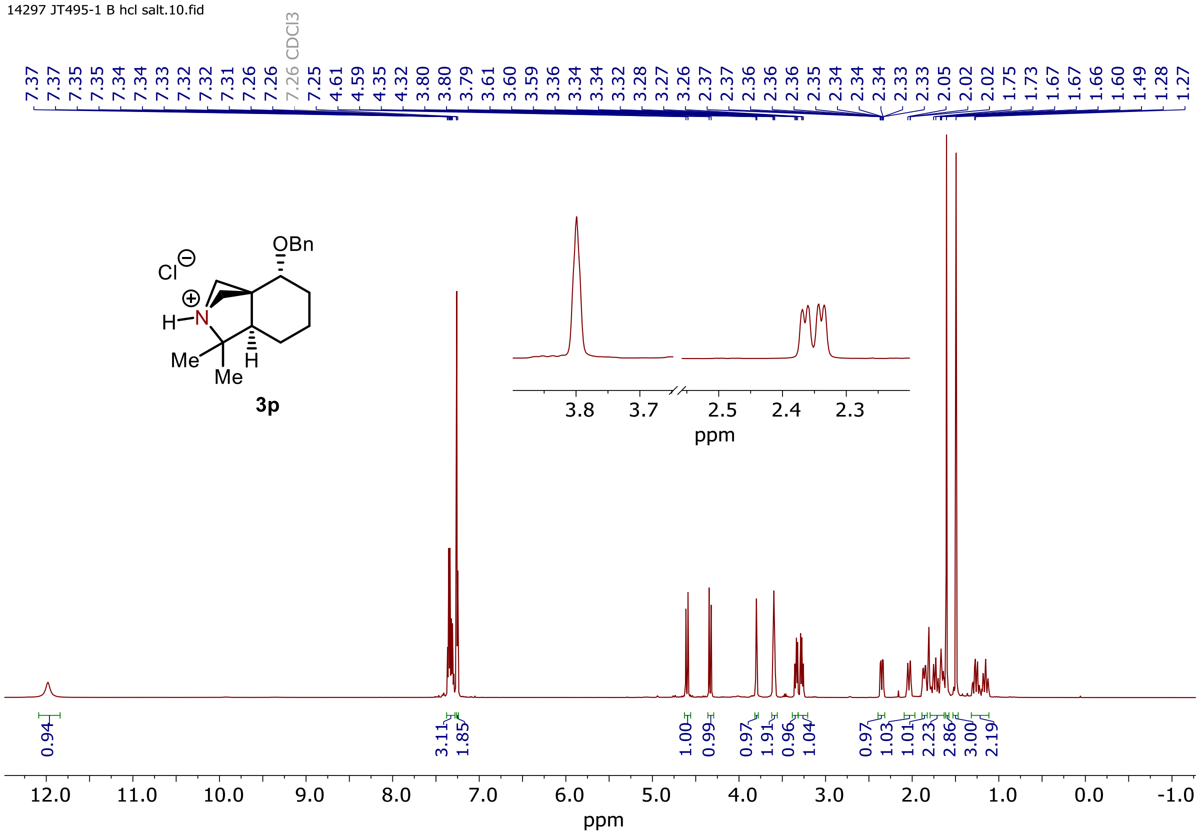

^{13}C NMR (126 MHz, CDCl$_3$) of **3p**

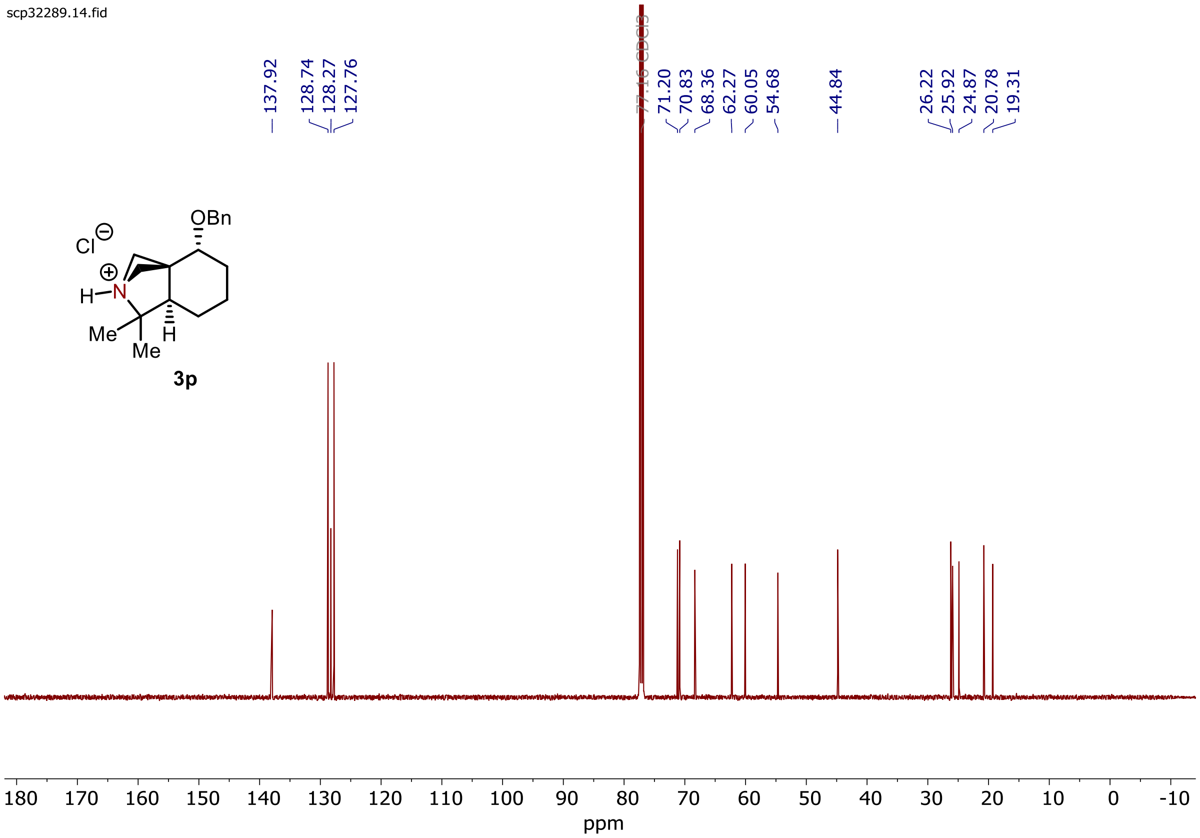

^{1}H NMR (400 MHz, $CDCl_3$) of **5b** (*see procedure*)

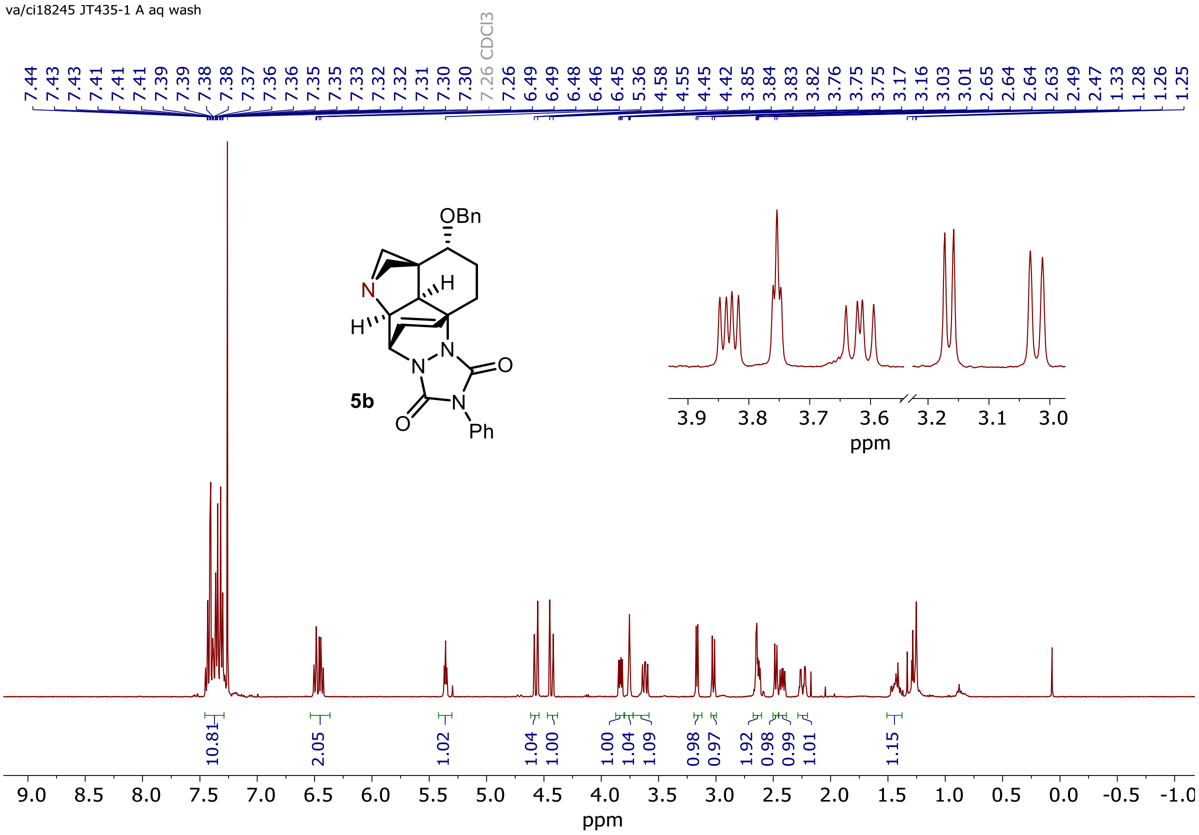

^{13}C NMR (126 MHz, $CDCl_3$) of **5b**

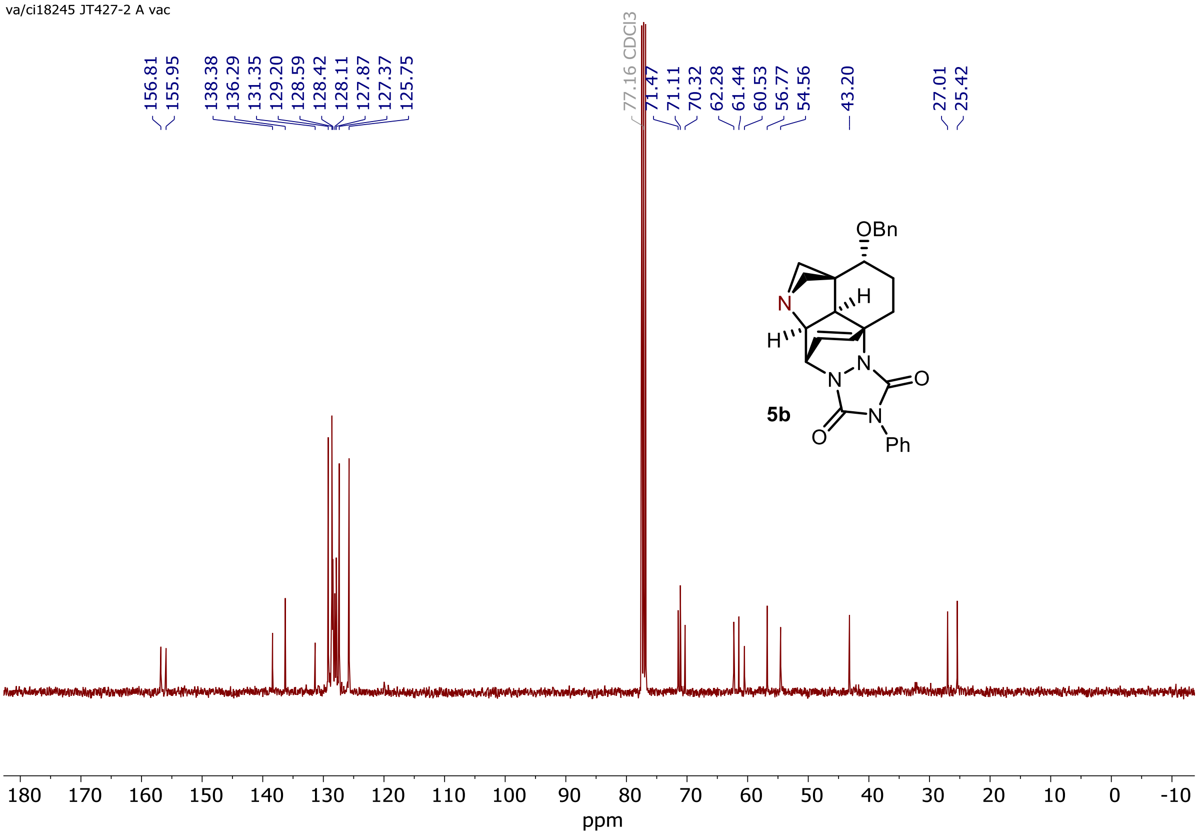

^{1}H NMR (500 MHz, $CDCl_3$) of **5d** (*see procedure*)

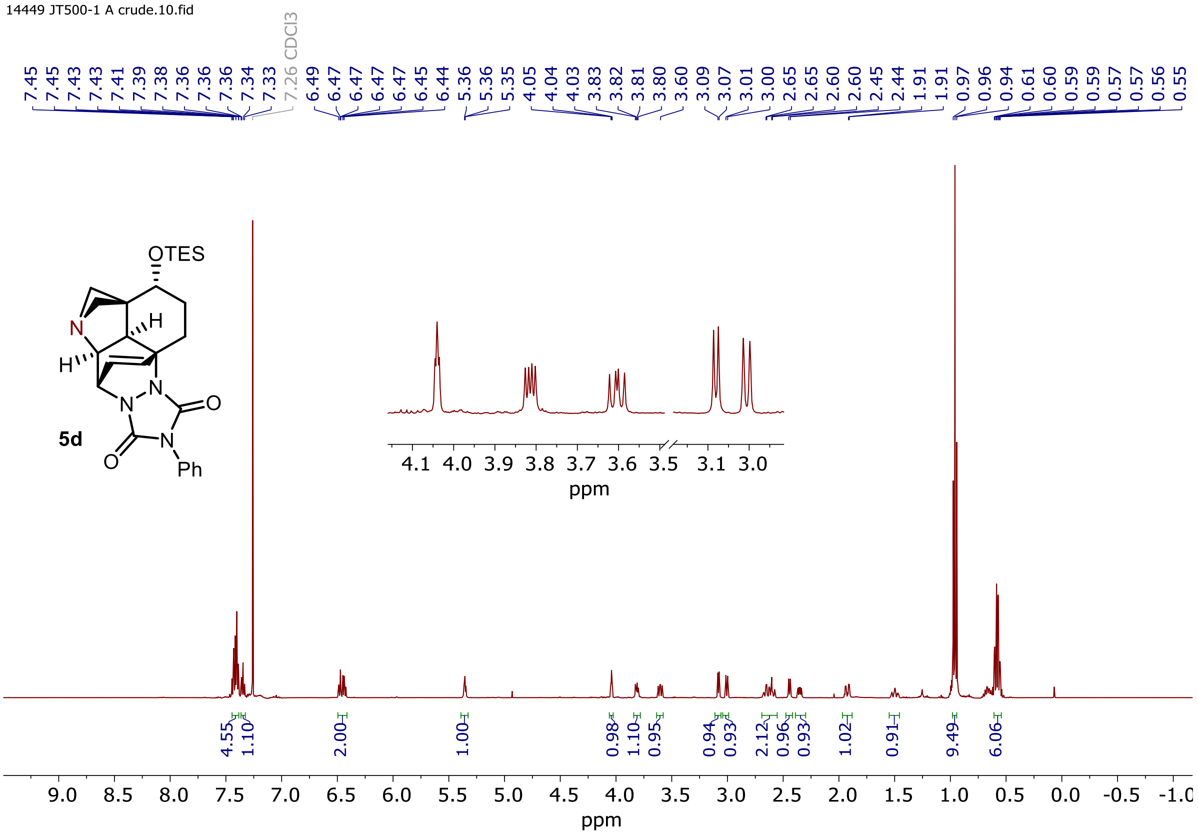

^{13}C NMR (126 MHz, $CDCl_3$) of **5d**

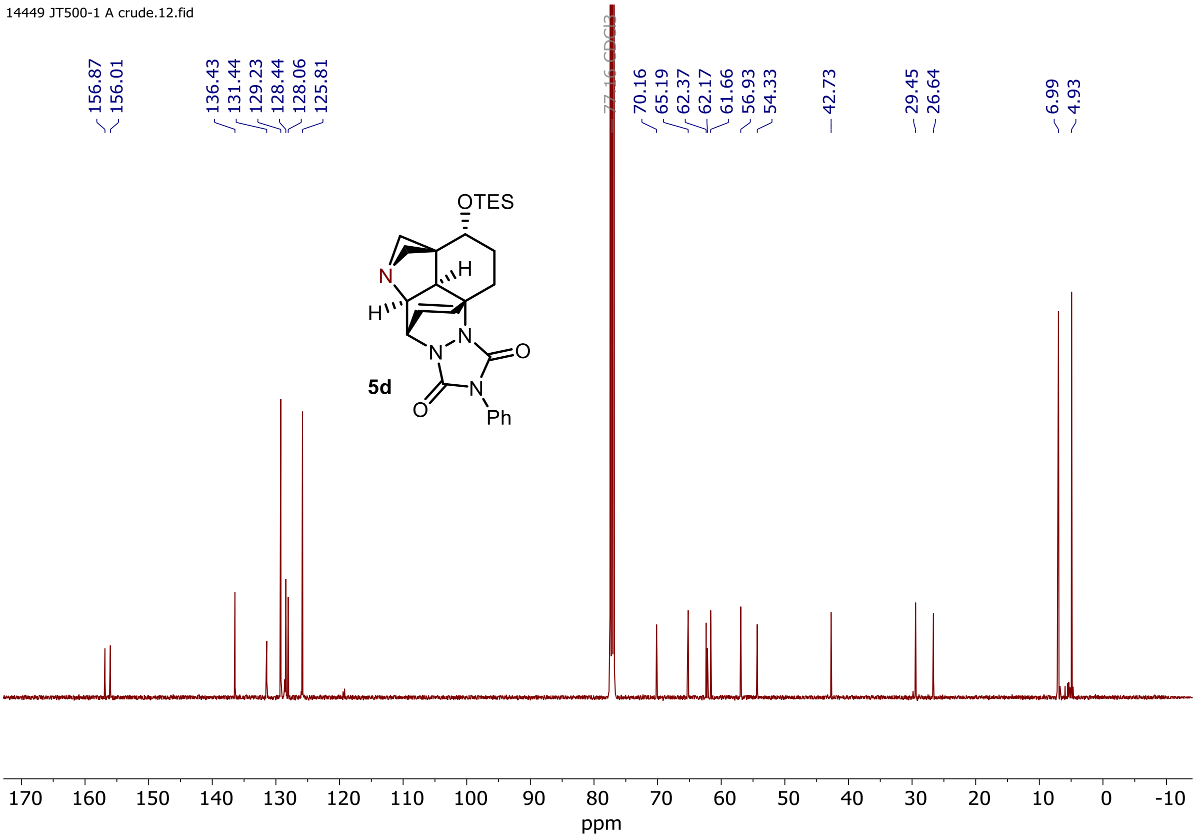

^{1}H NMR (400 MHz, $CDCl_3$) of **5f** (*see procedure*)

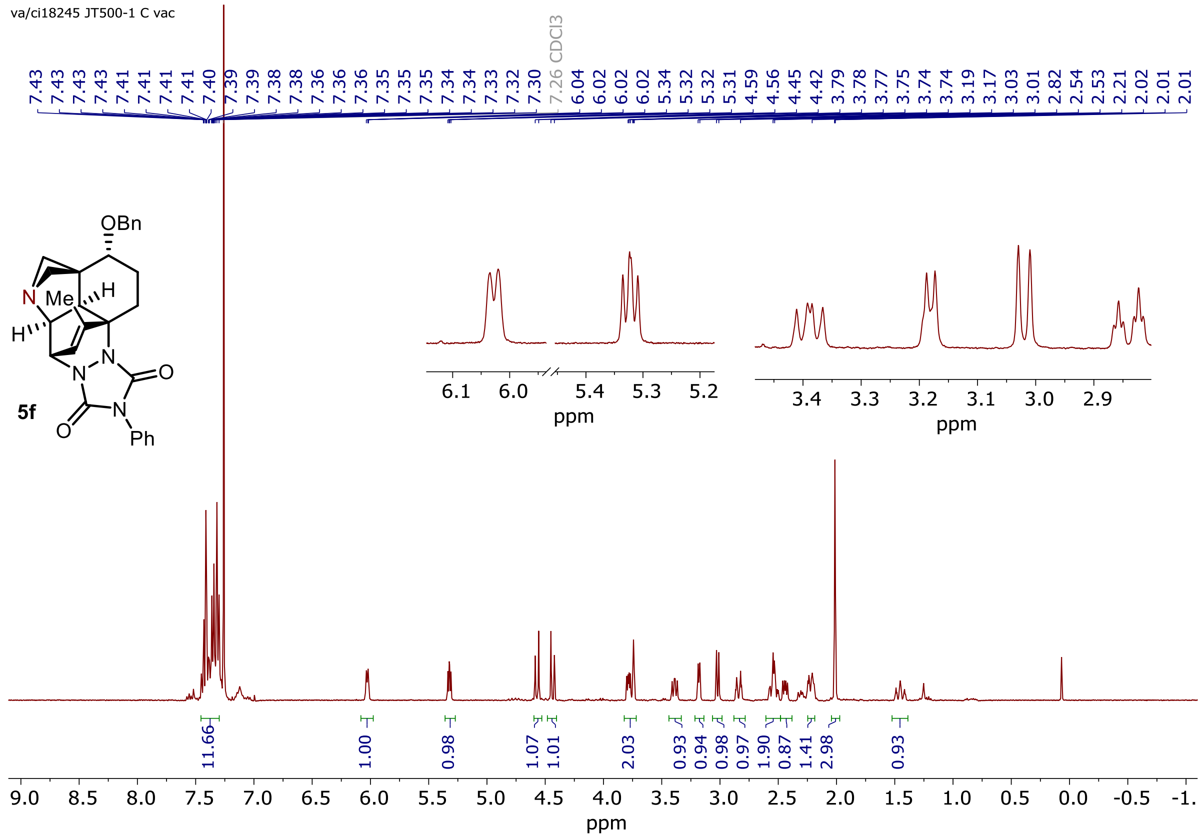

^{13}C NMR (126 MHz, $CDCl_3$) of **5f**

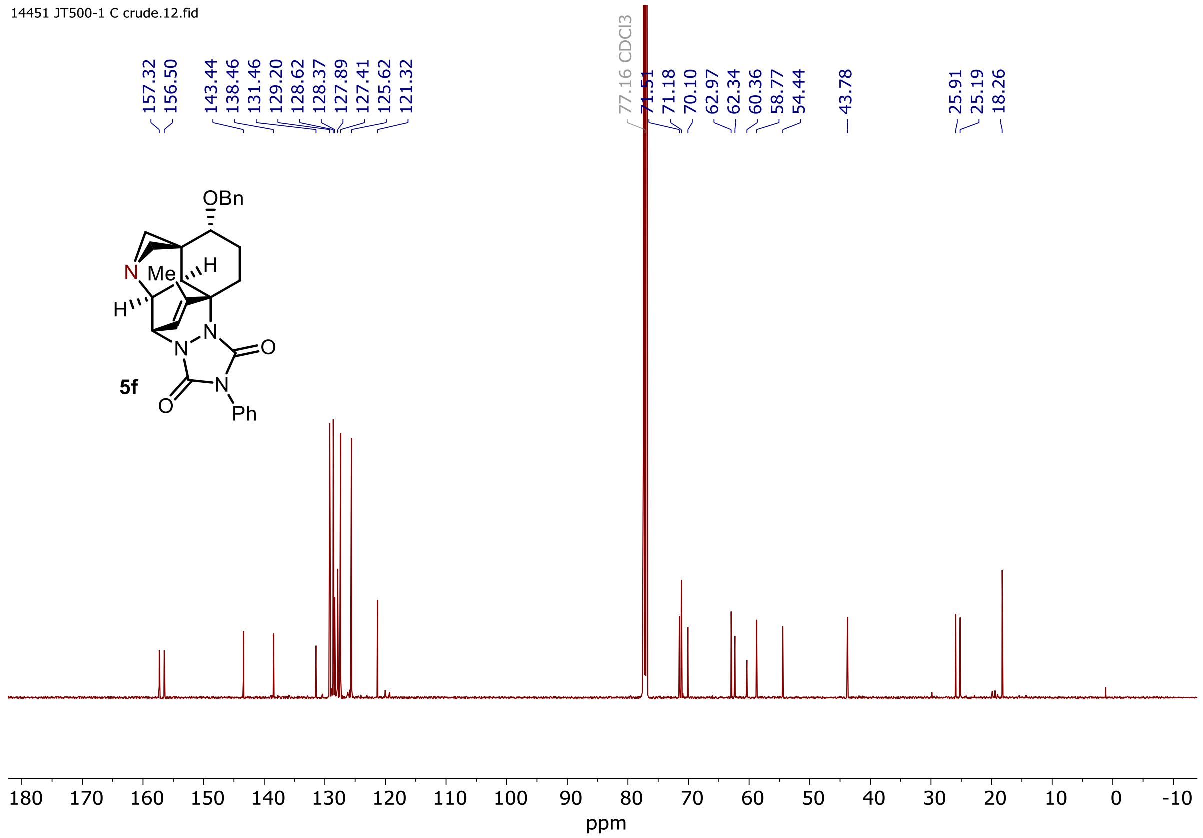

^{1}H NMR (400 MHz, $CDCl_3$) of **5j** (*see procedure*)

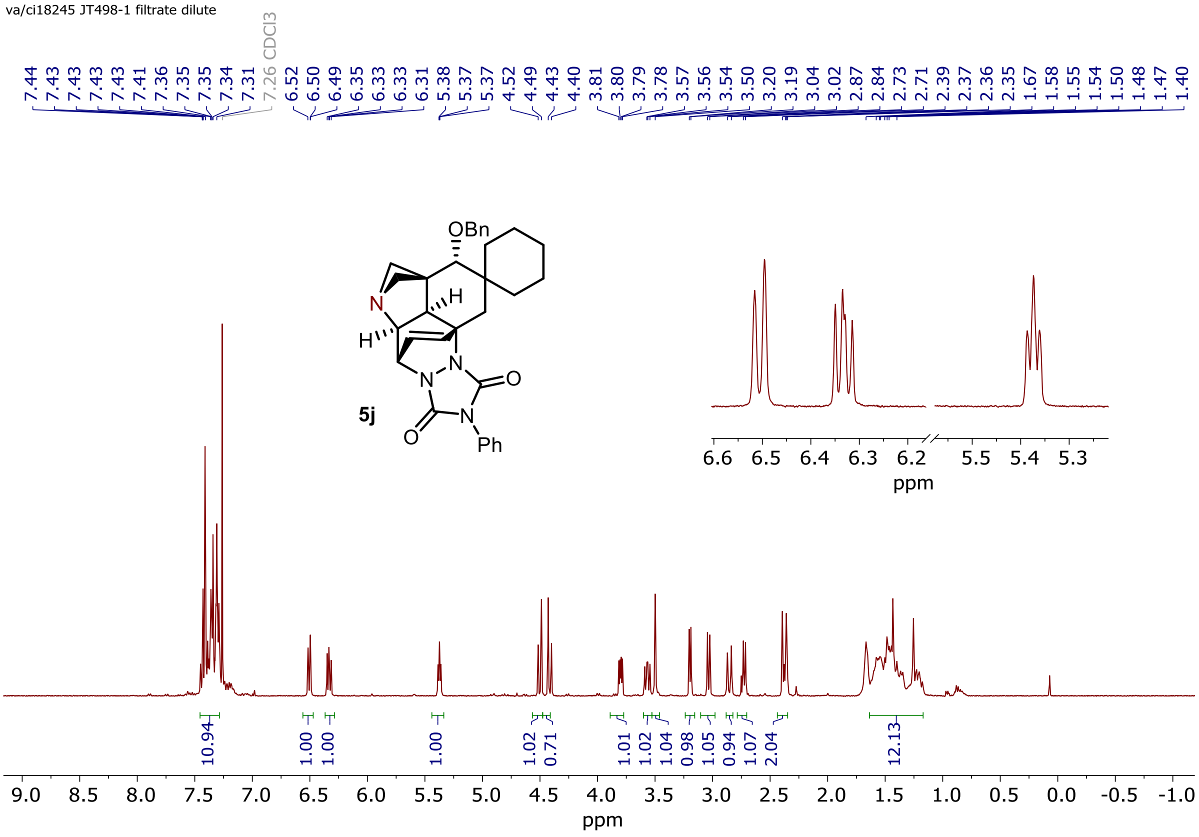

^{13}C NMR (126 MHz, $CDCl_3$) of **5j**

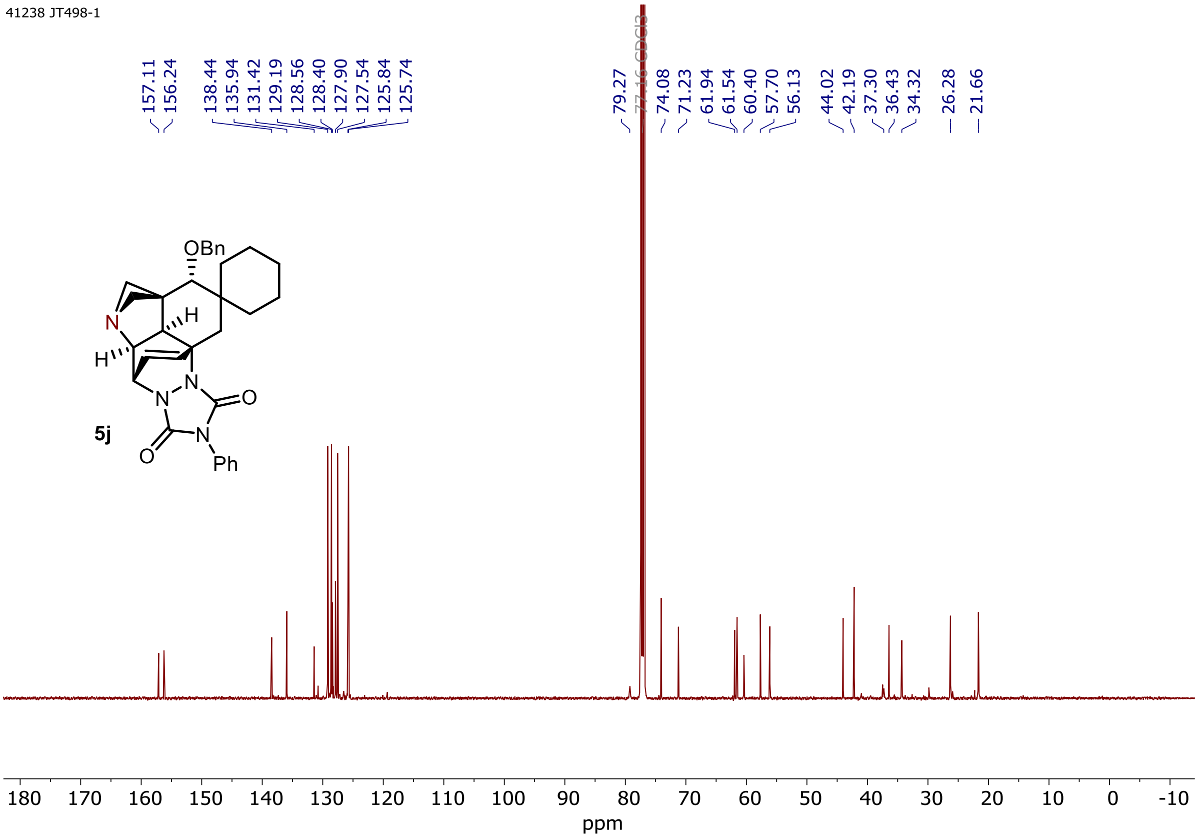

^{1}H NMR (400 MHz, $CDCl_3$) of crude **3b** (*see procedure*)

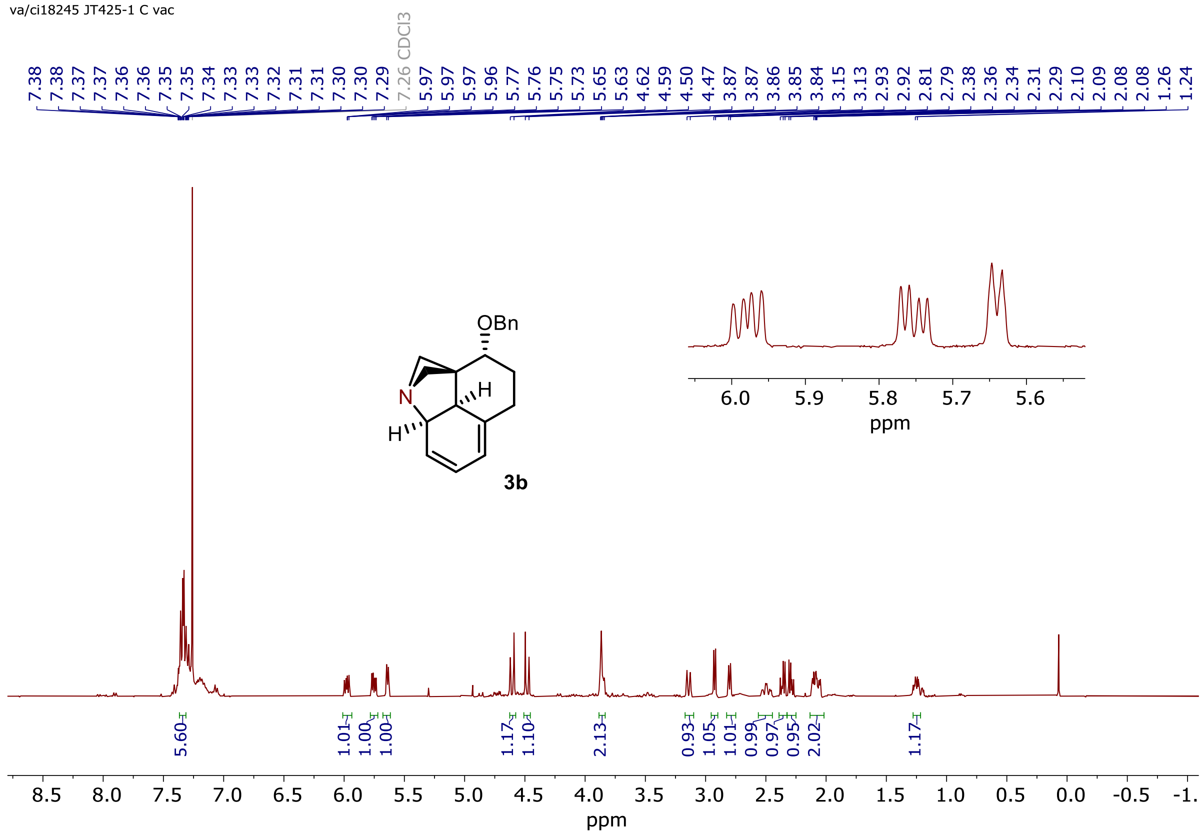

^{13}C NMR (126 MHz, $CDCl_3$) of crude **3b**

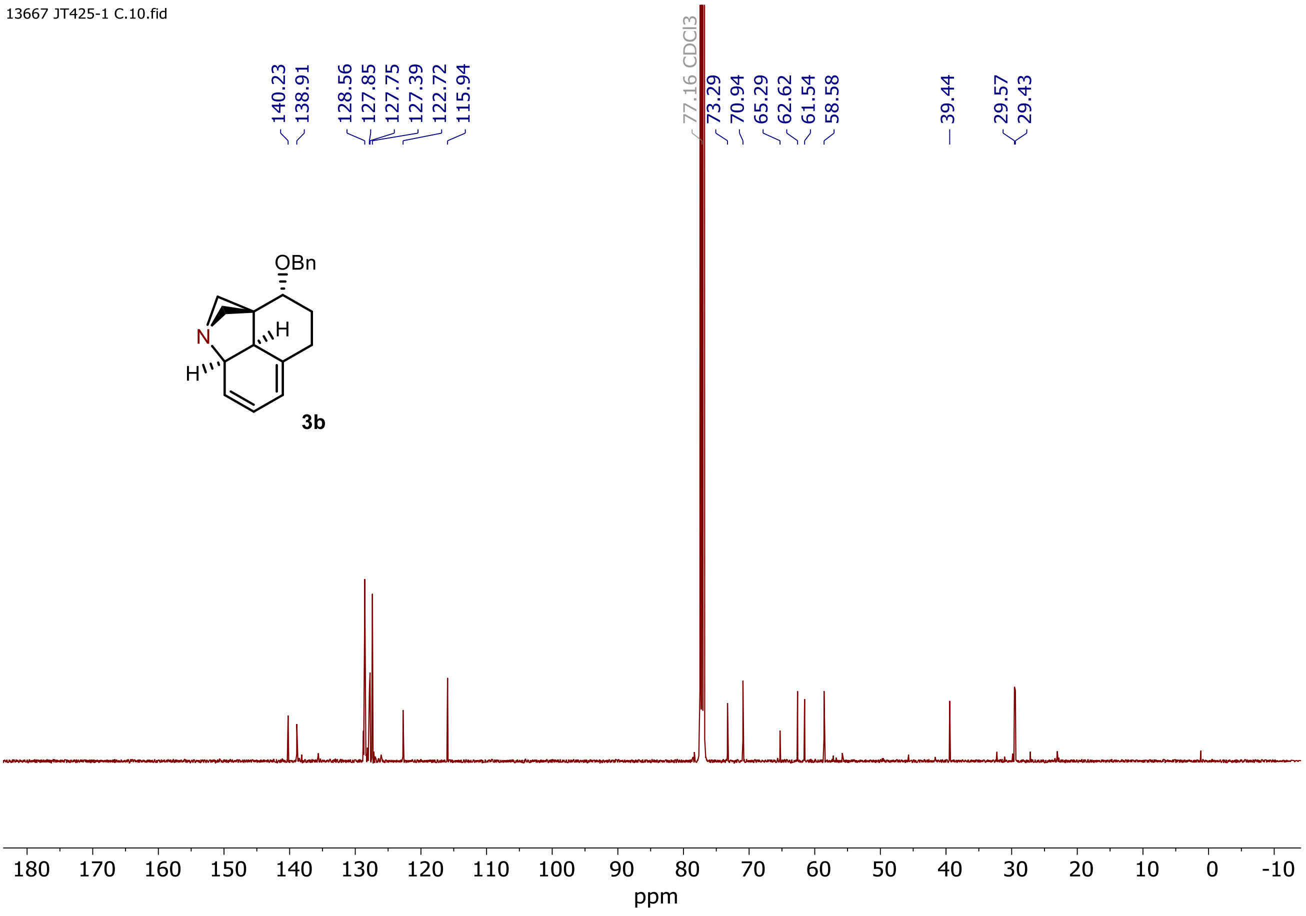

6. REFERENCES

1) Burchat, A. F.; Chong, J. M.; Nielsen, N. *J. Organomet. Chem.* **1997**, *542*, 281–283.

2) Ivkovic, J.; Lembacher-Faduma, C.; Breinbauer, R. *Org. Biomol. Chem.* **2015**, *13*, 10456–10460.

3) Musci, P.; von Keutz, T.; Belaj, F.; Degennaro, L.; Cantillo, D.; Kappe, C. O.; Luisi, R. *Angew. Chem. Int. Ed.* **2021**, *60*, 6395−6399.

4) Houjeiry, T. I.; Poe, S. L.; McQuade, D. T. *Org. Lett.* **2012**, *14*, 4394–4397.

5) Qiu, W.-W.; Surendra, K.; Yin, L.; Corey, E. J. *Org. Lett.* **2011**, *13*, 5893–5895.

6) Cinelli, M. A.; Li, H.; Chreif, G.; Martáse, P.; Roman, L. J.; Poulos, T. L.; Silverman, R. B. *J. Med. Chem.* **2014**, *57*, 1513–1530.

7) Qiu, G.; Mamboury, M.; Wang, Q.; Zhu. Z. *Angew. Chem. Int. Ed.* **2016**, *55*, 15377–15381.

8) Rasu, L.; John, J. M.; Stephenson, E.; Endean, R.; Kalapugama, S.; Clément, R.; Bergens, S. H. *J. Am. Chem. Soc.* **2017**, *139*, 3065–3071.

9) Shang, Y.; Jie, X.; Jonnada, K.; Zafar, S. N.; Su, W. *Nat. Commun.***2017**, *8*, 2273.

10) Wang, M.-M.; Ning, X.-S.; Qu, J.-P.; Kang, Y.-B. *ACS Catal.* **2017**, *7*, 4000–4003.

11) Paraja, M.; Matile, S. *Angew. Chem. Int. Ed*. **2020**, *59*, 6273–6277.

12) Speck, K.; Karaghiosoff, K.; Magauer, M. *Org. Lett.* **2015**, *17*, 1982–1985.

13) Baumgartner, Y.; Baudoin, O. *ACS Catal.* **2020**, *10*, 10508–10515.

14) Bruker, *SAINT+ v8.39.0 Integration Engine, Data Reduction Software, Bruker Analytical X-ray Instruments Inc., Madison, WI, USA*, 2018.

15) Bruker, *SADABS 2018, Bruker AXS area detector scaling and absorption correction, Bruker Analytical X-ray Instruments Inc., Madison, Wisconsin, USA*, 2018.

16) Sheldrick, G. M. *Acta Crystallogr. A* **2015**, *71*, 3–8.

17) Sheldrick, G. M. *Acta Crystallogr. A* **2008**, *64*, 112–122.

18) Sheldrick, G. M. *Acta Crystallogr. C* **2015**, *71*, 3–8.

19) Dolomanov, O. V.; Bourhis, L. J.; Gildea, R. J.; Howard J. A. K.; Puschmann, H. *J. Appl. Crystallogr.* **2009**, *42*, 339–341.
